# SNPs Meet CNVs in Genome-Wide Association Studies: HGV2007 Meeting Report

**DOI:** 10.1371/journal.pgen.1000068

**Published:** 2008-04-25

**Authors:** Xavier Estivill, Nancy J. Cox, Stephen J. Chanock, Pui-Yan Kwok, Stephen W. Scherer, Anthony J. Brookes

**Affiliations:** 1 Genes and Disease Program, Center for Genomic Regulation (CRG-UPF), Barcelona, Spain; 2 Public Health and Epidemiology Network Biomedical Research Center (CIBERESP), Barcelona, Spain; 3 Departments of Medicine and Human Genetics, University of Chicago, Chicago, Illinois, United States of America; 4 Division of Cancer Epidemiology and Genetics and Center for Cancer Research, National Cancer Institute, Bethesda, Maryland, United States of America; 5 Department of Dermatology, Cardiovascular Research Institute, University of California San Francisco, California, United States of America; 6 Institute for Human Genetics, University of California San Francisco, California, United States of America; 7 The Centre for Applied Genomics, Program in Genetics and Genomic Biology, Research Institute, The Hospital for Sick Children, Toronto, Ontario, Canada; 8 Department of Genetics, University of Leicester, Leicester, United Kingdom; The University of Queensland, Australia

The Ninth Meeting on Human Genome Variation and Complex Genome Analysis was held in Sitges, Spain, in September 2007. This annual meeting, which originally focused on single nucleotide polymorphisms (SNPs), broadened its scope from 2006 onward to encompass the entire range of genomic variability. Maintaining the relatively small format of 200 delegates, the meeting gathered leading investigators in copy number variation (CNV), SNP association studies, ultrasequencing, population genetics, statistical analysis, and database management, as well as young investigators who initiated careers in these fields. The two-and-a-half-day meeting combined sponsorship by several academic institutions and corporate entities in a venue that facilitated interaction and communication between the participants.

The Sitges venue was particularly conducive to the formal and informal discussions that have long characterized this meeting. These discussions were wide-ranging, and while they often began in the meeting rooms, they usually continued through meals and into the evening.

A key focus was the question of how to facilitate the continued success of genome-wide association studies (GWAS). It has been widely appreciated that large-scale collaboration has been hugely beneficial to early GWAS, and there was considerable discussion at the meeting of the need to establish and maintain the bioinformatics infrastructure necessary to make maximum use of the data being generated. While initial efforts toward building such infrastructure in the United States and Europe have made impressive inroads in serving both data and results of GWAS (summarized by a number of the participants—see below), the need for richer resources allowing integration of GWAS across more phenotypes, including expression phenotypes from multiple human tissues, was apparent. Such resources would allow more rapid and broad-based assessment of functional relationships among genetic variations (SNP and CNV), expression phenotypes, disease states, and related quantitative traits. There was also widespread sentiment that larger samples sizes, while clearly desirable, would, in the end, provide only a fraction of the contribution of genetic variation to complex disorders. Advances in analytic approaches coupling statistical genetics with bioinformatics may prove to be fruitful in extending results of GWAS.

There was vigorous discussion of the likely contribution of structural variation to human genetic disease. Although a number of participants confirmed the contribution of CNVs to various human disorders, known CNVs are highly skewed toward the lower end of the minor allele frequency spectrum. This skewing was thought to reflect an initial bias toward detection of larger CNVs; a discovery bias which precludes a comprehensive understanding of the contribution of this class of genetic variants to human disease and reduces the likelihood of being able to reliably “tag” CNVs even if most were the consequence of a single (rather than recurrent) events. As technology improvements allow detection of smaller CNVs that may have a higher minor allele frequency as well as more precise delineation of the exact sequences involved, we will not only get a more accurate picture of the contribution of these variants to disease but also gain insights into the dynamics, the evolutionary history, and the consequences of such variants. A key question is whether such sites are generally uniquely created, with a single originating event, or rather are commonly regenerated, due perhaps to the presence of repetitive elements. This will determine whether such variants can be tagged and indirectly interrogated (e.g., through imputation) or will need to be directly interrogated, which will in turn influence the design of later generation platforms for GWAS.

Technology, particularly the newest sequencing technologies, was also a major topic of discussion. A number of new approaches as well as more streamlined versions of existing technologies were discussed. The meeting certainly highlighted the steady steps toward the eventual goal of sequencing entire human genomes at reasonable financial cost and with efficient computational algorithms.

Abstracts of the meeting and an expanded version of the meeting report can be found on the meeting Web sites (http://hgv2007.nci.nih.gov; http://www.tcag.ca/hgv2008) and in Text S1 (abstracts) and Text S2 (meeting report).

Highlights of the presentations include the considerable progress reported by **Stephen Chanock** and colleagues at the National Cancer Institute (Bethesda, Maryland, United States) in recent months on the identification of genetic variants that predispose to common human cancers; the studies were based on a stepwise approach used in GWAS followed by meta-analysis on data from several other groups. **Xavier Estivill** (Center for Genomic Regulation, Barcelona, Spain) reported a common genomic feature of disorders for which CNVs have been detected, namely the presence of segmental duplications in the vicinity. Moreover, all CNV loci that have been found associated with common disorders are both complex and multi-allelic, making it difficult to tag these CNVs with SNPs. **Pui-Yan Kwok** (University of California San Francisco, California, United States) sounded a cautionary note regarding quality control of genotype data in the context of automated genotype data production.

In the area of sequencing the individual genome, one of the most challenging problems is the assembly of the sequences and the large number of differences between sequences, including many structural variation changes. **Samuel Levy** (J. Craig Venter Institute, Rockville, Maryland, United States) reported the details of the sequencing, assembly, and variant detection in the genome of Craig Venter. Using newly developed genome assembly strategies and comparative genome-to-genome mapping methods, they identified 25 Mb of diploid sequence differences, representing more than 4 million DNA variants, thereby increasing the estimate of DNA sequence differences between unrelated humans to 5–10 times more than previously thought. **Sanjeev Bhaskar** (Wellcome Trust Sanger Institute, Hinxton, United King) and **Ivo Gut** (Centre National de Génotypage, Evry Cedex, France) described their efforts in high-throughput, targeted sequencing using a variety of approaches. **George Church** (Harvard Medical School and Massachusetts Institute of Technology, Boston, Massachusetts, United States) showed that 1% of the genome harboring most causative alleles for medical and nonmedical traits could be targeted for sequencing using strategies he and others have developed. He pointed out that by combining these approaches with paired-end tags for rearrangements (such as those described by **Jan Korbel** at Yale University, New Haven, Connecticut, United States) and allele-specific RNA quantification, an affordable analysis of the human genome could be achieved at the individual level.

The intensity of CNV research was evident in the presentations of several groups at the HGV2007 meeting. For example, **Steve Scherer** (Hospital for Sick Children, Toronto, Ontario, Canada) reported the recent findings on chromosome rearrangements and imbalances in autism spectrum disorders, with evidence showing that chromosome rearrangements in autism are likely to be involved in 10%–20% of all cases. **Barbara Trask** (Fred Hutchinson Cancer Research Center, Seattle, Washington, United States) reported on the important role CNVs played in the evolution of three families of chemosensory receptors (olfactory receptors and two classes of vomeronasal receptors [V1Rs and V2Rs]) that help an organism interact with its environment. **George Perry** and **Charles Lee** (Brigham and Women's Hospital, Boston, Massachusetts, United States) presented data about the distribution of amylase gene (*AMY1*) copies in different populations that showed a positive or directional selection on *AMY1* copy number in human populations with diets high in starch but neutral evolution on *AMY1* copy number in low-starch populations. **Joris Veltman** (Radboud University Nijmegen Medical Centre, Nijmegen, The Netherlands) presented the use of dense bacterial artificial chromosome (BAC) arrays and SNP arrays to identify CNVs underlying mental retardation. The use of parallel approaches and data sharing by investigators from different countries has allowed them to identify new syndromes that were previously unrecognized. **Matthew Hurles** (The Wellcome Trust Sanger Institute, Hinxton, United Kingdom) reported on the use of ultrasequencing technologies to identify and characterize structural variation. He also presented data on the development of a comprehensive map for common CNVs using high-density oligonucleotide arrays with 42 million probes across the genome. Finally, he stressed the need for improved methods for CNV genotyping and quantification to deal with multiallelic CNVs and with differential biases in assessing CNVs in cases and controls. To facilitate the precise quantification of copy numbers of particular genes in subjects, **John Armour** (University of Nottingham, Nottingham, United Kingdom) described the development of paralogue ratio tests (PRTs) that improve the precision, economy, and throughput for complex CNV genotyping.

Combining CNV and SNP data in GWAS is a major challenge for statistical geneticists, and a number of groups presented strategies to tackle this problem. **Nancy Cox** (University of Chicago, Chicago, Illinois, United States) reviewed general approaches for direct and indirect assessment of CNV information to study common disorders. She reported on the use of TUNA (Testing Untyped Alleles) to utilize linkage disequilibrium (LD) to interrogate CNVs for which multilocus LD tags can be constructed. **Don Conrad** (University of Chicago, Chicago, Illinois, United States) described new methodology for integrating CNVs into the study of genetic traits. **Iuliana Ionita** (Harvard University, Boston, Massachusetts, United States) reported on the development of an extension of a family-based association test (FBAT) approach to analyze CNV data with family-based designs.

**Vivian Cheung** (University of Pennsylvania, Philadelphia, Pennsylvania, United States) reported on research to identify genetic variation affecting interindividual gene expression. Of 3,500 genes with variable expression levels, 235 were associated with SNPs (80% in *trans*, 5% in *cis*, and 15% with multiple effects) in a GWAS. **Manolis Dermitzakis** (Wellcome Trust Sanger Institute, Hinxton, United Kingdom presented data on the widespread genetic variation in mRNA levels of many genes across populations. Moreover, many detected associations between gene expression levels and SNPs are shared across human populations, and that signal is concentrated, within 100 kb from the promoter, symmetrically around transcription start sites.

**Chris Ponting** (University of Oxford, Oxford, United Kingdom) discussed the elevated density of genes, evolutionary rates, and gene functions, noting data consistent with the possibility that some of these regions have been positively selected in the human population due to advantageous gene dosage effects of copy number variants. **Jaume Bertranpetit** (Pompeu Fabra University, Barcelona, Spain) discussed the possibilities of computing population recombination rates from SNP frequency data. They found that most of the variation is among major human groups and a minor component of population variation is within continents, with most recombination hotspots conserved among human populations. **Esteban González-Burchard** (University of California San Francisco, San Francisco, California, United States) provided fundamental evidence of genetic differences between racial and ethnic populations relevant to differences in genetic risk for Alzheimer disease and HIV resistance. **Gilles Thomas** (National Cancer Institute, Bethesda, Maryland, United States) presented data on population stratification in two genome-wide studies in breast cancer and prostate cancer. Their results showed evidence of population structure on the European continent and pointed to the need to correct for population stratification in searching for association in European populations. In addition to the GWAS approach, **Angel Carracedo** (University of Santiago de Compostela, Galicia, Spain) discussed classical approaches to identifying genetic variations associated with both toxicity and efficacy. He emphasized the challenges of the current applications in clinical practice and the changes in labeling that have been recommended by the regulatory agencies in Europe and United States (European Medicines Agency [EMEA] and Food and Drug Administration [FDA], respectively) for about ten drugs).

Another important topic of discussion at the meeting was the current status and future needs for central genomic databases in the area of human variation. **Yum Lina Yip** (Swiss Institute of Bioinformatics, Geneva, Switzerland) gave a presentation on archiving single amino acid polymorphisms in the UniProt/Swiss-Prot knowledge base, with >30,000 single amino acid polymorphisms (SAPs) in about 6,000 human proteins already archived and many more to come. **Andrew Devereau** (National Genetics Reference Laboratory, Manchester, United Kingdom) reported on the use of a variation database for diagnostic molecular laboratories. This tool allows data from different laboratories and different sources to be integrated and analyzed for the interpretation of its clinical significance. **Anthony Brookes** (University of Leicester, Leicester, United Kingdom) presented progress toward developing HGVbaseG2P, a database of genotype-to-phenotype (G2P) relationships, which aims to pull together a comprehensive view of the world's genetic association study findings. He also described GEN2PHEN (http://www.gen2phen.org/), a European Commission Integrated Project designed to help provide globally relevant solutions for G2P databasing. **Ewan Birney** (European Bioinformatics Institute, Hinxton, United Kingdom) presented an overview of the Ensembl infrastructures for genomic information, from its storage through to analysis and visualization. The data included variation information for more than 6,000 human individuals and resequencing data from six. **James Ostell** (National Library of Medicine, Bethesda, Maryland, United States) described several of the resources of the National Center for Biotechnology Information (NCBI), including the Database of Genotype and Phenotype (dbGaP), which holds phenotype data from long-term clinical and cohort studies, and is linked to large-scale genotype results on the participants or to medical sequencing data in support of GWAS. **Lincoln Stein** (Cold Spring Harbor Laboratory, Cold Spring Harbor, New York, United States) presented the new features and tools of the HapMap Web site and discussed progress toward providing views of resequencing data, particularly as it moves toward sequencing entire human genomes. **Carole Charlier** (University of Liège, Wallonia, Belgium) reported on the use of Patrocles, a database of polymorphic miRNA-mediated gene regulation that assists in the identification of SNPs that affect such regulation. **Lars Feuk** (The Hospital for Sick Children, Toronto, Ontario, Canada) described the latest version of the Database of Genomic Variants, which contains all the published CNVs in the genome. This database faces the challenges of definition of CNV boundaries, detection of false positives, and determination of accurate population frequency information.

The meeting also devoted discussion to the ethical aspects of individualized sequencing. **Anne Cambon-Thomsen** (Inserm and University Paul Sabatier Toulouse III, Toulouse, France) reviewed the issue of human biobanks for studying human genome variation. She reported on the networking of biobanks (Public Population Project in Genomics, http://www.p3gconsortium.org/; European Biobanks, http://www.biobanks.eu/) and described some of the conflicting interests that have to be balanced, such as participant privacy, potential risks and benefits, methodological guidance for interpretation and use of data, professional recognition of investigators, sharing of samples and data, intellectual property rights, and characteristics of a centralized data repository or other repository.

The Sitges meeting also included more than 150 posters presented over the three-day meeting. The prevailing view was that each attendee left the meeting with new ideas in a field that is moving rapidly at the cutting edge of discovery of the genetic variants that will define disease predisposition and help to uncover new biological pathways for understanding human health and disease. The HGV2008 meeting (http://www.tcag.ca/hgv2008/) will be held 15–17 October 2008, in Toronto, Canada. That meeting will focus, in part, on further steps for the sequencing and resequencing of the human genome.

## Supporting Information

Text S1 Meeting Abstracts: Meeting Program Booklet Including the Meeting Agenda and All Abstracts
(14.7 MB PDF)

Text S2 Full Meeting Report: An extended version of the meeting report
(0.08 MB DOC)

