# Supplementary material for: SNPs Meet CNVs in Genome-Wide Association Studies: HGV2007 Meeting Report
Source: PLoS Genet. 2008 Apr 25;4(4):e1000068. doi: 10.1371/journal.pgen.1000068 (PMC2330071; doi:10.1371/journal.pgen.1000068)
Supplement: Text S1 — Meeting Abstracts: Meeting Program Booklet Including the Meeting Agenda and All Abstracts (14.7 MB PDF) [file pgen.1000068.s001.pdf]

Human Genome Variation and Complex Genome

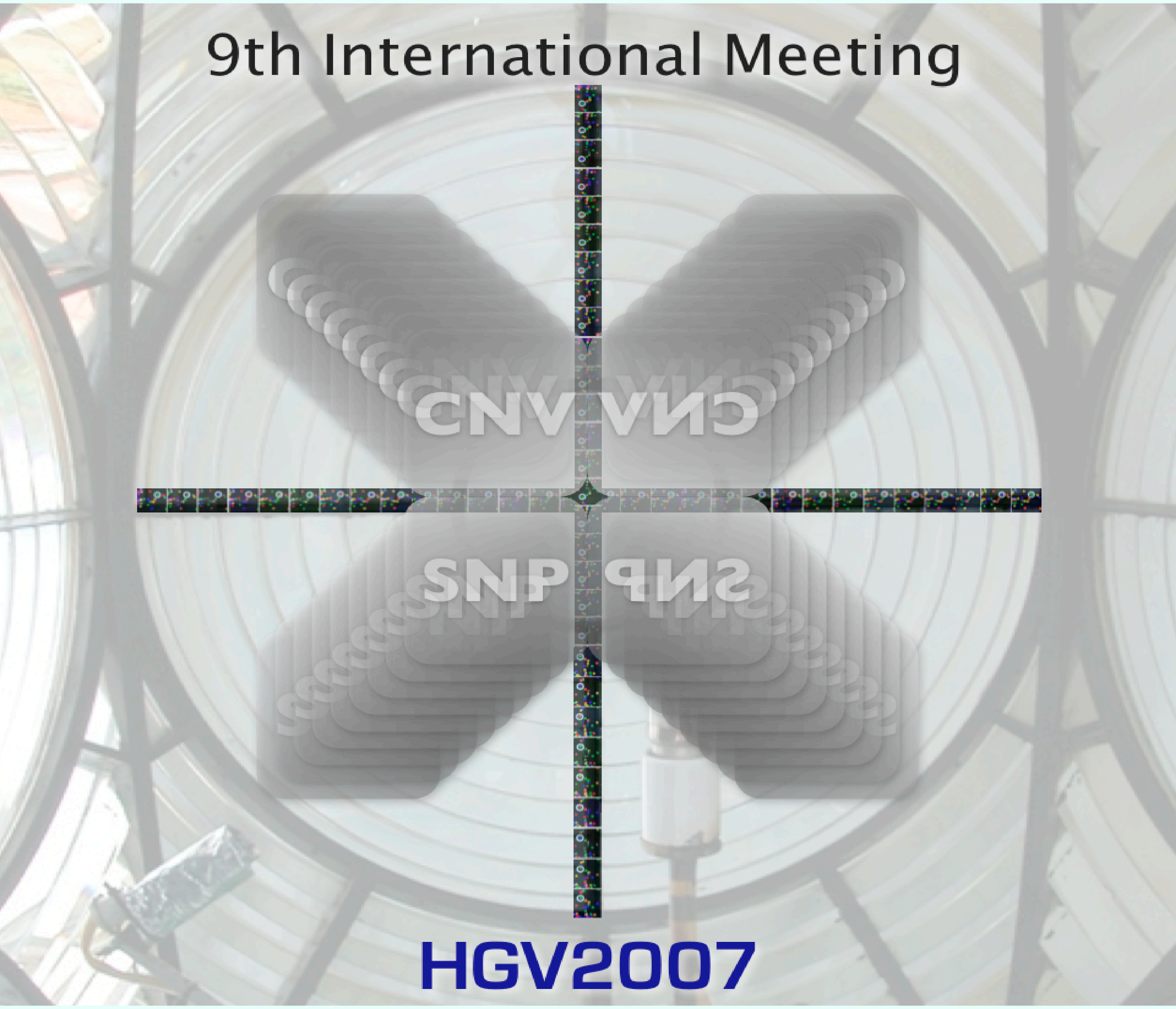

Hotel Dolce Sitges
Catalunya, Spain

September
6-8, 2007

9TH INTERNATIONAL MEETING ON

HUMAN GENOME VARIATION AND COMPLEX GENOME ANALYSIS

SEPTEMBER 6TH – 8TH, 2007

Sitges (Barcelona), Catalonia, Spain

Organizers

Anthony J Brookes

Stephen J Chanock

Nancy Cox

Xavier Estivill

Pui-Yan Kwok

Steve Scherer

Organisational Support

Aurea Rodríguez

Magalí Bertomeus

Bernice Parker

http://hgv2007.nci.nih.gov

Academic

Generalitat de Catalunya (Catalan Government)

Center for Genomic Regulation

Centro Nacional de Genotipado (CeGen)

Genoma España

McLaughlin Centre for Molecular Medicine

National Cancer Institute

National Human Genome Research Institute

Spanish Ministry of Science and Education

University of Leicester

Corporate

Affymetrix

Agilent Technologies

Applied Biosystems

Beckman Coulter, Inc

Biocomputing Platforms Ltd

deCODE Genetics

Genizon BioSciences

GlaxoSmithKline

Illumina, Inc

Kreatech Biotechnology

Nature Genetics

Nature Methods

Nature Reviews Genetics

NimbleGen Systems, Inc.

Roche Applied Science

Sequenom

ACADEMIC SPONSOR LOGOS

University of Leicester

Genoma España

National Human Genome Research Institute

CRG
Centre de Regulació Genòmica

NATIONAL CANCER INSTITUTE

AÑO de la CIENCIA 2007

McLaughlin Centre for Molecular Medicine

Agència de Gestió d'Ajuts Universitaris i de Recerca

CEGEN
Centro Nacional de Genotipado

MINISTERIO DE EDUCACIÓN Y CIENCIA

CORPORATE SPONSOR LOGOS

SEQUENOM®

Roche

NimbleGen® SYSTEMS, INC.

nature REVIEWS GENETICS

nature methods
Techniques for life scientists and chemists

nature genetics

KREATECH BIOTECHNOLOGY

gsk GlaxoSmithKline

illumina®

GENIZON BioSciences

deCODE genetics

BECKMAN COULTER.

BC Biocomputing Platforms Ltd

AB Applied Biosystems

Agilent Technologies

AFFYMETRIX®

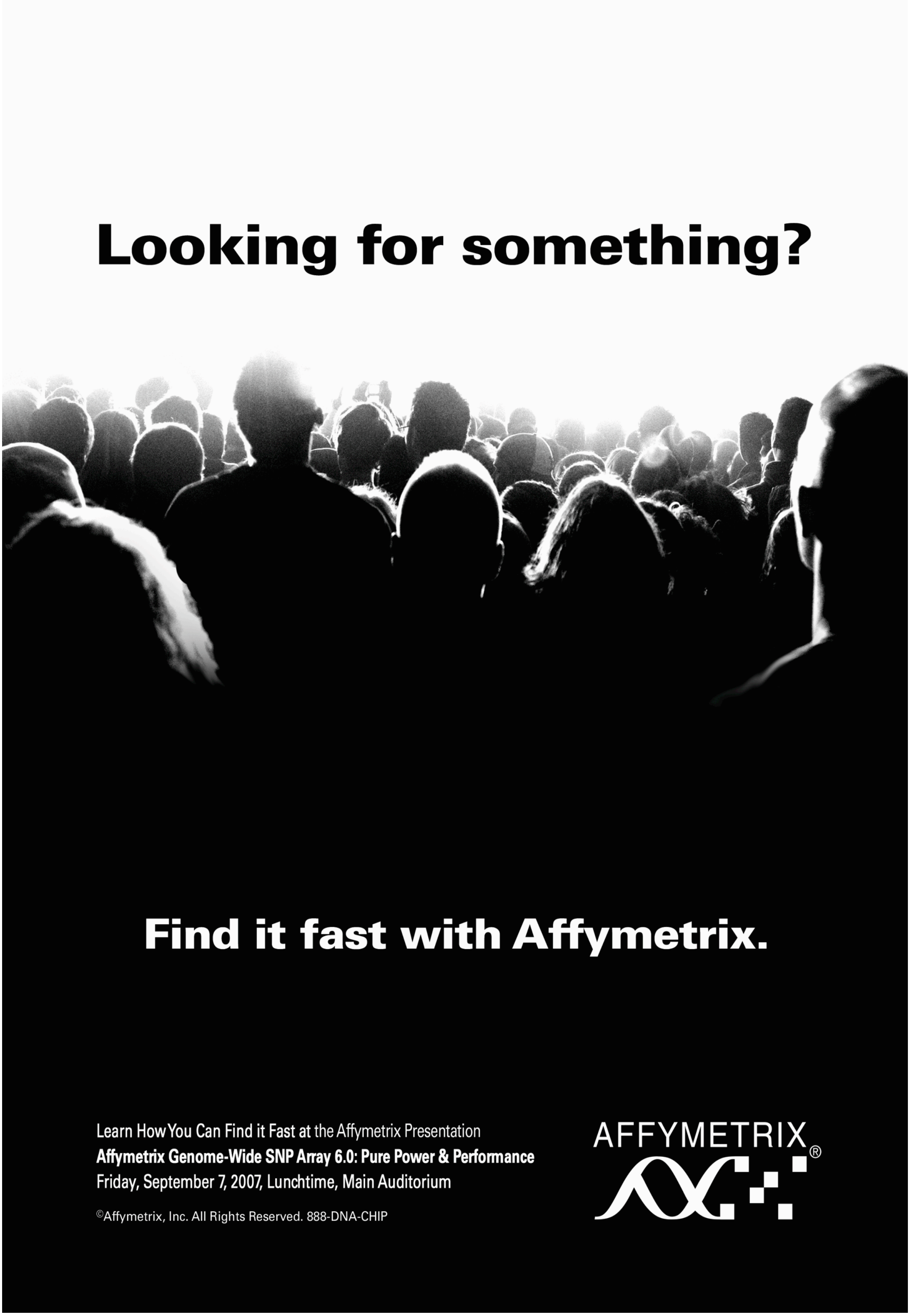
Looking for something?
Find it fast with Affymetrix.
Learn How You Can Find it Fast at the Affymetrix Presentation
Affymetrix Genome-Wide SNP Array 6.0: Pure Power & Performance
Friday, September 7, 2007, Lunchtime, Main Auditorium
©Affymetrix, Inc. All Rights Reserved. 888-DNA-CHIP
AFFYMETRIX®

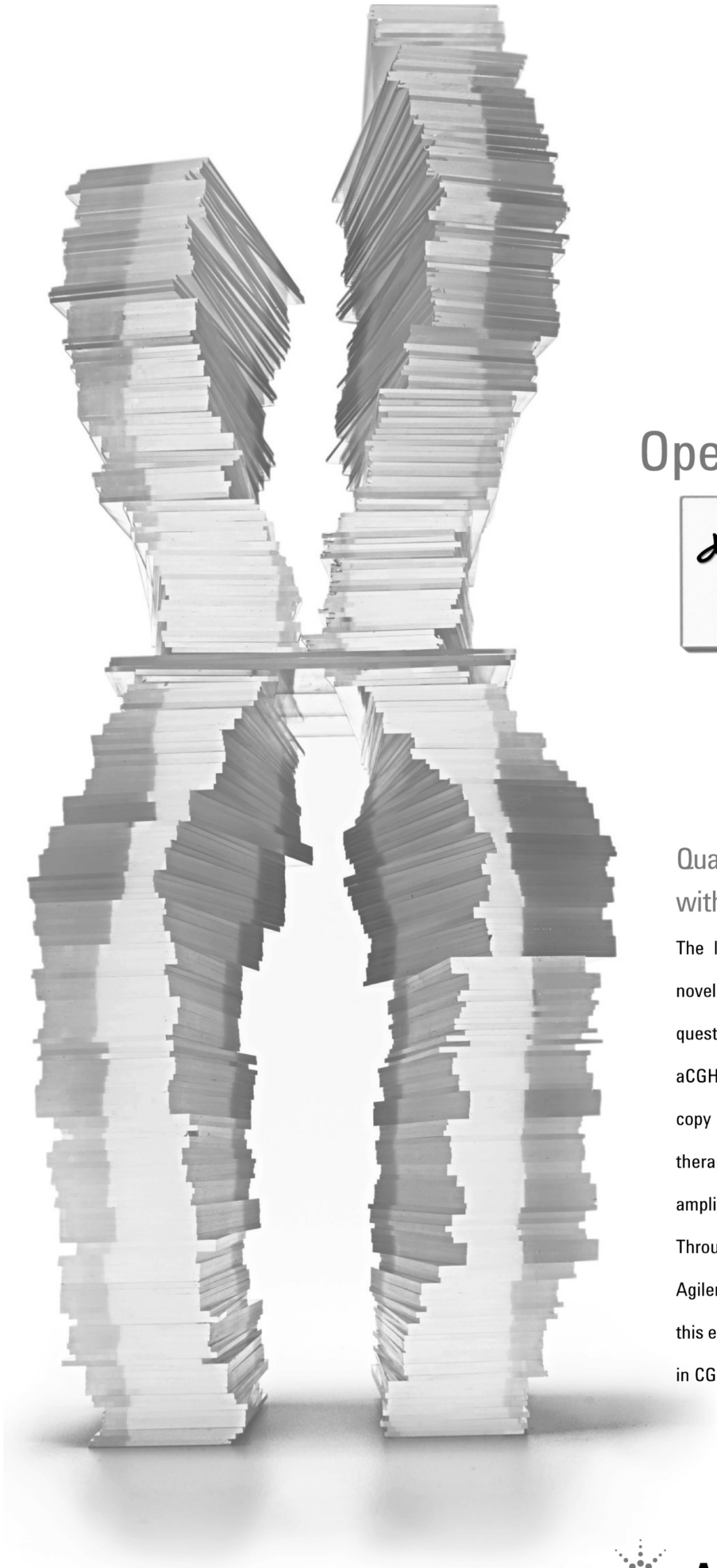

Open to:

detection of single copy changes

Agilent

Quantify chromosomal gains and losses with Agilent oligo aCGH microarrays

The landscape of genomic research is changing, with novel applications providing valuable insights into familiar questions. Researchers are now using Agilent's oligo aCGH arrays to detect genome-wide submicroscopic copy number changes.[1] Cancer researchers are finding therapeutic and diagnostic opportunities in recurrent amplifications and deletions in disease-linked genomes.[2] Through discriminating resolution and superior sensitivity, Agilent provides the tools that keep you at the forefront of this evolving field. To learn more about the latest advances in CGH, visit **OpenGenomics.com/CGH.**

1. Toruner et al. 2007. *Am J Med Genet A.* 143A(8): 824-829.
2. Tonon et al. 2005. *Proc Natl Acad Sci USA.*102(27): 9625-9630.

© Agilent Technologies, Inc. 2007

Agilent Technologies

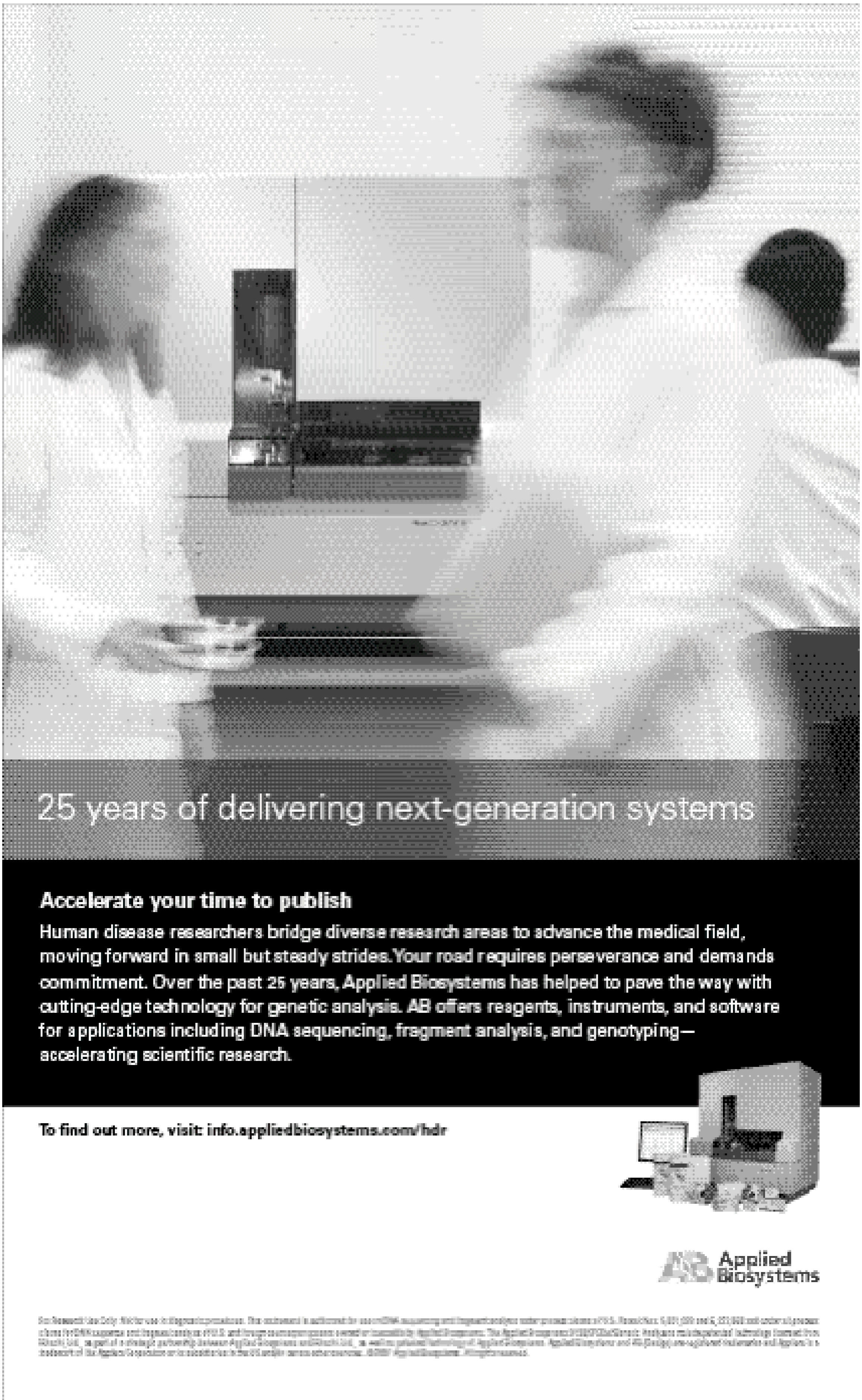
25 years of delivering next-generation systems
Accelerate your time to publish
Human disease researchers bridge diverse research areas to advance the medical field, moving forward in small but steady strides. Your road requires perseverance and demands commitment. Over the past 25 years, Applied Biosystems has helped to pave the way with cutting-edge technology for genetic analysis. AB offers reagents, instruments, and software for applications including DNA sequencing, fragment analysis, and genotyping—accelerating scientific research.
To find out more, visit: info.appliedbiosystems.com/hdr
Applied Biosystems

BC|SNPmax

Solving the Problems of Data Management in Whole Genome Association Studies

- A scalable database for a large amount of SNP data
- Automated entry of data from modern high-throughput SNP genotyping instruments
- More efficient data analysis for quicker publishing
- A cost-effective solution which can be quickly implemented

BC/SNPmax is a data management solution which integrates high-density SNP genotype data with phenotypes and maps, and creates a highly productive data analysis pipeline with automated input file generation for analysis programs.

BC/SNPmax has been designed to deal with the needs of modern whole genome association studies and to provide safe, structured, and well-documented storage of valuable research data. The implementation of this highly sophisticated system is both fast and cost-effective compared to customized internal programming projects.

BC Biocomputing Platforms Ltd

Contact
Biocomputing Platforms Ltd
Innopoli 2, Tekniikantie 14
FI-02150 Espoo, Finland
Tel. +358 9 2517 7340
Fax +358 10 296 1288
Email info@bcplatforms.com
www.bcplatforms.com

Are you missing a piece of the puzzle?

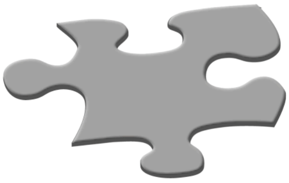

deCODE now offers the first comprehensive solution targeting the 'unSNPable' genome:

Copy Number Variation (CNV) analysis

- Targets the most variable part of the genome, using over 55,000 SNPs and probes not included in any other high-density genotyping platform
- Covers all known CNV regions and over 13,000 novel regions likely to contain CNV
- Comprehensive coverage of the complex MHC region, megasatellites and segmental duplications
- Added value with Disease Miner™ Professional, deCODE's proprietary software for fast and easy data analysis and management
- Available as three new BeadChip arrays, specifically designed to probe regions of CNV:
  - Custom 60K CNV array
  - HumanCNV370K array
  - Human1M array

For more information on deCODE's CNV analysis, Disease Miner™ Professional or other genomic services provided by deCODE, please contact us today at genotyping@decode.com

deCODE genomic services

Scientific Excellence – Complete Solutions

N-America: 1-866-713-DCGN Europe/ROW: +354-570-1900
E-mail: genotyping@decode.com Web: www.decode.com/genotyping

GENIZON BIOSCIENCES The leader in genetic solutions

Fast track your research program with 4 billion genotypes per month

Rapid, top-quality SNP genotyping and genetic analysis at Genizon

Genizon offers GLP-compliant genetic services - high throughput Illumina technology-based SNP genotyping and our proprietary genetic analysis computing pipeline - to provide industry leading statistical power for your study design. Off-the-shelf chips for large whole-genome studies or custom chips for targeted studies can be used with tens to thousands of samples.

Genizon has extensive experience from the completion of ten successful whole-genome association studies. These studies resulted in the rapid identification of multiple disease-associated genes, biomarkers and gene-based drug targets.

High Quality Genotyping

Call Rate >99%
Accuracy >99.9%
Reproducibility >99.9%
Data based on >8,300 samples and 3.6 billion genotypes

Quick start and high throughput

Genotyping for a study of 4,000 individuals using a 550k chip can be completed within 6 weeks.

Genetic analysis

- Fully automated analysis pipeline
  - Quality control
  - Phase determination
  - Haplotype and for single marker association analysis
- Customized analyses also available

Example from a Genizon whole-genome association study - the signal is at least 2 orders of magnitude greater with the Genizon process than with lower quality genotyping.

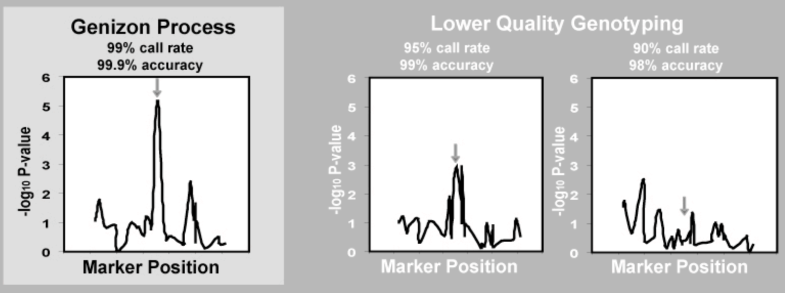

7-marker haplotypes represented, arrow indicates same chromosomal location

GENIZON BioSciences

Visit us at the exhibition hall!

1.514.865.4347 or 1.888.244.3888
services@genizon.com
www.genizon.com

you&i

SNP genotyping.
Gene expression.
And now Solexa® sequencing.

Let's find the answers together.

It's your research. You question. You test.
You want answers—quickly, accurately, and at a good value.

Illumina has the tools that address the core applications in modern genetic analysis—SNP genotyping, gene expression, and now sequencing. Working alongside you, Illumina strives to make it as easy as possible to find the answers that move your research forward. By continually replicating the cycle of listening, anticipating, and innovating, we are pioneering new applications like CNV analysis and DNA methylation.

Together we are the Illumina Community. We collaborate to deliver easy-to-use tools with the highest quality and best value that enable all of us to help understand, cure, and ultimately prevent disease.

Is it time for you to join the Illumina Community?
Let's find the answers together.

Find the answers at:
www.illumina.com

illumina®

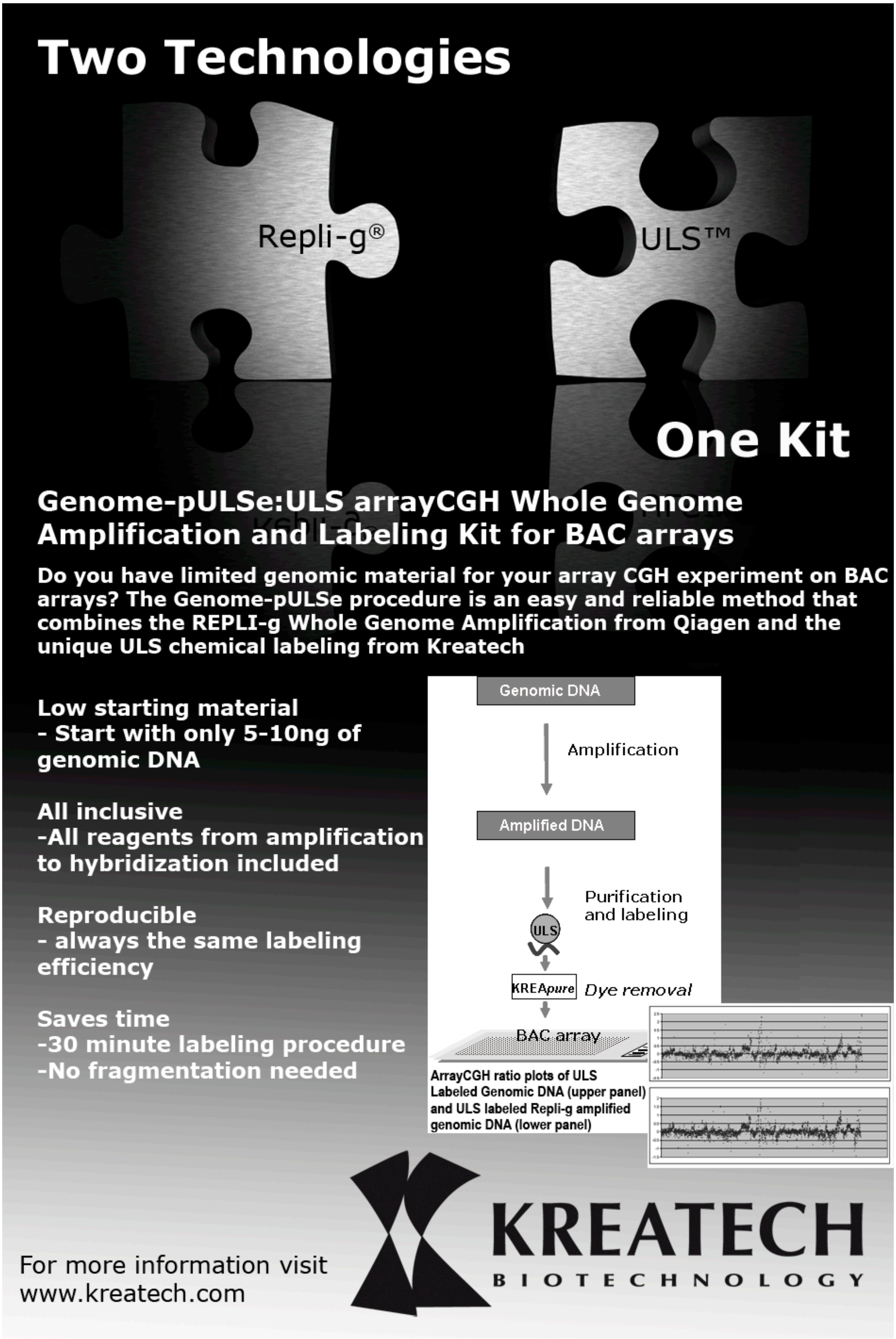
Two Technologies
Repli-g®
ULS™
One Kit
Genome-pULSe:ULS arrayCGH Whole Genome Amplification and Labeling Kit for BAC arrays
Do you have limited genomic material for your array CGH experiment on BAC arrays? The Genome-pULSe procedure is an easy and reliable method that combines the REPLI-g Whole Genome Amplification from Qiagen and the unique ULS chemical labeling from Kreatech
Low starting material
- Start with only 5-10ng of genomic DNA
All inclusive
-All reagents from amplification to hybridization included
Reproducible
- always the same labeling efficiency
Saves time
-30 minute labeling procedure
-No fragmentation needed
Genomic DNA
Amplification
Amplified DNA
Purification and labeling
ULS
KREApure
Dye removal
BAC array
ArrayCGH ratio plots of ULS Labeled Genomic DNA (upper panel) and ULS labeled Repli-g amplified genomic DNA (lower panel)
KREATECH
BIOTECHNOLOGY
For more information visit www.kreatech.com

nature REVIEWS GENETICS

CUTTING EDGE

With cutting edge Reviews written by leaders in the field, *Nature Reviews Genetics* puts key discoveries into context.

Ensure you have access to *Nature Reviews Genetics* and stay on top of your field.

www.nature.com/reviews/genetics

Nature Reviews Genetics – the no. 1 monthly review journal in genetics and heredity*.

*2006 Impact Factor 22.947: Journal Citation Reports, Thomson 2007

DEPTH BREADTH **CUTTING EDGE** QUALITY AUTHORITATIVE ACCESSIBLE

nature publishing group npg

Nature Publishing Group's genetics portfolio:

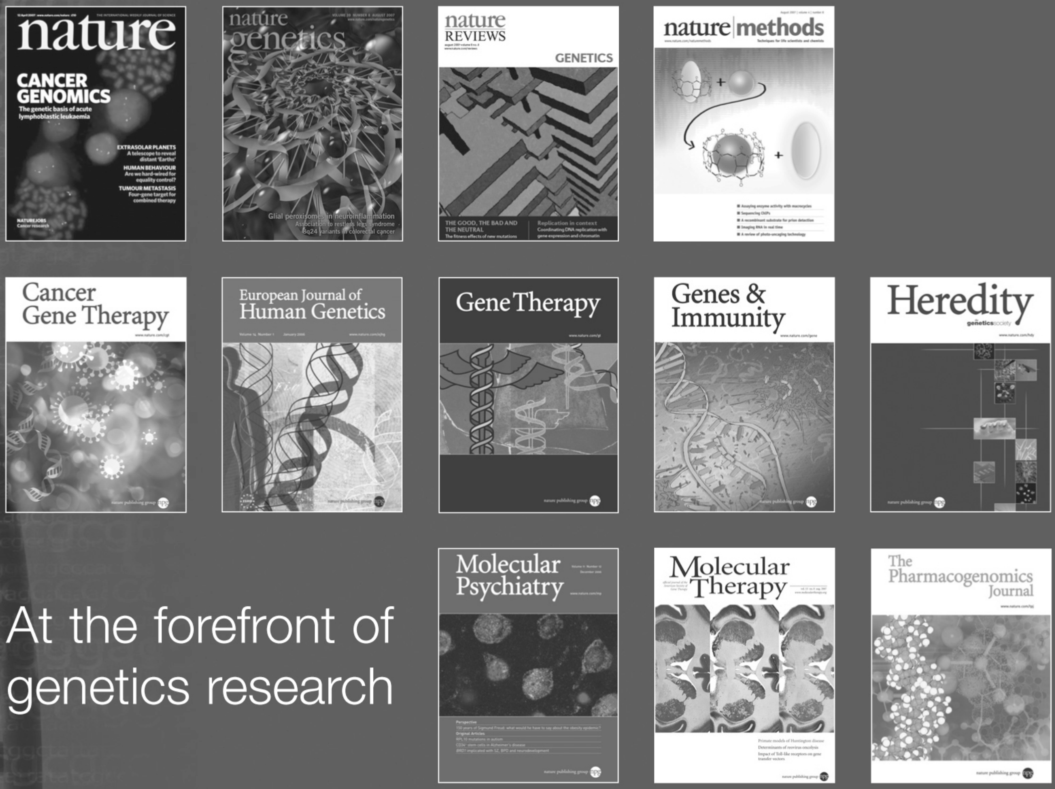

At the forefront of genetics research

Nature Publishing Group (NPG) journals and online gateways enable you to stay ahead in the fast-moving area of genetics.

View NPG's genetics subject area at **www.nature.com/genetics** for collections of leading research articles, special web focuses and readable, insightful comment on the latest research in genetics.

Other journals containing genetics content that also may be of interest include:

Nature Structural & Molecular Biology	**www.nature.com/natstructmolbiol**
Nature Cell Biology	**www.nature.com/naturecellbiology**
The EMBO Journal	**www.embojournal.org**
EMBO reports	**www.emboreports.org**
Cell Research	**www.nature.com/cr**
Molecular Systems Biology	**www.molecularsystemsbiology.com**

In addition the following resources are available free online:

The Signaling Gateway	**www.signaling-gateway.org**
The Omics Gateway	**www.nature.com/omics**

Visit www.nature.com and click subscribe for subscription information.

www.nature.com/genetics

nature publishing group npg

emergence convergence

Fondation IPSEN, *Nature Methods* and *Nature Genetics* present:

GENOME EVOLUTION AND STRUCTURAL VARIATION: Next steps in natural human genetic variation

This one day idea-generating meeting will draw on Seattle's research community, and invited speakers from the fields of human genetic variation and evolution. Discussion will focus on the overall question of the next steps in characterizing human genetic variation and genome evolution. Speakers will focus on challenges related to their topic, such as incorporating structural variation detection into current technologies, or within current large-scale projects to characterize natural genetic variation.

November 28, 2007
The Sheraton Seattle Hotel
Seattle, WA, USA

ORGANIZERS
Orli Bahcall (*Nature Genetics*, USA)
Veronique Kiermer (*Nature Methods*, USA)
Evan Eichler (University of Washington, USA)
Deborah Nickerson (University of Washington, USA)
Yves Christen (Fondation IPSEN, France)

SPEAKERS
Nigel P. Carter (The Wellcome Trust Sanger Institute, UK)
Andrew G. Clark (Cornell University, USA)
Evan E. Eichler (University of Washington, USA)
Kelly A. Frazer (The Scripps Research Institute, USA)
Matthew E. Hurles (The Wellcome Trust Sanger Institute, UK)
Elaine Mardis (Washington University School of Medicine, USA)
Deborah Nickerson (University of Washington, USA)
Jonathan Pritchard (University of Chicago, USA)
Stephen Scherer (The Hospital for Sick Children, University of Toronto, Canada)

Attendance at this meeting is Free on application.

For more information go to
www.nature.com/natureconferences/eandc/genetics

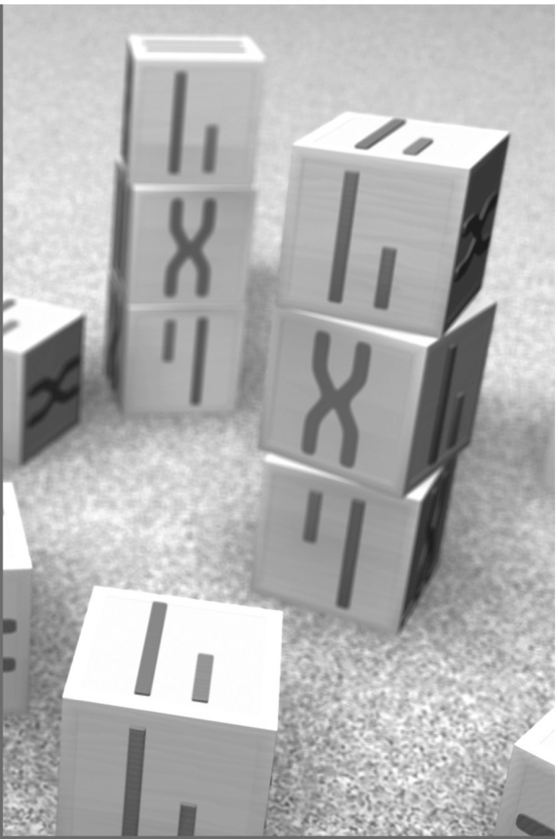

Image: Created by Paul H. Dear, MRC Laboratory of Molecular Biology

a nature conference

FONDATION IPSEN | nature methods | nature genetics

We are High-Definition Genomics℠

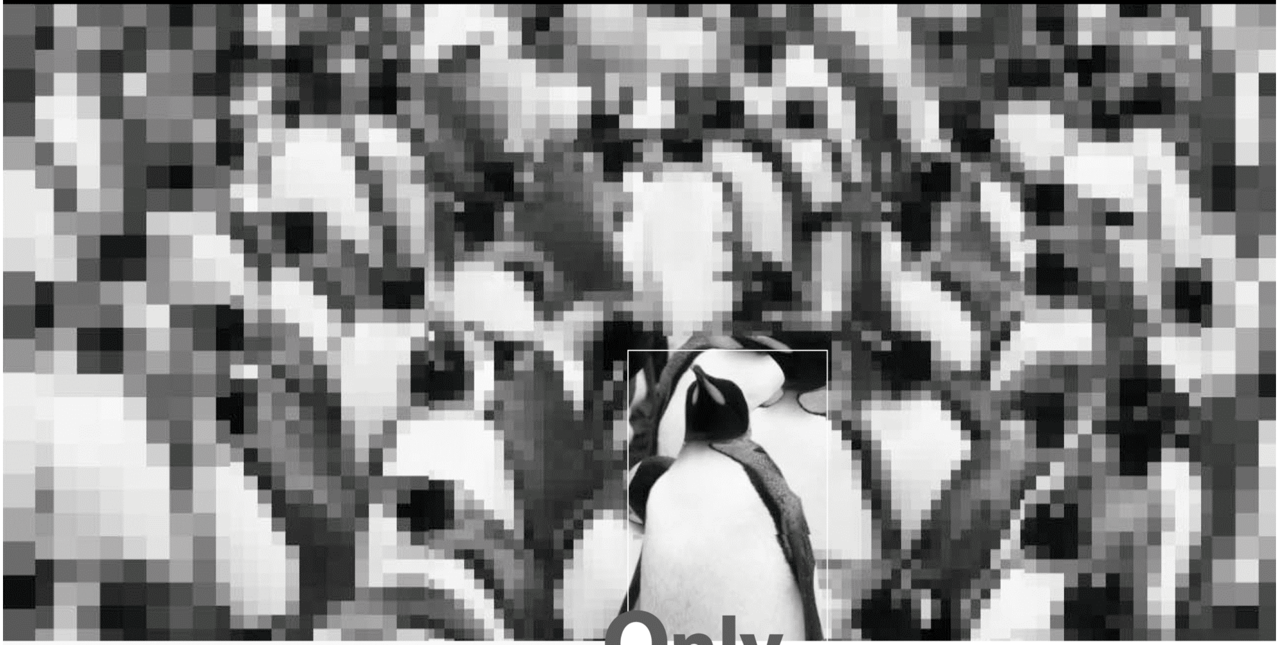

Only

NimbleGen

offers both ultra-high density and long oligo probes, allowing you to obtain both high volume and quality data in each experiment ■ Until you've compared our applications with your current approaches, you won't realize just how much you're missing.

High-Definition Genomics means a higher standard in genomic and epigenomic research, both with respect to individual analyses as well as from integration of data on many forms of genome variation. We work with scientists around the world to develop and deploy new tools for acquiring high-quality, high-resolution, genome-wide data. Our customers obtain a clearer view of genome variation and how it relates to biology and medicine.

NimbleGen cutting-edge applications:
- **ChIP-chip**—genome-wide transcription factor mapping
- **Array CGH**—DNA copy number with sub-Kb resolution
- **Gene Expression**—cost-effective multiplex options
- **DNA Methylation**—entire genomes at high resolution

Long oligonucleotide probes
Ensure highest sensitivity and better signal-to-noise ratios compared with short probes.

Ultra-high density arrays
Capture more information than other platforms with more than 2 million data points per experiment.

Whole genome or targeted region analysis

NimbleGen® SYSTEMS, INC.

Start your microarray project with NimbleGen today, and experience *High-Definition Genomics*.

© Copyright 2007 NimbleGen Systems, Inc. All Rights Reserved.

www.NimbleGen.com ■ ngsales@NimbleGen.com ■ (877) NimbleGen

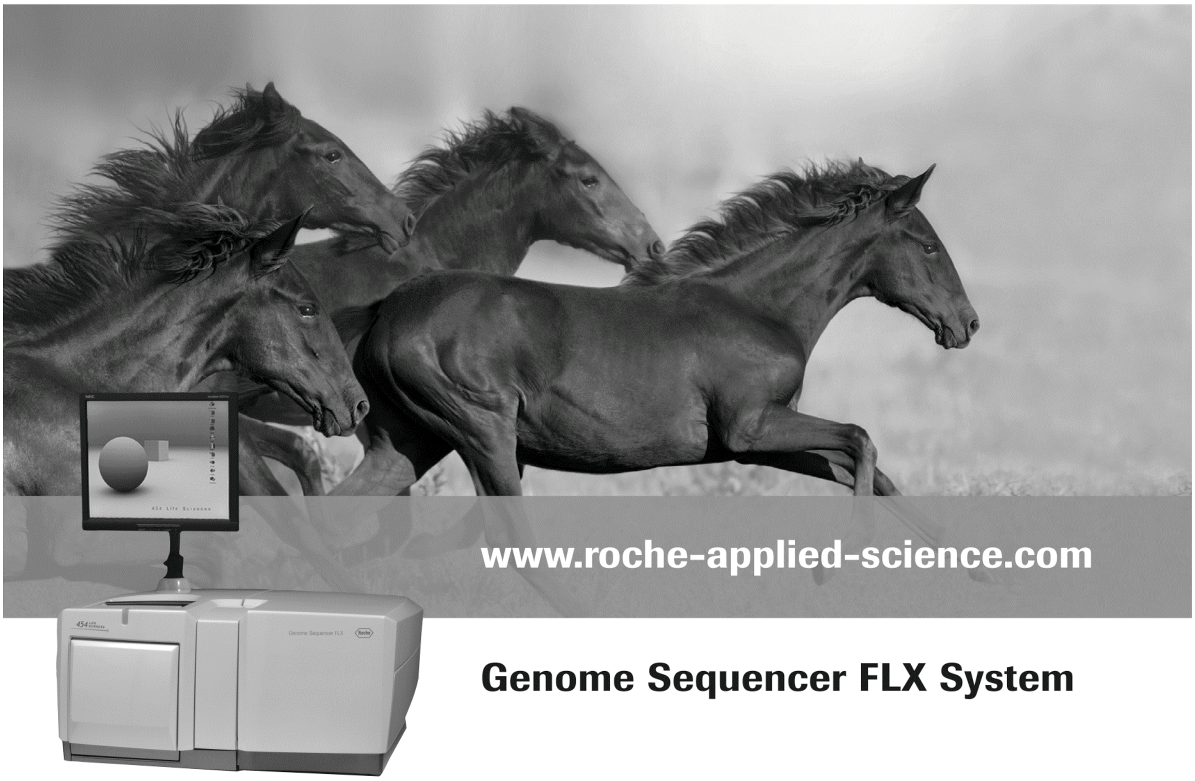

Genome Sequencer FLX System

Longer sequencing reads mean more applications.

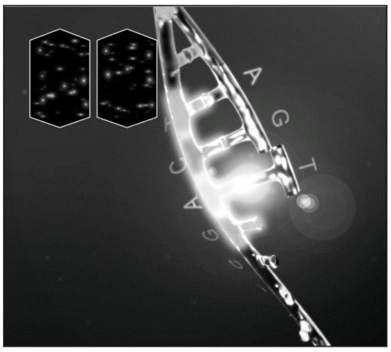

Sequencing-by-Synthesis: Using an enzymatically coupled reaction, light is generated when individual nucleotides are incorporated. Hundreds of thousands of individual DNA fragments are sequenced in parallel.

In 2005, the Genome Sequencer 20 System was launched
Read length: 100 bases
20 million bases in less than 5 hours

In 2007, the Genome Sequencer FLX System was launched
Read length: 250 to 300 bases
100 million bases in less than 8 hours

Available in 2008, the Genome Sequencer FLX with improved chemistries
Read length: >400 bases
1 billion bases in less than 24 hours

More applications lead to more publications

Proven performance with an expanding list of applications and more than 80 peer-reviewed publications.
Visit ***www.genome-sequencing.com*** to learn more.

454 LIFE SCIENCES

Roche Diagnostics GmbH
Roche Applied Science
68298 Mannheim, Germany

Roche

For life science research only.
Not for use in diagnostic procedures.

454 and GENOME SEQUENCER are trademarks of
454 Life Sciences Corporation, Branford, CT, USA.
© 2007 Roche Diagnostics GmbH. All rights reserved.

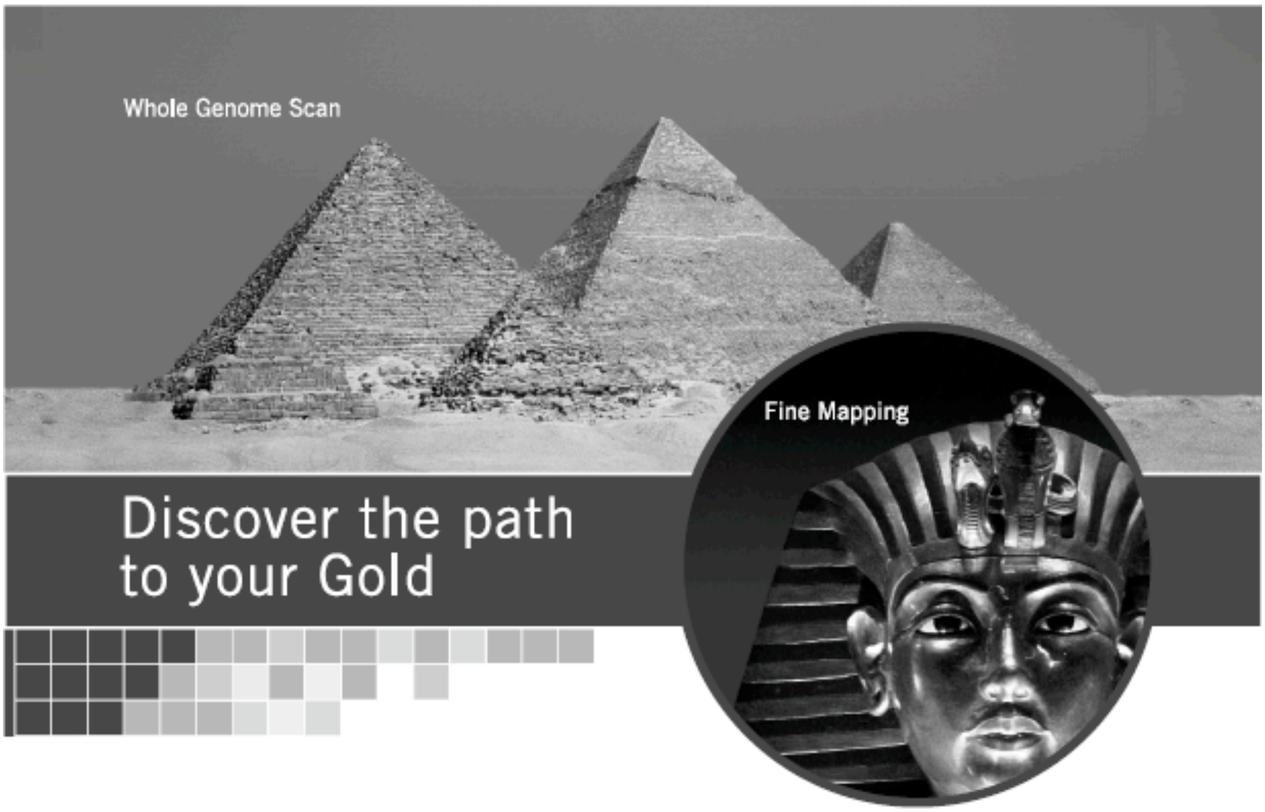

MassARRAY® *iPLEX*™ Gold for SNP Genotyping.
The Finest in Fine Mapping.

- Ideally suited to follow up your whole genome scan
- Highest available data quality for your research
- 3.5¢ per genotype in a 36-plex reaction
- Broad throughput ranges from a few hundred up to 150,000 genotypes per day

Do you know the name of the Pharaoh whose intact tomb was discovered in 1923? Find the answer at www.sequenom.com/pharaoh

SEQUENOM®
www.sequenom.com

CEGEN

Centro Nacional de Genotipado

SNP genotyping services by using high-throughput technologies (Illumina Beadarray, Affymetrix GeneChip, Applied Biosystems SNPlex and Sequenom MassARRAY)

CeGen (Spanish National Genotyping Centre) is an initiative of ***Genoma España*** aimed to offer a technological platform to deliver genotyping services in all species to the Spanish scientific community. We have provided **SNP** genotyping services since 2005, after a period of validation and testing. Up to August 2007, more than 230 genotyping projects are achieved or in progress within **CeGen**.

CeGen is organized following a nodal structure: there is a central structure in *Universitat Pompeu Fabra* that deals with coordination and three fully-equipped nodes that develop SNP genotyping projects: the first node is at *Centre de Regulació Genòmica* (SNPlex and Illumina technologies), the second node is at *Universidad de Santiago* (Sequenom and Affymetrix equipment) and the third node is at *Centro Nacional de Investigaciones Oncológicas* (Illumina and Taqman technologies). Moreover, complementary services such as DNA extraction, quantification and amplification are also offered. **CeGen** is also collaborating with **INB** (Spanish National Institute of Bioinformatics) for bioinformatics support at pre and post genotyping stages.

CeGen can offer a high-throughput SNP genotyping capacity at competitive prices, since they are partially supported by ***Genoma España***. High-throughput capacity, competitive costs and scalability in SNP genotyping are the main advantages of the **CeGen** initiative.

For more information, visit our web site at **www.cegen.org**
Or contact us at **coordinacion.cegen@upf.edu**

9TH INTERNATIONAL MEETING ON

HUMAN GENOME VARIATION AND COMPLEX GENOME ANALYSIS

CONTENTS

	Page
Organizers and Organisational Support ..	i
Meeting Sponsors	iv-v
Commercial Advertisements	vi-xx
Contents	xxiii
Meeting Summary	xxv
Meeting Agenda	xxvi
Abstracts for Oral Presentations	1-38
Abstracts for Company Presentations	xxxv
Abstracts for Poster Presentations	39-187
List of Presenting Participants	xlv
Participant Contact Details	li
Notes	lxv
Useful Meeting Information	lxxiii

MEETING SUMMARY

THURSDAY, 6TH SEPTEMBER

13.00-14.30	REGISTRATION
14.30-14.40	Welcome
14.40-16.45	SESSION 1 – STRUCTURAL VARIATION AND PHENOTYPE
16.45-17.30	COFFEE BREAK
17.30-19.05	SESSION 2 – VARIABILITY AND INFORMATICS
20.00-21.30	DINNER (Hotel Dolce Sitges)

FRIDAY, 7TH SEPTEMBER

08.30-10.30	SESSION 3 – STRUCTURAL VARIATION FUNDAMENTALS
10.30-11.10	COFFEE BREAK
11.10-12.30	SESSION 4 – FUNCTIONAL VARIABILITY
12.30-14.30	LUNCH
	LUNCH TIME COMPANY PRESENTATIONS
13.00-13.30	Affymetrix
13.30-14.00	Agilent
14.30-15.40	POSTER SESSION WITH COFFEE
15.40-17.50	SESSION 5 – VARIATION DATABASES
18.30-21.30	EXCURSION AND DINNER

SATURDAY, 8TH SEPTEMBER

08.30-10.20	SESSION 6 – VARIABILITY AND THE POPULATION
10.20-11.00	COFFEE BREAK
11.00-12.30	SESSION 7 – SEQUENCING INDIVIDUAL GENOMES
12.30-14.30	LUNCH
	LUNCH TIME COMPANY PRESENTATIONS
13.00-13.30	Roche Applied Science
13.30-14.00	Applied Biosystems
14.30-15.40	POSTER SESSION WITH COFFEE
15.40-17.20	SESSION 8 – VARIABILITY AND PHENOTYPE
17.20-17.30	Closing Remarks and Discussion
20.00-21.30	DINNER

MEETING AGENDA

THURSDAY, 6TH SEPTEMBER

13.00-14.30 **REGISTRATION**

14.30-14.40 **Welcome**

SESSION 1 "STRUCTURAL VARIATION AND PHENOTYPE"
Moderator – Stephen Scherer

14.40-15.05 **Barbara Trask**
Recent Changes in Mammalian Chemosensory Receptor Repertoires

15.05-15.30 **John Armour**
Developing Paralogue Ratio Tests (PRT) for High-Throughput CNV Typing in Association Studies

15.30-15.55 **Joris Veltman**
Copy Number Variation in Mental Retardation

15.55-16.20 **Xavier Estivill**
Copy Number Variants and Common Disorders: Filling the Gaps and Exploring Complexity

16.20-16.45 **Deborah Nickerson**
Lessons from SNP Discovery in Candidate Genes

16.45-17.30 **COFFEE BREAK**

SESSION 2 "VARIABILITY AND INFORMATICS"
Moderator – Barbara Trask

17.30-17.55 **Nancy Cox**
Direct and Indirect Assessment of Copy Number Variation

17.55-18.10 **Lluis Armengol**
PIWI-Interacting RNAs Are Enriched in Segmental Duplications and Copy Number Variable Regions in The Human Genome

18.10-18.35 **Heikki Lehvaslaiho**
Exchange Standard for Genomic Polymorphism and Genotyping Data

18.35-18.50 **Yum Lina Yip**
Archiving Single Amino Acid Polymorphisms In The UniProtKB/Swiss-Prot Knowledgebase

18.50-19.05 **Andrew Devereau**
A Variation Database for UK Diagnostic Molecular Laboratories–DmuDB

20.00-21.30 **DINNER (Hotel Dolce Sitges)**

FRIDAY, 7TH SEPTEMBER

08.30-08.35 **Announcements**

SESSION 3 "STRUCTURAL VARIATION FUNDAMENTALS"
Moderator – Nancy Cox

08.35-09.00 **Stephen Scherer**
Discovery of Structural Variation in Control and Disease Studies

09.00-09.25 **Don Conrad**
Genetics of Copy Number Variation in a Founder Population

09.25-09.50 **Iuliana Ionita**
On Analysis of Copy-Number Variations in Genome-Wide Association Studies: a Translation of the Family-Based Association Test

09.50-10.15 **Matthew Hurles**
Towards a Comprehensive Map of Copy Number Variation

10.15-10.30 **Jeff Gulcher**
New Platform for Discovery and Genotyping of Copy Number Variants Reveals Much Higher Number of Common CNVs in Human Genome

10.30-11.10 **COFFEE BREAK**

SESSION 4 "FUNCTIONAL VARIABILITY"
Moderator – Xavier Estivill

11.10-11.35 **Vivian Cheung**
Genetics of Natural Variation in Human Gene Expression

11.35-12.00 **Emmanouil Dermitzakis**
Causes of Regulatory Variation in the Human Genome

12.00-12.15 **Shamil Sunyaev**
Widely Distributed Non-Coding Selection in the Human Genome

12.15-12.30 **Allen Roses**
Haplotypes Within the APOE LD Region May Have Pharmacogenetic Effects Predicting Efficacy of Alzheimer's Patients Receiving Rosiglitazone

12.30-14.30 **LUNCH**

LUNCH TIME COMPANY PRESENTATIONS

13.00-13.30 **Affymetrix**
Marcus Hausch: Affymetrix Genome-Wide SNP 6.0, Pure Power and Performance

13.30-14.00 **Agilent**
Stephen Laderman: High Resolution Discovery and Characterization of Copy Number Variation in Apparently Healthy Individuals Using Custom Oligonucleotide DNA Microarrays.
Didier Goidin: A Brief Overview of Additional Array Applications Beyond CGH and CNV (Expression, miRNA, Methylation and ChIP)

14.30-15.40 **POSTER SESSION WITH COFFEE [odd numbers to present]**

SESSION 5 "VARIATION DATABASES"
Moderator – Pui-Yan Kwok

15.40-16.05 **Anthony Brookes**
International Progress in Databasing Genotype-To-Phenotype Relationships

16.05-16.30 **James Ostell**
Human Variation Resources at NCBI

16.30-16.55 **Ewan Birney**
Ensembl Variation and Functional Data

16.55-17.20 **Lincoln Stein**
The HapMap Web Site

17.20-17.35 **Carole Charlier**
Patrocles: a Database of Polymorphic miRNA-Mediated Gene Regulation

17.35-17.50 **Lars Feuk**
The Database of Genomic Variants Annotating Structural Variation in the Human Genome

18.30-21.30 **EXCURSION AND DINNER (Vallformosa Winnery, with tickets)**

SATURDAY, 8TH SEPTEMBER

08.30-08.35 **Announcements**

SESSION 6 "VARIABILITY AND THE POPULATION"
Moderator – Anthony Brookes

08.35-09.00 **Chris Ponting**
Evolutionary Dynamics of Recurrent Copy Number Variation in the Human Genome

09.00-09.15 **George Perry**
Diet and the Evolution of Human Amylase Gene Copy Number Variation

09.15-09.40 **Gilles Thomas**
Impact of Population Structure in Genome Wide Association Studies

09.40-09.55 **Jaume Bertranpetit**
Recombination Variation Among Human Populations, with an Insight on Recombination Evolution

09.55-10.20 **Esteban González Burchard**
The Importance of Race/Ethnicity and Genetics in Biomedical Research and Clinical Practice; Lessons from the Genetics of Asthma in Latino Americans (GALA) Study

10.20-11.00 **COFFEE BREAK**

SESSION 7 "SEQUENCING INDIVIDUAL GENOMES"
Moderator – Stephen Chanock

11.00-11.25 **George Church**
Personal Genome Project: Open-Source Software, Hardware, Wetware and Elsware

11.25-11.50 **Anne Cambon-Thomsen**
Ethics and the Challenge of Biobanks Exchange Policies

11.50-12.15 **Ivo Gut**
High-Throughput Resequencing by Mass Spectrometry

12.15-12.30 **Sanjeev Bhaskar**
High Quality, High-Throughput Quality Targeted Re-Sequencing

12.30-14.30 **LUNCH**

LUNCH TIME COMPANY PRESENTATIONS

13.00-13.30 **Roche Applied Science**
Jan Korbel: Long Read Length as Basis for Superior Re-Sequencing and De Novo Sequencing Performance

13.30-14.00 **Applied Biosystems**
Francisco de la Vega: SOLiD™-ifying the Findings of Genome-Wide Association Studies by Next-Generation, High-Throughput Sequencing

14.30-15.40 **POSTER SESSION WITH COFFEE [even numbers to present]**

SESSION 8 "VARIABILITY AND PHENOTYPE"
Moderator – George Church

15.40-16.05 **Stephen Chanock**
Genome Wide Association Studies in Breast and Prostate Cancer

16.05-16.30 **Angel Carracedo**
Pharmacogenetics: From Research to Clinical Use

16.30-16.55 **Pui-Yan Kwok**
Genome-Wide SNP Genotyping in Association Studies of Complex Traits

16.55-17.20 **Samuel Levy**
Toward the Comprehensive Identification of Complex DNA Variants in a Single Human

17.20-17.30 **Closing Remarks and Discussion**

20.00-21.30 **DINNER (Hotel Dolce Sitges)**

Abstracts for Oral Presentations

Armengol, Lluis

PIWI-Interacting RNAs Are Enriched In Segmental Duplications And Copy Number Variable Regions In The Human Genome

ARMENGOL, LLUIS; Brunet, Anna; Caceres, Mario; Estivill, Xavier
Genes & Disease Program, Center for Genomic Regulation (CRG)

Small noncoding RNAs (ncRNAs) act as guides to direct mRNA degradation, translational repression, heterochromatin structure and DNA removal. PIWI-interacting RNAs (piRNAs) are a newly recognized class of small (26~34 nt) noncoding RNAs identified in mammalian germline cells. They associate with PIWI members of the Argonaute protein family. In Drosophila, Piwi (P-element-induced wimpy testis) encodes a protein, which is essential for germ stem cell self-renewal and piRNAs might act by guiding the PIWI complex to their target sequence. It is still unclear if the PIWI targets are RNAs, DNA sequences or even both. However, PIWI has recently been shown to be a nuclear protein involved in gene silencing of transposons and preventing their mobilization in the germ line, suggesting a role in maintaining the integrity of the genome during meiosis.

We have examined the organization in the human genome of the ~32,000 piRNA genes described so far, and have found that they map to 70,736 sites and are structured in about 400 clusters containing each at least 10 piRNA genes. Surprisingly, over 37% of total piRNA genes are located in regions that are variable in copy number (CNVs), and about 25% of them do overlap with segmental duplications (SDs). Conversely, a large proportion of SDs and CNVs do also contain piRNA gene sequences. Given that SDs and CNVs account for about 5% and 12% of the human genome respectively, the significant enrichment of piRNAs could provide valuable information about functional elements located within these genomic regions with an important role in the aetiology of genomic disorders and in genome evolution. Given the postulated role of piRNAs in other species, we hypothesize that a subset of SDs could contain elements with a functional role in the integrity of the genome at the germline level. The particular localization and variability in copy number of piRNAs provides a new dimension to explore functional variation in the human genome.

Armour, John

Developing Paralogue Ratio Tests (PRT) For High-Throughput CNV Typing In Association Studies

JOHN ARMOUR, Raquel Palla, Suhaili Abu Bakar and Susan Walker
Institute of Genetics, University of Nottingham UK

Evaluating the role of copy number variation in complex phenotypes requires copy number to be measured accurately in formats suitable for large numbers of samples. Except in those few cases for which the copy number variation can be reduced to simple qualitative assays, the development of robust methods capable of measuring copy number on a large scale is still an incompletely solved technical issue. The greatest challenge for such technologies is posed by loci at which there is common variation over higher copy numbers (for example 2-7 in the case of the 8p23.1 beta-defensins). Paralogue ratio methods were first developed for diagnosis of trisomies, and use a single primer pair to amplify products from both test and reference loci in a comparative PCR; critically, test and reference products are made by amplification processes with nearly identical properties, but can be distinguished and quantified using internal sequence variants. We have extended this approach to measure copy number variation at DEFB4 and CCL3L1/4L1 by exploiting low-copy dispersed repeats within the copy-variable unit. Precisely-placed primers amplify exactly two loci – the copy-variable unit to be measured, and an unlinked reference locus (two copies). Although not applicable to all CNVs, PRT allows inexpensive but highly accurate typing at the DEFB4 and CCL3L1/4L1 loci. We have also developed supplementary tests based on microsatellites for quality control of CNV data. Further improvements in precision, economy and throughput may be available by multiplexing several PRT analyses in a single electrophoresis capillary.

Bertranpetit, Jaume

Recombination Variation Among Human Populations, With An Insight On Recombination Evolution

BERTRANPETIT, JAUME; Graffelman Jan; González-Neira, Anna; Calafell, Francesc; Casals, Ferran
Unitat de Biologia Evolutiva, Universitat Pompeu Fabra, Barcelona

The possibilities of computing population recombination rates from SNP frequency data has open many new possibilities in the understanding of the recombination dynamics. The first question is whether recombination does vary among human groups. In the present study we show that it does in a very clear way, and with statistical robustness. When looking at the structure of variation of recombination rates among humans we find a similar partition of the variance than in the classical papers on structure of the genetic variation (that is, variation at the sequence level). This means that most of the variation in within populations, with an important fraction among major human groups and a minor component of population variation within continents.

The second question is whether variation is due to presence/absence of recombination hotspots or to their variation among human groups. There is a complex pattern in which most of the hotspots are conserved among human groups, but having a very heterogeneous value that only partially is due to sampling variation. There is an interesting pattern of variation, with Africans presenting many hotspots but not very pronounced while Euro Asiatic populations present very much pronounced hotspots in the same location.

The third question is whether the differences of pattern (presence or absence of hotspots) and of intensity of recombination may show some kind of geographic and phylogenetic structure. The answer is that it is indeed possible to relate recombination differences with the major known genetic differences among humans (traced by genetic distances and phylogeography) and thus the structure found in present populations keep the footprint of the recent and fast evolving process of recombination, whose track has been lost when compared to chimpanzee patterns.

Bhaskar, Sanjeev

High Quality, High-Throughput Quality Targeted Re-Sequencing.

SANJEEV BHASKAR, Jenny Allen, Hazel Arbury, Graeme Bethel, Ruth Bennett, Claire Bryan, Jon Burton, Lauren Dywer, Jillian Durham, Tamsin Eades, Mark Earthrowl, Catherine Evans, Lucie Farmer, Emma Gray, Simon Holroyd, Eleanor Howard, Sarah Hunt, Lars Jorgesen, Steven Leonard, Pablo Marin-Garcia, Kirsten McClay, David Niblett, Richard Norris, Elilan Somaskantharajah, Jane Rogers and Alison Coffe
Wellcome Trust Sanger Institute, Wellcome Trust Genome Campus, Hinxton, Cambridge, CB10 1SA, UK.

We have established a robust pipeline for high-throughput sequencing of PCR products, supported by a flexible LIMS, which has a capacity of up to 1,500,000 reads per month. SNPs are called using ExoTrace, a novel algorithm developed in-house. Tools have been developed for data visualisation and manual review of data where necessary. Comparisons of SNP data generated with HapMap suggests a low false negative rate and reveals a > 97% genotype concordance.

To date we have re-sequenced exons from 14 human chromosomes in 48 Caucasian individuals and identified over 83,000 SNPs, approximately 41% of which are novel. 76% of the novel SNPs have a maf of <0.1 and 10% represent non-synonymous changes. All data are available for download from the ExoSeq website at http://www.sanger.ac.uk/humgen/exoseq. A full analysis of the SNP data will be presented. The study of allelic variation in normal individuals has now been transferred to the new sequencing platforms and the re-sequencing pipeline is entirely directed towards medical re-sequencing.

Targeted medical sequencing is in progress in several areas including epilepsy, cardiovascular disease, platelet biology, deafness, diabetes, depression and cognition. Exons, putative regulatory regions, or large genomic regions identified in genome-wide association studies are being sequenced in DNA samples extracted from a variety of source materials resulting in the direct detection of mutations and the identification of additional novel SNPs. Robust, automatable, protocols have been developed for the generation of long-range PCR products and nesting techniques in order to allow amplification and sequencing of difficult regions, for example repeats, duplications, regions of high GC content. Protocols have been developed that allow routine generation of PCR products of up to 5 kb and sequencing of regions of up to 80% GC. This is critical to allow complete sequencing of whole genes.

Pilot studies are underway exploring the use of the new sequencing technologies for medical sequencing in comparison to Sanger sequencing.

Birney, Ewan

Ensembl Variation And Functional Data

EWAN BIRNEY
European Bioinformatics Institute, Wellcome Trust Genome Campus, Hinxton, Cambridge, CB10 1SD, UK

Ensembl provides an infrastructure for genomic information, from its storage through to analysis and visualisation. In genomes such as Human and Mouse, the main protein coding content is well understood and steadily progressing towards completion. In contrast other functional elements are less well understood. Using data from ENCODE, and other genome wide functional datasets we are starting to produce and display more integrated views of genome function. These new functional elements often harbor more variants than protein coding sequences, and we are exploring the interplay between variation and function. I will present some of the recent results from ENCODE and correlate them with the HapMap and resequencing datasets available in human.

Brookes, Anthony J

International Progress In Databasing Genotype-To-Phenotype Relationships

ANTHONY J BROOKES
Department of Genetics, University of Leicester, UK

Research into the genetic basis of disease is proceeding at a phenomenal and ever-increasing pace, generating unprecedented amounts of raw and processed data. It is now imperative that the scientific community finds ways to effectively manage and exploit this tidal wave of information. Efforts to this end include the development of various genotype-to-phenotype databases, genome browsers, and internet technologies - but there is very little coordination of this work. Consequently, databases are emerging slowly, data exchange between depositories is minimal, and there is little integration or standardisation of systems. This approach will struggle to produce a unified, holistic, and truly powerful internet domain that does justice to the extensive and sophisticated datasets being produced.

To improve on this situation, the European Commission has committed EUR 12M for an Integrated Project (FP7 funding, 1st Call) that will orchestrate and enhance genotype-to-phenotype databasing across Europe. A bid entitled “Genotype To Phenotype Data Systems (GEN2PHEN)”, coordinated by the University of Leicester (AJB), has now been favourably reviewed, with probably funding starting in early 2008.

There are 9 core work packages in the GEN2PHEN proposal:
1) Analysing current needs and practices in the G2P field (global perspective)
2) Developing key standards for the G2P field
3) Creating generic database components, services and integration infrastructures
4) Creating search and data presentation solutions, anchored on Ensembl
5) Promoting and facilitating data population into G2P databases
6) Building a major G2P internet portal, as a nexus for the field
7) Assisted deployment of GEN2PHEN solutions to the community
8) Consideration of system durability and long-term financing
9) Execution of a system validation pilot study, with biomedical relevance

At the HGV2007 meeting, further details of the GEN2PHEN project shall be provided, along with ongoing activities headed by AJB to build a central genetic association database.

Cambon-Thomsen, Anne

Ethics And The Challenge of Biobanks Exchange Policies

ANNE CAMBON-THOMSEN. Inserm, University Paul Sabatier Toulouse III, Faculty of medicine, France

Human biobanks are essential tools for studying human genome variation. They are set up and used in different situations. Current tendencies are to constitute large populations/patients collections for research, therapeutic or public health purposes. Although small collections often have been gathered over many years by medical doctors/researchers as a side activity of their practice and without a specific budget the trend is now towards an official recognition and identification of biobanking activity as well as organising financial sustainability through professional biobanks or Biological Resource Centres, that will apply agreed standards, like OECD guidelines (http://www.oecd.org/dataoecd/7/13/38777417.pdf). A lot has already been published about the ethical issues raised by biobanks, challenging consent procedures, protection of personal data and public dialogue. Such preoccupations were targeted on ethics towards individuals and on public/participants/researchers relations. More recently the (inter)-institutional perspective came to the forefront, as well as the researchers' interactions around biobanks management and uses. Models of governance start to be drafted. At the same time efforts are being developed to optimize networking of such biobanks (http://www.p3gconsortium.org/). Among the points of ethical tension, an issue not often discussed comes up clearly: How to share and use widely and openly the samples/data for the optimal progress of knowledge and/or for health purposes while providing means of sustainable recognition of those who constituted the collections. This is one of the major challenges that need to be addressed. In addition, in order to assure long term recognition of this activity and sustainable budget for such tools that cost a lot, their utility must not only be proclaimed but assessed through their impact on research and health care. This is not trivial. There is presently no standardised and easy way to assess the use of a biobank and to relate it quantitatively to the scientific impact of the discoveries or to health care measures associated to its use. This need leads to the concept of BRIEF (BioResource Impact Evaluation Factor). A systematic, quantitative impact factor could become an integral part of how this activity is assessed. A first step is to find a standardized way of referring to a bioresource used, in publications, that could be automatically traced. We could envision a system whereby authors cite the biobank(s) used in the references section of their paper, in an agreed format so that the Institute for Scientific Information (ISI) can track this information. Over time, this index would become a rational measure of some aspects of the impact of a researcher's or institution's biobanking activities. It would probably boost the quality of biobanks, to promote their use and the sharing of their samples/data. But sharing and full openness are not synonymous. The recent Proposed Policy for Sharing of Data obtained in NIH supported Genome-Wide Association Studies (GWAS) has triggered a debate in the genetics research community and among other stakeholders (http://grants.nih.gov/grants/guide/notice-files/NOT-OD-06-094.html). GWAS are usually performed on a large number of cases and controls, using huge number of microsatellites and/or SNP spanning the genome, thanks to technological platforms. The potential advantages of sharing such data must be balanced against various scientific concerns, as well as the critical need to protect the confidentiality of the participants in the studies for which data are shared. The proposed NIH policy relies on a central repository, a 9 months limited period of data embargo, de-identified individual data made accessible to users who will have to declare that they will protect personal data and to describe their protocol, but not to provide an external ethics committee approval. However, it is usually accepted that GWAS genotype data can never be completely de-identified, since genotypes are themselves identifiers. In such a context a number of conflicting interests must be carefully balanced. They concern: 1) Participant privacy; 2) potential risks and benefits for individual participants; 3) provision of methodological guidance for interpretation and use of data available; 3) professional recognition of investigators; 4) intellectual property rights; 5) characteristics of a centralized NIH data repository or other repository. Thus after quality standards and individual focused ethical perspective, it seems that a major challenge around biobanks governance model is the sample/data sharing management. Both top-down and bottom-up approaches based recommendations are needed to inform policy in this matter.

1. Cambon-Thomsen A, Rial-Sebbag E., Knoppers B.M. Eur Resp J, 2007; 30: 1–10
2. Cambon-Thomsen A, Sallee C., Rial-Sebbag E., Knoppers B.M. Populational Genetic Databases: Is a Specific Ethical and Legal Framework Necessary? GenEdit 2005; 3: 1-13 http://www.humgen.umontreal.ca/int/GE/fr/2005-1.
3. Cambon-Thomsen A. The social and ethical issues of post-genomic human biobanks Nat Rev Genet, 2004 5: 866-73
4. Cambon-Thomsen A. Assessing the impact of biobanks. Nat Genet (Correspondence), 2003, 34: 25-26
5. Cambon-Thomsen A., Ducournau P, Gourraud PA, Pontille D. Biobanks for genomics and genomics for biobanks. Comparative and functional genomics, 2003, 4: 628-634
6. Hirtzlin I., Dubreuil C., Preaubert N., Duchier J., Jansen B., Simon J., Lobato De Faria P., Perez-Lezaun A., Visser B., Williams G. D., Cambon-Thomsen A. An empirical survey on biobanking of human genetic material and data in six EU countries. Eur J Hum Genet, 2003, 11 (6):475-488
7. Milanovic F., Pontille D., Cambon-Thomsen A. Biobanking and Data Sharing: a Plurality of Exchange Regimes. Genomics, Society and Policy. 2007, Vol.3, No.1, (in press, on line publication, http://www.gspjournal.com/)

Carracedo, Angel

Pharmacogenetics: From Research To Clinical Use

ANGEL CARRACEDO
Galician Foundation of Genomic Medicine (SERGAS) and Genomic Medicine Group (USC)-
University of Santiago de Compostela, Spain

Pharmacogenetics (PGX) is the study of variations in DNA sequence as related to drug response. It provides the possibility of targeting drugs according to a person's genetic make-up — so-called 'personalized medicine' — although it will probably be used to stratify patient populations into groups determined by their genotype.

From the genetic point the response to a drug is a complex trait subjected to the same problems in research that any other complex trait. Many association studies on PGX have been published and only a few replicated and as a consequence PGX is only a reality for a few drugs in clinical practice. However, PGX are not only hopes but a reality in clinical practice and changes in laabelling have been recommended by the regulatory agencies in Europe and USA, EMEA and FDA, for about ten different drugs. Examples of practical application of PGX will be given. These practical applications comprise not only biomarkers aproved by EMEA or FDA but validated biomarkers or even probable valid biomarkers that are being qualified through new biomarker qualification schemes aproved by the regulatory agencies.

PGX has also a promising future for research through association studies using both candidate genes or GWA approaches. Dedicated chips including most of metabolic and transporters genes have been designed facilitating research in this area. Examples of research projects in this area carried out by our group will be shown.

Chanock, Stephen

Genome Wide Association Studies In Breast And Prostate Cancer

STEPHEN CHANOCK(1,2), Meredith Yeager(1,3), Kevin Jacobs(1), David Hunter(3,4), Robert Hoover(1) and Gilles Thomas(1) for the CGEMS team
(1) Division of Cancer Epidemiology and Genetics, NCI, Bethesda, MD
(2) Pediatric Oncology Branch, Center for Cancer Research, NCI, Bethesda, MD
(3) SAIC-Frederick, NCI-FCRDC, Frederick, MD
(4) Channing Laboratory, Harvard Medical School, Boston, MA

The Cancer Genetic Markers of Susceptibility (CGEMS) Project is conducting genome-wide association studies in breast and prostate cancer. The design includes genome wide association scans (GWAS) of approximately 1150 cases and 1150 controls in each of breast and prostate cancer drawn from prospective cohorts (PLCO and NHS cohorts for the prostate and breast cancer GWAS respectively). For each cancer, more than 7000 cases and 7000 controls are available for follow-up replication studies in two stages.

In the prostate scan, a rapid Fast Track analysis of 8q24 identified two distinct regions, marked by rs 1447295 (p=1.41 x 10-11) and rs6983267 (p=9.42 x 10-13) which independently are associated with prostate cancer risk. The estimated population attributable risk for the centromeric SNP, rs6983267 is approximately three-fold larger than rs1447295. Ancestral Recombination Graph (ARG) analyses underscore at least two distinct regions. Follow-up haplotype analyses in these two loci of 8q24 reveal strong association signals. The first replication stage consists of over 28,880 SNPs tested in approximately 4400 cases and 4400 controls from four studies. The second stage will examine approximately a comparable number of cases and controls for prostate cancer.

In the breast cancer scan in post-menopausal women, four intronic SNPs in FGFR2 were associated with risk for breast cancer (rs1219648; p=1.1 x 10-10). ARG and haplotype analysis point towards a single locus. The first stage replication will test 33,000 SNPs in 4800 cases and 4800 controls.

Charlier, Carole

Patrocles: A Database Of Polymorphic miRNA-Mediated Gene Regulation.

Hiard, Samuel; Coppieters, Wouter; Kvasz, Alex; Tordoir, Xavier; CHARLIER, CAROLE; Georges, Michel.
Centre for Biomedical Integrative Genoproteomics, University of Liège, Belgium.

The expression level of at least one third of mammalian genes is fine-tuned by one or more of a total set of ~1,000 miRNA. This post-transcriptional regulation requires a functional silencing pathway with many components involved in nuclear and cytoplasmic miRNA processing, loading of the miRNP, recognition of the target 3'UTR and actual silencing. The corresponding sequence space, i.e. target 3'UTRs, miRNA precursors and silencing machinery is bound to suffer its toll of DNA sequence polymorphisms (DSP) of which some will be functional and possibly affect phenotype. That this is indeed the case has been demonstrated by the recent identification of (i) a mutation in the 3.UTR of the human SLITRK1 gene that is causing Tourette syndrome by re-enforcing miR-189 mediated downregulation (Abelson et al., 2006), and (ii) a mutation in the 3'UTR of the ovine MSTN gene that causes increased muscle mass by creating an illegitimate target site for co-expressed miR-1 and miR-206 (Clop et al., 2006). Polymorphisms affecting miRNA-mediated regulation are thought to create hypo- or hypermorphic alleles rather than highly penetrant gain or loss-of-function mutations. They are expected to contribute to the genetic variation of traits with complex rather than Mendelian inheritance (Georges et al., 2007). To assist in the identification of DSP that affect miRNA-mediated regulation we have searched the public domain databases for SNPs and other polymorphisms in the three sequence compartments involved in miRNA control (targets, miRNA precursors and silencing machinery). The outcome of this search is browsable via the Patrocles website (www.patrocles.org).

Cheung, Vivian G.

Genetics Of Natural Variation In Human Gene Expression

VIVIAN G. CHEUNG(1,2), Richard S. Spielman(2)
Depts of Pediatrics(1) & Genetics(2), University of Pennsylvania, Philadelphia, PA 19104, USA.

Expression levels of genes, like many other phenotypes, are highly variable among individuals. We have previously shown that there is a genetic component to this variation. Our goal is to identify the genetic determinants that influence variation in human gene expression.

We have measured the expression levels of several thousand genes in lymphoblasts of members of the CEPH families. We treated the expression levels as quantitative traits, and carried out genome-wide linkage analyses to determine the chromosomal locations linked to these expression phenotypes. The results showed significant evidence of linkage for several thousand expression phenotypes (p < 3 x 10-5). For some phenotypes, the evidence of linkage is far beyond this threshold. Among the chromosomal regions that are linked to the expression phenotypes, the majority (> 70%) act in trans, and many genes have multiple regulators (QTLs) of expression. To confirm and narrow the regulatory regions identified by the linkage analyses, we are performing association analyses. In addition, we are performing molecular studies to characterize the transcriptional regulators.

In this presentation, we will show data from these genetic analyses. We will use these to describe the global landscape of variation in gene expression and of transcriptional control in the human genome.

Church, George

Personal Genome Project: Open-Source Software, Hardware, Wetware, & Elsware.

GEORGE CHURCH
Lipper Center for Computational Genetics, Harvard Medical School, MIT, Boston, MA 02115. USA

Various 'next-generation sequencing' methods, by using solid-phase polymerase colonies plus sequencing-by-ligation (SbL) or extension (SbE), bring down the cost about 100-fold. New multiplex hybridization or circle-capture methods allow targeting of 1% of the genome which might harbor 97% of the causative alleles for medical and non-medical traits. This approach can be combined with modest numbers of paired-end-tags for rearrangements and allele-specific RNA quantitation for personal-functional-genomic data. An affordable, scaleable ($1K per person) version of these assays, extensive trait/environmental questionnaires, and some imaging data are being deployed in a study aimed at a diverse 100,000 volunteers, well-informed (and tested) in advance about modern genomics, human subjects research, ethical, legal and social risks & benefits and the likelihood of wide distribution of their identifying DNA and trait data. Every aspect of the project is intended to be broadly distributed and open. (See: http://pgen.us)

Conrad, Donald F.

Genetics Of Copy Number Variation In A Founder Population

DONALD F. CONRAD, Carole Ober, Jonathan K. Pritchard
Department of Human Genetics, The University of Chicago, CLSC 505,
920 East 58th Street, Chicago, IL 60615 USA

We used the Affymetrix 500k SNP genotyping platform and a custom oligonucleotide CGH array to scan for CNVs in 750 individuals from a young founder population, the Hutterites, all of whom have been phenotyped for 30 quantitative traits. A complete genealogy relating these individuals has been constructed from a > 12,000-member pedigree.

In this presentation, we describe new methodology for integrating copy number variants (CNVs) into the study of genetic traits and discuss realistic expectations on what can be done with existing datasets. Importantly, we demonstrate ways in which the properties of a founder population may improve the identification and genotyping of copy number variation. Some downstream applications, such as whole genome association studies, are examined.

Cox, Nancy J

Direct And Indirect Assessment Of Copy Number Variation

NANCY J. COX, Abhi Shinde, Anna Pluzhnikov, William Chyr and Dan L. Nicolae

Using data from a variety of studies on several platforms, we show the utility of assessing copy number variation (CNV) in association studies for complex disorders. In addition to direct assessment of CNVs through hybridization intensity and/or aspects of genotype information, such as stretches of homozygosity, departures from Hardy-Weinberg equilibrium, and alteration in SNP call rates, we utilize an approach based on Testing UNtyped Alleles (TUNA), which allows indirect interrogation of biallelic CNVs through use of reference samples (such as the HapMap) in which LD relationships between the biallelic CNVs and SNPs can be defined. The TUNA approach enables us to assay known CNVs using platforms not thought to facilitate CNV detection, and enhances our ability to assay CNVs in other platforms as well. Preliminary studies in a variety of complex phenotypes, including type 1 and type 2 diabetes, diabetic complications, stuttering, tAML and mesothelioma confirm that CNVs will be among those variants showing reproducible associations with complex disorders.

Dermitzakis, Emmanouil T.

Causes Of Regulatory Variation In The Human Genome

EMMANOUIL T. DERMITZAKIS
Wellcome Trust Sanger Institute, Hinxton, UK

The recent comparative analysis of the human genome has revealed a large fraction of functionally constrained non-coding DNA in mammalian genomes. However, our understanding of the function of non-coding DNA is very limited. In this talk I will present recent analysis in my group and collaborators that aims at the identification of functionally variable regulatory regions in the human genome by correlating SNPs and copy number variants with gene expression data. I will also be presenting some analysis on inference of trans regulatory interactions and evolutionary consequences of gene expression variation.

Devereau, Andrew

A Variation Database For UK Diagnostic Molecular Laboratories - DmuDB

DEVEREAU, ANDREW(1,2); Burke, Edward(1,2); Taylor, Graham(3); Elles, Rob(1)
(1) National Genetics Reference Laboratory Manchester, UK; (2) University of Manchester, UK; (3) Cancer Research UK Genomic Services, Leeds, UK

In this paper we describe a database designed to allow UK molecular diagnostic laboratories to share variation data in order to support their diagnoses. These laboratories generate variation data for genes of relevance to human health to a high and measurable level of quality and report on the clinical significance of the variants that they find, but few submit this data to shared databases or published journals. The reasons for this include the time required to submit data, limitations on database or journal acceptance and a lack of databases for some genes. This valuable resource is not therefore being made available to other UK diagnostic laboratories or to the wider scientific community. This project was undertaken to provide a database which will allow UK diagnostic laboratories to share data easily and securely with each other, and possibly to publish their data to a broader audience. The database holds point mutations and small insertions, deletions and duplications plus whole exon deletions and duplications, within genes analysed within the UK National Health Service (NHS) molecular genetic testing laboratory network. Access to the database is limited to members of staff from these laboratories.

The database - www.dmudb.net - is accessed via a web browser using two different applications. The first is a text-based interface which is used for administration of the database, submission and curation of data and data searching. Submission of data is achieved either one record at a time via a web form or in bulk from a spreadsheet via an XML-based interface. An approval system is used to maintain data quality, and original submitters remain in control of their data. Submissions are based on referrals to laboratories rather than on variants alone to reflect the reports issued by the laboratories. Collection of data in this way allows occurrences of multiple variants in a patient and the patient's phenotypic and demographic data (gender, year of birth etc.) to be kept together. The second application is a graphical browser which displays variants from the database against the gene sequence and allows data from other compatible data sources such as dbSNP to be displayed alongside it. This helps to maintain quality by highlighting disagreements between variants and other data sources, and it allows data from different laboratories and different data sources to be integrated for each variant and be used for the interpretation of its clinical significance.

The first genes to be actively targeted for the database were the breast cancer susceptibility genes BRCA1 and BRCA2, with over 2,500 referrals now collected. Differences and changes in interpretation of variants are beginning to be shown, as are situations such as co-occurrences of pathogenic and unclassified variants. Strategies for dealing with these are being developed. Potential improvements to the database have been identified and are being prioritised and implemented. Chief among these are ways to enhance the use of the database in interpreting clinical significance, for example we are looking at the potential to provide a platform for applying bioinformatics techniques for analysing a variant's effect: recent work to develop best practice guidelines for missense mutation classification in the UK, Ireland and the Netherlands showed the importance of a consistent approach to application of these tools, and our experience has shown that there is a lack of experience and confidence within the diagnostic community with the application of such techniques. We are also investigating ways in which users of the database can add annotations to variants and conduct discussions: these can be used to reach and record consensus opinions on the clinical significance of each variant.

Estivill, Xavier

Copy Number Variants And Common Disorders: Filling The Gaps And Exploring Complexity

XAVIER ESTIVILL
Genes and Disease Program, Center for Genomic Regulation (CRG-UPF), Barcelona, Spain

Genome-wide association scans (GWAS) using single nucleotide polymorphisms (SNPs) have been completed for more than a dozen common disorders and over 30 new associations have been detected but these variants only explain a small proportion of the genetic component of complex disorders. Structural variation, mainly in the form of copy number variants (CNVs) has not been explored in GWAS, but they have already been reported to be associated to several complex disorders in candidate gene/region approaches. A common feature of the identified associations between CNVs and complex/common disorders so far is the presence of segmental duplications. Also, all CNV loci that have been found associated with common disorders are both complex and multiallelic. Since many CNVs are not easily tagged by SNPs, have a wide range of copy number variability, and/or fall in genomic regions not well covered by whole genome array assays, current GWAS might have missed the contribution of CNVs to complex disorders. This reveals the need for new generation arrays (some already in the market) and the use of tailored approaches to explore the full dimension of genome variability beyond the single nucleotide scale. The systematic use of genotyping platforms that explore CNVs should disclose the contribution of structural variants and facilitate the identification of the genetic basis of common disorders. New type of arrays covering CNVs and segmental duplications should facilitate the identification of regions that contain CNVs, but likely fail to detect associations to a wide range of variability in copy number. CNVs have shown an implication in rare and common genomic changes, providing biological support to several pathophysiological pathways. Comprehensive analysis of common and rare CNVs should complement the SNP for GWAS, and also offer a new dimension to the many missing clues of the genetic components of common disorders and complex traits in humans and other organisms.

Feuk, Lars

The Database Of Genomic Variants Annotating Structural Variation In The Human Genome

LARS FEUK, Junjun Zhang, Bhooma Thiruv, Jeff MacDonald and Stephen W. Scherer
The Centre for Applied Genomics, Program in Genetics and Genomic Biology, Research Institute, The Hospital for Sick Children, Toronto, Canada

The Database of Genomic Variants (http://projects.tcag.ca/variation/) is a comprehensive and curated catalogue of structural variation in the human genome. Copy number variants (CNVs) and inversions larger than 1kb in size are included. Its current content is based on 40 publications, with 6,559 CNVs and 77 inversions represented. Merging entries for structural variants that overlap yields 3,643 variable loci or regions. In the clinical diagnostic setting, comparative genome hybridization (CGH) has been introduced as a means to search for submicroscopic CNVs in the investigation of certain clinical phenotypes, and potential applications are expanding rapidly. In the research laboratory, screening of large human cohorts for CNVs is now common. Currently, one of the major problems lies in the interpretation of the resulting data. In order to address some of these issues, we are actively trying to mine available datasets for additional CNVs. We are also doing platform comparison studies in order to better understand the limitations of different approaches to identify CNVs and accurately assign breakpoints. The cost of genome-wide screening still prohibits many researchers from running large groups of control samples. It is therefore important to have access to a comprehensive list of regions already identified as CNVs in previous studies. The Database of Genomic Variants facilitates the interpretation of studies screening for CNVs, in relation to previously published work. The data are represented in table format, genome browser format, and text files available for download. The genome browser is ideal for viewing structural variation in relation to other genomic features, such as genes, clones and segmental duplications. The database has been designed to be easily navigated and suitable for all users, independent of bioinformatics experience. Here we present an overview of the database along with our future plans for its expanded content and enhanced presentation.

González-Burchard, Esteban

The Importance Of Race/Ethnicity & Genetics In Biomedical Research And Clinical Practice; Lessons From The Genetics Of Asthma In Latino Americans (GALA) Study

ESTEBAN GONZÁLEZ-BURCHARD
Departments of Biopharmaceutical Sciences & Medicine, University of California, San Francisco. USA

A debate has recently arisen over the use of racial classification in medicine and biomedical research. In particular, with the completion of a rough draft of the Human Genome, some have suggested that racial classification may not be useful for biomedical studies since it reflects "a fairly small number of genes that describe appearance,"[1] and that "there is no basis in the genetic code for race."[2] Based in part on these conclusions, some have argued for the exclusion of racial and ethnic classification from biomedical research.[3] In the United States, race and ethnicity have been a source of discrimination, prejudice, marginalization and even subjugation. Excessive focus on racial/ethnic differences runs the risk of undervaluing the great diversity that exists among individuals within groups. However, this risk needs to be weighed against the fact that in epidemiologic and clinical research, racial and ethnic categories are useful for generating and exploring hypotheses on environmental and genetic risk factors and interactions between risk factors for important medical outcomes. Erecting barriers to the collection of information such as race and ethnicity may provide protection against the aforementioned risks, however it will simultaneously retard progress in biomedical research and limit effectiveness in clinical decision-making.

Today I hope to convey the importance of Race & Ethnicity in Biomedical, Genetic and Clinical Research. I will begin by providing fundamental evidence of genetic differences between racial and ethnic populations. I will then demonstrate racially-specific differences in genetic risk for diseases including Alzheimer's Disease and HIV-resistance. Finally, I will present data from the ongoing Genetics of Asthma in Latino Americans (GALA) Study.

1. Lander ES, Linton LM, Birren B, et al. Initial sequencing and analysis of the human genome. Nature 2001; 409:860-921.
2. Venter C, quoted in interview for NY Times News Article "Do Races Differ? Not Really, Genes Show" written by N. Angier. Do Races Differ? Not Really, Genes Show. New York Times 2000.
3. Schwartz RS. Racial profiling in medical research. N Engl J Med 2001; 344:1392-3.

Gulcher, Jeff

New Platform For Discovery And Genotyping Of Copy Number Variants Reveals Much Higher Number Of Common CNVs In Human Genome

Daníel Guðbjartsson, Áslaug Jónasdóttir, Arnaldur Gylfason, Adam Baker, Gísli Másson, Ari Karason, Aðalbjörg Jónasdóttir, Aðalheiður Ólafsdóttir, Sigríður Reynisdóttir, Sigurjón A. Guðjónsson, Daniel A. Peiffer#, Karine Viaud#, Luana Galver#, Lixin Zhou#, Kenneth Kuhn#, Richard Shen#, Sarah Murray#, Unnur Þorsteinsdóttir, Kári Stefánsson, JEFF GULCHER
Decode Genetics, Reykjavik, Iceland and # Illumina, Inc, San Diego, CA, USA

Given the overlap of many of the known CNVs with genes and its effect on gene expression and function, CNVs may be an important source of disease-associated variations. We have designed the CNV features on the Illumina Infinium BeadChips, including the HumanCNV-12, HumanCNV370-Duo, and Human1M DNA Analysis BeadChips, which all offer markers designed to specifically target this new content. We targeted 4 categories of segments that we predicted were more likely to have common or rare structural variation: 1) the unSNPable genome, defined as either outlier sized gaps of more than 15kb in human HapMap SNP coverage or gaps between 5 and 15kb with two or more HapMap SNP failures based on problems due to HWE or inheritance, rather than yield, 2) segmental duplications defined as low copy number duplicons 100 bases or greater (with size-dependent sequence similarity cutoffs ranging from 96 to 100%) within 3 Mb, 3) megasatellites (which we define as tandem repeats with 500 bases or greater unit length), 4) the numerous gaps in HapMap coverage within the MHC which accounts for most of the MHC given its importance we covered the MHC with 7000 probes. Extra markers were added as needed to ensure coverage of the 2700 known CNVs (at the time of design) in the Database of Genomic Variants.

In total 14,000 segments were targeted using 55,000 SNPs or probes of invariant bases; most of which are not covered by current SNP array platforms. The new regions not already covered by the Database of Genomic Variants comprise about 7% of the human genome. Two-thirds of the loci are SNPs while the remainder are probes of invariant bases. For segments that did not have reported SNPs in dbSNP (regardless of whether they worked in HapMap), probes at invariant bases are used to measure intensity. Although the new Affymetrix 5.0 Array has 400,000 extra probes added for CNV analysis, about 8,000 of the 11,000 new segments that we have selected are missed.

Our hypothesis was that the segments that we selected are much more likely to contain CNVs than the well-behaved SNPable genome. We further hypothesized that CNVs in these segments are much more likely to have higher non-wildtype allelic frequencies in the general population, especially when in the unSNPable category. We have now run the three HapMap plates along with 100 sets of Icelandic triads. The normalized intensities of the probes within megasatellites correlated well with copy number precisely defined by restriction fragment lengths on Southern blotting. We find that there is at least an order of magnitude increase in the number of common CNVs found within this 7% of the genome with non-wild-type frequency greater than 2% beyond those found using standard Illumina or Affymetrix arrays covering the 93% of the genome. Many CNVs significantly correlated to expression in unstimulated peripheral leukocytes of genes within or near the CNV.

Gut, Ivo Glynne

High-Throughput Resequencing By Mass Spectrometry

IVO GLYNNE GUT
Centre National de Génotypage, 2 rue Gaston Crémieux, 91057 Evry Cedex, FRANCE, email: ivogut@cng.fr

The completion of the human genome sequencing project has set the stage for the systematic elucidation of gene function and identification of genes involved in common, polygenic diseases. This objective has propelled the popularity of single nucleotide polymorphisms (SNPs) and now enables genome wide association studies. In the last 18 months several products have been launched to carry out genome-wide genotyping with several hundred thousand pre-defined SNPs (Illumina, Affymetrix). Recently genome-wide association studies have helped to identify substantial numbers of disease associated genes.

Whole genome association studies with the available genotyping products have several caveats. Genotyping products were biased towards SNPs with a high minor allele frequency. If many rare mutations in a particular gene contribute to the disease association, such as in BRCA1 for breast cancer or NOD2 for Crohn's disease associations will be harder to detect. In cases like these associations will more likely be detected by sequencing. Copy number variations (CNVs) pose other technical challenges. Currently 2nd generation DNA sequencing technologies are becoming available. Their inclusion into association studies might resolve some of the issues of rare variants and CNVs. However, once the disease causing DNA variants or regions of the genome are identified technologies are required that can handle diagnostic sequencing where currently available technology is still rather costly and do not provide ample throughput.

We have recently been working on simplifying protocols for DNA sequencing using high-throughput mass spectrometers for readout without sacrificing sequencing accuracy. These protocols are based on the use of ribonucleotide incorporating DNA polymerases in primer extension reactions and PCR, followed by cleavage with NaOH and fingerprinting.

Hurles, Matthew

Towards A Comprehensive Map Of Copy Number Variation

MATTHEW HURLES
Wellcome Trust Sanger InstituteWellcome Trust Genome Campus, Hinxton, Cambridge, CB10 1SA, UK

Structural variation (SV) in the genome is extensive and yet is grossly under-ascertained. SV comprises both balanced changes and Copy Number Variation (CNV). We know that smaller CNVs are far more numerous than larger CNVs, and so improved CNV detection resolution can be expected to dramatically increase the numbers of known CNVs. The Genome Structural Variation Consortium is performing comparative genome hybridisation on a genome-wide set of tiling oligonucleotide arrays to discover all common copy number variants >500bp in size in two populations with African and European ancestry. This array set covers the assayable portion of the human genome with 42,000,000 probes with a median spacing of ~50bp. These data reveal, as expected, that previous surveys captured only 5-10% of the CNVs within a single genome. The unparalleled resolution of these arrays precisely define the boundaries of thousands of CNVs and so allow us to identify accurately functional sequences that are copy number variable, as well as providing new insights into the mechanisms generating chromosomal rearrangements.

Ionita, Iuliana

On The Analysis Of Copy-Number Variations In Genome-Wide Association Studies: A Translation Of The Family-Based Association Test

LULIANA IONITA
Harvard University, Harvard School of Public Health, Boston MA 02115. USA

We propose a generalization of family-based association tests (FBATs) to allow for the analysis of copy number variations (CNVs) at a genome-wide level. We translate the popular FBAT-approach so that, instead of genotypes, raw intensity values that reflect copy number can be used directly in the test statistic. Thereby, we do not have to rely on a genotyping algorithm for CNVs. Moreover, both inherited and de novo CNVs can be analyzed without any prior knowledge about the type of CNV, making it easily applicable to large scale association studies. All robustness properties of the genotype FBAT-approach are maintained and all its extensions, including FBATs for time-to-onset, multivariate FBATs, and FBAT-testing strategies, can be directly transferred to the analysis of CNVs. Using simulation studies, we evaluate the power and the robustness of the new approach. Further, we assess the need for CNV-genotyping prior to performing an association study, by comparing FBATs based on genotype calls with FBATs that are directly based on the intensity data. An application to a CNV genome-wide-association study of asthma illustrates the potential of the approach.

Kwok, Pui-Yan

Genome-Wide SNP Genotyping In Association Studies Of Complex Traits

PUI-YAN KWOK
Department of Dermatology, Cardiovascular Research Institute, and Institute for Human Genetics, University of California, San Francisco

As the cost of SNP genotyping falls the new SNPs sets for genome-wide association studies continue to improve, it is now feasible to perform such studies at reasonable cost and in just a few months in an average laboratory. In this presentation, we will discuss our experience in conducting a number of studies using the genome-wide SNP genotyping approach.

Lehvaslaiho, Heikki

Exchange Standard For Genomic Polymorphism And Genotyping Data

HEIKKI LEHVASLAIHO (1), and the International Biodata Interoperability Consortium
(1) South African National Bioinformatics Institute, University of the Western Cape, Private Bag X17, Bellville 7535, South Africa

Polymorphism Markup Language (PML) is an XML-based format for representing all aspects of sequence variation and genotyping data. It has been designed to unify terminology and structure of information reported by assay instruments, generated by genotyping laboratories, and stored in population allele reports and generic polymorphism databases. It is an approved Object Management Group (OMG) standard supported all major SNP databases.

Levy, Samuel

Toward The Comprehensive Identification Of Complex DNA Variants In A Single Human

SAMUEL LEVY(1), Granger Sutton(1), Pauline C. Ng(1), Lars Feuk(2), Aaron L. Halpern(1), Brian P. Walenz(1), Nelson Axelrod(1), Jiaqi Huang(1), Ewen F. Kirkness(1), Gennady Denisov(1), Yuan Lin(1), Jeffrey R. MacDonald(2), Andy Wing Chun Pang(2), Mary Shago(2), Timothy B. Stockwell(1), Alexia Tsiamouri(1), Vineet Bafna(3), Vikas Bansal(3), Saul A. Kravitz(1), Dana A. Busam(1), Karen Y. Beeson(1), Tina C. McIntosh(1), Karin A. Remington(4),John Gill(1), Jon Borman(1), Yu-Hui Rogers(1), Marvin E. Frazier(1), Stephen W. Scherer(2), Robert L. Strausberg(1), J. Craig Venter(1)

(1) J. Craig Venter Institute, 9704 Medical Center Drive, Rockville, MD 20850, USA.

(2) Program in Genetics and Genomic Biology, The Hospital for Sick Children, and Molecular and Medical Genetics, University of Toronto, Toronto, Ontario, M5G 1L, Canada

(3) Department of Computer Science and Engineering, University of California, San Diego, 9500 Gilman Drive, La Jolla, California 92093-0404, USA

(4) Your Genome, Your World, 1 Research Court, Suite 450, Rockville MD 20850

Employing a novel, high through-put, random shotgun sequencing, assembly, variant detection and haplotype assembly approach we have generated the diploid genome sequence of a single human donor. Using newly developed genome assembly strategies and comparative genome-to-genome mapping methods it was possible to identify 4.1 million DNA variants, encompassing 12.3 Mb. These variants (of which 1,288,319 were novel) included 3,213,401 single nucleotide polymorphisms (SNPs), 53,823 block substitutions (2-206 bp), 292,102 heterozygous insertion/deletion events (indels)(1-571 bp), 559,473 homozygous indels (1-82,711 bp), 90 inversions, as well as numerous segmental duplications and copy number variation (CNV) regions. Non-SNP DNA variation accounts for 22% of all events identified in the donor, however they involve 74% of all variant bases. These findings permit a detailed and personal depiction of the donor's genetic contribution and a preliminary glimpse into our understanding of personal biology.

Nickerson, Debbie

Lessons From SNP Discovery In Candidate Genes

DEBORAH A. NICKERSON
Department of Genome Sciences, University of Washington, Seattle, WA 98195

We have amplified and sequenced more than 1,000 candidate genes to identify single nucleotide substitutions and insertion-deletion (indels) polymorphisms in several human populations. The approaches we have applied will be highlighted and some of the more unusual findings from our analysis will be presented, including the identification of structural variation. We have performed a number of association studies in human populations with quantitative trait measurements to identify cis-acting variation; examples of these studies will be presented. We have also determined the effectiveness of the HapMap and different commercially available genome-wide panels in capturing the patterns of common variation in candidate genes. Our findings suggest that further SNP discovery, either genome-wide or in candidate genes, is required to increase the coverage of common variation across different human populations.

Ostell, James

Human Variation Resources At NCBI

JAMES OSTELL
National Library of Medicine, 45 Center Drive Room 5AS51, Bethesda MD 20892-6510. USA

The National Center for Biotechnology Information (NCBI) has recently deployed three major new projects in support of research into the genetic basis of human disease. The Database of Genotype and Phenotype (dbGaP) holds phenotype data from long term clinical and cohort studies such as the Framingham Heart Study or the Age Related Eye Disease Study, and is linked to large scale genotype results on the participants or to medical sequencing data in support of Genome Wide Association Studies (GWAS). The Collaboration, Education, and Test Translation (CETT) program captures the results of clinical genetic testing linked to diagnostic information into a central research database for rare diseases. The RefSeqGene project provides a standard genetic coordinate system for mutation reports from these sources, as well as new data flows from Locus Specific Mutation Databases, OMIM, and others. A summary of these projects and their interconnections with existing resources such as RefSeq, dbSNP, and the NCBI human genome annotation projects may be a helpful guide to some useful resources for researchers in the field of human variation.

Perry, George

Diet And The Evolution Of Human Amylase Gene Copy Number Variation

GEORGE H. PERRY(1,2), Nathaniel J. Dominy(3), Katrina G. Claw(2,4), Arthur S. Lee(1), Heike Fiegler(5), Richard Redon(5), John Werner(4), Fernando A. Villanea(3), Joanna L. Mountain(6), Rejeev Misra(4), Nigel P. Carter(5), Anne C. Stone(2), and Charles Lee(1,7)
(1) Department of Pathology, Brigham and Women s Hospital, Boston, MA, USA; (2) School of Human Evolution and Social Change, Arizona State University, Tempe, AZ, USA; (3) Department of Anthropology, University of California, Santa Cruz, CA, USA; (4) School of Life Sciences, Arizona State University, Tempe, AZ, USA; (5) The Wellcome Trust Sanger Institute, The Wellcome Trust Genome Campus, Hinxton, Cambridge, UK; (6) Department of Anthropological Sciences, Stanford University, Stanford, CA, USA; (7) Harvard Medical School, Boston, MA, USA

The consumption of starch is a prominent characteristic of agricultural societies and hunter-gatherer groups in arid environments. In contrast, the traditional diets of rainforest and circum-arctic hunter-gatherers and some pastoralist groups contain substantially reduced amounts of starch. This behavioral variation, in the form of diet, has led us to question whether different selective pressures have acted at the structural genomic level on the salivary amylase gene (AMY1), which encodes for an enzyme responsible for starch hydrolysis. We found that AMY1 gene number correlates positively with salivary amylase protein levels, and that individuals from populations with high-starch diets have, on average, higher AMY1 copy numbers than those with traditionally low-starch diets. Comparisons with other loci in a subset of these populations, using a BAC-based whole genome tiling path array comparative genomic hybridization platform, suggest that the level of AMY1 copy number differentiation is highly unusual. Our data are consistent with a model of positive or directional selection on AMY1 copy number in at least some high-starch populations but neutral evolution (i.e., genetic drift) on AMY1 copy number in low-starch populations.

The functional significance of AMY1 copy number variation also provides insight into the longer-term ecological and evolutionary histories of humans and non-human primates. The average human has ~3 times more AMY1 copies than chimpanzees, and outgroup comparisons with other great apes suggest that copy number was most likely gained in the human lineage, rather than lost in chimpanzees. Therefore, the initial human-specific increase in AMY1 copy number may have been coincident with an adaptive shift toward the consumption of starchy food resources early in human evolution.

Ponting, Chris

Evolutionary Dynamics Of Recurrent Copy Number Variation In The Human Genome

Duc-Quang Nguyen, Caleb Webber, Jayne Hehir-Kwa, Rolph Pfundt, Joris Veltman, CHRIS P. PONTING
MRC Functional Genetics Unit, University of Oxford, Department of Physiology, Anatomy and Genetics, Le Gros Clark Building, South Parks Road, Oxford, OX1 3QX, UK & Department of Human Genetics, Nijmegen Centre for Molecular Life Sciences, Radboud University Nijmegen Medical Centre, Nijmegen, The Netherlands

In a previous study (PLoS Genet. 2006 2:e20), we examined a set of copy number variable regions of diverse experimental origins, and then argued, from their elevated density of genes, evolutionary rates, and gene functions, that some of these regions have been positively selected in the human population due to advantageous gene dosage effects. We took advantage of newly-available data sets to examine whether copy number variants could instead be segregating in the population simply by neutral drift. We present a new data set which is unusual in containing mainly large (~0.5-1.0Mb) and low frequency, yet recurrent, variants; these regions exhibit an elevated G+C content (44%). By comparing segmental duplications that are variable, to those that are apparently fixed, in the human population, we then infer that high G+C regions are unusually susceptible to copy number variation. Finally, we provide additional evidence that human copy number variation has been subject to drift alone. Gene densities and evolutionary rates are significantly elevated within copy number variable regions; these observations cannot be explained simply by the increase in G+C content. Positive, rather than neutral, evolution thus appears to have acted upon some of these genomic regions.

Roses, Allen

Haplotypes Within The APOE LD Region May Have Pharmacogenetic Effects Predicting Efficacy Of Alzheimer's Patients Receiving Rosiglitazone

ROSES A.D., Ramamurthy L., Barnes M., Davies K., Saunders A.M.

Several genome wide association scans of large series of Alzheimer's disease patients have been presented or published recently. All confirm extremely high associations with the LD region [10^{-8} to 10^{-62}] containing the APOE gene. To date, all have focused on different associations at the borderline of Bonferroni corrections of the SNP associations – none of which seems to be the same gene and remain to be meta-analyzed. We have concentrated on studying other polymorphisms located in the APOE LD region, performing long range sequencing and evolutionary analyses of the TOMM40-APOE-APOC1 genes and sequences coded within this region. To date, there are several potentially important sets of data to suggest that multiple variants within the LD region may contribute to biological relevance. These data have suggested testable hypotheses regarding AD pathogenesis and pharmacogenetic responses. We will present pharmacogenetic data derived from the rosiglitazone Phase IIB trial of 511 patients that have demonstrated that a polymorphism which occurs only on an APOE3 background may contribute to the efficacy response of AD patients receiving rosiglitazone. Clearly, the entire APOE LD region may be so highly associated with AD because there are several additive biological effects relevant to separate polymorphisms on various APOE backgrounds - other than just the difference between APOE4, APOE3 and APOE2. Additional relevance to the effects of apoE4>apoE3 C-terminal fragment effects on mitochondrial function will also be presented. The confirmation of these data will be tested in three separate, large rosiglitazone Phase III trials now in progress.

Scherer, Stephen W

Discovery Of Structural Variation In Control And Disease Studies

STEPHEN W. SCHERER
The Centre for Applied Genomics, Hospital for Sick Children and University of Toronto

Our group is engaged in numerous studies of structural variation ranging from technology and algorithm development for discovery, to populating the Database of Genomic Variants with new control data, to assessing the role in disease. Our latest unpublished data from each of these lines of investigation will be presented.

Stein, Lincoln

The HapMap Web Site

LINCOLN D. STEIN, Marcela Tello-Ruiz, Lalitha Krishnan, Albert Smith
Cold Spring Harbor Laboratory, Cold Spring Harbor, NY 115724 USA

The HapMap web site, located at www.hapmap.org, is the primary repository and portal for the International Haplotype Map Project data set and associated analyses. This talk will discuss the features the site offers for exploring patterns of diversity and linkage disequilibrium in the HapMap populations, generating customized extracts of the data, and interactively viewing and interpreting the results of association scans. We will also discuss our progress towards providing views of resequencing data and individual genomes.

Sunyaev, Shamil

Widely Distributed Non-Coding Selection In The Human Genome

SHAMIL SUNYAEV(1); Asthana, Saurabh(1); Noble, William Stafford(2); Kryukov, Gregory(1); Grant, Charles(2); Stamatoyannopoulos John(2)
(1) Division of Genetics, Department of Medicine, Brigham and Women's Hospital, Harvard Medical School, Boston, MA 02125, USA; (2) Department of Genome Sciences, University of Washington, 1705 NE Pacific Street, Box 355065, Seattle, WA 98195, USA

It is widely assumed that human non-coding sequences comprise a substantial reservoir for functional variants impacting gene regulation and other chromosomal processes. Evolutionarily conserved non-coding sequences (CNSs) in the human genome have attracted considerable attention for their potential to simplify the search for functional elements and phenotypically important human alleles. A major outstanding question is whether functionally significant human non-coding variation is concentrated in CNSs or distributed more broadly across the genome. Here we combine whole-genome sequence data from four non-human species (chimp, dog, mouse, and rat) with recently available comprehensive human polymorphism data to analyze selection at single nucleotide resolution. We show that a substantial fraction of active purifying selection in non-coding sequences occurs outside of CNSs and is diffusely distributed across the genome. This suggests the existence of a large complement of human non-coding variants that may impact gene expression and phenotypic traits, the majority of which will escape detection using current approaches to genome analysis.

Thomas, Gilles

Impact Of Population Structure In Genome Wide Association Studies

GILLES THOMAS(1), Zhaoming Wang(1, 2), Kai Yu(1), David Hunter(3), Robert Hoover(1), and Stephen J Chanock(1, 4) for the CGEMS team
(1) Division of Cancer Epidemiology and Genetics, NCI, Bethesda, MD
(2) SAIC-Frederick, NCI-FCRDC, Frederick, MD
(3) Channing Laboratory, Harvard Medical School, Boston, MA
(4) Pediatric Oncology Branch, Center for Cancer Research, NCI, Bethesda, MD

The Cancer Genetic Markers of Susceptibility project (CGEMS) is a NCI initiative aimed at the identification of breast and prostate susceptibility genes in the European population. The first phase of the study consisted in performing two genome wide association scans (GWAS) on groups of cases and controls selected from prospective cohorts (PLCO and NHS cohorts for the prostate and breast cancer GWAS respectively). Because the European population is known to be heterogeneous and because such heterogeneity may be a potent confounder in association studies, we have attempted to characterize the population structure that may exist in the CGEMS groups of cases and controls. Several sets of thousands of SNPs with low pair-wise linkage disequilibrium were selected. These sets were used, one at a time, 1/ to evaluate the continental admixture vector of each individuals participating in the study 2/ to detect relatedness between pair of individuals 3/ to perform a principal components analysis of the genotypes. Our results indicate that reliable evidence of population structures can be found in the CGEMS data. The parameters of population structure are robust to the set of diagnostic SNPs being used. In particular, the number of significant principal components and the identification of admixed individuals or that of pairs of related individuals are little affected when the set is changed. These observations have implication on the design of genome wide scans based on the participation of self described European Americans and on the procedure to be followed in order to best correct for population stratification while searching for association.

Trask, Barbara

Recent Changes In Mammalian Chemosensory Receptor Repertoires

BARBARA J. TRASK(1), RaeLynn Endicott(1), Leo Goodstadt(2), Ralf Luche(1), Janet Young(1)
(1) Human Biology Division, Fred Hutchinson Cancer Research Center, Seattle WA
(2) Department of Human Anatomy and Genetics, University of Oxford, Oxford, UK

Six gene families encode chemosensory receptors that make it possible for mammals to detect an enormous variety of odorants, pheromones, and tastes. We have studied the evolution of the very large odorant receptor (OR) family and of the two families of putative pheromone receptors (V1R and V2R) expressed in the vomeronasal organ. All three families have undergone extensive change during the divergence of mammals, with gene birth through many duplications and gene loss through deletion or mutation. Half of mouse OR genes arose after the evolutionary split between primates and rodents. Only about one third of the OR genes inferred to have existed in the common ancestor of mouse and rat have retained a one-to-one orthologous relationship in these species. Rodent genes that underwent duplication in one species or the other, or both, show significantly higher rates of amino-acid change than the ORs maintained as one-to-one orthologs; these higher rates support the possibility that the proteins encoded by duplicate genes might have diverged to acquire the ability to respond to different odors than the ancestral receptor. Evolutionary change is even more striking in the V1R and V2R families. We have mined the genome assemblies and sequence trace archives to identify V1R and V2R family members in about 20 mammalian species. We find evidence of a remarkable degree of lineage-specific duplications that have generated largely species-specific V1R and V2R repertoires. These dramatic changes are likely to reflect strong evolutionary pressures for species-specific social signaling through pheromones. Notably, V1R and V2R families have been virtually decimated in primate and dog genomes. Given their dynamic history, it is perhaps not surprising that ORs and V1Rs are enriched in portions of genome subject to copy number variation in humans. One subtelomeric OR present in 6 to 11 apparently functional copies in different humans is an extreme example of this type of normal variation. However, enrichment of ORs in regions of copy-number variation is probably evolutionarily neutral (not selected for or against) and merely a consequence of selection against dosage changes in most other regions of the genome. Comparative sequence analyses of OR promoter regions, as well as a cis-(and possibly trans-) enhancer are providing some clues as to how the expression of OR genes is regulated such that a single receptor gene is expressed in each olfactory sensory neuron. Our work was supported by NIH RO1 DC04209.

Veltman, Joris

Copy Number Variation In Mental Retardation

VELTMAN JA
Radboud University Nijmegen Medical Centre, Department of Human Genetics, Nijmegen, The Netherlands

Mental retardation occurs in 2-3% of the general population. The underlying genetic defect remains difficult to diagnose in the majority of patients suffering from mental retardation. In addition, little is known about the biological pathways involved in this disease. Recent developments in genomic microarray technology now allow for the genomewide detection of submicroscopic chromosomal alterations, and this has been demonstrated to significantly improve the diagnostic yield in this patient group. This technology has rapidly matured and now appears ready for widespread introduction in a routine clinical setting. The major challenge now lies in the correct clinical interpretation of the many copy number variants that can be detected on these arrays. In this presentation I will discuss various aspects of this diagnostic application and present data from a collaboration with two other European diagnostic centres and Affymetrix, in which we assessed the use of Mapping 500k SNP arrays for molecular karyotyping in patients with mental retardation. Also, I will discuss the use of copy number analysis as a tool to identify novel candidate genes in mental retardation.

Yip, Yum Lina

Archiving Single Amino Acid Polymorphisms In The UniProtKB/Swiss-Prot Knowledgebase

YUM L YIP and the Swiss-Prot group
Swiss Institute of Bioinformatics, CMU, 1 Rue Michel-Servet, CH-1211 Geneva, Switzerland.

Single nucleotide polymorphisms (SNPs) represent about 90% of human DNA sequence variation. Among the SNPs, coding non-synonymous SNPs (nsSNPs) or single amino acid polymorphisms (SAPs) are the one most related to diseases. This is not surprising as an alteration in protein sequence can directly influence the structure or the function of the protein. Currently, numerous central genomic databases record information on SNPs. These databases are mostly gene-centric and provide limited information on the structural and functional consequences of SAPs.

UniProtKB/Swiss-Prot (Swiss-Prot) (http://www.uniprot.org/) is a manually curated knowledgebase providing information on protein sequences and functional annotation. It is part of the Universal Protein Resource (UniProt) (http://www.uniprot.org/) that also includes the automatically annotated UniProtKB/TrEMBL section. Although Swiss-Prot is not a SNP-oriented database, it currently records 31,356 SAPs in 5,824 human proteins (Release 52.4, 1 May. 2007). Nearly all SAPs are derived from literature reports. For each SAP, the knowledgebase provides, apart form the position of the mutation and the resulting change in amino acid, information on the effects of SAP on protein structure and function, as well as their involvement in diseases. Presently, there are 15,757 disease-related SAPs, 13,627 polymorphisms and 1,972 unclassified variants recorded in the database. In order to increase the coverage of SAPs and to maintain the database up-to-date, two measures are taken in Swiss-Prot. First, additional data is imported from dbSNP, a central repository of genetic variation that includes both high quality SNPs as well as candidate SNPs. Only SNPs validated by frequency and/or double hit, or SNPs that are described in the literature are integrated into Swiss-Prot. Second, text-mining techniques are used to retrieve document relevant for SAPs annotation.

Relevant information on SAPs can be found in various sections of a Swiss-Prot entry (e.g. the "Features" table, the "Comments" section and the "References" section). In addition to these, cross references to human gene databases (OMIM, GeneCards, GeneLynx, Genew) as well as other gene-specific databases, are being added continually. In 2004, the Swiss-Prot variant pages were created to provide a concise view of the information related to the SAPs recorded in the Swiss-Prot database [1]. The pages also provide access to additional structural information, including 3D homology models. Recently, novel features are being added in the Swiss-Prot variant pages. These include (1) the display of conservation score of the SAP at sequence and structural level; (2) the display of residues involved in protein-protein interactions, and (3) the display of the local structural environment of SAP. More specifically, the presence of 'special' residues (e.g. residues involved in ligand binding, post-translationally modified residues) in the structural neighborhood of the SAP is shown. This display option is supported by a regularly updated database providing Swiss-Prot - PDB mapping.

When compared to the information on missense variants listed in other mutation databases, it should be noted that Swiss-Prot does not simply catalogue amino acid changes predicted from nucleotide variations, but it stores, when available, information on direct protein sequencing and characterization including post-translational modifications (PTM). This is important, as the real effect of missense variants on proteins, such as PTM and structural phenotype, cannot be deduced from simple translation of single nucleotide substitutions at the DNA level. The direct link to OMIM entries (when exist) further enhance the integration of phenotype information with data at protein level. In this regard, SAP information in Swiss-Prot complements nicely those exist in genomic and phenotypic databases, and is valuable for the understanding of SAPs and diseases.

Reference:

1. Yip, Y.L.; Scheib, H.; Diemand, A.V.; Gattiker, A.; Famiglietti, L.M.; Gasteiger, E. and Bairoch, A. The Swiss-Prot variant page and the ModSNP database: a resource for sequence and structure information on human protein variants. Human Mutation, 2004; 23(5):464-70.

Abstracts for Company Presentations

AFFYMETRIX

Affymetrix Genome-Wide SNP 6.0: Pure Power and Performance

Marcus Hausch

The Genome-Wide SNP 5.0 and SNP 6.0 array are based on the next generation whole genome genotyping platform from Affymetrix. The SNP 5.0 and SNP 6.0 demonstrate industry leading performance with average call rates of >99% and the ability to detect both SNPs and CNVs with a single assay and array. The SNP 6.0 features over 906,000 SNPs and over 946,000 non polymorphic probes providing the genetic community with the most advanced tools to understand the genetic components of complex diseases like Alzheimer's, diabetes, heart disease and others.

The SNP 6.0 provides the most leading edge coverage in the market place. Even more importantly it provides the highest genetic power for a budget and enables study designs, where both the initial whole genome screen as well as the statistical replication will be done at the whole genome level. This approach will facilitate more international collaboration to meta analyse standardized whole genome marker sets to cross validate associations from different cohorts (Ref. 1,2,3,)

1.) Saxena et al. Science Express published online April 26th. "Genome-Wide Association Analysis Identifies Loci for Type 2 Diabetes and Triglyceride Levels"

2.) Zeggini et al. Science Express published online April 26th "Replication of Genome-Wide Association Signals in U.K. Samples Reveals Risk Loci for Type 2 Diabetes"

3.) Scott et al. A Science Express published online April 26th "Genome-Wide Association Study of Type 2 Diabetes in Finns Detects Multiple Susceptibility Variants

AGILENT

High Resolution aCGH Technology For The Discovery And Characterization Of Copy Number Variation

High Resolution Discovery and Characterization of Copy Number Variation in Apparently Healthy Individuals Using Custom Oligonucleotide DNA Microarrays

Stephen Laderman, PhD. Senior Manager Life Science & Nanotechnology, Agilent Laboratories, Santa Clara, California, USA

The discovery and characterization of copy number variations (CNVs) in healthy individuals will help researchers determine the genetic basis of disease. This task is far from complete, and due to the resolution of detection systems used, the majority of loci reported so far are relatively large. In order to detect CNVs that encompass a wide range of sizes and locations, we have identified ~16 million oligonucleotide probes suitable for DNA microarray-based hybridization assays employing genomic DNA within the non-repeat masked regions of the genome, at an average and median spacing of ~100 bp and ~50 bp, respectively. These probes were selected using an empirical model that utilizes scores for homology, thermodynamics, secondary structure, and sequence complexity, in order to predict their relative performance in the assay. We used a workflow for the analysis and visualization of CNV data that includes statistically robust approaches for calling CNV intervals in individual samples as well as methods for grouping variants from multiple samples into CNV regions (CNVRs).

We evaluated the performance of the platform by profiling DNA from thirty individuals from the HapMap collection using two custom 244K feature arrays with probe selection focused on previously reported CNVRs.2 We observed an average of 11 multi-probe calls in three self-self hybridization experiments; in contrast, an average of 451 multi-probe CNVs were called on autosomes within our replicated differential sample set. These 451 CNV calls were highly reproducible. In addition, the boundaries of the regions were called consistently; more than 85% of the boundaries agreed to within a single probe. The variations observed within the thirty HapMap samples overlapped the reported variations with greater than 90% concordance, but many regions were found to be significantly smaller. In another study, DNA from eight individuals from the HapMap collection were examined with two custom 244K arrays focused on intervals identified as variant with respect to the reference assembly by fosmid end-sequencing.3 Concordance of genotyping calls for thirty-five variant regions that had been previously characterized by sequencing and PCR was 98%. Finally, applying a two-stage high resolution array approach to analyze fifty healthy Caucasian males from northern France, we found variants detected by more than one consecutive probe could be clustered into 1469 CNVRs, of which 721 are thought to be novel.4 These 721 novel regions contain 367 genes, 150 of which are represented in the Online Mendelian Inheritance in Man (OMIM) database. A further 6089 putative variants were detected by single probes: 48% of these were observed in more than one individual and 2662 lie outside previously reported CNVRs. Taken together, the results of these three studies show that highly reproducible, sensitive, and specific microarray methods can reveal new CNVs, clarify the boundaries and structure of known CNVs, and uncover how CNVs vary across populations.

1 Additional Agilent employees engaged in these studies: Nick Sampas, Amir Ben-Dor, Anya Tsalenko, Alicia Scheffer-Wong, Peter Tsang, Alice Yamada, Zohar Yakhini, Laurakay Bruhn.
2 Work performed in collaboration with George H. Perry and Charles Lee, Brigham & Women's Hospital and Harvard Medical School, Boston MA.
3 Work performed in collaboration with Greg Cooper, Tera Newman, and Evan Eichler, University of Washington, Seattle, WA.
4 Work performed in collaboration with Adam de Smith, Phillipe Frougel, and Alexandra Blakemore, Imperial College London, London, UK.

A Brief Overview Of Additional Array Applications Beyond CGH And CNV (Expression, miRNA, Methylation and ChIP)

Didier Goidin, PhD, Application Scientist, Agilent Technologies Europe

ROCHE APPLIED SCIENCE

The Genome Sequencer FLX System from Roche: Long Read Length As Basis For Superior Re-Sequencing And De Novo Sequencing Performance

Dr. Jan Korbel

After the launch of its 2nd generation sequencing system GS20 in 2005, Roche Applied Science has launched its next version sequencer, the Genome Sequencer FLX, in 2007.
FLX means flexibility and reflects the enormous applications versatility of the system. Due to its outstanding combination of read length and throughput and its significantly improved sequence accuracy, a broad variety of break-through sequencing applications can be addressed at superior data quality and data completeness. It offers the broadest range of applications for research fields such as cancer research, genetic diseases, infections diseases, plant genomics, metagenomics and many more.

The presentation will focus on applications possible to address with the Genome Sequencer FLX, with special emphasis on the discovery of genetic variations such as SNPs, short indels or large structural variations, and will also highlight the future of the system.

APPLIED BIOSYSTEMS

SOLiD™-ifying the Findings of Genome-Wide Association Studies by Next-Generation, High-Throughput Sequencing

Francisco M. De La Vega*, Fiona Hyland*, Jon Sorenson*, Heinz Breu*, Heather Peckham‡, Joel Malek‡, and Kevin McKernan‡. Applied Biosystems, 850 Lincoln Centre Dr., *Foster City, CA 94404; Applied Biosystems, ‡Beverly, MA, USA

Genome-wide associations studies have flourished and are starting to return many disease-associated regions and alleles. However, for many of these associations the identity of the causative variants have not been established, in part due to extensive likage disequilibrium. An important application for next-generation sequencing (NGS) platforms is the comprehensive resequencing of association regions for the discovery and genotyping of a whole range of sequence variants including SNPs, rare mutations, indels, and copy number, in order to firmly establish the diease causing alleles and functional regions. Nevertheless, at present NGS platforms are challenged to address the large number of samples required to obtain statistical significance in association analyses. Thus, methodologies to effectively select specific genomic regions for re-sequencing, multiplex and utilize pooled samples, and to properly utilize paired-end reads for identifying structural variation, are necessary. We are developing a library "barcoding" scheme that would enable to pool DNA samples from different subjects for sequencing by oligonucleotide ligation and detection (Applied Biosystems SOLiD™ System), allowing the identification of each read source. Although variant discovery from Sanger sequencing reads is reasonably well understood, NGS platforms present new challenges not only for the massive amount and short lenght of the reads (25-35bp), but also due to the different underlying error models that are critical to distinguish false positives and negatives. We developed a model of digital genotyping in the presence of error. We discover that accurate genotyping and measurement of allele frequency of variants is critically dependant on the read substitution error rate. Heterozygote calling with 99.5% confidence in shotgun digital sequencing becomes a counting exercise that requires at least 15X depth of coverage at substitution error rates of ≈ 0.01%. Increasing the coverage cannot overcome error rate, which imposes the lower limit of detection when measuring alleles in pooled samples. Our results suggest that even if coverage needs to increase significantly when using short reads as compared with di-deoxy sequencing, low platform substitution error rate is paramount for utilizing next generation sequencing platforms in genetic epidemiology studies.

Abstracts for Poster Presentations

Abnizova, Irina

Inferring Functionality To Regulatory SNPs

ABNIZOVA, IRINA; Foco, Luisa, Naumenko, Fedor, te Boekhorst, Rene and Bernardinelli, Luisa
MRC-BSU, Cambridge, UK; University of Pavia, Italy; University of Hertfordshire, UK

This work is devoted to the analysis of human variations in complex human diseases and combines the strengths of both genetics and genomics by investigating single nucleotide polymorphisms (SNPs) in regulatory regions instead of genes. By bringing together the computational search and characterisation of regions in DNA that regulate gene expression on the one hand and information about individual variation in the structure of human DNA on the other hand, it aims to identify likely regulatory regions, the individual variation in their molecular make up and the effect this may have in the phenotypic expression of genes. As a result, we developed a computational method for inferring the possible effect of SNPs in the development of human diseases.

Currently, there is strong interest in regulatory SNPs [1-7]. Combined experimental and computational evidence has demonstrated that the promoter regions of human genes provide a rich source of functional single nucleotide polymorphisms [4-7]. There are, however, currently almost no computational tools (but see [7] for a tool exclusively applied to promoters) that can be used to assess directly from a sequence of regulatory DNA whether or not a given variant is likely to alter gene expression and hence is of functional significance.

Here, we present a method that enables a computational estimation of the likely functional consequences of single nucleotide changes in putative regulatory DNA. It is based on the integration of at least 16 sources of supervised sequence information about a given DNA stretch in combination with unsupervised methods [8,9]. We have also incorporated a novel method that analyses the possible functionality of a SNP. Essentially, this method consists of identifying regions in the human genome that are likely to be important in the regulation of gene expression and contains over- and under-represented motifs that identity them as putative Transcription Factor Binding Sites. We then establish whether the motifs contain SNPs and if so, in how far these mutations destroy the signal by which regulatory proteins recognize the motifs as binding sites. Especially these SNPs could be strong candidates for further experimental verification to establish their possible role in the genesis of and susceptibility for particular diseases.

Results. To test the method, we collected several known disease-associated regulatory SNPs from the literature [1-3]. We found indeed that functional disease-associated regulatory SNPs were predicted by our method. Furthermore, we found that certain SNP variants, known to play a role in the genesis of certain diseases, formed significantly underrepresented motifs.

References
1. Monsuur AJ, et al. (2005) Nat Genet. 37:1341-4.
2. Ueda H, Howson JM, et al. (2003) Nature 423:506-11.
3. Morahan G, et al., (2001) Nat Genet. 27:218-21.
4. Hoogendoorn, B., et al (2004), Hum. Mutat. 24, 35-42.
5. Hudson, T. J. (2003). Wanted: regulatory SNPs. Nat. Genet. 33, 439-440
6. Paul R. Buckland et al., (2005) Hum. Mutat. 24, 35-42.
7. Khan I, et al. and Chuzhanova N. (2006) *In Silico* Biology 6, 0003
8. Irina Abnizova, Alistair G. Rust, Mark Robinson, Rene te Boekhorst and Walter R. Gilks, (2006) Prediction of TFBS using Markov models, J. of Bioinformatics and Comp. Biology, v4, n2, pp 425-441
9. Irina Abnizova, Rene te Boekhorst, Klaudia Walter and Walter R. Gilks, (2005), BMC Bioinformatics, 6:109

Adamusiak, Tomasz

Analysis Of The Downstream Sequences In Relation To Genes Expression, Cytosolic Phospholipase A2 (cPLA2) Signal Transduction And Peroxisome Proliferator-Activated Rreceptor Response Element (PPRE) Consensus Sequence.

TOMASZ, ADAMUSIAK; James H, Shelhamer; Rafal, Pawliczak
Department of Immunopathology, Faculty of Postgraduate Medical Education, Medical University of Lodz; Critical Care Medicine Department, Warren Grant Magnuson Clinical Center, National Institutes of Health, Bethesda, MD

Introduction: Peroxisome proliferator-activated receptor (PPAR) interacts with retinoid X receptor (RXR) on PPAR response elements (PPREs) and subsequently regulates transcription of PPAR-responsive genes. It has been shown that cytosolic phospholipase A2 (cPLA2) is able to activate IL-8 and COX-2 expression at least in part through PPAR. An intact DNA-binding domain is not required for successful PPAR activation, and almost always the consensus sequence is at least to some extent degenerated. There are only a few studies that actually investigate into how this level of degeneration relates to PPAR binding. Temple et al demonstrated that the 3 half site is more influential on the PPARgamma/RXR heterodimer binding. However no dependable non-laboratory methods of identifying PPAR-responsive genes exist.

Methods: The aim of this study was to assess the bioinformatic approach to identifying PPAR-responsive genes. Some 400 genes, with previously measured positive response levels to cPLA2 activation in a microarray assay, were considered as candidates for PPAR regulation. We analyzed their putative promoter elements (-10000 downstream sequences based on NCBI Entrez Nucleotide reference assembly) for frequency, position, maximum and average similarity to PPRE consensus. Halfsites and less conservative sequences were also considered. Spearman R statistic was used for testing correlations and Multiple Linear Regression to assess different PPREs models.

Results: On average PPREs richer sequences tended towards the origin site. Although the correlation between maximum similarity to PPRE consensus and expression change, dependant on cPLA2 pathway, was not established. However, the average similarity to a standard PPRE consensus had a small, borderline significant correlation with the expression levels ($R = -0.10$; $p = 0.042$). This improved for a PPRE variant with a conservative 3 half-site ($R = -0.15$; $p < 0.01$). On the other hand, in a subset of genes ($n = 60$), that increased their expression at least twofold, the average similarity to a standard PPRE consensus correlated positively with the expression levels ($R = 0.32$; $p = 0.01$) and the sequences in question were found significantly closer to the origin site only on the positive strand.

Conclusions: Similarity of the downstream sequence to PPREs could partially explain the effect of signal transduction through cPLA2 on genes expression. The results may suggest the existence of different concurring pathways through PPAR. Further study seems necessary to elucidate the molecular basis.

Agueda, Lidia

A Haplotype-Based SNP Selection For Comprehensive Assessment Of Genetic Variation Of LRP5

L AGUEDA(1), M Bustamante(1), N Garcia-Giralt(2), S Jurado(2), X Nogues(2), L Mellibovsky(2), S Balcells(1), A Diez-Perez(2), D Grinberg(1)
(1)Dept Genetics, UB, CIBERER, IBUB; (2)URFOA, IMIM, HM, UAB. Barcelona, Spain

LRP5 encodes the low-density lipoprotein receptor-related protein 5, involved in Wnt signalling. LRP5 is an important regulator of osteoblast growth and differentiation and mutations in it result in severe bone phenotypes. In order to evaluate the role of its common variation in relation to osteoporotic phenotypes, a haplotype-based approach was used to select the minimum set of SNPs to comprehensively examine it. The selection of haplotype-tagging SNPs was based on the Caucasian reference panel of HapMap. Gentotypes from region Chr11:67829880-6780145, including LRP5 (136.6kb) and 6.8kb of each flanking region, were downloaded and analyzed with Haploview 3.32. A MAF cut off was set at 1% which led to a final density of 1SNP/2kb. Two different block definition were applied: Confidence Intervals method and the Four Gamete Rule. For each resulting block, the minimal combination of SNPs that identified the >95% of the haplotypes was selected. Subsequently, LD-Select was used to identify highly correlated (r2>0.8) SNPs among this subset in order to avoid redundant genotyping. Finally 22 htSNPs were selected, which constituted an 85% reduction of total SNPs (149) and 69% reduction of HapMap validated SNPs (70) in this region.

According to the Confidence Intervals method, 6 blocks were determined with an average size of 13.4kb which covered 54% of the region, and were represented by 2-3 tags per block (14 htSNPs in total). According to the Four Gamete method, 8 blocks were determined with average size of 11.4kb which covered 60.5% of the region, and were represented by 2-3 tags per block (20 htSNPs in total). Considering both methods together, the average block size was 12.4kb and the coverage of the region achieved 62.3%. An average of 3 major haplotypes per block was consistent with limited haplotype diversity. Other approaches for indirect association studies point the selection of tagging SNPs based in terms of the LD measure r2, independently of block structures. If we consider this strategy, our collection of 22 SNPs is covering 91.4% of the known genetic variation (SNPtagger software). The 22 selected htSNPs were included in a 48-plex and genotyped simultaneously by SNPlex at the Genotyping platform of the Centro Nacional de Genotipado (CEGEN, Barcelona) in a cohort of Spanish women (n=964). Nineteen of the SNPs were successfully genotyped (pass rate 86.4%) with an average of 95.6% call rate. The genotyping of the three remaining htSNPs was completed by Taqman. For these the average call rate was slightly lower (86%). Quality control was assessed by re-genotyping 6% random samples by PCR-RFLP (>95% reproducibility). Additional quality control on one LRP5 SNP was performed by typing it with both technologies (99.5% concordance). Genotypes from the Spanish women were analyzed to obtain MAFs, LD values and haplotypic frequencies. These data was then compared to the equivalent values in the original CEU-HapMap population. Allele frequencies were consistent between the two populations, with a high positive Pearson correlation (R=0.801, p<0.0001). All the polymorphisms were in Hardy-Weinberg equilibrium except for one (p=0.026). Since quality control did not show evidence of genotyping errors, this departure is likely to have occurred by chance. LD coefficients (r2) were highly correlated (R=0.828, p<0.0001) between both populations and haplotype frequencies, for both block definitions, were also highly correlated (R=0.917, p<0.0001 for Confidence Intervals method and R=0.946, p<0.0001 for the Four Gamete). In conclusion, the HapMap Caucasian population may be an appropriate reference panel for selecting htSNPs for a further gene-based association analysis performed in this Spanish cohort. HapMap has proved effective in selecting tagSNPs that allow the capture of the majority of common SNPs (MAF>5%) when applied to other cohorts.

Ahn, Joo Wook

Developing Diagnostic ArrayCGH For Constitutional Cytogenetics – CNV Issues

JOO WOOK AHN, Sally Walsh, Kathy Mann, Caroline Ogilvie
Cytogenetics Department, Guy's and St Thomas' NHS Foundation Trust, UK

Array technology is being introduced in our hospital for the diagnosis of submicroscopic imbalance in patients with idiopathic mental retardation and/or congenital abnormalities. Currently, a loop strategy is used where patients are hybridised against other phenotypically distinct patients (A vs B, B vs C, C vs A, all in dyeswap, one array/patient). To date, we have tested 78 patients using this strategy, and have found that, on average, 6 probes/case indicated abnormal copy number (normal log2 ratios defined as 0 +/- 4SD, 1 Mb BAC platform); all such abnormal results were followed up by interrogation of CNV databases, and 62% (293/476) were found to be due to previously-reported CNVs. For the remaining copy number changes, FISH probe development for confirmation and family studies is carried out and has shown that in some cases, these changes have been inherited from phenotypically normal parents. Therefore we are now considering a trio strategy where each patient is hybridised once against mother and once against father. This strategy retains two tests per patient in dyeswap, and most importantly only identifies de novo abnormalities. Preliminary piloting of this approach has indicated that the additional consumable cost (two arrays/patient) is more than compensated for by efficiency and cost savings in analysis, interpretation and follow-up testing. As array platforms increase in resolution, these savings are likely to become even more significant.

Aka, Peter

Genetic Variation In DNA Repair Genes May Increase Genotoxicity In Radiation Workers

AKA P(1, 3), Mateuca R(1), Buchet, J, Thierens H(2), and Kirsch-Volders M(1)
(1). Laboratory of Cell Genetics, Free University of Brussels, Brussels, Belgium; (2). Department of Biomedical Physics and Radiation Protection, University of Ghent, Ghent, Belgium; (3). Medical Research Council Laboratories, The Gambia, West Africa

Minor genetic variations, known as single nucleotide polymorphisms (SNPs) may play an important role in promoting susceptibility to diseases as well as the response of the individual to various drugs and environment/carcinogens.

We performed a preliminary study in 32 male seasonal cleaners of a nuclear plant and 31 control workers to assess the predictivity of the hOGG1, XRCC1 and XRCC3 genotypes and DNA repair capacity in the induction of genotoxic effects (DNA damage and micronuclei). At the population level, a significant contribution of the hOGG1 genotypes to DNA repair capacity was found. Genetic polymorphisms in XRCC1 and XRCC3 did not significantly influence repair capacity. At an individual level, the hOGG1 variants Ser/Cys and Cys/Cys genotypes showed a slower *in vitro* DNA repair than the Ser/Ser hOGG1genotype. A multivariate analysis performed with genotypes, age, cumulative dose, exposure status and smoking as independent variables indicated that in the control population, repair capacity was influenced by age and polymorphisms in hOGG1. In the exposed population, DNA damage was higher in older men and in smokers. Repair capacity was slower in individuals with Ser/Cys or Cys/Cys hOGG1 genotypes compared to those with the Ser/Ser hOGG1 genotype. Micronuclei frequencies increased with age and the cumulative dose of gamma rays. Overall genetic polymorphisms in XRCC1 resulted in higher residual DNA damage and the Met/Met variant of XRCC3 resulted in an increased frequency of micronuclei. This preliminary analysis confirms that micronuclei frequencies are reliable biomarkers for the assessment of genetic effects in radiation workers.

As a follow up on our previous studies, we have further developed a haplotype tagging SNP set to capture the genomic diversity in the region of the hOGG1 XRCC1 and XRCC3 genes. We will use the SNP set to type people exposed to radiation (n=500) and controls (n=500) to assess whether inherited variations in these DNA repair genes are truely linked to the development of genotoxicity.

References

Aka P, Mateuca R, Buchet J P, Thierens H and Kirsch-Volders M 2004 Are genetic polymorphisms in OGG1, XRCC1 and XRCC3 genes predictive for the DNA strand break repair phenotype and genotoxicity in workers exposed to low dose ionising radiations? Mutat. Res. 556 169.181

Balmain A, Gray J and Ponder B 2003 The Genetics and Genomics of Cancer; Nature Genet. 33 238

Alakulppi, Noora

Kidney Transplantation Genetics

NOORA ALAKULPPI; Jukka Partanen; Jarmo Laine
Finnish Red Cross Blood Service, Research and Development, Helsinki, FINLAND

Kidney transplantation is the treatment of choice for end-stage renal disease. Transplantation was made possible with the invent of immunosuppressive medications. Immunosuppressants are given to prevent rejection. However, over-immunosuppression can lead e.g. to infection, cancer or nephrotoxicity. The major contributors for immunological acceptance of graft are the HLA (human leukocyte antigen) genes. Mismatches in HLA alleles between a recipient and a donor influence the graft survival. The search for other genes associating to the graft outcome and enabling individualized treatment continues. The graft function is monitored by non-specific clinical and laboratory markers. Graft biopsy is an invasive procedure used to diagnose e.g. rejection. Non-invasive and robust biomarker diagnostics are needed for rejection recognition.

My thesis divides into two parts. First part consists of studies aiming at finding associations between candidate gene polymorphisms and kidney transplantation outcomes. Second part addressed the searching for gene expression biomarkers to diagnose clinical and subclinical rejection.

Recipient candidate gene polymorphisms in TNF and IL10 were associated to rejection. Donor TNF polymorphism was associated to delayed graft function. In addition, donor IL10 polymorphisms were associated to cytomegalovirus incidence and later infection occurrence in a subpopulation of recipients. Further, thrombosis associated polymorphisms were studied from recipients and donors. Recipient or donor polymorphisms were not associated to thrombosis, infarction, rejection or graft survival.

In the study of rejection biomarkers, whole blood samples were prospectively collected from adult patients. Real-time quantitative PCR (RT-QPCR) was used to quantify the gene expression of cytotoxic and co-stimulatory lymphocyte molecules. CD154 and ICOS differentiated rejection patients from control patients but not from patients with other causes of graft dysfunction.

Subclinical rejection biomarkers were studied from pediatric patients. RT-QPCR was used to quantify gene expression from candidate genes in low-density array format and to validate results from microarray analysis. MALAT1 was found to differentiate subclinical rejection from normal patients.

In genetic terms, kidney transplantation can be regarded as a complex trait. There are several still unknown genetic factors influencing the outcome of transplantation. In addition, their interplay with environmental factors makes all genetical studies very complicated. Further, more biomarkers are needed to sufficiently separate patient populations and to have clinical value.

References:
Alakulppi NS et al. Cytokine gene polymorphisms and risks of acute rejection and delayed graft function after kidney transplantation. Transplantation. 2004 Nov 27;78(10):1422-8.
Alakulppi NS et al. The impact of donor cytokine gene polymorphisms on the incidence of cytomegalovirus infection after kidney transplantation. Transpl Immunol. 2006 Nov;16(3-4):258-62.
Alakulppi NS et al. Diagnosis of acute renal allograft rejection by analyzing whole blood mRNA expression of lymphocyte marker molecules. Transplantation. 2007 Mar 27;83(6):791-8.
Alakulppi NS et al. Lack of association between thrombosis associated candidate gene polymorphisms and acute rejection or vascular complications after kidney transplantation. Submitted.
Alakulppi NS et al. Diagnosing subclinical renal allograft rejection in children by whole blood gene expression analysis. Submitted.

Albert, Thomas

Microarray Strategy Combining Genome Copy Number Analysis And Exon Selection Coupled To High-Throughput Sequencing

THOMAS ALBERT, Roland Green, Rebecca Selzer, Christina Middle, and Matt Rodesch
NimbleGen Systems, Inc. Madison WI, USA

We have developed a microarray-based approach to select genomic regions from the human genome that can then be sequenced using next-generation, high-throughput sequencing instruments. The strategy can purify either large contiguous regions of several Mb in size, or tens of thousands of small discontiguous regions of a few hundred bases. We have used this approach to select and sequence several thousand exons from genes suspected to be involved in human cancers. We demonstrate that the selection array can also serve a dual purpose, identifying copy number variation in samples while selecting exons for sequencing. This approach provides a simple strategy for identifying a broad range of genomic variation in targeted regions of the human genome.

Albrechtsen, Anders

Estimating Local Relatedness For Genome-Wide Data In The Presence Of Linkage Disequilibrium

ALBRECHTSEN, ANDERS; Nielsen, Rasmus
Department of Biostatistics, Copenhagen University, Denmark; Center for Bioinformatics, Copenhagen University, Denmark

Estimates for relatedness have several applications such as identification of relatives or in linkage mapping. Many methods have been proposed but the new high throughput data, such as SNP-chip data, give new applications for relatedness and pose new problems. Unlike a sparse set of markers the new data allows for identification tracks of relatedness and the identification of distant relatives. The problem with current methods is that they either ignore the genomic context of the markers, assumes a known level of identity by descent (IBD), and/or does not take linkage disequilibrium (LD) into account.

Here we present a continuous time Markov model where the hidden states are the number of IBD between pairs of individuals. We allow both the length of the tracks and the degree of IBD sharing to be unknowns and estimate them using the Baum-Welch algorithm. Instead of using single markers to calculate the emission probabilities we use emission probabilities based on haplotypes thereby taking local LD into account.

After training of the markoc model IBD tracts can be estimated providing a map of local relatedness across the genome.

Aledo, Rosa

Expression Analysis Of The C.1-25 C>G Polymorphism Of The LRP1 Gene Promoter

R. ALEDO(1), P. Costales(1), V. Llorente-Cortes(1), C. Ciudad(2), V. Noe(2), A. Tapias(2), R Älonso(3), P Mata(3), J. Villar(4), L Badimon(1)

(1) Cardiovascular Research Center, CSIC-ICCC, Hospital de la Santa Creu i Sant Pau, Barcelona, Spain
(2) Biochemistry and Molecular Biology Departement, School of Pharmacy, University of Barcelona, Spain
(3) Fundación Jimenez Díaz and Fundación Hipercolesterolemia Familiar, Madrid, Spain
(4) Hospital Virgen de las Nieves, Sevilla, Spain

Background: Low density lipoprotein receptor-related protein (LRP1) is a multiligand binding protein that plays a pivotal role in the atherotrombotic transformation of vascular cells among other functions. Alterations in LRP1 expression might be relevant for the onset and progression of vascular disease. The aim of this study was to analyse polymorphisms in LRP1 promoter relevant for LRP1 gene transcriptional activity and premature clinical manifestation of atherosclerosis.

Methods and Results: The promoter and flanking regions (bp -1242 to +429) were amplified by overlapping fragments and analysed by SSCPs and sequencing in an initial sample of 86 hFH subjects. We found the polymorphism c.1-25C>G, already described by Shulz et al., (2002), that putatively creates a new GC transcription box. This polymorphism was analysed in 354 hFH subjects and 257 controls (Repository of Genoma España and samples donated by Dr. J.Villar). Its frequency was not significantly different between hypercholesterolemic patients and controls (16%), neither between hFH with or without atherosclerosis (P>0,05). However, a significant difference in the frequency of the G allele was found in hypertensive hFH patients (3.4% in hypertensives vs 16.7% in normotensives). The association of the allele G with blood pressure in this sample was tested by logistic regression adjusted by age, gender and body mass index using http://bioinfo.iconcologia.net/SNPstat, results showed that the presence of G allele seemed to be a protective factor against hypertension in this sample, P=0,013, OR=0,16; IC95: 0,04-0,68.

The functionality of the c.1-25 C>G polymorphism of LRP1 gene was analysed by transfection studies in HeLa cells with wild type or mutated LRP1 promoter constructions into luciferase gene vector. The luciferase activity was reduced to 73+/-1.39 (P<0,05, anova test) when the C>G change was introduced by site-directed mutagenesis. In cotransfections studies with Sp1 and Sp3 factors, commonly binding to this GC box, Sp1 acts as an activator and Sp3 as an inhibitor of the transcriptional activity. Electrophoretic mobility shift assays performed with nuclear extracts from HeLa cells and antibodies against Sp1 and Sp3 confirm the functionality of this new GC box by the specific binding of transcription factors.

Conclusions: The presence of the polymorphism c.1-25 C>G in the promoter of LRP1 gene is not associated with premature clinical manifestation of atherosclerosis in this hFH sample, but it seems to be associated with normal blood pressure in hFH patients.

The observed reduction in the transcriptional activity of the LRP1 gene associated with this polymorphism could act as a protective factor against other risk factors affecting atherosclerosis. Further study seems warranted.

Altmäe, Signe

Variations In Estrogen Receptor Alpha Gene (ESR1) Predict The Outcome Of Ovarian Stimulation In *In Vitro* Fertilization

ALTMÄE, SIGNE; Haller, Kadri; Peters, Maire; Hovatta, Outi; Stavreus-Evers, Anneli; Karro, Helle; Salumets, Andres; Metspalu, Andres.
Institute of Molecular and Cell Biology, University of Tartu, Estonia Estonia and Karolinska Institutet, Stockholm, Sweden

Introduction
Infertility is a widespread medical and social problem affecting up to 15% of the couples of fertile age. Several methods can be used to treat infertility; *in vitro* fertilization (IVF) is the most successful among them. Still, only ~30% of women get pregnant after the IVF procedure. The outcome of *in vitro* fertilization (IVF) depends substantially on the effectiveness of controlled ovarian hyperstimulation (COH) by administration of follicle stimulating hormone (FSH). Endogenously produced estrogens extend the action of exogenous FSH in promoting folliculogenesis in COH. In the current study we determined the association between genetic variations in ESR1 and ESR2 genes and different causes of infertility, and analyzed the influence of these variations on the COH outcome in regards to the age and clinical parameters of IVF patients.

Material & methods
PvuII T/C (rs2234693) and XbaI A/G (rs9340799) SNPs, and (TA)n microsatellite polymorphism in ESR1 gene as well as RsaI G/A (rs1256049) SNP and (CA)n microsatellite polymorphism in ESR2 gene were genotyped in 159 IVF patients.

Results
Women's age was associated with poorer COH outcome in linear manner. In addition, patients with endometriosis represented diminished ovarian response to FSH compared to tubal factor infertility. ESR1 PvuII T/C, XbaI A/G and ESR2 RsaI G/A were associated with the length of the microsatellites of the respective genes. Shorter ESR1 (TA)n gave a higher risk for unexplained infertility. Longer ESR1 (TA)n repeat in association with ESR1 PvuII C allele were associated with better COH outcome in an age-independent manner.

Conclusions
Variations in ESR1 gene, in addition to the woman s age, predict the outcome of COH in IVF.

Future perspectives
For better understanding and improving the infertility treatment, our next focus will be conducting a large-scale SNP analysis on genes involved in the FSH regulatory pathway among infertile women.

Barbadilla, Antonio

Bioinformatics Of Nucleotide Diversity

BARBADILLA, ANTONIO; Casillas, Sònia; Egea, Raquel; Petit, Natalia
Departament de Genètica i Microbiologia, Universitat Autònoma de Barcelona 08193 Bellaterra, Barcelona, Spain

Nucleotide sequences available in public databases can be used to describe and to interpret the patterns of nucleotide diversity in a broad range of species and genes. However, population genetics have not available a secondary database that provide the available polymorphic sets with their associated estimates. Aiming to perform a comparative analysis of pattern of polymorphism among species and genes, we have created a novel software, PDA, Pipeline Diversity Analysis(1) that automatically can (i) search for polymorphic sequences in large databases, (ii) detect and validate SNPs from the aligned sequences and (iii) estimate their genetic diversity. Sequence sets are grouped depending on their similarity scores. Main diversity parameters, including polymorphism, synonymous and non-synonymous substitutions, linkage disequilibrium and codon bias can be estimated for different functional regions.

In the new version of PDA have been introduced new methods for data mining and grouping sequences, and new criteria for data quality checking. PDA is a powerful tool to obtain and synthesize existing empirical evidence on genetic diversity in any species or species group and has been used to create an on-line database of SNPs and genetic polymorphism in Mammals (MamPol2). A data model for the storage, representation and analysis of haplotypic variability was defined based on the 'polimorphic set' as the basic storing unit: a group of homologous sequences for a given gene and species. With the information available in MamPol we have performed a large-scale study of adaptive selection of nucleotide substitution. We used the McDonald-Kreitman test, which was implemented in a freely available web server application. The main results of this multi-locus and multi-species analysis of divergence and polymorphism will be summarized.

1 Casillas, S & A. Barbadilla (2004). PDA: a pipeline to explore and estimate polymorphism in large DNA databases. Nucleic Acid Research, Web Server issue 32: W166-W169.
2 Egea, R, S. Casillas, E. Fernández, MA Senar & A. Barbadilla (2006). MamPol: a database of nucleotide polymorphism in the Mammalia class. Nucl. Acids Res. doi: 10.1093/nar/gkl833

Barber, John

Chromosomal Copy Number Variation

JOHN BARBER
Wessex Regional Genetics Laboratory and National Genetics Reference Laboratory (Wessex), Salisbury NHS Foundation Trust, Salisbury District Hospital, Salisbury, SP2 8BJ, UK
Human Genetics Division, Southampton University Hospitals Trust, Southampton, SO16 6YD, UK

Twenty-seven families have been added to the latest version of the Chromosome Anomaly Collection (www.ngrl.org.uk/Wessex/collection/) which contains 227 families with 149 cytogenetically visible unbalanced chromosome abnormalities (UBCAs) and 78 euchromatic variants (EVs) which have been directly transmitted from parents to children. These UBCA and EV families are divided into three groups depending on the presence or absence of an abnormal phenotype in parents and offspring.

No detectable phenotypic effect was evident in 25/149 (17%) (Group 1) ascertained mostly at prenatal diagnosis. Among these was a family with a transmitted duplication of 6.2 Mb from 12q21.31 to 12q22 including 48 genes of which several have roles in cell proliferation, apoptosis and cancer. The average size of these Group 1 deletions was 8 Mb and of the duplications 14 Mb. In another 34/149 (23%) families, the affected proband had the same UBCA as other phenotypically normal family members (Group 2). Bias of ascertainment, imprinting and a variable phenotype extending into the normal range are likely to explain the discordant phenotype within many of these families. These transmitted imbalances indicate that deletions of gene poor regions and duplications of regions of moderate gene density are compatible with a normal phenotype. In addition, a number of the gene poor regions contain multiple related genes which may help explain their apparent haplosufficiency.

Most of the UBCAs were unique to a family but additional familial diad deletions of distal 3p are consistent with the idea of "chromosomal non-penetrance". Our understanding of genotype/phenotype correlations is also challenged by proximal 1q within which transmitted 10 Mb duplications of 1q21-q22 can be found in normal family members while small transmitted duplications and deletions within 1q21.1 are found in affected children and normal parents as are the 200 kb deletions associated with thrombocytopenia absent radius (TAR) syndrome. We have also found that large 4-8 Mb segmentally duplicated tracts of chromosome 9 can be deleted and duplicated without phenotypic consequences.

The Chromosome Anomaly Collection is also being extended to include the 69 transmitted subtelomeric imbalances in the literature of which the 4/69 (6%) were group 1 and 49/69 (71%) were group 2. With 37.5% of abnormalities found using array CGH present in one of the parents, the continuum of severity associated with UBCAs and the variability of the genome at the sub-cytogenetic level will require further close collaboration between medical and laboratory staff to distinguish clinically silent variation from pathogenic rearrangement.

Bayés, Mònica

Exploration Of 19 Serotoninergic Candidate Genes In Adults And Children With Attention-Deficit/Hyperactivity Disorder Identifies Association For 5HT2A, DDC And MAOB

M Ribasés(1), JA Ramos-Quiroga(1,2), A Hervás(3), R Bosch(1), A Bielsa(1), X Gastaminza(1), J Artigas(4), Silvia Rodriguez-Ben(3), X Estivill(5,6,7), M Casas(1,2), B Cormand(8,9,10) and M BAYÉS(5,6,7)
(1)Department of Psychiatry, Hospital Universitari Vall d Hebron, Barcelona, Catalonia, Spain; (2)Department of Psychiatry and Legal Medicine, Universitat Autónoma de Barcelona, Catalonia, Spain; (3)Child and Adolescent Mental Health Unit, Hospital Mútua de Terrassa, Barcelona, Catalonia, Spain; (4)Unitat de Neuropediatria, Hospital de Sabadell, Corporació Sanitària Parc Taulí, Barcelona, Catalonia, Spain; (5)Genes and Disease Program, Center for Genomic Regulation (CRG), UPF, Barcelona, Catalonia, Spain; (6)Centro Nacional de Genotipado(CeGen), Barcelona, Catalonia, Spain; (7)CIBER Epidemiología y Salud Pública, Instituto de Salud Carlos III (CRG), Barcelona, Catalonia, Spain; (8)Departament de Genètica, Facultat de Biologia, Universitat de Barcelona, Catalonia, Spain; (9)CIBER Enfermedades Raras, Instituto de Salud Carlos III, Barcelona, Catalonia, Spain; (10)Institut de Biomedicina de la Universitat de Barcelona (IBUB), Catalonia, Spain

Attention-deficit/hyperactivity disorder (ADHD) is a common psychiatric disorder in which different genetic and environmental susceptibility factors are involved. Several lines of evidence support the view that at least 30% of ADHD patients diagnosed in childhood continue to suffer the disorder during adulthood and that genetic risk factors may play an essential role in the persistence of the disorder throughout life span. Genetic, biochemical and pharmacological studies support the idea that the serotonin system participates in the etiology of ADHD. Based on these data we aimed to analyze SNPs across 19 genes involved in the serotoninergic neurotransmission in a clinical sample of 451 ADHD patients (188 adults and 263 children) and 400 controls with no DSM-IV ADHD symptoms using a population-based association study. Several significant associations were found after correcting for multiple testing: i) the DDC gene was strongly associated with both adulthood (P=0.00053; OR=2.17) and childhood ADHD (P=0.0017; OR=1.90); ii) the MAOB gene was found specifically associated with the adult ADHD phenotype (P=0.0029; OR=1.90) and iii) the 5HT2A gene showed evidence of association only with the combined ADHD subtype both in adults (P=0.0036; OR=1.63) and children (P=0.0084; OR=1.49). Our data support the contribution of the serotoninergic system in the genetic predisposition to ADHD, identifying for the first time common childhood and adulthood ADHD susceptibility factors, associations that are specific to ADHD subtypes and one variant potentially involved in the continuity of the disorder throughout life span.

Belbin, Olivia

Modeling The Effects Of Regulatory Polymorphisms On Cognitive Decline In Alzheimer's Disease Patients

OLIVIA BELBIN(1), Janette L. Dunn(2), Helen Beaumont(3), Donald Warden(3), David Smith(3), Noor Kalsheker(1), Kevin Morgan(1)
(1)Division of Clinical Chemistry, Institute of Genetics, Queen's Medical Centre, University of Nottingham, NG7 2UH, UK; (2)School of Physics and Astronomy, University of Nottingham, University Park, Nottingham NG7 2RD, UK; (3)OPTIMA, Oxford Centre for Gene Function, Department of Physiology, Anatomy & Genetics, Oxford, OX1 3PT, UK.

Alzheimer's disease (AD) is a complex neurodegenerative disorder characterized by the formation of senile plaques (SP) and neurofibrillary tangles in the brain. These plaques result from extracellular deposition of b-amyloid protein (Ab). Intracellular Ab1-42 is proteolytically derived from amyloid precursor protein (APP) by β- and γ-secretases, and induces neuronal cell death resulting in severe cognitive impairment.

Mutations in APP and presenilin genes, PS1 and PS2, are known to cause familial early-onset AD, which account for around 5% of AD cases. Genetic associations for the remaining 'sporadic AD' cases, other than the risks associated with the apolipoprotein (APOE) ε4 allele, are currently not fully established. The complexity of AD pathology has produced a large number of candidate genes, which have principally been studied using traditional case-control single SNP association analyses. As a result, lack of statistical power has been a major confounder for unraveling subtle genetic effects. An alternative approach is to model the effect of SNPs in AD candidate genes on cognitive performance. The advantage of using a quantitative trait such as cognitive decline is a substantial gain in statistical power.

In this study, 28 regulatory region SNPs from four candidate genes were selected based on involvement of their respective proteins in A² formation or removal. These included APP, the A² precursor, PS1, a protein involved in ³-secretase cleavage of APP, and two significant constituents of AD senile plaques; APOE and alpha-1-antichymotrypsin (ACT).

Tests of cognition were administered using the CAMCOG score system to 169 AD patients every 6 months throughout the course of the disease. The scores were fit to a non-linear model, upon which, genotype-specific effects for each SNP were investigated in turn. In addition to the APOE µ4 allele, x SNPs (x in APP, 4 in PS1, 2 in APOE and 1 in ACT) affected the rate of cognitive decline. Interestingly, the ACT SNP was also associated with an earlier age of decline. This suggests that SNPs in four AD candidate genes affect different parameters (rate and age) of cognitive decline. Further larger studies need to be conducted investigating these parameters in order to reveal any potential epistatic interactions between these genes.

Below, Jennifer

Gene-Environment Interaction In Mesothelioma

BELOW, JENNIFER; Pluzhnikov, Anna; Steele, Ian; Mossman, Brooke; Pass, Harvey; Testa, Joseph R.; Carbone, Michele; Cox, Nancy J.
The University of Chicago, Chicago, IL; University of Vermont, Burlington, VT; New York University, New York, NY; Fox Chase Cancer Center, Philadelphia, PA; University of Hawaii, Honolulu, Hawaii.

About 50% of deaths in three villages in Cappadocia, Turkey, are attributed to malignant mesothelioma (MM) (BARIS and GRANDJEAN 2006; BARIS et al. 1978). Although MM is typically associated with asbestos exposure in the industrialized world, the epidemic in Cappadocia has been linked to exposure to erionite, a fibrous zeolite mineral suggested to be a potent carcinogen for MM. Preliminary pedigree analysis demonstrates that in these villages, MM is common in some families and not in others, and when high-risk MM family members marry into a low-risk family, MM appears in the descendents. Analysis of a 6-generation extended pedigree of 526 individuals revealed that MM has a transmission pattern consistent with an autosomal dominant genetic model, with reduced penetrance due perhaps to gene-environment interaction. Additionally, MM does not seem to develop in members of high-risk families born and raised outside these villages (CARBONE et al. 2002). In the complex etiology of MM, environmental carcinogens and genetic factors may cause malignancy alone, in concert, or synergistically. To identify genes that confer susceptibility to mesothelioma, families in the Cappadocian villages of Karain, Old Sarihidir, and Tuzkoy that are at very high risk for MM have been identified, and pedigrees of these families have been established. DNA is being collected from both non-malignant (blood) cells and tumor biopsies of living and deceased members of these families. Analyses of these data will focus on both traditional methods of linkage and association analysis, and novel approaches designed to take into account the genomic information on association, DNA copy number variation and the possibility that affected individuals will share genetic risk factors identical-by-descent from a recent common ancestor. We present preliminary results of the studies primarily focused on the novel methods of analysis.

BARIS, Y. I., and P. GRANDJEAN, 2006 Prospective study of mesothelioma mortality in Turkish villages with exposure to fibrous zeolite. J Natl Cancer Inst 98: 414-417.
BARIS, Y. I., A. A. SAHIN, M. OZESMI, I. KERSE, E. OZEN et al., 1978 An outbreak of pleural mesothelioma and chronic fibrosing pleurisy in the village of Karain/Urgup in Anatolia. Thorax 33: 181-192.
CARBONE, M., R. A. KRATZKE and J. R. TESTA, 2002 The pathogenesis of mesothelioma. Semin Oncol 29: 2-17.

Bhardwaj, Anshu

Comprehensive Scheme To Prioritize Mitochondrial Disease Markers

ANSHU, BHARDWAJ(1); Shipra, Sharma(2); Vinod, Scaria(2); Mitali, Mukherjee(2); Shrish, Tiwari(1)
(1)Centre for Cellular and Molecular Biology, Hyderabad India; (2)Institute of Genomics and Integrative Biology, Delhi, India.

Establishing pathogenicity of a sequence change in mitochondrial DNA is a major difficulty and many attempts have been made in this direction [1,2,3]. Given the highly polymorphic nature of the mitochondrial DNA, along with other heterogeneties, it becomes mandatory to assess sequence changes on a wide-range of parameters. To this end, we have developed a novel comprehensive scheme with the objective to prioritize mutations based on their published role in biochemical defect, functional studies in cell lines (transmitochondrial cybrids and single muscle fibre PCRs), multiple independent reports and mutations without any specific background nucleotide co-occurrence. A few pathogenicitity criteria have been adopted from published reports [3], in addition to the predictions made by SIFT, PolyPhen, PLHOST and PHD-SNP for protein-coding genes and a compensatory evolution based method for the tRNA genes. Besides, mutations in regulatory regions, age of onset and nuclear modifiers are other parameters that are also considered. Thus, our method has the most comprehensive set of parameters to assess mtDNA mutations. A novel feature of the method is to score individuals harboring these variations that may help in selection of pathogenic mutations and evaluation of mitochondria in disease predisposition. Patients and normal individuals are scored on the basis of the variations present in the two groups based on predictive parameters. This method is automated by developing PERL scripts. Ataxia (93 - Indian patients) and mtSNP (entire mitochondrial genome sequences of Japanese individuals belonging to 7 different groups - 96 patients each) patients have been analyzed for pathogenic mutations. The analysis generated a small set of putative pathogenic markers for these datasets. In addition to published reports, the mutations are also assessed for their functional significance and possible role in disease etiology using various *in silico* tools, as this overcomes the undesired bias toward better-studied diseases and genes. Based on this, our method selected a few novel mutations that can be studied further to assess their pathogenic role by performing functional assays. With this method, the large number of variations observed in these datasets is prioritized thus reducing the number of potent markers to ~ 10%. In addition to assigning pathogenicity, the scheme is customized to distinguish normal individuals from patients. Our analysis of the above datasets indicates its high prediction power with Matthews correlation coefficients and other performance estimating parameters, like accuracy, ranging from 0.8 to 0.9. We tested this prediction by comparing normal datasets and by supervised and unsupervised SVM (Support Vector Machine) methods. Our analysis of the 8 datasets (1 ataxia + 7 mtSNP) supports the criteria selected for pathogenicity. The scheme developed here could be implemented as a regular prescreening method to prioritize mitochondrial variations. This scoring scheme has been automated and is implemented in a user-friendly webserver, that is customized not only for scoring mutations but also for accurately finding and mapping mutations from the sequence data. We expect the performance of this method will improve in future regular updates as a result of the advent of additional literature, better association studies with enhanced sample sizes and a widespread production of standardized genome-wide association data.

References:

1. McFarland R et al. Trends Genet. 2004 (20), 591-596.
2. Kondrashov FA. Hum Mol Genet. 2005 (14), 2415 2419.
3. Mitchell AL et al. J Med Genet. 2006 (43), 175-179.

Blasiak, Janusz

Polymorphisms Of The Rad51 And hOGG1 Gene In Gastric Cancer: Genotype-Phenotype Correlation

BLASIAK, JANUSZ(1); Poplawski, Tomasz(1); Arabsk,i MichaB(1); Morawiec, Zbigniew(2); and Chojnacki, Jan(3)
(1)Department of Molecular Genetics, University of Lodz, Lodz, Poland; (2)N. Copernicus Hospital, Lodz, Poland; (3)Department of Gastroenterology and Internal Diseases, Medical University of Lodz, Lodz, Poland

The cell's susceptibility to mutagens and its ability to repair DNA lesions are important for cancer induction, promotion and progression. Both the mutagen's sensitivity and the efficacy of DNA repair may be affected by variability in many genes, including DNA repair genes. The hOGG1 gene encodes glycosylase of base excision repair and RAD51 codes for a key protein in homologues recombination repair. Both can be involved in the repair of oxidative DNA lesion, which can contribute to stomach cancer. In the present work we determined the level of basal and oxidative DNA damage and kinetics of removal of DNA damage induced by hydrogen peroxide in peripheral blood lymphocytes of 30 gastric cancer patients and 30 healthy individuals. The metrics from DNA damage and repair study were correlated with the genotypes of common polymorphisms of the hOGG1 and RAD51 genes: a G -> C transversion at 1245 position of the hOGG1 gene producing a Ser -> Cys substitution at the codon 326 (the Ser326Cys polymorphism) and another G -> C transversion at 135 (5.-untranslated region) of the RAD51 gene (the G135C polymorphism). The level of DNA damage and the kinetics of DNA repair were evaluated by alkaline single cell gel electrophoresis (comet assay) and oxidative and alkylative DNA damage were assayed with the use of DNA repair enzymes: endonuclease III (Nth) and formamidopyrimidine-DNA glycosylase (Fpg), recognizing oxidized DNA bases. The genotypes of the polymorphism were determined by restriction fragment length polymorphism PCR. We observed a strong association between gastric cancer occurrence, impaired DNA repair in human lymphocytes and the G/C genotype of the G135C polymorphism of the RAD51 gene. Moreover, there was a strong correlation between that genotype and stomach cancer occurrence in subjects with high level of oxidatively damaged DNA. We did not observe any correlation between the Ser1245Cys polymorphism of the hOGG1 gene and gastric cancer. Our result suggest that the G135C polymorphism of the RAD51 gene may be linked with gastric cancer by the modulation of the cellular response to oxidative stress and this polymorphism may be a useful additional marker in this disease along with the genetic or/and environmental indicators of oxidative stress.

Bosch, Elena

Evidence For Positive Selection In The Human Olfactory Receptor OR5I1 Gene: Putting The 'More' In The 'Less-Is-More' Hypothesis

Andrés Moreno(1), Ferran Casals(1), Anna Ramírez-Soriano(1), Baldo Oliva(2), Francesc Calafell(1), Jaume Bertranpetit(1), ELENA BOSCH(1).
(1) Unitat de Biologia Evolutiva, Facultat de Ciències de la Salut i de la Vida, Universitat Pompeu Fabra, 08003 Barcelona, Catalonia, Spain; (2) Grup de Bioinformàtica Estructural (GRIB-IMIM), Facultat de Ciències de la Salut i de la Vida, Universitat Pompeu Fabra, 08003 Barcelona, Catalonia, Spain.

The human olfactory receptor repertoire is reduced in comparison to mammals and to other non-human primates. Nonetheless, this olfactory decline opens an opportunity for evolutionary innovation and improvement. In the present study, we analyse an olfactory receptor gene, OR5I1, which had previously been shown to present an excess of amino acid replacement subsitutions between humans and chimpanzees. We describe the genetic variation for OR5I1 in worldwide samples and find an excess of high frequency derived alleles in all populations. Additional evidence for selection includes negative, and often significant, neutrality statistics. We suggest that positive selection has modelled the pattern of variation found in the OR5I1 gene. Moreover, we provide evidence of the structural and putatively functional relevance of one of the nonsynonymous polymorphisms defining the presumably adaptive protein form of the OR5I1. These results are compatible with a relatively ancient, mild selective sweep predating the Out of Africa expansion of modern humans.

Bosch, Nina

Expansion Of The FAM90A Primate Specific Gene Family, A Copy Number Variant Located On Human Chromosome 8p23.1

NINA BOSCH(1); Mario Cáceres(1); Maria Francesca Cardone(2); Anna Carreras(1); Ester Ballana(1); Mariano Rocchi(2); Lluís Armengol(1); Xavier Estivill(1), 3
(1) Genes and Disease Program, Center for Genomic Regulation (CRG-UPF), Barcelona, Catalonia, Spain; (2) Department of Genetics and Microbiology, University of Bari, Bari, Italy; 3Experimental and Health Sciences Department, Pompeu Fabra University, Barcelona, Catalonia, Spain

Following the characterization of the segmental duplications found flanking the 8p23.1 region, we have detected the presence of FAM90A, a novel multiple copy gene family. FAM90A, which stands for 'family with sequence similarity 90%', is composed of at least 25 copies according to the last human genome assembly (NCBI build 36), and appears to be polymorphic in human general population. Quantitative PCR enabled us to observe differences up to 6.5 fold increase between general population individuals with respect to FAM90A copy number. Among the FAM90A copies, we have been able to discern two subfamilies according to their genomic structure: subfamily I (single copies on proximal duplicons and chromosome 12p13.31) and subfamily II (clustered copies on 8p23.1 distal duplicons). Members of subfamily I are composed of six exons, while subfamily II members lack the first 1kb, shortening these copies from six to five exons. Evidences for the transcription of both subtypes of copies have been obtained by RT-PCR assays.

Further in-silico analyses on different genomes reveal that FAM90A is specific of the primate lineage, showing an expansion along the great apes. Comparative FISH analysis in chimpanzee, gorilla, orangutan and macaque have confirmed the expansion of FAM90A in primates. On Rhesus macaque, subfamily II copies are present on chromosome VIII, while subfamily I members are absent. Thus, we propose a mechanism by which these copies could have evolved and expanded through the primate lineage based on the assumption that subfamily II members are the ancestral ones. The FAM90A cluster constitutes a very good example of how segmental duplications and rearrangements can promote the formation of new gene sequences with potential important functional consequences.

Brión, Maria

Highthroughput Mutation Profiling In Hypertrophic Cardiomyopathy

BRION M(1), Allegue C(1), Monserrat L(2), Hermida M(2), JR Gimeno(3), F Marín(3), Castro-Beiras A(2), Carracedo A(1).
(1) Grupo de Medicina Xenomica, CEGEN-IML, University of Santiago de Compostela, Spain; (2) Juan Canalejo Hospital, A Coruña, Spain; (3) Hospital Universitario Virgen de la Arrixaca, Murcia, Spain. Red RECAVA.

Hypertrophic cardiomyopathy (HCM) is a myocardium primary disease caused by mutations in sarcomeric proteins genes. HCM affect 1:500 adults in general populations and it is the most frequent cause of sudden death in youth and in sportmen. The clinical presentation of the disease is very variable, and the identification of patients at risk for sudden death has low sensitivity and specificity. The prognosis is highly dependent of the specific mutation responsible for the disease, but we have very few data on genotype-phenotype correlations.
The establishment of high throughput technologies for SNP typing has allowed the development of mutation analysis screening tools that allow the detection of a large number of mutations related with any disease or character of interest. Specifically, in the case of HCM, more that 600 mutations in 16 genes are already associated with the disease, making the genetic analysis with classical technologies rather difficult and time consuming.

The flexibility and variability of new genotyping chemistries and platforms improves the choice of approach, so making the analysis of mutation, on a large scale, easier and cheaper. Based on this knowledge we have developed a mutation screening tool to quickly analyse 600 mutations associated with HCM using the Mass Array System of Sequenom. Mutations are distributed in 31 iPLEX assays, which are based on single base pair extension using modified nucleotides, with a conversión rate (successful incorporation of a mutation into a working assay) over 90%.

In order to validate the accuracy and sensibility of the gentic test developed more than 1000 Spanish individuals from families known to be at risk of HCM have been analysed, with an average succes rate per sample of 91%

Brugts, Jasper

Pharmacogenomics Of Angiotensin-Converting Enzyme Inhibition In Patients With Stable Coronary Artery Disease. Pergene: A Substudy Of The Europa-Trial.

J.J. BRUGTS(1), M. de Maat(2), E. Boersma(1), J. Danser(3), C. van Duijn(4), J. Witteman(5), A. Uitterlinden(6), M.L. Simoons(1).
From the departments of Cardiology (1), Haematology (2), Pharmacology (3), Genetic Epidemiology (4), Epidemiology & Biostatistics (5), Internal Medicine and Genetic Laboratory (6) of the Erasmus MC, Rotterdam, The Netherlands.

The Renin-Angiotensin-Aldosteron System (RAAS) plays an important role in the pathogenesis of cardiovascular disease. Recent investigations of the angiotensin-converting enzyme, angiotensinogen, and angiotensin II type 1 receptor genes have led to the discovery of several polymorphisms, which may be implicated in the pathogenesis of cardiovascular disease. These polymorphisms may influence the risk of cardiovascular events and modify the treatment benefit of ACE-inhibitors.

Setting:
The EUROPA study is a randomized double-blinded clinical trial, which enrolled 12,218 patients. After a mean follow-up of 4.2 years, 8.0% of patients randomized to perindopril and 9.9% of those randomized to placebo reached the primary endpoint (cardiovascular death, myocardial infarction or resuscitated cardiac arrest), which yields a 20% relative risk reduction with perindopril (HR 0.80; 95% CI 0.71-0.91). However, cost-effectiveness of such treatment may be addressed since absolute benefits are modest in relatively low risk populations. In the clinical studies, the cardioprotective effects of perindopril were consistent among all predefined subgroups. Thus, simple clinical characteristics cannot be used to distinguish patients who do and do not benefit from treatment with an ACE-inhibitor. The new field of pharmacogenomics can have an important role in the cost-effective selection of patients for prophylactic treatment.

Study objectives:
During the run-in period all patients (n=12218) were treated with perindopril and mean blood pressure decreased from 137/82 to 128/78 (mean difference -9/4 mmHg), however in 25% of the patients no reduction in blood pressure occurred (mean difference > 0 mmHG). Furthermore, about 800 patients withdraw from treatment during the follow-up period due to side-effects or intolerance. We will investigate whether RAAS-polymorphisms are related to the amount of blood pressure reduction with an ACE-inhibitor and with the side effects of ACE-inhibitors. In addition we will investigate whether RAAS-polymorphisms are related to incident adverse cardiovascular events during 4 years of follow-up in a sufficiently large sample size. In the selection of SNPs, we used the haplotype-tagging approach to cover as much common variation as possible (>90%) in RAAS-genes (ACE, AGT, AGTR-1, AGTR-2, REN, BK-1, BK-2, Aldosteron Synthase). Functional SNPs and SNPs located in promoter regions are preferred. DNA samples were available in 10497 patients.

The PERGENE-substudy is the first large cardiovascular pharmacogenomic study that will systemically investigate genetic associations of the renin-angiotensin-aldosteron system (hypothesis-driven) in multiple haplotype tagging-SNPs (40) in multiple genes together (8) in relation to cardiovascular events, drug response, and side effects of ACE-inhibitors. A genetic profile may identify patients at specific risk of cardiovascular events and individualize (secondary) preventive therapy in patients with stable coronary artery disease. This will lead to further developments in tailored-therapy in cardiovascular medicine.

DNA-isolation of the samples was done using an automated Autogen (NA3000) robot. Currently SNPs are genotyped on high-throughput Taqman 7900HT machines. Preliminary results can be presented at the HGV 2007 meeting.

Brunet, Anna

Analysis Of Schizophrenia Positive And Negative Syndrome Scale (PANSS) Symptoms And Association With NTRK2 And NTRK3

ANNA BRUNET(1,2,4), Mònica Gratacòs(1,3), Jordi Real(4), Vicenç Vallès(5), Maite Garolera(5), Joaquin Valero(6), María José Cortés, Antonio Labad, Roser Guillamat(5), Miriam Guitart(4) and Xavier Estivill(1,3,7)
(1) Genes and Disease Program, (2) CeGen and (3) CIBERESP, Center for Genomic Regulation (CRG-UPF); (4) Corporació Sanitària Parc Taulí; (5) Consorci Sanitari de Terrassa; (6) Hospital Universitari Institut Pere Mata, Unitat de Psiquiatria, Universitat Rovira i Virgili, Reus; (7) Pompeu Fabra University, Barcelona, Catalonia

Schizophrenia is a neurodevelopmental disorder characterized by disturbances in nearly every function of the brain: cognition, perception, affect and thought. Disturbances of neuron migration, alteration in neural plasticity and changes in synaptic connection are important factors in the pathogenesis of schizophrenia. Neurotrophic factors are involved in cellular proliferation, migration, differentiation and survival of neurons in the CNS during embryogenesis, organogenesis and in adult life. Clinically variable symptoms of schizophrenia are rarely considered in the majority of genetic studies that implicitly assume a unitary view of the disorder. The Positive and Negative Syndrome Scale (PANSS) is one of the most common instruments used in assessment of symptoms in schizophrenia. PANSS has been used to detect different associations of symptoms to assess the variance in clinical features. We have studied the relationship between schizophrenia symptom dimensions and polymorphisms in two neurotrophic genes and their receptors. We applied factor analyses to PANSS scale of 354 schizophrenic patients and we identified five factors (Negative, Disorganised, Positive, Excited and Depressive-Anxiety). We have genotyped 121 SNPs in NTRK2, BDNF, NTRK3 and NTF-3 in 143 schizophrenic subjects. Significant differences were found for three SNPs located in NTRK2 in subjects with higher scores for items included in the 'excited' factor. We also found two significant SNPs in NTRK3 in subjects with higher scores for items included in the 'disorganised' factor. We conclude that factor analysis might contribute to reduce clinical heterogeneity and increase the power of genetic studies in schizophrenia.

Supported by ISCIII, Fundació Parc Taulí and Genoma España.

Brunson, Tiffany

P47phox Genetic Polymorphisms in Cardiovascular Disease

TIFFANY A. BRUNSON; Dr. Qing Song
Morehouse School of Medicine, Atlanta, GA

P47phox (also called Neutrophil Cytosolic Factor 1, NCF1) is a component of NAD(P)H oxidase, which plays an essential role in the regulation of NAD(P)H oxidase activity, superoxide (O_2^-) production, and homeostasis of reactive oxygen species (ROS). It is known that ROS are involved in cellular signaling, normal vascular physiology, and contribute substantially to the development of cardiovascular disease. The redox state is controlled by a balance between enzymes that generate ROS such as NAD(P)H oxidases and intrinsic anti-oxidant enzymes such as superoxide dismutase. If this balance is perturbed in pathological conditions, it may play an important mediator role in the pathogenesis of atherosclerosis, hypertension and obesity/diabetes. A recent study of the Framingham cohort has documented that oxidative stress varies between individuals, and the level of systemic oxidative stress is positively associated with an increased risk for cardiovascular disease. It is postulated that the cellular factors that determine the pro-oxidant state also predispose to the progression of vascular disease and thereby increase the susceptibility to cardiovascular events.

There is compelling evidence that genetic factors play a significant role in the susceptibility to hypertension, hyperlipidemia, atherosclerosis, stroke and myocardial infarction. A recent family study indicated a strong heritability of plasma total antioxidant status (an indicator of redox homeostasis) in humans. It was estimated that approximately 51% of variance of redox homeostasis is due to additive effects of genes ($P<0.001$). This effect is especially strong among smokers in which heritability accounts for 83% of variance, whereas it accounts for 49% in nonsmokers. This finding is consistent with our working hypothesis that genetic polymorphisms may be a risk factor in determination of ROS homeostasis and therefore the susceptibility to vascular disease. The proposed study will begin to examine the role of the candidate gene p47phox as a key mediator of ROS homeostasis in humans.

The p47phox gene is expressed in both phagocytic and nonphagocytic cells, including lymphocytes, vascular smooth muscle cells (VSMC) and vascular endothelial cells. The p47phox expression can be controlled by vasoactive stimuli such as angiotensin II and the mechanical forces associated with hypertension, as well as growth factors or pro-inflammatory cytokines. NADPH oxidase generates O_2^- and H_2O_2. The biological effects of O_2^- and H_2O_2 on vascular function are critically dependent on their amounts within cells. Low intracellular amounts of ROS can act as second messengers to modulate the function of homeostatic biochemical pathways mediating various vascular responses; however, higher amounts of ROS can cause DNA damage, significant toxicity, or even apoptosis.

P47phox is the crucial regulator of NAD(P)H activity. We hypothesize that any genetic variants that influence the p47phox quantity may interrupt the ROS homeostasis and lead to predisposition to diseases. Since the induction of p47phox gene expression occurs at the transcriptional level, we further hypothesize that genetic polymorphisms in the 5' and 3' flanking regulatory regions of the p47phox gene may influence the transcription and protein production of this gene in vascular cells. Therefore, individuals with certain p47phox genotypes may exhibit basal increases in oxidative stress and evidence of vascular dysfunction. This study will provide important new insights into the molecular determinants of ROS homeostasis.

Bustamante, Mariona

Association Study Between 21 Polymorphisms And Bone Mineral Density In Spanish Postmenopausal Women: The Barcos Cohort

BUSTAMANTE M.(1), Àgueda L.(1), Jurado S.(2), Nogués X(2), Mellibovsky L(2), Díez-Pérez A(2), Balcells S(1), Grinberg D(1).
(1) Department of Genetics, Universitat de Barcelona, IBUB, CIBERER, Barcelona, Spain; (2) Internal Medicine, URFOA, IMIM, Hospital del Mar, Universitat Autònoma de Barcelona, Barcelona, Spain

Osteoporosis is a multifactorial disease, with a strong genetic component. Several genes have been reported to be involved in this pathology but there are controversial results and new candidates should be evaluated. In this study, the effect of polymorphisms in classical osteoporosis genes (VDR, ESR1, COL1A1 and TGFB1) as well as in less studied genes (RUNX2 and IL6R) have been evaluated in relation to lumbar spine (LS) and femoral neck (FN) bone mineral density (BMD) in the BARCOS cohort of Spanish postmenopausal women. This cohort cohort participates in the GENOMOS project, a multicentre consortium created to study the genetic bases of osteoporosis through prospective meta-analyses.

A total of 21 polymorphic variants were genotyped by SNaPshot, sequencing, RFLP or Genescan methodologies in a cohort varying from 556 to 821 samples. Statistic methods included chi-square tests, analysis of variance and covariance. The minor allele frequencies (MAFs) obtained ranged from 0.03 to 0.47, and were similar to the ones described previously in Caucasian populations.

Regarding classical osteoporosis genes, it was observed that the PvuII (ESR1) and the Leu10Pro (TGFB1) polymorphisms were associated with FN and LS BMD, respectively. However, the association between Leu10Pro and BMD disappeared when the genotypes of the cohort were enlarged with 200 new samples. The polymorphism -1997 G/T (COL1A1) and its interaction with the -1663 indelT (COL1A1) or with the Sp1 (COL1A1), which are in strong linkage disequilibrium (LD), were found to be associated with LS BMD. Finally, none of the polymorphisms in the VDR gene was associated with BMD. The effects on BMD and fracture risk of the polymorphisms located in classical osteoporosis genes were also studied in the GENOMOS meta-analyses. The results obtained in the GENOMOS project, that included approximately 26,000 samples, showed that none of the polymorphisms analyzed was associated with BMD, except for the SNP Sp1 (COL1A1). In the GENOMOS meta-analyses, the polymorphism Sp1 (COL1A1), Cdx2 (VDR) and XbaI (ESR1) were also associated with the risk of fracture, a phenotype not analyzed in the BARCOS cohort. In summary, the polymorphisms found to be associated with BMD in the BARCOS cohort or in the GENOMOS studies are not exactly the same. This could be due to various factors such as lack of statistical power in the BARCOS cohort or heterogeneity caused by unknown genetic and/or environmental factors, which could modify the association in a population specific manner.

Polymorphisms in less studied classical osteoporosis genes were also analyzed in the BARCOS cohort. In particular, two polymorphisms located each in one of the two RUNX2 promoters, and three SNPs located in the IL6R gene were genotyped. While the -1025 T/C polymorphism (promoter 2 of RUNX2 gene) was associated with FN BMD, the -330 G/T (promoter 1 of RUNX2) was not. On the other hand, the two SNPs analyzed in the IL6R promoter region were associated with FN BMD and with body mass index (BMI). Additionally, a non-synonymous SNP situated in exon 9 of the IL6R gene was associated with LS BMD. To confirm these associations between IL6R and RUNX2 polymorphisms and BMD, the studies should be replicated in meta-analyses similar to the ones undertaken by the GENOMOS consortium.

Buzdin, Anton

New Efficient Technique For Genome-Wide Monitoring Of Transcriptional Activity Of Repetitive Elements And Its Application To The Recovery Of Promoter-Active Human Specific Endogenous Retroviruses

ANTON BUZDIN, Elena Gogvadze, Elena Kovalskaya & Eugene D. Sverdlov
Shemyakin-Ovchinnikov Institute of Bioorganic Chemistry, 16/10 Miklukho-Maklaya, Moscow 117997, Russia.

To investigate the promoter activity of human-specific endogenous retroviruses, we developed a technique called GREM (Genomic Repeat Expression Monitor) that can be applied to genome-wide isolation and quantitative analysis of any kind of transcriptionally active repetitive elements. Briefly, the technique includes three major stages: (i) generation of a transcriptome wide library of cDNA 5' terminal fragments, (ii) selective amplification of repeat flanking genomic loci, and (iii) hybridization of the cDNA library (i) to the amplicon (ii) with subsequent selective amplification and cloning of the cDNA-genome hybrids. The sequences obtained serve as "tags" for promoter active repetitive elements. The advantage of GREM is an unambiguous mapping of individual promoter active repeats at a genome wide level. The technique provides instrument for both qualitative and quantitative experimental analyses. We applied GREM for genome-wide experimental identification of human specific endogenous retroviruses and their solitary long terminal repeats (LTRs) acting *in vivo* as promoters. The GREM technique enabled to identify 76 new functional human promoters created by retroviral LTRs. At least 50% of human specific LTRs possessed promoter activity, and many of them were up- or down regulated in germ-line cancer tissue. Individual LTRs were expressed at markedly different levels ranging from ~0.001 to ~3% of the housekeeping beta-actin gene transcript level. We demonstrated that the main factors affecting the LTR promoter activity were the LTR type (5' proviral, 3' proviral or solitary), and LTR position with regard to genes. The averaged promoter strengths of solitary and 3' proviral LTRs were almost identical in both tissues, whereas 5' proviral LTRs displayed 2-5-fold greater promoter activities. The relative content of promoter-active LTRs in gene-rich regions was significantly higher than in gene-poor loci. This content was maximal in those regions where LTRs .overlapped. read-through transcripts. Although many promoter active LTRs were mapped near known genes, no clear-cut correlation was observed between transcriptional activities of genes and neighboring LTRs. Our data suggest also selective suppression of transcription for the LTRs located in gene introns.

References:

[1] Buzdin, A., E. Kovalskaya-Alexandrova, E. Gogvadze, and E. Sverdlov. 2006. At least 50% of human-specific HERV-K (HML-2) long terminal repeats serve *in vivo* as active promoters for host nonrepetitive DNA transcription. J Virol 80:10752-62.
[2] Buzdin, A., E. Kovalskaya-Alexandrova, E. Gogvadze, and E. Sverdlov. 2006. GREM, a technique for genome-wide isolation and quantitative analysis of promoter active repeats. Nucleic Acids Res 34:e67.

Cahan, Patrick

An Ultra-Dense CNV Map Of The 129X1/SvJ Inbred Mouse Genome

PATRICK CAHAN, Laura Godfrey, Peggy Eis, Todd Richmond, Rebecca Selzer, Michael Brent, Howard McLeod, Tim Ley, Tim Graubert
Departments of Internal Medicine and Genetics, Division of Oncology, Stem Cell Biology Section, Washington University, St. Louis, MO. NimbleGen Systems Inc., Madison, WI. University of North Carolina.

CNVs are defined as genomic sequences that differ in copy number relative to a consensus genome and span thousands to several million bases. Although recent studies have reported an abundance of CNVs in mammalian genomes, the size distribution of CNVs remains unknown due to the limited ability of existing assay platforms and analysis algorithms to resolve short CNVs (<20kb). We present an application of the Hidden Markov Model (HMM) decoding methodology to map CNVs in the 129X1/SvJ inbred mouse genome using both high-resolution (HR: >300,000 probes/array) and ultra-dense (UD: >3 million probes/array) array comparative genomic hybridization (aCGH) data. Our algorithm utilizes sequence divergence information to improve sensitivity and a scoring function in combination with data randomization to assess the significance of CNV calls. We assess the performance of the algorithm by independent validation of 10 CNVs and by comparison of HR to UD calls. The 129X1/SvJ UD map includes 142 CNVs, which is more than an order of magnitude greater than the number previously reported in this genome. We also apply the method to HR and UD human data and report 70 and 81 CNVs in two individuals, 39 of which are novel. Taken together, these results indicate that many CNVs remain to be discovered in mammalian genomes. The gene and microRNA density within CNVs is similar to randomly selected segments of the 129X1/SvJ genome with identical length distributions. The CNVs range in length from 950 to over 3 million bases and therefore this combination of technology and analysis is able to approach the lower end of the CNV length distribution.

Calafell, Francesc

Homogeneity Of LD Patterns Among Spanish Populations

F. CALAFELL(1,2), P. Garagnani(3), A. González-Neira(1,4), J. Bertranpetit(1,2)
(1) Unitat de Biologia Evolutiva, Departament de Ciències Experimentals i de la Salut, Universitat Pompeu Fabra, Barcelona, Catalonia, Spain; (2) CIBER Epidemiología y Salud Pública (CIBERESP), Spain; (3) Università di Bologna, Bologna, Italy; (4) Centro Nacional de Investigaciones Oncológicas, Madrid, Spain.

The population genetic characteristics of samples included in genetic association studies can have a significant impact on the outcome of such studies. On one hand, extracting cases and controls from different populations (either by ignorance of the underlying population structure or because of differences in the prevalence of the disease across populations) can lead to spurious associations; on the other hand, genetically isolated populations offer the promise of enhanced power for association detection because of increased linkage disequilibrium (LD) and reduced heterogeneity in the genetic basis of the disease. To the best of our knowledge, no European population has been shown with single nucleotide polymorphisms (SNPs) to show increased LD.

The Basques are culturally isolated populated, living across the western border between France and Spain and speaking a non-Indoeuropean language. They show outlier allele frequencies in the ABO, RH, and HLA loci. To test whether Basques are a genetic isolate with the features that would make them good candidates in genetic association studies, we genotyped 123 SNPs in a gene-free 1-Mb region in chromosome 22 in Basque samples from France and Spain, as well as in samples from northern and southern Spain, and in three Moroccan samples. Both Basque samples showed similar levels of heterozygosity to the other populations, and the decay of LD with physical distance was not different between Basques and non-Basques. Thus, Basques do not show the properties that are expected of genetic isolates. We also found that genetic differentiation in our sample set was low, with only 14 out of the 123 SNPs showing $F_{st} > 0.05$. The full data set did not present a clear substructure as detected with the STRUCTURE program; however, the 14 SNPs with the to Fst values differentiated Iberian from N African populations.

Carpenter, Danielle

Analysis Of RYR1 Haplotype Profile To Identify Patients At Risk Of Malignant Hyperthermia

CARPENTER DANIELLE(1), Rachel Robinson(1), Patrick Booms(2), David Iles(2), Jane Halsall(1), Derek Steele(3), Philip M Hopkins(1), Marie-Anne Shaw(2).
(1) MH Investigation Unit, Academic Unit Anaesthesia, St James University Hospital, Leeds LS9 7TF; (2) Institute of Integrative and Comparative Biology, Faculty of Biological Sciences, L C Miall Building, University of Leeds, Leeds LS2 9JT; (3) Institute of Membrane and Systems Biology, Faculty of Biological Sciences, L C Miall Building, University of Leeds, Leeds, LS2 9JT.

Malignant hyperthermia (MH) is a pharmacogenetic disorder of skeletal muscle that, at least in the great majority of cases (~70%), follows an autosomal dominant pattern of inheritance and is caused by mutations in the gene encoding the skeletal muscle ryanodine receptor, RYR1.

Laboratory confirmation of MH is through the invasive *in vitro* contracture test (IVCT), for which the European MH group has a well defined and standardised protocol. The phenotypes of MH susceptible (MHS) and MH normal (MHN) are assigned according to the European MH Group protocol (www.emhg.org).

This study represents a new approach to characterising patients at risk of MH through the use of a recently published method for identifying high-risk haplotypes in candidate genes. We aim to ascertain the RYR1 haplotype profile in UK patients at risk of MH in order to assess whether it is possible to predict a patient's susceptibility status on the basis of a particular RYR1 haplotypic background, based on the premise that patients at risk of MH share a common founder haplotype(s). We present analysis based upon the largest standardised database of MH patients worldwide. We used unphased RYR1 SNP data directly to (1) assess RYR1 haplotype frequency differences between susceptible cases (MHS) and control groups (MHN and Caucasian population controls) and (2) analyse population-based association via clustering of RYR1 haplotypes based on disease risk. Our results show a significant difference in frequency of RYR1 haplotypes between susceptible cases (MHS) and both normal samples ($p=0.01$) and Caucasian population controls ($p=0.01$). Furthermore we find strong evidence for haplotypic association with MH and identify a high-risk cluster of haplotypes. We illustrate that this high-risk haplotype is associated with the commonest known MH causative mutation p.G2434R/c.7300G>A. These results are encouraging for assessing at-risk patients and also demonstrate the applicability of this new and practical method for population based association analysis.

Carter, Christopher

Genetic Signalling Networks Used By Environmental Risk Factors In Polygenic Diseases.

C.J.CARTER
176 Downs Road, Hastings, East Sussex, TN34 2DZ, UK

Gene association studies in polygenic diseases are notoriously inconsistent. However, in Alzheimer's disease, Bipolar disorder, and schizophrenia, the trail left by such studies etches out clearly defined signalling networks relevant to the disease pathology (http://www.polygenicpathways.co.uk). The products of the susceptibility candidates are functionally related; form elements of signalling cascades, control each other.s transcription, or compose elements of well-defined multi-protein complexes. Genes associated with Alzheimer's disease, including cholesterol synthetic enzymes and transporters, lipoproteins and their receptors as well as APP, BACE and the presenilins, can be related to a cerebral cholesterol shuttle responsible for the delivery of cholesterol from glia to neurones. Many of these genes have also been implicated in atherosclerosis, and the deleterious effects of these common susceptibility candidates are likely to be influenced both by diet and by cholesterol-lowering regimes.

Nearly 25% of > 200 genes implicated in schizophrenia code for proteins belonging to the postsynaptic density, a structure containing diverse elements necessary to ensure the maintenance of NMDA-receptor mediated long-term potentiation and synaptic efficiency. Others code for proteins concerned with glutathione and quinone-related oxidative stress or oligodendrocyte viability. Each of these families is relevant to different subpathologies of schizophrenia. Many genes implicated in Bipolar disorder code for proteins comprising consecutive elements of the growth factor-stimulated phosphoinositide kinase/AKT signalling cascade.

Genes that are generally accepted as important influences in these conditions appear to occupy equally key positions in these signalling networks. For example, DISC1, a key influence in schizophrenia and Bipolar disorder, binds to at least 6 other proteins implicated in these disorders (CIT, DPYSL2, FEZ1, MLC1, NDE1, PDE4B) [1]. Almost 60 other proteins coded for by schizophrenia or bipolar disorder susceptibility genes can be closely linked to the inputs or ouputs of a DISC1 transduction hub. Other important genes code for polyvalent proteins able to influence several of the underlying sub-pathologies of the disease. Less well-supported genes may code for components of more isolated branches of these pathways.

Bipolar disorder and schizophrenia, share a number of susceptibility genes, many of which code for elements of growth factor signalling, or of oxidative or endoplasmic reticulum-related stress pathways. These converge on an eif2-alpha kinase-signalling network that controls protein synthesis via the translation initiation factor eif2B. This same network is engaged by the environmental risk factors commonly associated with these diseases (famine, stress and viral or bacterial infection), and would appear to play an important role in controlling oligodendrocyte function. Oligodendrocyte loss is a feature of both bipolar disorder and schizophrenia.

When candidate genes belong to the same signalling network, others coding for proteins in the same cascade influence the degree of genetic risk afforded by each candidate [2]. As environmental factors may well use the signalling cascades etched out by the susceptibility genes, these too are likely to influence the results of association studies. These principles may in part explain the heterogeneity in single-gene association studies. Identification of the polygenic signalling networks, and of the factors that appropriate them may be relevant to the understanding and treatment of many other diseases of multigenic origin.

1. Camargo,L.M. et al. Mol Psychiatry 12, 74-86 (2007).
2. Wu,X. et al. Am. J Hum. Genet. 78, 464-479 (2006).

Casals, Ferran

Worldwide Variation In Innate Immunity Genes

CASALS, FERRAN; Ferrer-Admetlla, Anna; Bosch, Elena; Sikora, Martin; Bertranpetit, Jaume
Unitat de Biologia Evolutiva, Departament de Ciències Experimentals i de la Salut, Universitat Pompeu Fabra, Barcelona, Spain

One of the objectives of evolutionary biology focuses on the detection and understanding of differences among populations. As time has gone by populations have been adapting to the environment surrounding them. Particular pathogens (viruses, bacteria, parasites&) might have affected their hosts genome. Selective pressures of pathogens leave footprints on the host genome that can be identified. In order to detect the footprint of selection mediated by pathogens, we have typed 100 SNPs in 14 different genes involved in the innate immune system, which have been grouped in 9 genome regions, in 39 populations representing worldwide variation. The goal of this study is to measure and compare the amount of genetic heterogeneity (among populations and among continents) in genes related to the innate immunity.

The innate immune system constitutes the first defence to infection by pathogenic organisms. The innate immunity is present in vertebrates and invertebrates, it is not specific since it confers protection to a wide range of pathogens, and does not require a previous exposition to the pathogen. We hypothesize that genes involved in the pathogen-host interaction could show signals of differential adaptation and thus, that they could show strong differences among human populations living in different places on earth.

We have tested for different signatures of natural selection, as high differentiation of allele frequencies among populations, an excess of minimum allele frequencies (MAF) or derived allele frequencies (DAF) extremely high or low, the existence of some population specific haplotypes, differences in the linkage disequilibrium pattern, or an unusual haplotype homozygosity extension. In spite of some particular genes showing moderately high values of population differentiation or other signals of departure of neutral evolution, when compared to other gene categories, innate immunity genes turn out to show low levels of population differentiation, and their variability structure seems to remain homogeneous among populations. This conservation is probably due to the non-specificity of the innate immune system and the importance of this system during the first months of life.

Chambers, Isfahan

SNPs In The Regulatory Regions Of The Human Notch 3 Gene And Their Possible Implications In Ischemic Stroke

ISFAHAN CHAMBERS, Qing Song
Morehouse School of Medicine, Atlanta GA

Notch-3 is a member of the Notch receptor family that is involved in cell fate determinations. It is postulated that pathological changes in vessel structure are induced in part by signaling pathways that govern cell growth, death, differentiation and matrix production. Within the spectrum of identified arteriopathies, CADASIL (cerebral autosomal dominant arteriopathy with subcortical infarcts and leukoencephalopathy), is a heritable syndrome of systemic small vessel disease predisposing to stroke and vascular dementia. Vessel changes are characterized by prominent and progressive degeneration and loss of vascular smooth muscle cells (VSMC). The etiological basis for the CADASIL syndrome is loss-of-function mutations occurred within the coding exons of the Notch-3 gene. These coding SNPs are rare in humans and cannot account for the majority of the stroke, but reveal clues about the biological significance of Notch 3 in its relation to the common form of stroke. So far, the non-coding regulatory regions for the Notch-3 gene expression have not been systematically studied using molecular genetics and epidemiology.

Our preliminary studies have demonstrated that the level of Notch-3 gene expression may play an important role in arteriopathies. Our results showed that neointima formation in the injured vessels of carotid balloon injury rat model is associated with a significant increase of Notch-3 expression. Furthermore, the upregulation of Notch-3 expression promoted VSMC growth and inhibited VSMC apoptosis. We postulate that the Notch-3 gene expression may control the Notch-3 signaling in the vasculature. We further postulate that the genetic polymorphisms within the non-coding regulatory regions of the human Notch-3 gene may impair the regulation of Notch-3 gene expression. These non-coding regulatory genetic variants may be not as penetrant as those rare mutations occurred in the coding exons, but they may still play an important role in Notch-3 signaling and ischemic stroke.

Chan, Tingfung

Understanding Disease Causing Collagen Mutations In The Context Of Natural Variations

TINGFUNG CHAN (1); Annie Poon (1); Analabha Basu (1); Dale Bodian (4); Linda A. DiMeglio (2); Peter H. Byers (3); Teri E. Klein (4); Pui-Yan Kwok (1)
(1) Department of Dermatology, Cardiovascular Research Institute, and Institute for Human Genetics, University of California - San Francisco, San Francisco, CA; (2) Section of Pediatric Endocrinology and Diabetology, Riley Hospital for Children, Indiana University School of Medicine, Indianapolis, IN; (3) Departments of Pathology and Medicine (Medical Genetics), University of Washington, Seattle, WA; (4) Department of Genetics, School of Medicine, Stanford University, Stanford, CA

Collagens are members of one of the most important families of structural proteins in higher organisms. Collagen molecules are grouped into families based on their structural features. Types I, II, and III fibril collagens are the most abundant forms and account for 70% of the total body collagens. Mutations in the major fibrillar collagen genes lead to osteogenesis imperfecta (OI) (COL1A1 and COL1A2 that encode the chains of Type I collagen), chondrodysplasias (COL2A1 that encodes the chains of type II collagen), and vascular Ehlers-Danlos syndrome (COL3A1 that encodes the chains of type III collagen).

For the last two decades, mutations in these collagen genes have been catalogued to understand the molecular etiology of those diseases. The observation that some affected individuals with the same mutation (even within the same family) have different disease phenotypes is quite perplexing to geneticists. In order to characterize the genotype-phenotype relationships and build a robust model to predict the molecular and clinical outcomes of mutations, we undertook a study to define the natural variations in several populations.

We have screened exons, flanking intronic regions, and conserved non-coding regions for variations in the COL1A1, COL1A2, COL2A1 and COL3A1 in DNA from 48 individuals from each of four populations: African American, Mexican American, Chinese American, and European American. We also performed deep re-sequencing on COL1A1 and COL1A2 in 105 patients diagnosed with OI. We identified 459 single nucleotide polymorphisms (SNPs) in our healthy subjects, more than half of which were novel and not found in public databases. Of the 52 SNPs found in coding regions, 15 caused amino acid substitutions while 37 were synonymous. We found 53 missense mutations in COL1A1 and COL1A2 in the OI patients. By correlating the disease causing mutations in the context of background variations in the genes, we are able to explain differences in disease phenotype. Data from our study will also allow us to determine the sequence-structure-function relationships in collagen proteins.

This work is supported by NIAMS AR051582 (T. Klein, PI)

Charchar, Fadi

Copy Number Variation In The Spontaneously Hypertensive Rat

FADI J CHARCHAR and Nilesh J Samani
University of Leicester, Cardiology Group, Department of Cardiovascular Science, Glenfield Hospital, Leicester, UK

Introductions and Aims: The spontaneously hypertensive rat (SHR) is one of the most widely used genetic models for hypertension. The SHR model is characterized by hypertension, insulin resistance, hypertriglyceridaemia and hypercholesterolemia, yet, despite intensive searches for hypertension causative genes in this model very few specific annotated genes have been unambiguously identified. This may, in part, be due to the nature of mutations commonly searched for, mainly single base pair mutations. Copy number variation (CNV) is increasingly recognized as a source of inter-individual differences in genome sequence and has been proposed as a driving force for phenotypic variation. .The association of CNVs in hypertension has not been previously investigated in the SHR. We performed preliminary experiments to determine whether CNVs may play a role in the disease progression in the SHR.

Methods and Results: We performed a 2X2 comparative genomic hybridization (CGH) using a whole-genome array (Nimblegen Inc.) with a 5303bp median probe to detect alterations in DNA copy number between the SHR model and the non-hypertensive Wistar Kyoto (WKY) model from which the SHR was genetically derived. Briefly, SHR and WKY genomic DNA samples were extracted from spleen and randomly fragmented into lower molecular weight species. Genomic DNA (1 ¼g) was labeled with Cy3 (SHR) or Cy5 (WKY), and hybridizations were performed by NimbleGen Systems in a two-color format to a NimbleGen CGH array. Data were extracted and normalized using NimbleGen's DNACopy and SignalMap. A criteria of 5 or more probes in a segment, mean amplitude of log2 shift across segment = ±0.5 were used to define the final set of high confidence CNV calls. To validate CNVs detected by CGH, non-qPCR assays were used to measure copy number in altered regions relative to a control region of invariant copy number between the two strains. CNVs were detected on various rat autosomes and varied in size from 100 kb to 2 Mb. Interestingly, most of these variations were located in areas where previous QTLs for cardiovascular risk factors reside.

Conclusion: The identification and characterization of rat model CNVs are important because inbred strains of rats are the most widely used model system to explore hypertension genetics. Our preliminary CNVs seem to be located near or within blood pressure QTLs, more often than would be expected by chance, which supports the hypothesis that CNVs are causally linked. Importantly, many of the CNVs contain known genes and thus may underlie both gene expression and phenotypic variation between the rat models. Further studies and finer tiling arrays are warranted.

Colobran, Roger

Population Structure In Copy Number Variation (CNV) And Single-Nucleotide Polymorphisms (SNPs) In The CCL4L Gene

ROGER COLOBRAN(1), David Comas(2), Rosa Faner(1), Edurne Pedrosa(1), Roger Anglada(3), Ricardo Pujol-Borrell(1), Jaume Bertranpetit(2), Manel Juan(1).
(1) Laboratory of Immunobiology for Research and Application to Diagnosis (LIRAD), Tissue and Blood Bank (BST), Institut d Investigació en Ciències de la Salut Germans Trias i Pujol (IGTP), 08916 Badalona and Department of Cell Biology, Physiology and Immunology, Universitat Autònoma de Barcelona (UAB). 08193 Bellaterra, (Barcelona) Spain; (2) Unitat de Biologia Evolutiva, Departament de Ciències Experimetals i de la Salut, Universitat Pompeu Fabra, Barcelona, Spain; (3) Servei de Genòmica. Departament de Ciències Experimentals i de la Salut. Universitat Pompeu Fabra. Barcelona, Spain.

The recent description of a large amount of copy number variation (CNV) in the human genome has extended the concept of genome diversity. CNVs account for a large proportion of genetic variation, influencing gene expression and disease susceptibility. In this study we integrate the analysis of CNV and SNPs, combining the assessment of gene copy number with the genotyping of relevant SNPs in human CCL4L gene. CCL4L is a non-allelic copy of CCL4/MIP-1beta chemokine and displays a CNV that includes also the CCL3L gene, a non-allelic copy of CCL3/MIP-1alpha. This CNV and two functionally relevant CCL4L SNPs (rs4796195 and rs3744595) have been recently associated to HIV pathology in three independent studies. We have quantified the CCL4L copy number and genotyped both SNPs in samples from HGDP-CEPH Diversity Panel, ascribing the exact number of CCL4L copies to each allelic variant in heterozygous samples with more than two CCL4L copies. For the first time, we report worldwide population data combining both types of variation, CNV and SNPs, and our results show a clear population differentiation which is three times greater in the CNV than in the SNPs in CCL4L. Sub Saharan African populations display the highest number of CCL4L copies (mean > 4, including individuals with 8, 9 or 10 copies), whereas European populations present the lowest copy number (mean < 2, including a 25% of individuals with only one CCL4L copy). A strong correlation between CCL4L CNV and one of the SNPs analysed is found, whereas no linkage disequilibrium is found between the two SNPs despite their close distance (647 bp), suggesting a recent appearance of the second SNP when the diversity in the first one and CNV had already been generated. The present results suggest strong stratification in the susceptibility to diseases related to CCL4L variation. The present study points out that, in genes with CNV, it may be a key issue to combine the assessment of gene copy number with the genotyping of relevant SNPs to understand the phenotypic impact of genome variation in the immune response.

d'Adamo, Pio

Genetic Basis Of Urinary Absorption Of Amino Acids

P. D'ADAMO(1), S. Ulivi(1), L.Esposito(1), A.D'Eustacchio(1), P.Gasparini(2)
(1) IRCCS Burolo-Garofolo, Trieste
(2) Universita' degli studi di Trieste, Trieste

Complex diseases are difficult to analyze because there is a plethora of genes that underlays the disease and a variable environmental contribution.
One of the proposed methods to reduce complexities is to study complex traits in isolated populations.
Aminoacidurias are a group of complex diseases caused by defective epithelial amino acid transport systems.
Up to date, five aminoacidurias have been described in patients: cystinuria, lysinuric protein intolerance (LPI), Hartnup disorder, iminoglycinuria, and dicarboxylic aminoaciduria.
The genes responsible for the first three aminoacidurias have been identified in humans, whereas dicarboxylic aminoaciduria has been observed only in a KO mouse model of Slc1a5 and no single gene has been linked to iminoglycinuria yet.
For cistinuria, the most studied aminoaciduria, higher the levels of cystine in the urine, higher the probability of stone formation, which leads to obstruction, infections, and ultimately renal insufficiency.
People with the same mutation in genes involved in cystinuria however, can show different level of cystine excretion, and people with the same levels of cystine can have very different predisposition in stone formation in the urinary tract.
So there must be modulator genes and genes (or others factors) that predispose to calculi formation.
The scenario of amino acids in urine is complicated also by the fact that for some of them, there are no specific known kidney transporters.
Here we describe a study of genes potentially involved in urinary excretion of amino acids in order to find modulator genes and new amino acids transporters.
This study has been conducted in Carlantino and Stoccareddo, two isolated villages in the South and North of Italy respectively, previously selected and studied because of their historical, geographical and genetic evidence on the homogeneity of their populations due to the isolation.
We have chosen two populations with different genetic background in order to find association with polymorphism that have a very strong impact on phenotypes.
In Carlantino we have collected 479 urinary samples, and 184 in Stoccareddo and analyzed all with NMR.
We have detected levels for 17 amino acids, 8 organic acids, 3 keton bodies and 7 N-methyls. All the data were subsequently normalized on creatinin levels.
This is the first time in which a high number of amino acids and urinary metabolites were measured in a non-selected population. We first looked for a correlation between levels of different amino acids in order to find possible common transporters.
We found some correlations, the stronger of which is between Leucine and Valine.
For the genetic side, we chose 10 genes, with known or putative roles in amino acids reabsorption in kidney. We also selected 100 tag SNPs in the region on these genes (from 10kb before the gene to 10kb after the genes), according with HapMap, and genotyped all the samples using Taqman methodology. All the data were analyzed using ANOVA and Kruskal-Wallis and when possible, with QTDT test.
We found expected associations (like Lysine and SLC7A7, Phenylalanine and SLC6A18 that is a transporter of neutral amino acids, etc.) and some unexpected that we are currently evaluating.
For example, we founded a strong association between Alanine and SLC7A9. This association can indicate that SLC7A9 can play a role in the exchange with dibasic amino acids or can be an indirect, functional interaction with SLC7A8.
We are currently performing a genome wide search using the ILLUMINA Linkage Panel with more than 6000 tag SNPs in order to find new amino acids transporters and modulator genes for known transporters, and to elucidate the roles of the different genes in urinary absorption of amino acids.

D'amato, Mauro

The Neuropeptide S Receptor (NPSR1) Gene Is Associated With Susceptibility To Inflammatory Bowel Disease

MAURO D'AMATO, Sara Bruce, Francesca Bresso, Marco Zucchelli, Sini Ezer, Ville Pullkinen, Cecilia Lindgren, Marco Astegiano, Mario Rizzetto, Paolo Gionchetti, Gabriele Riegler, Raffaello Sostegni, Marco Daperno, Sandra D Alfonso, Patricia Momigliano-Richiardi, Leif Torkvist, Pauli Puolakkainen, Maarit Lappalainen, Paulina Paavola-Sakki, Leena Halme, Martti Farkkila, Ulla Turunen, Kimmo Kontula, Robert Lofberg, Sven Pettersson and Juha Kere
Department of Biosciences and Nutrition, Karolinska Institutet, Stockholm, Sweden

The neuropeptide S receptor (NPSR1) gene has been previosuly associated to asthma, and maps in a region of chromosome 7 previously linked also to inflammatory bowel disease (IBD). We genotyped NPSR1 polymorphisms in 2490 IBD patients and controls from Italy, Sweden and Finland, and analyzed the expression of its 2 major isoforms (NPSR1-A and NPSR1-B) in the intestine of IBD patients and healthy individuals. Global analysis of the whole dataset identified strong association of a NPSR1 haplotype block with IBD (P = 0.0018), and its two major forms Crohn s disease (CD) (P = 0.026) and ulcerative colitis (UC) (P = 0.003). Predisposing and protective haplotypes were also identified, with specific effects on both CD (P = 0.0005 for haplotype H2) and UC (P = 0.003 for haplotype H8), respectively. The expression of NPSR1 was increased in IBD patients compared to controls, both at the protein and RNA level. Moreover, NPSR1 risk haplotypes correlated with higher expression of both NPSR1-A (P = 0.024) and NPSR1-B (P = 0.047) mRNAs. Thus, specific NPSR1 alleles might act as common genetic risk factors for chronic inflammatory diseases of the epithelial barrier organs, such as asthma and IBD.

Dapprich, Johannes

Automated Separation Of Genomic Duplications

Ferriola, Deborah; Kunkel, Mark; DAPPRICH, JOHANNES
Generation Biotech, Lawrenceville, NJ

Genetic duplications are a common factor in the human genome that frequently complicate accurate molecular analysis, with few direct solutions available. Copy number variants and structural variation can have significant influence on the accuracy of SNP typing, sequencing and haplotype analysis, and the interpretation of typing results can greatly be affected by the underlying genomic context. Current methods for characterizing individual samples for a range of genomic variation tend to be costly and inefficient, especially for large candidate regions of interest. The assembly of multicopy or rearranged regions is considerably more time consuming, difficult or even impossible for complex genomes if read lengths are short. This is the case for next-generation sequencing approaches, which typically have ten-fold shorter read lengths (~60 bases) compared to conventional Sanger sequencing techniques. Therefore generic, high-throughput methods to establish the large-scale genomic context of short sequence reads are needed.

Region-specific extraction (RSE) is an automated method that reduces the complexity of a sample by physically isolating targeted genomic elements, including any flanking sequence of interest, from a sample of genomic DNA. An enzymatic step incorporates biotin labels only for probes targeting specific sequences. This results in single-base specificity coupled with high capture efficiency, including for large genomic regions. Magnetic beads are then attached and isolate the targeted chromosomal fragments from a mixture of genomic DNA. The size of the captured fragments can be modified by the method used for initial DNA preparation, by optional shearing or enzymatic digestion and by the extraction method.

Here we demonstrate the selective separation and analysis of a highly homologous, duplicated and copy number variable gene region called MICA/MICB, located in the major histocompatibility complex (MHC) region of chromosome 6. This region is implicated in numerous autoimmune and other diseases such as diabetes and is routinely typed for transplantation tissue matching. RSE probes were used to selectively extract the duplication containing the MICA gene from the duplication containing the MICB gene using SNPs that differentiate the two copies. The extractions were then used directly in standard downstream assays, thereby resolving a common and persistent obstacle to successful sequencing of this locus. A similar approach was used to separate based on tagSNPs, resolve homologous gene cassettes in the killer immunoglobulin-like receptor (KIR) region on chromosome 19 and map the location and copy number of mobile genomic elements in yeast on DNA microarrays.

RSE is directly compatible with essentially any typing method and can be carried out in multiplexed (multiple targeted sites per extraction) and 96-well parallel format on commercially available systems (BioSprint96, EZ1, Biorobot M48, Tecan Genesis). This provides a sample preparation tool that can deconvolute complex genomic regions in a high-throughput mode by combining the flexibility of current genome analysis methods with the more informative content that is typically achieved only by more laborious screening methods.

Darvasi, Ariel

Type 2 Diabetes Whole Genome Association Study In Four Populations: The DiaGen Consortium

Jukka T Salonen, Pekka Uimari, Juha-Matti Aalto, Mia Pirskanen, Jari Kaikkonen, Boryana Todorova, Jelena Hyppönen, Veli-Pekka Korhonen, Janne Asikainen, Christopher Devine, Tomi-Pekka Tuomainen, Kristiina Nyyssönen, Jan Luedemann, Matthias Nauck, Wolfgang Kerner, Richard H. Stephens, John P New, William E Ollier, J Martin Gibson, Antony Payton, Michael A Horan, Neil Pendleton, Walt Mahoney, David Meyre, Jerôme Delplanque, Philippe Froguel, Oren Luzzatto, Benjamin Yakir, ARIEL DARVASI
Oy Jurilab Ltd, Microkatu 1, 70210 Kuopio, Finland,
Research Institute of Public Health and School of Public Health and Clinical Nutrition, University of Kuopio, P.O. Box 1627, 70211 Kuopio, Finland
Institute of Clinical Chemistry and Laboratory Medicine, Ernst Moritz Arndt University Greifswald, Ferdinand-Sauerbruch-Str., 17487 Greifswald, Germany
Center of Cardiology and Diabetes, Greifswalder Str. 11, 17495 Karlsburg, Germany
Vascular Research Group, University of Manchester, Clinical Sciences Building, Hope Hospital, Salford, UK M6 8HD
Centre for Integrated Genomic Medical Research, University of Manchester, Stopford Building, Oxford Road, Manchester UK M13 9PT
Division of Medicine and Neurosciences-Hope, University of Manchester, Hope hospital, Salford, UK M6 8HD
Nanogen Inc., 10398 Pacific Center Ct., San Diego, CA 92121, U.S.A.
Unité mixte de Recherche CNRS, 8090, Institut Pasteur de Lille, 1 rue Calmette, 59000 Lille, France
Genomic Medicine, Hammersmith Hospital, Imperial College London, UK
Dept. of Genetics, Institute of Life Sciences, The Hebrew University of Jerusalem, Jerusalem 91904, Israel
Dept. of Statistics, The Hebrew University of Jerusalem, Jerusalem 91904, Israel

Type 2 diabetes (T2D) is a common, polygenic chronic disease with high heritability. The purpose of this whole-genome association (WGA) study was to discover novel T2D-associated genes. We genotyped 500 familial cases and 497 controls with over 300,000 HapMap derived tagging single nucleotide polymorphism markers (SNPs). Following a stringent statistical correction for multiple testing, the only significant SNP was at the TCF7L2 gene, which has already been discovered and confirmed as a T2D susceptibility gene. For a replication study, we selected ten SNPs in six chromosomal regions with the strongest association (singly or as part of a haplotype) to retest in an independent case-control set including 2573 T2D cases and 2776 controls. The most significant replicated result was found at the AHI1-LOC441171 gene region.

De, Subhajyoti

Landscape Of Changing Constraints On Protein Coding Genes In The Evolution Of Homo Sapiens

SUBHAJYOTI DE (1), Sarah A. Teichmann (1) & Nuria Lopez-Bigas (2)
(1) MRC Laboratory of Molecular Biology, Hills Rd, Cambridge CB2 2QH, UK; (2) Research Unit on Biomedical Informatics, Universitat Pompeu Fabra, Dr. Aiguader 88, 08003 Barcelona, Spain

Accumulation of mutations followed by selection leads to evolution of new species. Protein-coding regions in a genome evolve primarily by sequence divergence of individual genes, and gene gain and loss, altering the gene content of the organism. However, it is not well understood how these processes have resulted in the enormous diversity of metazoa present today. Have metazoans evolved through a process of incremental changes occurring evenly across genes of all different functions, or is there a dependence on function and phylogenetic distance? We quantify the divergence of human proteins at different levels, from diversity at nucleotide positions in human populations to protein conservation from mammals to fungi. This reveals that proteins involved in core processes such as metabolism are highly conserved across all eukaryotes, while regulatory proteins such as transcription factors, signal transducers and receptors are divergent. Previous studies considered individual aspects of genome evolution in detail, and many results are suggestive of this general trend. Our comprehensive analysis puts these results into the larger context of the evolution of the human genome and its selective constraints. We find that transcription factors have been subject to recent evolution specifically human lineage, while adaptive processes such as immunity evolve rapidly in all mammals as well as in humans. This global overview shows that morphological changes in metazoa have been driven by variation in regulatory rather than enzymatic and structural genes, supporting previously postulated theories of robustness and evolvability.

Reference:
Subhajyoti De, Sarah A. Teichmann & Nuria Lopez-Bigas. Evolvability and protein divergence in the evolution of Homo sapiens. (submitted to Nature Reviews Genetics as analysis article).

Dear, Paul

Single-Molecule Approaches To Genomic Variation: Tools For The Analysis Of Copy-Number, Structural Rearrangements And Haplotypes.

Hanif Ali (1), Alan T. Bankier (2), Angelika Daser (3), Paul Edwards (4), Bernard A. Konfortov (2), Frank. McCaughan (5), Jessica Pole (4), Pamela Rabbitts (1), Terry Rabbitts (1), Madan Thangavelu (4), and PAUL H. DEAR (2)
(1) Leeds Institute of Molecular Medicine, Leeds, England; (2) MRC Laboratory of Molecular Biology, Cambridge, England; (3) Institut fuer Humangenetik, Mainz, Germany; (4) Hutchison/MRC Research Centre, Cambridge, England; (5) Centre for Respiratory Research, UCL, London, England.

Methods based on the analysis of single DNA molecules make it possible to examine genomic variation in ways not possible by conventional, bulk-DNA approaches. For example, using single-molecule approaches:

*Copy-number can be assessed more robustly and directly by counting molecules than by measuring hybridisation signals or qPCR response curves.
*Copy-number can be measured reliably in very small numbers of cells, such as subpopulations recovered by laser-capture microdissection from fixed material
*Genomic rearrangements in individuals or in specific tissues can be analysed by local mapping
*Intra- as well as inter-cellular variation can be addressed: complex multi-locus haplotypes can be resolved, without the need for pedigrees.

At the meeting, we will present examples of the use of these single-molecule methods for addressing variation at the organism, tissue, cellular and subcellular levels. We will also discuss some technical complexities which all such approaches need to take into consideration.

De La Vega, Francisco

Next Generation Sequencing - Opportunities And Constraints In Pooling And Quantitative Sequencing

FRANCISCO M. DE LA VEGA(1), Fiona C.L. Hyland(1), Jon Sorenson(1), Edward Cuppen(3), Kevin McKernan(2).
Applied Biosystems, (1)Foster City, CA, and (2)Beverly, MA, USA, and (3)Hubretch Lab, Utrecht, The Netherlands.

Next generation, rapid, low-cost genome sequencing promises to address a broad range of genetic analysis applications. Some of these involve quantitative sequencing of mixtures, for example, to identify somatic mutation profiles in cancer, to investigate allele-specific expression, or to detect rare mutations or differences in allele frequencies in pooled samples of cases and controls to elucidate the genetic basis of complex disease. We have developed a model to simulate digital sequencing with pooled samples, in the presence of error. We estimate the number of reads (coverage) that will be necessary to discover rare variants. We discover that for pooling and quantitative sequencing, the number of samples that can be pooled and the minor allele frequency of variants that can be detected is critically dependant on the threshold for SNP calling, which in turn is strongly influenced by the measurement error rate. The higher the error rate, the fewer samples can be pooled for detection of rare variants. As next generation sequencing platforms typically produce short reads (25-35bp), coverage needs to increase over 20-fold to compensate. Nevertheless, beyond a certain point, increasing the coverage improves the estimate of the error rate, but cannot overcome problems with detecting very low frequency variants with large numbers of pooled samples, in the presence of error. We validated this model through empirical sequencing by oligonucleotide ligation and detection (Applied Biosystems SOLiD(tm) system) of 81 PCR amplicons from exons of EMS-mutagenized C. elegans worms encompassing ~25kb of sequence with over 1500x coverage. Amplicons were pooled at different dilutions down to a 1:100 ratio (1:200 ratio for alleles as all mutants are heterozygous). The results were compared with di-deoxy sequencing data carried out independently for each amplicon. Our results suggest that even if coverage needs to increase significantly when using short reads as compared with di-deoxy sequencing, low platform error rate is the most critical factor for detecting allele variants in pooled samples or mixtures by next generation sequencing platforms. Furthermore, variance introduced by measurement or experimental procedures reduced the ability to detect rare variants, which suggest that simple sample preparation methods are desirable.

Deyneko, Igor

Compensatory Mutations In Promoters: Human And Chimpanzee Are Closer Than Thought Before

DEYNEKO I(1,2); Kalybaeva Y(1); Kel A(3); Blöcker H(1) and Kauer G(4)
(1) Department of Genome Analysis, Helmholtz Centre for Infection Research, Braunschweig, Germany; (2) Institute of Cytology and Genetics SB RAS, Novosibirsk, Russia; (3) BIOBASE GmbH, Wolfenbüttel, Germany; (4) University of Applied Sciences, Emden, Germany

We performed a comparative analysis of promoters of orthologous genes of human and chimpanzee located on chromosomes 21. Similarities between DNA sequences were calculated as similarity of their 'melting enthalpy' characteristics (using FeatureScan [1]) and identity of nucleotide sequences (using ClustalW).

Close investigation of promoters of chimpanzee and human genes, which differ by less than 2% of nucleotides, showed that the similarities of their melting characteristics are significantly higher than it can be expected (under pure random and transition/transversion biased models). We found that 139 out of 198 orthologous promoter pairs showed higher signal similarity than can be expected - with a p-value of 2,43e-8.

Using the EMBL-EBI gene ontology classification, we examined the distribution of genes, which showed high melting similarity of their promoters. A subset of 15 genes involved in the molecular function 'metal ion binding' and another subset of 11 genes involved in 'nucleotide binding' were identified, with p-values of 4,9e-3 and 4,52e-5, respectively. Contrarily, applying ClustalW, no significant association with GO terms was found.

A strong correlation of signal similarity with gene expression was found by comparing our present results with our earlier data [2]. The prediction accuracy of gene expression by comparing melting characteristics of their promoters reaches a sensitivity of 83% and a selectivity of 60% using FeatureScan. At the same time, using bare letter conservation (ClustalW) under the same conditions provides only 30% and 40%, respectively.

As we may speculate from the presented results, a nucleotide substitution having occurred during the course of evolution, which for example, lowered down melting temperature of a locus, may have induced an evolutionary pressure on further changes to elevate it back. As further investigations and developing the proposed methodology [3], it will be interesting to probe changes of characteristics (melting temperature, conformation and others) caused by SNP mutations and their correlations with phenotypes or diseases.

1 Deyneko IV. et.al., FeatureScan: revealing property-dependent similarity of nucleotide sequences. Nucleic Acids Research, 2006, 34, W591-W595. http://genome.helmholtz-hzi.de/featurescan/
2 Watanabe, H. et al., DNA sequence and comparative analysis of chimpanzee chromosome 22. Nature, 2004, 429, 382-388.
3 Kauer G. et.al., Applying signal theory to the analysis of biomolecules. Bioinformatics 2003, 19, 2016-2021.

Dopazo, Hernan

Evolutionary Strength At The Codon Level Improves The Prediction Of Disease Related Protein Mutations In Human

CAPRIOTTI, E.(1), L. ARBIZA(1), R. CASADIO(2), J. DOPAZO(1), H. DOPAZO and M. MARTI-RENOM(1)
(1) Bioinformatics Department, Centro de Investigación Príncipe Felipe (CIPF), Valencia, Spain; (2) Laboratory of Biocomputing. CIRB/Department of Biology. University of Bologna, Bologna, Italy.

Predicting the functional impact of a protein variation is one of the most challenging problems in Bioinformatics. A rapidly growing number of genome-scale studies provide large amounts of experimental data allowing the application of rigorous statistical approaches for predicting if a given single point mutation has or not an impact on human health. Up until now, existing methods have limited their source data to either protein or gene information. Novel in this work, we take advantage of both and focus on protein evolutionary information by using estimated selective pressures at the codon level.

Here we introduce a new method (SeqProfCod) to predict the likeliness that a given protein variant is associated or not with a human disease. We have developed a Support Vector Machine classifier trained using three sources of information: protein sequence, multiple protein sequence alignments and the estimation of selective pressure at the codon level. SeqProfCod has been benchmarked with a large dataset of 9,979 single point mutations from 1,599 human proteins from SWISS-PROT. It achieves 77% overall accuracy and a correlation coefficient of 0.49 indicating that the estimation of the selective pressure helps in predicting the functional impact of single-point mutations. Thus, this study demonstrates the synergic effect of combining the two classical sources of information for predicting the functional effects of protein variants: protein sequence/profile-based information and selective pressures at codon level.

The results of large-scale application of SeqProfCod over all annotated point mutations in SWISS-PROT are available for download at http://bioinfo.cipf.es/sgu/services/SeqProfCod/.

Eis, Peg

Investigation Of DNA Copy Number Variants In The Human Genome Using 2.1M Feature Long Oligonucleotide Microarrays.

EIS, PEGGY; Selzer, Rebecca; Richmond, Todd; Green, Roland
NimbleGen Systems Inc., Madison, WI 53711 USA

Microarray-based comparative genomic hybridization (array CGH) methods have been widely used to investigate chromosomal abnormalities associated with cancer and developmental disorders on a genome-wide level. Historically, assessment of copy number changes in tumor genomes were performed with BAC array CGH, which is limited to 0.1-1 Mb resolution. Recently, oligonucleotide-based approaches have been adopted, providing an opportunity for orders of magnitude increase in resolution since they are smaller in size (< 100 bp) as compared to the average BAC clone (100,000 bp). In fact, the size range of copy number variants (CNVs) in the human genome is already known to occur down to 1 Kb in the HapMap samples, and this range is expected to extend further into the indel range of 2-1,000 bp. Thus, it is critical to develop higher resolution copy number analysis platforms to investigate the full spectrum of genome-wide variation among the population.

We have developed the first array CGH platform containing 2.1 million long oligonucleotide probes per array, offering a probe every 1 Kb across the entire human genome in an unbiased tiling-path design format. The 2.1M-feature array enables detection of deletions and amplifications down to ~5 Kb in size, as well as breakpoint mapping of larger sized chromosomal changes at the gene level. In some cases, CNVs previously characterized on lower resolution platforms, show complex structural variation when examined with higher probe density (e.g., a deletion immediately adjacent to an amplification). In addition to whole-genome arrays, we have developed higher throughput multiplex formats of both 390K- and 2.1M-feature microarrays (e.g., 4 sub-arrays of 390K providing 70K probes per sample) to fine-map single genes or regions, or multiple loci of interest in the genome. These new microarray technology improvements will enable rapid progress in defining the size range and frequency of copy number changes and genome variation and the role it plays in both normal biology and diseased states, and may accelerate the use of copy number changes in genome-wide association studies for complex diseases.

Fernandez, Israel

Genetic Background Predicts The Evolution Of Ischemic Stroke Patients Treated With t-PA

FERNANDEZ-CADENAS, ISRAEL; Del Rio, Alberto; Mendioroz, Maite; Domingues, Sophie; Ribó, Marc; A. Molina, Carlos; Cuadrado, Eloy; Corbeto, Natalia; Hernandez, Mar; Borrell, Maria; Ortega-Torres, Laura; Penalba, Anna; Navarro, Miriam; Garcia, Lidia; Arias Raquel; Rosell, Anna; Álvarez-Sabín, José; Montaner Joan.
Neurovascular Research Laboratory, Neurovascular Unit.
Department of Neurology of Vall d¿Hebron University Hospital.

Introduction: Ischemic stroke is one of the leading causes of mortality and the first cause of incapacity in the world; thombolytic therapy with t-PA is the only approved therapy (1). Despite its proven efficacy and benefits, 6 to 5% (2) of the patients suffer symptomatic hemorrhagic transformations with high death rates, and in up to 50% (3) of the cases arterial recanalization is not achieved early enough leading to poor outcomes. Our group identified baseline plasmatic levels of some molecules related to blood brain barrier disruption or fibrinolysis inhibition as predictors of hemorrhagic complications or resistance to recanalization (2,4). The appearance of hemorrhagic transformation has avoid the general application of this drug in all ischemic stroke patients, been only the 1% of ischemic stroke patients who can be treated with this drug. Determining the biological mechanisms that involve the toxic response and low efficacy of the fibrinolytic therapy could help to improve and generalize the administration of t-PA in ischemic stroke patients.

Objectives: To study whether individual genetic background predicts clinical evolution after t-PA treatment, and to discover new molecules associated with hemorrhagic transformation or artery recanalization, susceptible to be used as diagnostic and therapeutic targets to avoid hemorrhagic transformation and/or resistance to recanalization.

Methods: We analyzed the incidence of a total of 15 gene polymorphisms in 200 t-PA treated stroke patients with a documented occlusion in the middle cerebral artery. In order to determine polymorphisms we will use direct secuenciation, RFLP and allelic discrimination techniques. To study the functional implications of the most significant polymorphisms, we used ELISA, Von Claus, coagulation kits and nephelometry. Recanalization was diagnosed by means of Transcranial Doppler at 1 hour, 6 hour and 24 hour post-tPA, neurological worsening was measured by NIHSS score and hemorrhagic transformation appearance by computed tomography at 48 hours or earlier if a neurological worsening occurred.

Results: Polymorphisms of coagulation factors genes, fibrinolysis inhibition genes and vasoconstriction genes were associated with recanalization rates, hemorrhagic transformation appearance, neurological outcome and mortality after t-PA administration. Similarly, protein levels of inflammation markers were associated with hemorrhagic transformation appearance and mortality rates.

Conclusions: Genetic background predicts the risk and the efficacy of t-PA treatment in ischemic stroke patients. Pharmacogenetic analysis before t-PA administration could improve the benefit of the administration of t-PA and perhaps generalize the use of this treatment in ischemic stroke in the future.

1. The National Institutes of Neurological Disorders and Stroke rt-PA Stroke Study Group. Tissue plasminogen activator for acute ischemic stroke. N Engl J Med 1995; 333:1581-1587.
2. Montaner J, Molina CA, Monasterio J, Abilleira S, Arenillas JF, Ribo M, Quintana M, Alvarez-Sabin J. Matrix metalloproteinase-9 pretreatment level predicts intracranial hemorrhagic complications after thrombolysis in human stroke. Circulation. 2003 Feb 4;107(4):598-603.
3. Alexandrov AV, Demchuk AM, Felberg RA, Christou I, Barber PA, Burgin WS, Malkoff M, Wojner AW, Grotta JC. High rate of complete recanalization and dramatic clinical recovery during tPA infusion when continuous monitored with 2-MHz transcranial Doppler monitoring. Stroke 2000; 31:610¿614.
4. Ribo M, Montaner J, Molina CA, Arenillas JF, Santamarina E, Alvarez-Sabin J. Admission fibrinolytic profile predicts clot lysis resistance in stroke patients treated with tissue plasminogen activator.
Thromb Haemost. 2004 Jun;91(6):1146-51.

Fernando, Olga

Patterns And Rates Of Intron Divergence Between Humans And Chimpanzees: The Hallmark Of Selection

OLGA FERNANDO(1,3), Elodie Gazave(1), Tomàs Marqués-Bonet(1), Brian Charlesworth(2), Arcadi Navarro(4)
(1) Unitat de Biologia Evolutiva. Departament de Ciències Experimentals i de la Salut. Universitat Pompeu Fabra. Barcelona, Catalonia, Spain; (2) Institute of Evolutionary Biology. University of Edinburgh. King's Buildings. West Mains Road EH7 3JT. Edinburgh. Scotland. UK; (3) Instituto de Tecnologia Química e Biológica (ITQB), Universidade Nova de Lisboa, Portugal; (4) Institucio Catalana de Recerca i Estudis Avancats (ICREA) and Unitat de Biologia Evolutiva. Departament de Ciències Experimentals i de la Salut. Universitat Pompeu Fabra. Barcelona, Catalonia, Spain.

Introns, which constitute the largest fraction of eukaryotic genes and which had been considered to be neutral sequences, are increasingly acknowledged as having important functions. Several studies have investigated levels of evolutionary constraint along introns and across classes of introns of different length and location within genes. However, these studies, have so far yielded contradictory results. We present the first analysis of human-chimpanzee intron divergence, where differences in the number of substitutions per intronic site (Ki) can be interpreted as the footprint of different intensities and directions of the pressures of natural selection. We show that the higher divergence of first introns is related to their larger size. Also, the lower divergence of short introns suggests that they may harbor a relatively higher proportion of regulatory elements than long introns. Moreover, our results are consistent with the presence of functionally relevant sequences near the 5' and 3' ends of introns. Although located in non-coding sequences, functional elements should evolve under selection. The presence of many highly conserved sequences described in introns suggests the action of purifying selection on intronic sequences. In addition, we demonstrate that positive selection has shapped divergence patterns of intron sequences.

Gazave, Elodie

On The Association Between Chromosomal Rearrangements And Genic Evolution In Humans And Chimpanzees

ELODIE GAZAVE(1), Tomàs Marquès-Bonet(1), Jesús Sànchez-Ruiz(1), Lluís Armengol(2), Razi Khaja(3), Jaume Bertranpetit(1), Núria Lopez-Bigas(4), Mariano Rocchi(5), Arcadi Navarro(6)
(1) Unitat de Biologia Evolutiva Departament de Ciències Experimentals i de la Salut. Universitat Pompeu Fabra. Barcelona, Catalonia, Spain; (2) Genes and Disease Program, Center for Genomic Regulation, Barcelona Biomedical Research Park, Barcelona, Catalonia, Spain; (3) The Center for Applied Genomics, Toronto, Canada; (4) Research Unit on Biomedical Informatics of IMIM/UPF. Barcelona, Catalonia, Spain; (5) Dipartimento di Genetica e Microbiologia. Universita di Bari. Bari, Italy; (6) Institucio Catalana de Recerca i Estudis Avancats (ICREA) and Unitat de Biologia Evolutiva, Departament de Ciències Experimentals i de la Salut. Universitat Pompeu Fabra. Barcelona, Catalonia, Spain.

The role that chromosomal rearrangements might have played in the speciation processes that have separated the lineages of humans and chimpanzees has recently come into the spotlight. To date, however, results are contradictory. Here we revisit this issue by making use of the available human and chimpanzee genome sequence to study the relationship between chromosomal rearrangements and rates of DNA sequence evolution. Contrary to previous findings for this pair of species we show that genes located in the rearranged chromosomes that differentiate the genomes of humans and chimpanzees, especially genes within rearrangements themselves, present lower divergence than genes elsewhere in the genome. Still, there are considerable differences between individual chromosomes. Chromosome 4, in particular, presents higher divergence in genes located within its rearrangement.

A first conclusion of our analysis is that divergence is lower for genes located in rearranged chromosomes than for those in colinear chromosomes. We also report that non-coding regions within rearranged regions tend to have lower divergence than non-coding regions outside them. These results suggest an association between chromosomal rearrangements and lower non-coding divergence that has not been reported before. Some chromosomes do not follow this trend and could be associated with a speciation event but, as a whole, results also suggest that chromosomal speciation has not been common along the human and chimpanzee lineage.

Gijselinck, Ilse

Genomic Progranulin Deletion In Frontotemporal Dementia Patient

I. GIJSELINCK(1,3,5), S. Engelborghs(4,5,6), J. van der Zee(1,3,5), K. Peeters(1,3,5), M. Mattheijssens(1,3,5), E. Corsmit(1,3,5), D. Goossens(2,5), J. Del-Favero(2,5), P. P. De Deyn(4,5,6), C. Van Broeckhoven(1,3,5) and M. Cruts(1,3,5)
(1) Neurodegenerative Brain Diseases Group and (2) Applied Molecular Genomics Group, Department of Molecular Genetics, VIB; (3) Laboratory of Neurogenetics; (4) Laboratory of Neurochemistry and Behavior, Institute Born-Bunge; and (5) University of Antwerp, Universiteitsplein 1, BE-2610 Antwerpen, Belgium; (6) Memory Clinic, Division of Neurology, Middelheim General Hospital, Antwerpen, Belgium

Mutations in progranulin (PGRN) were recently identified in patients with ubiquitin-positive frontotemporal dementia linked to chromosome 17q21 (FTDU-17). These mutations resulted in loss of functional PGRN due to the creation of null alleles suggesting a haploinsufficiency mechanism. Therefore genomic deletions leading to a non-functional allele could be expected in FTD patients. A Belgian series of 103 pure FTD patients was systematically screened for such mutations by multiplex amplicon quantification (MAQ) and real time PCR allele quantification (qPCR). We identified one PGRN-containing genomic deletion in an FTD patient that was absent in 267 control individuals. Using a panel of semi-quantitative multiplex PCRs we finemapped the extent of the PGRN-containing deletion to a region of at least 54 kb and maximum 69 kb containing the complete PGRN gene. In the same patient we demonstrated the presence of a second deletion 77kb upstream of PGRN, which most likely represents a known copy number variation (CNV). We extended the PGRN mutation spectrum of which genomic PGRN deletions explain at least 1% of the genetic etiology of FTD in the Belgian sample. Our data also underscore the role of PGRN dosage in the neurodegenerative process of FTD and underline the importance of CNVs as they are indicators of genomically unstable regions where also disease-causing copy number changes can occur.

Gonzalez, Juan

Maximizing Asociation Statistics Over Genetic Models

GONZALEZ, JR; Carrasco, JL; Dudbridge, F; Armengol, L; Estivill, X; Moreno, V
Genes and disease program, Center for Genomic Regulation. Biostatistic Unit, Department of Public Health, UB. MRC Biostatistics Unit, Cambridge, UK. IDIBELL, Catalan Institute of Oncology, Barcelona, Spain.

The assessment of the association between a candidate locus and a disease requests the adoption of an inheritance model. Most researchers select the additive model and test the association with the Cochran-Armitage trend test. This test assumes a dose-response effect regards the number of copies of the variant allele. However, if there is a reason to expect dominance or recessiveness in the effect of the variant allele, the heterozygous genotype may be grouped with one of the two homozygous, depending on the inheritance model, and use a simple chi-square test on the 2x2 table to assess independence. When the underlying genetic model is unknown, association may be assessed using the max-statistic, which selects the largest test statistic from the dominant, recessive and additive models. The statistical significance of the max-statistic has been previously addressed using permutation or Monte Carlo simulation approaches. We aimed to provide simpler alternatives to the max-test to make it feasible in large-scale association studies. Our simulations show that this procedure has an effective number of tests of 2.2, which can be used to correct the significance level or p-values. We also derive the asymptotic distribution of max-statistic, which leads to a simple way to calculate the significance level and allows the derivation of a formula for power calculations in the design of studies that plan to use the max-statistic. A simulation study shows that the use of the max-statistic is a powerful approach that provides safeguard against model uncertainty.

González-Neira, Anna

SNP Versus CGH Arrays In The Detection Of Both Copy Number Changes And Copy-Neutral LOH Events In Acute Myeloid Leukemia

GONZÁLEZ-NEIRA, ANNA (1); Suela, Javier (2); Alonso, Rosario (1); Álvarez, Sara (2); Benítez, Javier (3); Cigudosa, Juan C (2)
Human Cancer Genetics Programme.
(1) Genotyping Unit; (2) Molecular Cytogenetics Group; (3) Human Genetics Group.
Spanish National Cancer Centre (CNIO). Melchor Fernández Almagro 3 . 28029 Madrid.

Traditionally, two key techniques have been used to measure DNA copy number in DNA samples: CGH is a powerful tool for the detection of amplifications and large homozygous deletions, and LOH has been used to detect regions of allelic homogeneity indicative of hemizygous deletions or copy-neutral LOH.

The development of high-density CGH array technology has enabled 100-kb resolution using whole-genome BAC arrays containing >33,000 BAC clones (Ishkanian et al. 2004) and LOH is typically assessed through the analysis of polymorphic genetic markers, traditionally either VNTRs or RFLPs (Singh et al. 1993; Dockhorn-Dworniczak et al.1994).

The introduction of high-density SNP genotyping technology to genomic profiling, represents a further advance, since simultaneous measurement of both signal intensity variations and changes in allelic composition makes it possible to detect both copy number changes and copy-neutral loss-of-heterozygosity (LOH) events.

We demonstrate the utility of SNP markers using Infinium Whole-Genome Genotyping BeadChips to detect chromosomal aberrations in Acute Myeloid Leukemia (AML). The Whole-Genome Genotyping assay is composed of four basic components: whole-genome amplification, hybridization capture on a 50mer probe array (BeadChip), array-based primer extension SNP scoring, and immunohistochemistry-based signal amplification (Gunderson et al. 2005; Steemers et al. 2006). The 317K BeadChip (Human- Hap300) consists of >300,000 tagSNP assays derived from the HapMap project with a 9-kb mean spacing between SNPs.

We analysed and compared the detection of DNA copy number by both high-density CGH and Infinium Whole-Genome Genotyping arrays in a series of 16 AMLs diagnostic samples. Results have demonstrated the high concordance between the two techniques in terms of copy number changes and at the same time have allowed copy-neutral LOH to be assessed. Copy-neutral LOH is receiving greater attention as a mechanism of possible tumor initiation (Langdon et al. 2006), and so we identified these copy-neutral LOH regions at whole genome level using SNP arrays, which has provided a detailed genetic characterization of the AML cases. We found a surprisingly high number of LOH regions that were observed consistently in multiple samples suggesting that the combined evaluation of copy number aberrations and LOH will be necessary to accurately determine the contribution of chromosomal defects to tumor development in AML.

Göransson, Jenny

Precise Genome Analyses Using Selector Probes

JENNY GÖRANSSON, Magnus Isaksson, Carolina Wählby, Mats Nilsson

Department of Genetics & Pathology, Uppsala University, Rudbeck Laboratory, SE-751 85 Uppsala, Sweden.

Cost effective and rapid methods are needed for genome-wide clinical studies of copy number variations. We describe two approaches for multiplex analysis of copy number deviations based on the selector technique [1].

Selectors are oligonucleotide probes with target-complementary end-sequences that are linked by a double-stranded general sequence comprising a universal sequence motif. In the selection process, a pool of selectors is combined with denatured restriction digested DNA. Each selector probe hybridizes to its target and specifically forms individual circular complexes. The general sequence introduced into the circularized target fragments allows parallel amplification using PCR or circle-to-circle amplification (C2CA) [2]. The selected genomic sequences are length-encoded and the PCR-products can thereby be resolved with capillary gel electrophoresis [3]. The method has been applied to verify, size, and diagnose a 133 kb duplication involving five genes causing a ridge-back phenotype in dog [4].

Rolling circle amplification of the selected genomic sequences results in single-stranded concatemer products that spontaneously form micron-sized coils. These can easily be identified using hybridization with labeled tag-oligonucleotides and fluorescence microscopy, allowing digital quantification of abundance of different sequences with high quantitative precision [5,6,7]. Multiplex decoding is enabled by serial hybridizations to immobilized rolling circle products with intermediate sandwich probes, comprising combinations of tag sequences. We are now applying the selector technique to this random array platform to validate 35 different candidate copy number deviations [Göransson et al, in preparation].

1. Dahl, F., et al., Multiplex amplification enabled by selective circularization of large sets of genomic DNA fragments. Nucleic Acids Res, 2005. 33: e71.
2. Dahl, F., et al., Circle-to-circle amplification for precise and sensitive DNA analysis. Proc Natl Acad Sci U S A, 2004. 101: 4548-4553.
3. Isaksson, M. et al. Submitted 2007.
4. Salmon Hillbertz, N.H.C., et al.., submitted 2007.
5. Blab, G.A., T. Schmidt, and M. Nilsson, Homogeneous detection of single rolling circle replication products. Anal Chem, 2004. 76: 495-498.
6 Jarvius, J., et al. Digital quantification using amplified single-molecule detection. Nat. Methods, 2006. 3: 725-727.
7 Larsson, C., et al. In situ genotyping individual DNA molecules by target-primed rolling-circle amplification of padlock probes. Nat. Methods, 2004, 1: 227-232.

Gratacòs, Mònica

Association Study Of Five Human Genes Involved In Melatonin Signaling Pathway In Mood Disorders: AANAT, MTNR1A, MTNR1B, OPN3, OPN4.

Virginia Soria (1), MÔNICA GRATACÒS (2), Juan R. González (2), Joaquín Valero (3), Èrika Martínez-Amorós (1), Mònica Bayés (2), Alfonso Gutiérrez (3), Rafael de Cid (2), José M. Crespo (1,4), Lourdes Martorell (3), Elisabet Vilella (3), Antonio Labad (3), José Manuel Menchón (1,4), Julio Vallejo (1,4), Xavier Estivill (2,5), Mikel Urretavizcaya (1)
(1) Mood Disorders Clinical and Research Unit, Psychiatry Department, Hospital Universitari de Bellvitge, Feixa Llarga s/n, L Hospitalet de Llobregat, Barcelona 08907, Catalonia, Spain; (2) Genes and Disease Program and CeGen Barcelona Genotyping Node, Center for Genomic Regulation (CRG), Charles Darwin s/n (Dr. Aiguader 88), PRBB Building, Room 521, 08003 Barcelona, Catalonia, Spain; (3) Grup d Investigació en Psiquiatria. Hospital Universitari Institut Pere Mata, Rovira i Virgili University. Reus. Tarragona, Catalonia, Spain; (4) Department of Psychiatry, Bellvitge Campus, Barcelona University. Barcelona, Catalonia, Spain; (5) Experimental and Health Sciences Department, Pompeu Fabra University, Barcelona, Catalonia, Spain.

Disruption of circadian rhythms including abnormalities of circadian phase position and melatonin secretion have been described in mood disorders (MD). We hypothesize that variants in genes encoding for melatonin signaling pathway could partially underlie the susceptibility to MD. We performed a genetic case-control study for the following candidates genes: Arylalkylamine N-acetyltransferase (AANAT), opsin 3 (OPN3), opsin 4 (OPN4), melatonin receptor 1A (MTNR1A) and melatonin receptor 1B (MTNR1B). The sample consisted of 365 unrelated patients (218 Unipolar Major Depressive Disorder, 147 Bipolar Disorder) diagnosed according to DSM-IV criteria and 419 screened control subjects. We genotyped a set of 29 TagSNPs representative of the patterns of common variation identified in European population selected from HapMap project dataset covering the entire genomic region of OPN3, OPN4, MTNR1A, MTNR1B and validated variants in AANAT. The SNPlex Genotyping System was used. Single SNP case-control association analysis considering the MD phenotype identified a positive association in MTNR1B which did not remain significant after Bonferroni correction and a significant association in a SNP located in the promoter region of AANAT which remained significant after multiple testing correction. Thus, subjects carrying the AANAT rare allele had almost two-fold probabilities of suffering from MD (OR = 1.84; CI95% = 1.32-2.56; p = 0.0003).

We have shown evidence of the association of a genetic variant in a key enzyme involved in circadian oscillations of melatonin levels in the susceptibility to MD. Our results support the hypothesis that the circadian clock mechanisms could contribute to the pathophysiology of MD.

Gräbsch, Carolin

Association Of Gene Polymorphisms Of Xenobiotica-Metabolizing Enzymes With Eczema

CAROLIN GRÄBSCH(1), Stefan Röder(1), Olf Herbarth(1)(2), Ursula Krämer(3), H.-Erich Wichmann(4), Joachim Heinrich(4), Mario Bauer(1), the LISA study group
(1) Helmholtz Centre for Environmental Research GmbH - UFZ, Department for Human Exposure Research and Epidemiology, Leipzig, Germany; (2) University of Leipzig, Medical Faculty, Environmental Hygiene and Epidemiology, Leipzig, Germany; (3) Institut für Umweltmedizinische Forschung (IUF) at the Heinrich-Heine-University Düsseldorf, Düsseldorf, Germany; (4) GSF National Research Centre for Environment and Health, Institute of Epidemiology, Neuherberg, Germany

The prevalence of eczema increased over the last decades. Until now, the causes for eczema are not well-known. Genetic predispositions as well as numerous environmental factors may play an important role for the clinical manifestation of eczema.

To examine the role of individual responsiveness to environmental pollutants, children of a Caucasian cohort (LISA-study, n=785, eczema prevalence 30,1%) were genotyped for common polymorphic xenobiotica-metabolizing enzymes involved in biotransformation of industrial pollutants, cigarette smoke and in defence against oxidative stress.

The participants were genotyped for the xenobiotica-metabolizing enzymes CYP2D6 (*1, *4 alleles), GSTM1 (wt, null alleles); GSTT1 (wt, null alleles) and GSTP1 (*1A, *1B alleles). The study revealed, that children, heterozygote for GSTP1 (*1A/*1B) which additional carry a CYP2D6*4 allele (CYP2D6*1/*4 or CYP2D6*4/*4) had a decreased risk for eczema (OR=0.53, 95%CI: 0.53-0.34, p<0.01) after adjustment for smoking during pregnancy, gender, renovation during pregnancy and positive family history of atopy. This association was confirmed by a cross-check (homozygote for GSTP1 in conjunction with missing CYP2D6*4 allele), expressing a trend of increasing risk for eczema (OR 1.36, 95%CI: 0.99-1.86, p=0.056).
We conclude that the risk association identified in our study indirectly points to xenobiotics as etiologic agents for eczema.

Guðbjartsson, Hákon

Population Based Genetic Variation Analysis Using The DiseaseMiner Software System

H. GUDBJARTSSON, S. Stefansson, A. Gylfason, R. Valdimarsson, O. Arthursson, V. Palsson, K. Roth, G. Masson, B. Palsson

Over the last decade, deCODE genetics Inc. has developed various software systems in order to conduct population-based genetic research in Iceland. The DiseaseMiner Professional system is a software platform for downstream genetic variation analysis that deCODE is currently offering to its genotyping customers. Here we describe how the DM system is ideally suited to handle the large volume of genetic data from modern SNP chip platforms and how we have provided additional functionality in the system for copy number variation analysis.

The main components of the DiseaseMiner are: An ad-hoc query and reporting module, based on a proprietary Set Definition Language (SDL), which allows users easily to express simple as well as advanced disease models based on subjects phenotypes and the family data. Similarly, the SDL system can be used to find genomic features such as genes and markers based on public attributes as well as annotations derived in the DiseaseMiner system. A genealogy database with advanced family algorithms to find related and unrelated individuals based on multiple inheritance models is incorporated. Family relationships are also easily used in the SDL queries, such as to express disease conditions based on the phenotypes of children or parents. The genetic analysis is primarily based on statistical software algorithms developed at deCODE, namely Allegro and NEMO for linkage and haplotype analysis respectively. These programs are bundled with a load balancing and queueing system as well as tools to present the statistical results, e.g. a genome browser.

Genotypes are not directly integrated into the ad-hoc query tools due to their large volumes and the nature of their use. Rather, summary information of genotypes is stored in the database on subjects as well as in the marker database but the genotypes themselves are in socalled genotype providers. At deCODE, where we store genotypes on tens of thousands of subjects, we have found relational database technology impractical to store the large number of genotypes that results from chip based SNP typing. Thus, we typically use a binary based file format to store SNP genotypes. The genotype provider in the DiseaseMiner does however have an interface that encapsulates not only genotypes stored in binary formats but also in our legacy relational table structure and text files. The genotype provider does also provide logic to merge genotypes from multiple sources and equivalent markers, e.g. due to differences in names and assays. For single point genome wide case-control analysis, with thousand subjects and three hundred thousand SNPs, we find that the analysis time is in tens of minutes when genotypes are accessed through the Java based genotype provider interface whereas if the algorithms accesses the binary file directly, the analysis time is measured in tens of seconds. For multipoint haplotype analysis, where more computations are needed such as in the EM steps of NEMO, the difference is reduced because of a smaller fractional cost of data loading.

The high density of SNPs and the quality of modern chips makes intensity analysis of their signal interesting to capture copy number variation (CNV) information. By analysing the SNP intensity signal in a sliding genomic window, we convert this signal into discrete CNV genotypes. Subjects and markers can be searched for based on CNV attributes and the existing tools in the DiseaseMiner, such as NEMO and the haplotype tool can be used to inspect CNV information in conjunction with other data, e.g. perform inheritance checks on CNV status or estimate the conservation of CNV using case-control analysis.

Hakonarson, Hakon

A Pediatric Genome-Wide Association Study Identifies A Novel Type 1 Diabetes Gene

HAKONARSON, HAKON (1,2), Grant, Struan F.A. (1,2), Bradfield, Jonathan P. (1), Marchand, Luc (3), Kim, Cecilia E.(1), Glessner, Joseph T. (1), Grabs, Rosemarie (3), Casalunovo, Tracy (1), Taback, Shayne P. (4), Frackelton, Edward C. (1), Lawson, Margaret L. (5), Robinson, Luke J. (1), Skraban, Robert (1), Lu, Yang (3), Chiavacci, Rosetta M. (1), Stanley, Charles A. (6), Kirsch, Susan E. (7), Rappaport, Eric F. (8), Orange, Jordan S. (9), Monos, Dimitri S. (9,10), Devoto, Marcella (2,11), Qu, Hui-Qi (3), Polychronakos, Constantin (3).

(1) Center for Applied Genomics, and (2) Division of Human Genetics, Children's Hospital of Philadelphia; (3) Departments of Pediatrics and Human Genetics, McGill University, Montreal; (4) Department of Pediatrics and Child Health, University of Manitoba, Winnipeg; (5) Division of Endocrinology, Children's Hospital of Eastern Ontario, University of Ottawa, Ottawa; (6) Division of Endocrinology, Children's Hospital of Philadelphia; (7) Markham-Stouffville Hospital, Markham, Ontario; (8) The Children's Hospital of Philadelphia Nucleic Acid and Protein Core, Philadelphia; (9) Department of Pediatrics University of Pennsylvania, School of Medicine, Philadelphia; (10) Department of Pathology and Laboratory Medicine, The Children's Hospital of Philadelphia; (11) CCEB, University of Pennsylvania, Philadelphia

Type 1 diabetes (T1D) is strongly heritable, with first-degree relatives of cases being at 15 times greater risk than the general population. A number of genetic determinants of T1D have already been established through candidate gene studies, primarily within the MHC but also with other loci. To identify novel genetic factors that confer risk to the pathogenesis of T1D, we performed a genome-wide association study using the Illumina Infinium HH550 platform in a large pediatric cohort of Western European decent. In addition to confirming previously identified loci, we observed highly significant association with variation within a novel locus. Three common non-coding variants in strong LD reached genome-wide significance for T1D association (P-value range= 6.12x10-8 - 1.03x10-10, OR range= 0.65 - 0.66). A subsequent TDT replication study in an independent cohort confirmed that variation within this gene is associated with T1D.

To investigate whether genotype influences expression of the gene, we utilized a synonymous SNP in strong LD with the associated variant class to evaluate relative abundance of each allele in steady-state mRNA from ten lymphoblastoid cell lines; allele ratios in mRNA, determined through bi-directional sequencing, were not different from those in DNA, indicating no such effect. However, expression of the gene in natural killer (NK) cells, a pivotal lineage in the pathogenesis of T1D, showed that its mRNA is expressed differently in cells homozygous for the protective variant (NKL cell line).

These results provide evidence for a promising lead to new T1D therapeutics. The identity of the locus will be presented.

Hall, Ira

Recurrent DNA Copy Number Variation In The Laboratory Mouse

Chris M Egan(1), Srinath Sridhar(2), Michael Wigler(1), IRA M HALL(1)

(1) Cold Spring Harbor Laboratory
(2) Computer Science Department, Carnegie Mellon University

Variability in segmental DNA copy number is now recognized to be an abundant source of genetic variation in mammals, but the manner and frequency with which these differences arise over generational time is not well understood. Taking advantage of genetic divergence between closely related lineages, we have conducted a genome-wide investigation into the nature of spontaneous DNA copy number change in the laboratory mouse. We used high resolution microarrays to identify de-novo copy number variants (CNVs) between 14 colonies of the C57BL/6 strain spanning approximately 966 generations of inbreeding, and genotyped these CNVs across 12 commonly-used inbred strains. We find recurrent copy number mutation to be remarkably common: nearly half of the variants that we identified arose more than once in the history of inbred mice, and some CNVs appear to recur at extremely high rates. In contrast most of the genome appears to be relatively stable. That most recurrent CNVs contain one or more entire genes raises questions about their contribution to natural variation.

Hallast, Pille

The Role Of Gene Conversion In Shaping The Human And Chimpanzee Duplicated LHB/CGB Gene Cluster

PILLE, HALLAST(1); Aarno, Palotie(2); Maris Laan(1)
(1)Institute of Molecular and Cell Biology, University of Tartu, Tartu, Estonia; (2)Finnish Genome Center, University of Helsinki, Helsinki, Finland

Human genome is rich in segmental duplications, which comprise approximately 5% of the genome, range up to several hundred kilobases in size and show very high sequence similarity (90%-100%). Duplications have an important role in gene evolution and genome rearrangement, and they may be involved in phenotypic variation, including differences in disease susceptibility. Duplicons are prone to gene conversion known to homogenize and spread mutations.

One of the gene families that has evolved through duplication events in the primate lineage is the Gonadotropin Hormone Beta subunit (GtHB) family, which includes the Luteinizing hormone/ Chorionic Gonadotropin beta (LHB/CGB) genome cluster. The LHB/CGB genes encode for beta-subunit of gonadotropin hormones that have an essential role in fertility and reproduction. The few described nonsynonymous mutations lead to either infertility or reduced gonadal function. The human LHB/CGB gene cluster (19q13.3) consists of seven highly homologous (85-99%) genes: 1 LHB and 6 CGB genes. Recently we resequenced the LHB/CGB genes in three human populations. We found the diversity level of the cluster being one of the highest reported for human genes and that directional gene conversion from the center towards the periphery of the cluster has had an important role in spreading polymorphisms among the duplicon copies and generating short-range LD around them (Ref 1).

In order to study the recent evolution of the segmentally duplicated LHB/CGB genome cluster, we constructed a shotgun library and sequenced the entire LHB/CGB cluster in the common chimpanzee. Comparison of the human and chimpanzee clusters showed that chimp cluster (1 LHB, 5 CGB) harbours one CGB gene less than human cluster (1 LHB, 6 CGB). The mean sequence divergence between human and chimp LHB/CGB clusters was 5% (2.3% substitutions, 2.7% indels) that is substantially higher compared to single-copy regions (1.2-1.5%). We also found that the fraction of transitions was ~10% lower in the studied cluster compared to unique genomic regions (Ref 2,3). Active intraspecies gene conversion between multiple duplicated and highly homologous segments might be one explanation for both the high divergence and high proportion of tranversions in the cluster. Gene conversion has no preference for either transitions or transversions and within species any *de novo* arisen mutation has a potential to be spread from the original locus to other gene copies. We also found that the nucleotide divergence within genes decreased substantially (up to half) when human intraspecific diversity was taken into account (Ref 1) indicating that the divergence estimates between human and chimpanzee might be substantially lower than reported when the intraspecies variation is taken into account.

1. Hallast P et al (2005) Gen Res 15(11):1535-1546
2. Ebersberger I et al (2002) Am J Hum Genet 70, 1490-73
3. Anzai T et al (2003) Proc Natl Acad Sci U S A 100, 7708-13

Hansen, Thomas

Genetic Analyses Of Brain Expressed MicroRNAs In Schizophrenia

THOMAS HANSEN(1,8), Line Olsen(1), Morten Lindow(2), Klaus D. Jakobsen(1), Henrik Ullum(3), Erik Jonsson(4), Ole A. Andreassen(5), Srdjan Djurovic(5), Ingrid Melle(5), Ingrid Agartz(4), Håkan Hall(4), Sally Timm(6), August G. Wang(7), Thomas Werge(1)
(1) Research Institute of Biological Psychiatry, Sct. Hans Hospital, DK-4000 Roskilde, Denmark; (2) Bioinformatics Centre, Institute of Molecular Biology, University of Copenhagen, Denmark; (3) Department of Clinical Immunology, Rigshospitalet, Copenhagen, Denmark; (4) Human Brain Informatics (HUBIN), Department of Clinical Neuroscience, Psychiatry Section, Karolinska Institutet and Hospital, Stockholm, Sweden; (5) TOP-project, Department of Psychiatry, Ullevål University Hospital, Oslo, Norway; (6) University Department of Psychiatry, Frederiksberg Hospital, DK-2000 Frederiksberg, Denmark; (7) University Department of Psychiatry, Amager Hospital, DK-2300 Copenhagen S, Denmark; (8) Centre for Pharmacogenomics, University of Copenhagen, 2200 Copenhagen N, Denmark

Background: Protein encoding genes have been the major targets for research in schizophrenia genetics. However, with the identification of regulatory microRNAs (miRNAs) in human brain tissues, brain-expressed miRNAs genes have emerged as good as candidates for schizophrenia. Indeed, the grooving understanding of the regulatory properties and pleiotrophic effects that miRNA have on molecular and cellular mechanisms, suggests that alterations in the interactions between miRNAs and their mRNA targets may contribute to phenotypic variation.

Methodology/Principal Findings: We have studied the association between schizophrenia and genetic variants associated with genes for brain-expressed miRNAs using a case-control study design on three independent Scandinavian samples. Data were analyzed using a 2-step strategy with full analysis of 18 known SNPs within or near brain-expressed miRNAs in a Danish sample (384 schizophrenia patients and 808 control subjects). Subsequently, a joint analysis in all three samples (in total 765 cases and 1274 control subjects) was performed on SNPs showing nominal significant association in step 1. One SNP (rs17578796) in hsa-mir-206 showed nominal significant allelic (P=0.007) and genotypic (P=0.007) association to schizophrenia in the Danish sample. However, a joint analysis of this variant in all three Scandinavian samples did not support this finding.

Conclusions/Significance: In this early attempt to determine a miRNA disease relationship we found no association between brain-expressed miRNAs and schizophrenia. This does not exclude the possibility that associations exist for variants of other miRNAs thus more research in this area are encouraged.

Henrichsen, Charlotte N.

Submicroscopic Deletion In Patients With Williams-Beuren Syndrome Influences Expression Levels Of The Nonhemizygous Flanking Genes

CHARLOTTE N. HENRICHSEN, Giuseppe Merla, Cédric Howald, Alexandre Reymond
Center for Integrative Genomics, University of Lausanne, Lausanne, Switzerland; Servizio Genetica Medica, IRCCS Casa Sollievo della Sofferenza, San Giovanni Rotondo, Italy

Segmental aneuploidies are important contributors to human diseases and, potentially, to phenotypic variation, as well as a major force of evolutionary changes. There is evidence that such genomic insertions or deletions contribute to phenotypic variation by modifying the expression levels of genes within the aneuploid segments. We hypothesise that these large chromatin rearrangements influence the transcription levels of genes that map centromerically or telomerically to the critical region and the repeats, even if these genes are present in two copies.

To test this hypothesis we used real-time quantitative PCR to accurately measure the expression of genes mapping to the recurrent deletion on chromosome 7q11.23 harboured by Williams-Beuren syndrome (WBS) patients. We studied in 20 WBS patients and 20 controls the relative expression of 51 genes mapping within the WBS critical region, its flanking repeats and neighboring regions in two different cell lines (skin fibroblasts and lymphoblastoids). As anticipated, in WBS samples almost all the genes mapping to the common deletion interval show relative levels of expression decreased by 50%. Remarkably, a decrease in relative expression, albeit not as large, was observed for the non-hemizygous genes that map on both sides of the common deletion region.
These results suggest that in genomic disorder not only the aneuploid genes but also the genes that map close to the rearrangement should be considered as candidate genes for the specific features of these pathologies.

Hesselson, Stephanie

Natural Variation In 115 Human Membrane Transporter Genes

HESSELSON, STEPHANIE, Kroetz, Deanna, Giacomini, Kathy, Kwok, Pui-Yan
Cardiovascular Research Institute and Department of Biopharmaceutical Sciences, University of California, San Francisco

Genetic variation in membrane transporters alters drug absorption, distribution, and elimination among individuals. Polymorphisms in the promoters of genes that alter the protein levels of these membrane transporters may also play a role in adverse drug reactions and drug efficacy. We sequenced 250bp upstream and 50bp downstream of the mRNA start site of 115 promoters in order to identify and estimate the allele frequency of polymorphisms in the proximal promoters of these genes. This region was sequenced in 272 individuals from the SOPHIE cohort. These 272 samples consisted of 68 African Americans, 68 Caucasians, 68 Chinese and 68 Mexicans from the Bay area. Currently 364 polymorphisms have been identified. The majority of these polymorphisms are SNPs, but 35 indels have been identified as well. There are 1.6 fold more mutations observed in the proximal promoters than were previously observed in the same length of exonic sequence of previously sequenced membrane transporters. Sixty-eight percent of the polymorphisms observed in the proximal promoters are population specific. Most of the population specific alleles were found in the African American samples. This was true for singletons and for polymorphisms with allele frequencies above 1%. Asians had the second highest level of population specific alleles. In cases where 3 populations had minor alleles present, Asians most frequently were not among those three populations. This is most likely due to the European admixture of Mexicans and African Americans. The distribution of minor alleles in the proximal promoters is 42% singletons, 36% with a minor allele frequency between 1-10%in at least one population, 7% with a minor frequency between 10-20% in at least one population, and 13.5% with a frequency greater than 20% in at least one population. There are more polymorphisms with allele frequencies below 1% and fewer polymorphisms with minor alleles above 20% than would be expected under the neutral mutation model. Common polymorphisms in the proximal promoters are being tested to determine if they alter expression levels using cell lines with a GFP assay. The transport of substrates across cell membranes *in vitro* will also be tested with altered levels of membrane transporter protein. Individuals that carry alleles that have been shown to alter expression levels of membrane transporters and affect drug metabolism are available for *in vivo* testing. The polymorphisms identified in this study will lead to a better understanding of how natural genetic variation effects drug metabolism and may help to identify individuals who will have a deleterious reaction to commonly used medications.

Hogan, Kirk

Perioperative Genomic Profiles By Structure-Specific Cleavage Of Oligonucleotide Probes

KIRK HOGAN, M.D., J.D., James K. Burmester, Ph.D., Michael D. Caldwell, M.D., PhD., Quinn H. Hogan, M.D., Douglas B. Coursin, M.D., Dawn N. Green, M.S., Rebecca M.R. Selzer Ph.D., Thomas P. Broderick, M.D., Deborah A. Rusy, M.D., Mike Poroli, B.S., Anna L. Lutz, B.S., Allison M. Sanders, B.S., Mary C. Oldenburg, M.S., James A. Koelbl, M.S., Monika de Arruda-Indig, Ph.D., Michael J. Domanico, Ph,D.
Department of Anesthesiology, University of Wisconsin School of Medicine and Public Health, Madison, Wisconsin USA 53792, Marshfield Clinic Research Foundation, Marshfield, Wisconsin USA54449, Department of Anesthesiology, Medical College of Wisconsin, Milwaukee, Wisconsin USA 53226, Third Wave Technologies, Inc., Madison, Wisconsin USA 53719

Introduction: Adverse events occur in over 20% of anesthetics, with 3% or more resulting in moderate, serious or catastrophic sequelae[1]. The prevalence and severity of many perioperative complications are strongly associated with genetic predispositions that are often unknown in advance of surgery, including drug toxicity and inefficacy, aberrant thrombosis, prolonged paralysis, bronchospasm, and sepsis.

Methods: With IRB approval, 450 patients undergoing general anesthesia and surgery were tested for 50 polymorphisms (e.g., SNPs, CNVs) in genes predicting phenotypes of interest to perioperative caregivers i.e., BChE, B 2AR, HBB, ApoE, MYH7, FII, FV, MTHFR, MTR, MTRR, CBS, TPMT, CCR5, TNF a, TNF b, CYP2C9, CYP2C19, CYP2D6, CYP3A4, CYP3A5, ABC, ACE, Gender, and ABO. Using structure-specific invasive cleavage of oligonucleotide probes (InvaderTM, Third Wave Technologies, Inc.), 96-well plates were configured so that each well contained reagents for detection of both the wild-type and mutant alleles at each locus. Three plates were used per patient with inclusion of positive control and no-template negative control assays. Each assay was performed at the test site of sample origin, and repeated at the manufacturer's reference laboratory.

Results: A total of 22,500 genotypes were obtained and confirmed in duplicate. After polymorphisms in non-pathogenic genes (i.e., the ABO blood group and gender-specific alleles) were withdrawn from analysis, 391 of 450 patients were found to be mutant homozygotes at 1 or more loci, with a mean number of 2 mutant homozygous loci per patient. In turn, a mean of 11 mutant alleles in aggregate (i.e., homozygous plus heterozygous mutant polymorphisms) were observed per patient. Of 45,000 genotypes, 165 (0.37%) were replicated because of low signal intensity, and resolved. 164 (0.36%) were replicated because of signal intensity in the equivocal range, and resolved. Discordant genotypes were observed in 58 of 22,500 (0.25%) assays compared between the test center of origin and the manufacturer's reference laboratory. Of these 55 were resolved on replication. The remaining 3 pairs of templates were directly sequenced, and resolved.

Conclusion: These results demonstrate that significant genetic heterogeneity is present in most patients in advance of surgery, and cannot be accounted for using contemporary tools for detection, e.g., a family history check-box. As well, structure-specific cleavage of oligonucleotide probes in biplex formats provides a robust, accurate, and efficient platform for intermediate- to high-throughput clinical applications. Future investigations will expand the perioperative allele panel, and test the efficacy of genomic profiles in preventing deleterious outcomes during and after surgery and anesthesia.

Reference:

1. Bothner, U. et al., Building a large-scale perioperative anaesthesia outcome-tracking database: Methodology, implementation, and experiences from one provider within the German quality project. Br J Anaesth 2000; 85:271-80.

Hollox, Ed

Genotyping Copy Number And Sequence Variation Of FCGR3 Using Paralogous Ratio Tests (PRT).

Dehnugara, Tushna and HOLLOX, EDWARD J
Department of Genetics, University of Leicester, UK

Accurate determination of copy number variation (CNV), especially at high copy number, has posed a formidable technical challenge. In particular, large-scale association studies require methods capable of genuinely high throughput without compromising quality. We have found that a Paralogue Ratio Test (PRT), which adapts multiplex PCR to use a single primer pair to amplify both test and reference loci, does provide the necessary accuracy, throughput and economy. Low copy number of the Fc gamma receptor gene FCGR3 has previously been shown to be associated with glomerulonephritis in patients with systemic lupus erythematosus (SLE). This gene, together with the other members of the FCGR family at the same chromosomal locus, is a candidate for altering susceptibility to inflammatory and autoimmune disease.

We show how sequence variant genotyping coupled with copy number genotyping allows determination of FCGR3 total copy number, relative copy numbers of FCGR3A and FCGR3B and allelic status of NA1/NA2 allotypes at FCGR3B. We present data on a UK population to determine allele frequencies at this locus, and to examine the relationship between copy number and sequence variation.

Inouye, Michael

A Fast Genotype Calling Algorithm For The Illumina Genotyping Beadchip Platforms

Teo, Yik Y.; INOUYE, MICHAEL; Clark, Taane; Small, Kerrin; Kwiatkowski, Dominic; Deloukas, Panos
Wellcome Trust Sanger Institute

The Illumina BeadChip series consists of high density oligonucleotide genotyping arrays which enable whole genome genotyping of up to 655 thousand SNP markers. The large number of markers mean that automated genotype calling procedures are required for assigning genotypes from the hybridization intensities.

There have been recent advances in genotype calling algorithms which focus on the use of computationally intensive Bayesian and supervised-learning algorithms to cluster genotype clouds for each SNP. Such techniques typically require initial processing of the intensity data to suitable coordinates before running the algorithms and this pre-processing step is usually identical for every SNP. Due to fluctuations in the extent of the hybridization for SNPs across the genome, a pre-processing scheme which is fixed for every SNP on the platform does not yield the same genotype calling efficacy as desired for every SNP. This can result in the calling algorithms underperforming for a fraction of the SNPs.

Here, we present a novel and fast genotype calling algorithm that combines the phases of pre-processing and genotype calling by first identifying an optimal transformation of the intensity data for each SNP and using an Expectation-Minimization-based procedure with outlier detection to assign the genotypes. By identifying suitable transformations which produce distinct genotype clusters, we ameliorate the calling procedure without requiring fanciful and computationally-intensive statistical algorithms.

We apply the algorithm to hybridization intensities generated from multiple genome wide chips for several cohorts of varying DNA quality, and evaluate its performance relative to the proprietary Illumina GenCall algorithm.

James, Michael

Serotonin Transporter Genotype X Environment Interaction For Risk Of Depression.

JAMES, MICHAEL R.; Coventry, Will. L.; Wray, Naomi; Martin, Nicholas G.
Queensland Institute of Medical Research, Brisbane, Australia.

Since the 2003 landmark paper of Caspi et al. there have been many inconclusive reports on the interaction between variation in the promoter length polymorphism (HTTLPR) of the serotonin transporter gene SLC6A4 (previously 5HTT) and increased risk for depression, contingent on stressful life events (SLE). Among many inconsistent reports, some support the original finding, some find no interaction and others report an interaction in the opposite direction. In addition it has become clear that the HTTLPR is more complicated than originally thought and that other polymorphisms within both the LPR and the gene may contribute to the variable expression.

To improve the accuracy of the genotyping of the Long/Short (L/S) alleles of the HTTLPR we designed two small PCR products which could be 4-fold multiplexed on gels which allowed triplicate data points from two independent PCRs in an efficient laboratory protocol. We devised a MassArray extension assay for the A/G variant (rs25531) in repeat VI (deleted in the S allele) of the HTTLPR so as to define La and Lg alleles. It has been reported that Lg is functionally equivalent to S but until recently most studies did not survey this variant. This simple assay was multiplexed with a dozen SNPs in the SLC6A4 gene, promoter and potential microRNA target sites in the 3'UTR, for genetic association and haplotype analyses. Finally we typed the VNTR within intron-2 that has been reported to affect expression of the gene.

Previous uncertainties seen in these studies may have been partly due to incomplete and/or inaccurate genotype information combined with a lack of power due to the often small sample size. Further, it has been suggested that the GxE detected may be an artefact of the psychometric properties of the psychiatric constructs employed. We have addressed each of these issues and recently have completed data collection for our large twin family collection (N~7,000 individuals) for whom depression diagnoses, risk factors and DNA are available. Comprehensive data analysis should bring considerable power to the question of GxE interaction for depression.

Jonasdottir, Aslaug

The Megasatellite Family Of Copy Number Variants Are Very Polymorphic And May Affect Expression Of Genes Within Or Near Them

ASLAUG JONASDOTTIR, Adam Baker, Gisli Masson, Sigridur Reynisdottir, Adalheidur Olafsdottir, Patrik Sulem, Catalina Lopez-Correa1, Birgitta Birgisdottir, Shyamali Ghosh, Soley Bjornsdottir, Sigurborg Matthiasdottir, Adalbjorg Jonasdottir, Daniel Gudbjartsson, Kari Stefansson, Unnur Thorsteinsdottir, Jeffrey R. Gulcher
deCODE Genetics, Sturlugata 8, IS-101 Reykjavik, Iceland

Large tandem repeats with unit length greater than 500bp, which some have been defined as megasatellites, are distributed throughout the human genome. We developed an algorithm for systematic prediction of large (>500 bp) head-to-tail tandem repeats, which subsequently identified 207 megasatellites in the human genome. About 40% of these megasatellites overlap with known genes or predicted genes. They have a broad range in repeat sizes from 512bp up to 262,000bp, illustrating that it is important to carry out this analysis with large window sizes. However, the vast majority of megasatellites (80%) had a unit size between 550bp and 10,000bp. There was also a broad range in the predicted number of repeats within each megasatellite, ranging from 2 to 43, with an average repeat number of 6.5. We have tested 33 of these predictions using Pooled Pulsed-field Southern Blotting (PPSB), and TaqMan quantitative PCR, and found the overall polymorphism rate to be 73%. Our Southern blotting method is based on size of the overall restriction fragment containing the entire megasatellite rather than on intensity and is therefore more precise than quantitative PCR. The human sequence assembly collapses most megasatellites to fewer than the average number of repeats. Here we show results for three independent megasatellites analyzed in 96 individuals (16 HapMap CEU triads and 15 Icelandic control triads) using the Illumina iSelect CNV beadchip. We demonstrate the polymorphic state of these megasatellites and the tight correlation of the observed copy numbers between the beadchip and the two validation platforms, Southern blotting and quantitative PCR. The three megasatellites overlap known genes PGA3, AK097615, and NSF, respectively, and we demonstrated a significant positive correlation between the expression of the three genes and copy number of the corresponding megasatellites. These findings show the potential of how the megasatellite class of copy number variations can have implications for development of complex genetic traits, and why it is very important to screen the genome systematically for new and novel structural variations.

Kapasa, Maria

In Silico Analysis Of Regulatory Elements Of WISP1 And Orthologs. Comparisons With The Coregulated CTGF Gene.

M. KAPASA, A. Gavalas & S. Kossida
Biomedical Research Foundation of the Academy of Athens, Athens, Greece

WISP1 and CTGF are members of the CCN family of growth factors. They both encode secreted proteins belonging to the extracellular matrix and participating in several developmental and tumorigenic processes (Moussad & Brigstock, 2000). WISP1 is a connective tissue growth factor induced by the Wnt signaling pathway. CTGF is the most studied member of the CCN family (Sharpe et al., 2001, Xu et al., 2000, Grotendorst 1997, Oemar and Lüscher 1997, Bradham et al., 1991) and shares common domains (modules) with WISP1. Module 1 is an insulin-like growth factor (IGF)-binding domain, module 2 is a von Willebrand type C domain, module 3 is a thrombospondin-1 domain, and module 4 is a C-terminal domain containing a putative cystine knot. (Bork 1993, Brigstock 1999, Lau & Lam 1999, Perbal 2001). Microarray data indicated that WISP1 and CTGF are both regulated by neurogenin 3 (NGN3), a transcription factor playing a key role in the specification of the endocrine pancreas. WISP1 orthologs were identified from all currently available genomes. The promoter regions of WISP1 gene and orthologs were searched for putative regulatory motifs and common transcription factor binding models were identified. Comparative analysis of CTGF and its orthologs revealed the presence of a conserved enhancer in both WISP1 and CTGF regulatory regions. This conserved enhancer contains NGN3 binding sites as well as binding sites for other transcription factors such as PDX1, HNF6 and HNF1, crucial for pancreatic development.

Katoh, Masaru

SNPs Of Genes Encoding Stem Cell Signaling Components In Gastric Cancer

Masuko Katoh (1), and MASARU KATOH (2)
(1) M & M Medical BioInformatics, (2) National Cancer Center, Japan

WNT signaling molecules play key roles during embryogenesis, tissue regeneration, and carcinogenesis. We engaged in the wet biology of WNT signaling molecules during 1996-2002 to clone and characterize WNT2B, WNT3, WNT3A, WNT5B, WNT6, WNT7B, WNT8A, WNT8B, WNT9A/WNT14, WNT9B/WNT14B, WNT10A, WNT10B, WNT11, FZD1, FZD2, FZD3, FZD4, FZD5, FZD6, FZD7, FZD8, FZD10, FRAT1, FRAT2, NKD1, NKD2, VANGL1, RHOU/ARHU, RHOV/ARHV, GIPC2, GIPC3, FBXW11/betaTRCP2, SOX17, and TCF7L1/TCF3. We then engaged in the dry biology of WNT signaling molecules to identify and characterize PRICKLE1, PRICKLE2, DACT1/DAPPER1, DACT2/DAPPER2, DAAM2 and BCL9L (Ref 1). Recently, we have been working on the stem cell signaling network consisting of WNT, Notch, FGF, Hedgehog and BMP signaling pathways (Ref 2-5).

Genetic factors, Helicobacter pylori infection, salt over-uptake, smoking, metabolic syndrome, and decreased consumption of vegetables, fruits, or tea are risk factors of human gastric cancer. Germline mutations of CDH1 gene encoding E-cadeherin, and SNPs of PTPN11 (SHP2), TLR4, IL1B, TNFA genes are associated with gastric cancer.

SNPs of genes associated with gastric cancer had been searched for with the genome-wide approach in Japan during 2000 to 2004 fiscal years by consuming more than 100 million US dollars, and only raw data for the allele frequencies of SNPs among gastric cancer patients were disclosed in 2006. Because their data were not user-friendly, we carried out meta-analyses on the disclosed SNP data.

We selected the following 18 genes encoding the stem cell signaling molecules from their raw data: WNT4, MARK3, CELSR1, ROR2, DAAM1, MAML2, MAML3, FGF6, FGF19, GLI3, BMP6, BMP7, GDF15, RUNX1, RUNX3, POU2F3, POU5F1, and GATA4 genes. We next investigated expression of these 18 genes in gastric cancer, and selected MARK3, ROR2, DAAM1, MAML3, BMP6, GDF15, RUNX1, RUNX3, POU2F3, POU5F1, and GATA4 genes. We then compared the allele frequencies of the 11 genes in gastric cancer patients and those in the general Japanese population to finally select SNPs of BMP6, GDF15, and RUNX3 genes associated with gastric cancer.

[References]

(1) Special Topics of Fast Moving Fronts published by the Thomson ESI (http://esi-topics.com/fmf/2005/september05-MasaruKatoh.html).
(2) Katoh M, Katoh M. Bioinformatics for cancer management in the post-genome era. Technol Cancer Res Treat 5: 169-176, 2006.
(3) Katoh Y, Katoh M. Hedgehog signaling pathway and gastrointestinal stem cell signaling network. Int J Mol Med 18: 1019-1023, 2006.
(4) Katoh M, Katoh M. Crosstalk of WNT and FGF signaling pathways at GSK3beta to regulate beta-catenin and SNAIL signaling cascades. Cancer Biol Ther 5: 1059-1064, 2006.
(5) Katoh M, Katoh M. Notch signaling in the gastrointestinal tract. Int J Oncol 30: 247-251, 2007.

Kauwe, John

Candidate Gene Screen Identifies SNPs Which May Influence Risk For Alzheimer's Disease By Modulating Cerebrospinal Fluid Amyloid β Levels

JOHN S.K. KAUWE, Sumi Chakraverty, Anne M. Fagan, David M. Holtzman, John C. Morris Alison M. Goate.
Washington University School of Medicine, St. Louis, MO 63110

INTRODUCTION: Thousands of SNPs in hundreds of biological and positional candidate genes have been evaluated for association with Alzheimer's disease (AD). With the exception of the ApoE4 allele, no allele has shown consistent and replicable association with AD. Bertram et al (2004) suggested that current case-control approaches may not have the statistical power to detect the small effect sizes we might expect in a complex disease such as AD. Statistical power is further degraded by the inherent heterogeneity of samples ascertained using subjective clinical exams. Meta-analyses of published results available at Alzgene.org address the problem of small sample sizes to some extent and have identified single nucleotide polymorphisms (SNPs) that are associated with AD. The use of quantitative endophenotypes for studying the genetics of complex disease may also confer several advantages, providing greater power because they are less heterogeneous than clinical diagnoses and more directly affected by genetic variation. Aggregation of amyloid β (Aβ) peptides into insoluble plaques in the brain is a central feature of AD pathology and is reflected in cerebrospinal fluid (CSF) levels of Aβ. In this study we have used CSF Aβ levels as an endophenotype for AD in a screen of 1536 SNPs in 40 candidate genes.

METHODS: CSF was collected from 262 subjects. CSF Aβ levels were measured using ELISA. Using the Illumina Golden Gate technology, 1536 SNPs were genotyped. TagSNPs for 40 AD and Aβ candidate genes were selected to represent bins in which pairwise r^2 values exceeded 0.8. SNPs with functional annotations and those located in gene regions conserved between humans and mice were also genotyped. A set of SNPs that are associated with LOAD in the Alzgene.org meta-analysis were also included. SNPs were analyzed for association with CSF total Aβ (Aβ40 + Aβ42) and CSF Aβ42/Aβ40 ratio by genotype using ANCOVA after adjusting for age, gender, clinical dementia rating (CDR) and the number of ApoE4 alleles. Multiple test corrections were performed using eigen values of the correlation matrix as presented by Li and Li (2005). For a set of high priority genes with strong prior hypotheses corrections were performed considering only the SNPs that were genotyped in each individual gene region. Other genes were grouped together for multiple test correction.

RESULTS: We analyzed data for approximately 1,300 SNPs after removing SNPs which were non-polymorphic or deviated from Hardy-Weinberg equilibrium in non-demented individuals. SNPs in four genes were significantly associated with total Aβ levels and/or the CSF A²42/A²40 ratio after multiple test correction. Two SNPs which were significant in the Alzgene meta-analysis also show significant association ($p<0.05$) with CSF Aβ levels in our sample.

CONCLUSIONS: Using a novel endophenotype-based approach we have identified SNPs and genes which may influence risk for AD by modulating CSF Aβ levels. These data suggest that the use of CSF Aβ levels as a quantitative endophenotype will provide an alternative and powerful approach for the identification of novel genetic risk factors for AD.

REFERENCES:
Bertram, L. and R. E. Tanzi (2004). "Alzheimer's disease: one disorder, too many genes?" Hum Mol Genet 13 Spec No 1: R135-41.
Li, J. and L. Ji (2005). "Adjusting multiple testing in multilocus analyses using the eigen values of a correlation matrix." Heredity 95(3): 221-7.

Kaya, Namik

De Novo Copy Number Variations In Unknown Dysmorphic Syndromes.

KAYA, NAMIK; Al-Zahrani, Jawaher; Colak, Dilek; Al-Odaib, Ali; Inan, Mehmet; Nester, Michael; Al-Owain, Mohammad; Al-Hasnen, Zohair; Ozand, Pinar; Sakati, Nadia; Meyer, Brian.
King Faisal Specialist Hospital and Research Center, PO Box 3354, Riyadh 11211, KSA.

One of the major research challenges is the identification of chromosome changes in various types of dysmorphic syndromes. Until recently, the research has been hampered by the lack of high resolution techniques to study the possible alterations. Another major obstacle has been the absence of inheritance of most such disorders, precluding the crucial genetic linkage studies. These disorders mostly are *de novo* changes indicating germ line mutations. Recently, two leading groups working on autism published their results in Nature Genetics (39:319-328, 2007) and Science (316:445-449, 2007). One of these groups, Sebat et al. (Science 316:445-449, 2007) conducted a comparative genomic hybridization (CGH) on the genomic DNA of autistic subjects and their parents. The CGH was performed by a high resolution technique called ROMA, a form of comparative genomic hybridization, performed by a two-color assay of co-hybridization to a single oligonucleotide array. They found copy number variations (CNV's) in 20 % of the study group; they were *de novo* mutations. The study also included the parents, in a large percentage of whom the same CNV could not be found. We performed a similar study in our dysmorphic patients. This group of 50 children included: dysmorphic syndromes involving eye or renal malformations, dysmorphic syndromes with only skeletal abnormalities or with CNS malformations and mental retardation, and complex cases of autism in association with dysmorphia. A few of these cases were familial. For such cases, we utilized linkage studies using Affymetrix's 10K Mapping chips and also performed aCGH experiments on the affected family members using Agilent's 244K chips. For the non-familial cases, some of the parents were also included in the study in order to conclude that the observed CNVs in the patient are a *de novo* occurrence, a germline mutation. A large number of such changes were observed, some reported (http://projects.tcag.ca/variation/) and some unreported CNVs. This presentation will list those findings. The data are presented since we believe, such studies should contribute to our better understanding of dysmorphias and should lead to more basic studies on the genes or gene groups identified underlying the dysmorphic syndromes.

Kim, Jong-Won

Comparison Of SNP Genotyping With The Different Platforms

JONG-WON KIM, M.D. Ph.D.
Department of Laboratory Medicine and Genetics, Samsung Medical Center, School of Medicine, Sungkyunkwan University, Kangnam-gu, Seoul, Korea

Genotypic calls and consistencies were compared by competing SNP platforms. Overlapped genotypes produced by a GeneChip® custom 20K SNP kits (TG chip, Affymetrix, USA), Affymetrix Genechip Human Mapping 500K array sets (500K chips, Affymetrix, USA) and Humanhap 550 genotyping beadchip platforms (550K chip, Illumina, USA) on chromosome 2 and 7 region of 90 Korean samples were analyzed.

Overlapping 2,538 single-nucleotide polymorphisms (SNPs) among 500K chips and TG chips and 4,090 SNPs among 550K chips and TG chips, were compared. Genotype results from 500K chips and 550K chips common in the TG chips show the agreement of 94.3% and 95.8% respectively. When both platforms make a genotypic call, the agreement is a 98.6% and 99.4%. For the quality control (QC) genotypes, excluding variations less than 80% genotyped and deviated from HWE test, the rates of genotype agreement are 98.7% and 99.3%.

In conclusion, both 500K chips and 550K chips have high consistencies with TG chips.

Kojima, Toshio

Identification Of Genome Structural Variations From Signal Distribution Of Genotyping Array

KOJIMA, TOSHIO; Hasegawa, Aki; Konagaya, Akihiko
Genomic Sciences Center, RIKEN, Yokohama, Japan

Here, we describe a novel algorithm for identifying genome-wide copy-number variations based on a multi-chip approach using Affymetrix GeneChip Human Mapping 500K arrays. The algorithm comprises 2 parts: (1) elimination of the chip-to-chip nonbiological variation via normalization and (2) evaluation of the signal intensity distribution at the probe level and subsequent identification of genome-wide copy-number variations using various indexes obtained from the evaluation. We applied our algorithm to a publicly available dataset for which the genome-wide copy-number variations have already been analyzed and reported; we succeeded in identifying the genome-wide copy-number variations extremely efficiently. Our algorithm has certain advantages in comparison with other existing ones: (1) it is conceptually simple, robust, and easy to understand and (2) since it only uses the distribution of the signal intensities and is independent of certain platform-specific assumptions, it is also applicable to data obtained from platforms other than the Affymetrix 500K set.

Kok, Eloise

Association Of Genetic Variation Of CRP With Senile Plaques And Neurofibrillary Tangles Of Alzheimer's Disease

KOK E, Alanne M, Haikonen S, Luoto T, Huhtala H, Viiri L, Kristiansson K, Hurme M, Haapasalo H, Perola M, Karhunen PJ
School of Medicine and School of Public Health, University of Tampere, Finland
Tampere University Hospital, Finland. Department of Molecular Medicine, National Public Health Institute, Helsinki, Finland

Alzheimer's Disease (AD), the single most common form of dementia can currently only be definitively diagnosed post-mortem. Over the past two decades, many studies have shown that the apolipoprotein E (APOE) ε4 allele has an association with the disease (Kivipelto et al., 2002), although the exact mechanism is uncertain. Many recent studies have now suggested that inflammation plays a part in the disease (Block et al., 2007), although whether it causes, or is the result of the disease is still debated. C-reactive protein (CRP) is a common inflammatory molecule and a universal indicator of the presence of inflammation in tissues (Uchikado et al., 2004). Elevated CRP blood levels and certain haplotypes of the CRP gene associated with higher CRP production, have recently been shown to be involved in the increased mortality of patients (Hurme et al., in press). Recently, it was also shown that CRP is expressed in the pyramidal neurons of AD patients, and that CRP is up-regulated in affected areas of the AD brain (Yasojima et al 2000).

We investigated 603 men and women (aged 0-97 years) who died out-of-hospital in the Tampere region (Finland) during 2002-2004. The frequencies of senile plaques in the frontal cortex and neurofibrillary tangles in the hippocampus area were scored using a modified version of the CERAD protocol. All plaques were taken into account, irrespective of the neuropathological stage of the plaque (diffuse, neuritic). DNA was extracted from post-mortem blood samples using standard techniques and CRP genotyping of six different single nucleotide polymorphisms (SNPs) was performed utilising Sequenom's mass array system.

At least one senile plaque (SP) was found in 31.0 % of cases, with the plaque score varying between 0 and 5.41%. SP had a strong correlation with age ($r_s = 0.46$, $p < 0.001$). Neurofibrillary tangles (NFT) were found in 42.1% of all cases varying between 0 and 29.60 tangles/mm2. NFT count also had a strong relationship with age ($r_s = 0.52$, $p < 0.001$). In logistic regression analysis of the six SNPs (-717 A/G, -286 C/T/A, +1059 G/C, +1444 C/T, +1846 G/A, +4466 G/T) for the presence of SP with sex and age as covariates, showed that carriers of the T allele of the +4466 SNP had an elevated risk (OR 2.263, p=0.020), as well as G allele carriers of the -717 polymorphism (OR 1.636, p=0.056), along with age (p<0.0001). There were no associations between these SNPs and NFT, nor between the other SNPs studied and any of the neuropathological hallmarks of AD.

Variation in the CRP gene correlates with the presence of SP, most commonly associated with AD, supporting the theory that inflammation plays a role in the pathogenesis of the disease.

Kollin, Jussi

Imitating The Outcome Of Ascertainment Process For Coalescent Simulator Output

KOLLIN, JUSSI; Koivisto, Mikko; Mannila, Heikki
Helsinki Institute for Information Technology, Department of Computer Science, University of Helsinki, Finland

Whole-genome single nucleotide polymorphism (SNP) data can be used in search for population substructures with a score computed for chromosome segments. To evaluate the significance of the results, the sampling distribution of the score under the null hypothesis may have to be solved in order to compute the relevant p-values. Presenting this in an analytical form can be difficult, if the score is computed over a sequence of SNPs. Simulations can be used to form the empirical sampling distribution.

Genome-wide SNP data sets might use a complex ascertainment scheme to ensure maximal coverage over the genome. This poses a problem for correcting the ascertainment bias in the score statistics. Coalescent simulation produces SNP data without ascertainment bias. Assuming the simulator using best-fit parameters calibrated by Schaffner et al. [1] can be used to produce data with the same characteristics as the human genome, simulating ascertainment process means removing SNPs from the synthetic data to reproduce the same bias as the real data has. If the process utilizes notions such as genic regions not implicitly modeled in the simulator, straightforward simulation of the process is at best difficult.

We examine a method that aims to reproduce the effects of ascertainment process seen in extensive SNP data sets without knowledge on the ascertainment process. We evaluate the performance of the method by comparing the r^2 mean, minimum allele frequency (MAF) and SNP spacing histograms of the authentic data and the filtered simulation data, generated by the coalescent simulator calibrated by Schaffner et al. [1].

Our method selects for each synthetic segment a random segment from the authentic data to serve as a model. For each SNP in the authentic segment, we include in the filtered synthetic segment the best free match in the synthetic segment to minimize the error in physical distance and MAF.

From the authentic data set, we randomly sparsify too dense SNP regions, as this feature is unlikely to be properly reproduced in the synthetic data set at the same position. We also eliminate SNPs with low MAF from both data sets and SNPs that are near identical with another SNP within a fixed physical distance. For the simulation data we model genotyping error as random noise.

In the results, the mean r^2 of pairs of SNPs of the simulated European population after filtering is on the average 0.04 lower than for the CEU subset of Hapmap phase I data as a function of distance under 50kb. The MAF histograms follow each other moderately well, but the histogram of successive SNP distances replicates only a downward trend that is not a close match to the features present in the authentic data.

With Perlegen data, the fitted MAF and SNP spacing histograms of the simulation data follow the authentic data well. Unlike in the case of HapMap data, the three populations were joined together and handled as a single data set. The filtered simulated populations had lower mean r^2 with nearby SNPs (difference below 0.08 for each population separately), and at high distances higher mean r^2 (difference below 0.06 at the distance of 50kb).

In conclusion, fitting the MAF and spacing histograms alone did not guarantee equal r^2 patterns. With Hapmap CEU population alone as the model, LD levels were moderately well replicated in the filtered data. When simulating three populations simultaneously with Perlegen data, the levels were not as good a match. By tweaking the simulation parameters, we expect to fit also the LD levels better.

[1] Schaffner et al. Calibrating a coalescent simulation of human genome sequence variation. Genome Res., 15:1576--1583, 2005.

Korbel, Jan

Charting And Sequencing Structural Variation Using High-Resolution Paired-End Mapping (HR-PEM)

KORBEL, JAN; Urban, Alexander Eckehart; Affourtit, Jason; Grubert, Fabian; Kim, Philip; Du, Lei; Carriero, Nicholas; Godwin, Brian; Turcotte, Cynthia; He, Wen; Taillon, Bruce; Simons, Jan; Kidd, Kenneth, Carter, Nigel; Hurles, Matthew; Harkins, Tim; Weissman, Sherman; Gerstein, Mark; Egholm, Michael; Snyder, Michael
Yale University, New Haven, CT; 454 Life Sciences, Branford, CT; The Wellcome Trust Sanger Institute, Hinxton, Cambridge, UK; Roche Applied Science, Indianapolis, IN

Structural variants (SV), i.e. deletions, duplications, insertions, and inversions involving kilo- to Megabases of genomic DNA, were recently suggested to be responsible for a considerable amount of phenotype variation, and possibly, disease in humans (1-6). However, to date, most methods for identifying structural variants have resolutions in the order of 50-75 kb (7), and thus do not precisely identify the boundary sequences (i.e. breakpoints) of SVs. Furthermore, the majority of approaches used so far for cataloging SVs in the human genome do not detect copy-number neutral variation events such as inversions and balanced translocations.

We present a novel approach, High-Resolution Paired-End Mapping (HR-PEM), which makes use of 454/Roche sequencing technology, and combines computational analysis, high-throughput PCR assays, and amplicon-cocktail-sequencing to rapidly identify SVs at high resolution, and subsequently sequence across the breakpoints associated with these variants. The approach involves sequencing the ends of circularized 3 kb genomic fragments and mapping them onto the human genome reference sequence. The resolution of breakpoint assignments is d3 kb and thus well-suited for PCR validation. We have used HR-PEM to map and sequence SVs i.e. simple deletions, insertions, and inversions, as well as more complex structural rearrangements in two individuals in order to generate a precise map of SVs and their associated breakpoints. From 21 million and 10 million paired-end sequences, respectively, from each individual, several hundred SVs have been predicted so far, ranging from 2 kb to several Mb in size. A first pass PCR analysis indicates that at least 60% of the predicted SVs can be amplified in a single PCR band and analyzed using DNA sequencing. Our results reveal as yet unexplored aspects of structural variation in the human genome, and suggest mechanisms by which this layer of genomic variation has arisen.

References

1. Sebat et al. (2004) Science 305, 525-8.
2. Iafrate et al. Nat Genet 36, 949-51.
3. Tuzun et al. Nat Genet 37, 727-32.
4. Redon et al. (2006) Nature 444, 444-54.
5. Gonzalez et al. (2005) Science 307, 1434-40.
6. Stranger et al. (2007) Science 315, 848-53.
7. Coe et al. (2007) Genomics 89, 647-53.

Kramer, Patricia

Identifying Genetic Associations With Alzheimer-Free Survival

KRAMER, PATRICIA (1); Kaye, Jeffrey (1); Ott, Jurg (2)
(1) Oregon Health and Science University, Portland Oregon; (2) Rockefeller University, New York, NY

Genetic and environmental mechanisms control the timing of dementia with aging. The genetic correlates of survival to advanced age without Alzheimer s disease (AD), although largely unknown, have become a tractable target for discovery. Most cases of AD occur after 65 years of age. They do not follow simple patterns of inheritance, suggesting more complex etiologies. Apolipoprotein-E until recently has been the only consistently confirmed susceptibility gene for late-onset AD.

It is not uncommon to find individuals of advanced age who show no clinical dementia at death but have substantial AD pathology in the form of neuritic plaques and/or neurofibrillary tangles at autopsy. According to cognitive reserve theory, individuals differ in their capacity to maintain normative cognitive function; subsequently, individuals with greater capacity are better equipped to delay or circumvent the damaging effects of brain lesions that, in other less equipped individuals, lead to clinical manifestations of dementia and AD.

We are conducting a genome-wide SNP and copy number variant (CNV) association study, using a large sample of well-characterized AD subjects and elderly non-demented subjects, to identify genetic factors that influence cognitive reserve. We have over 700 subjects, collected prospectively at ten collaborating National Institute on Aging AD Centers, who fulfill criteria for the study. These include 300 AD subjects who entered programs non-demented and converted to AD prior to death, had at least two clinical evaluations, autopsy-confirmed AD and DNA; and 450 subjects who entered the program non-demented and remained free of clinical AD symptoms at death, had at least two evaluations, an autopsy and DNA. All were Caucasian in order to limit potential effects of ethnic heterogeneity. Each subject sample will be genotyped at deCODE Genetics using the Illumina Human CNV370 Duo Bead Chip, which contains 317,000 SNP loci and 56,000 features for CNV detection. Genotyping will be completed in July 2007.

Our first aim is to identify genetic variants related to delay in age at onset of AD, as one manifestation of cognitive reserve. We will utilize all demented subjects and an equal number of non-demented subjects and conduct a case-control association study. Our second aim is to identify variations associated with maintenance of cognitive resilience in the presence of neuropathological damage, as an alternate expression of cognitive reserve. We will utilize all non-demented subjects, divided into two categories of neuropathological status (low or high) representing the likelihood of pathological processes leading to AD. We will then conduct a second case-control association study.

To evaluate each SNP for association, we will conduct a genotype test and an allele test. P-values < 10-7 will be considered significant. To identify those tests with a false discovery rate, FDR<0.05, all tests will be assessed with q-values (http://faculty.washington.edu/jstorey/qvalue/). If one or more SNPs exhibit significant association to disease, we will look for gene-gene interaction effects between such a SNP and all other SNPs, using likelihood ratio tests. Joint association effects of multiple SNPs will be assessed with set association analysis (http://www.genemapping.cn/sumstat.html).

We will present a descriptive profile of the study sample, including data on demographic, clinical, co-morbidity and neuropathologic characteristics. These data illustrate the complexity of the human aging phenotype, and the challenges posed to studies of genetic mechanisms involved in the maintenance of cognitive function.

Kryukov, Gregory

Low Frequency Missense SNPs: Are They Informative For Human Genetic Studies?

KRYUKOV, GREGORY; Sunyaev, Shamil
Division of Genetics, Department of Medicine, Brigham and Women's Hospital, Harvard Medical School, Boston, MA 02125, USA

Several recent reports showed that common complex phenotypes can be caused by multiple rare non-synonymous variants, and proposed association studies based on complete re-sequencing of candidate genes. In a study of this design, a cumulative frequency of rare deleterious mutations in a candidate gene, rather than individual SNPs frequencies, is compared between disease and control cohorts. The success of such approach critically depends on the proportion of deleterious mutations among all detected missense polymorphisms and on our ability to distinguish deleterious amino acid substitutions from neutral ones. If the majority of amino acid substituions detected in the study are neutral, then, the power of the method will be low because of the low signal to noise ratio.

It was not known what fraction of missense substitutions among *de novo* mutations and polymorphisms are strongly detrimental, mildly deleterious or effectively neutral. We estimated these values by comparing expected and observed numbers of nonsense, missense and synonymous changes among disease mutations, human SNPs identified by systematic re-sequencing projects and substitutions fixed in the human lineage after divergence from chimpanzee. As expected, fraction of deleterious mutations among common polymorphism was extremely low. However, despite commonly held belief that even among rare missense SNPs most are effectively neutral, our results indicate that the majority of human missense polymorphisms with detected frequency below 1% are, in fact, deleterious. This suggests that allele frequency alone can serve as a strong predictor of functional significance of missense polymorphic variants. We estimated that, on average, each human genome has approximately 600 moderately deleterious missense SNPs associated with selection coefficients in the range of 10^{-2}-10^{-3}.

Our work serves as a theoretical foundation to association studies aimed at detection of rare missense mutations enrichment. To test the feasibility of scaling-up this approach to a genome-wide level, we performed forward population simulation to predict spectrum of missense SNPs in the human population, including very low frequency tail of the distribution. We, then, computationally simulated outcomes of re-sequencing studies of various designs. Given the emergence of new cost-effective DNA sequencing technologies, abundance of low frequency polymorphism in the human population and favorable functional-to-neutral ratio among rare missense SNPs, genome-wide Mutation Excess Sequencing Studies (MESS) might become a viable approach to study genetics of complex diseases in a foreseeable future.

Laan, Maris

Genetic Component Of Human Blood Pressure Targeted By 500,000 SNPs

Elin Org(1); Susana Eyheramendy(2); Christian Gieger(2); Peter Lichtner(3); Siim Sõber(1); Thomas Illig(2); Erich Wichmann(2); Thomas Meitinger(3) and MARIS LAAN(1)
(1) Institute of Molecular and Cell Biology, University of Tartu, Estonia; (2) Institute of Epidemology and 3Institute of Human Genetics, GSF National Research Center of Environment and Health, Neuherberg, Germany

Blood pressure (BP) measured in two components, systolic and diastolic BP (SBP, DBP), is a quantitative human trait dependent on individual s sex, age, lifestyle, BMI, prevalence of different diseases (e.g. diabetes), circadian rhythm (lower at night), physiological condition and activity (e.g. difference in BP in standing, sitting, walking and running) etc. Although nowadays BP is easy and standardized to measure, it truly represents a complex trait determined for a test subject at a certain time-point by the combination of genetic heterogeneity with environmental factors. Thus, mapping the genetic component of BP requires a well-phenotyped and -described genetically homogenous cohort, repeated (over time) BP measurements in standardized conditions and high-resolution genetic test system allowing targeting and testing multiple loci simultaneously. A condition of elevated blood pressure without any obvious cause is called essential hypertension (EH). It is the most common cardiovascular disease, with a prevalence of nearly 27% worldwide, and a major risk factor for stroke, heart disease and end-stage renal disease. Based on twin studies, the genetic component of essential hypertension has been estimated to be only 30-60%. The population-based association studies with currently recognized candidate genes have shown inconsistent results: ~150 loci have been listed and tested as potential BP affecting and/or EH susceptibility genes. Followed by the past success of whole-genome linkage studies for rare familial hyper- and hypotension, we have turned to the genome-wide association (GWA) study to address the genetic component of blood pressure traits (SBP, DBP and EH) in general population. We have performed a GWA for blood pressure traits using Affymetrix 500K SNP platform in the framework of KORA 500K consortium. KORA represents a genetically homogenous population cohort sample from Augsburg region in Southern Germany, which has been targeted by several epidemiological studies over years (Wichmann et al.2005; Gesundheitswesen 6 (Suppl): S26-S30). The individuals entering analysis aged 35-69 and had BMI<30. For each participating individual the BP has been measured twice, in the beginning of collection and then during the follow-up study 10 years later, which allows to decrease phenotypic heterogeneity and to verify the diagnosis. SNPs entering the statistical analysis had call rate >93%, MAF>1% and were in HWE (p>0.001). Both, case-control (severe EH as a trait, n=364/596) and quantitative (SBP& DBP, n=1017) association analysis were performed using different genetic models. Consistent with the genetic heterogeneity of blood pressure determination, no single locus appeared as the major contributor to the trait. The strongest association (P value <10-6) was detected with markers on chromosomes 1, 2, 5, 6, 10 and 16. Chr 1, 2, 6 and 16 contain multiple QTLs identified in linkege studies for blood pressure traits (Samani, 2003; AJH 16: 167-171). The landscape of most significant (p<5x10-5) associations yielded several novel potential candidates for blood pressure traits. Interestingly, several (ca 19%) top associated SNPs were localized in CNV (copy number variant) regions in human genome. Whether these are true associations or outcomes of biased statistical tests, still needs to be determined. The validation experiments for the most significantly associated SNPs from GWA located in unique genomic regions are currently under way.

Laayouni, Hafid

Measuring Spanish Population Structure And Selection Of Markers For Stratification Detection Using Genomic DNA Pooling And Whole Genome Scan Analysis

HAFID LAAYOUNI, Francesc Calafell, and Jaume Bertranpetit
Unitat de Biologia Evolutiva, Universitat Pompeu Fabra, Barcelona, Spain

Population structure affects the sampling process and thereby both the validity and power of association studies. In a structured population, allele frequencies will vary geographically and as cases and controls may be drawn from different locations, their allele frequencies will also tend to differ, leading to false positive association tests. To assess the population structure extent in the Spanish DNA Bank (a common resource for scientific community for use in forthcoming association studies), we estimated the allele frequency differences between different Spanish populations by Whole Genome genotyping analysis using illumina 317K arrays. Genotyping was performed using DNA pools constructed using about 30 individuals with well-established ancestry. Population differentiation assessed by FST statistics was very low (mean 0.025 and 95% of SNPs show an FST value less than 0.05). Low level of population structure is detected among peninsula population including the Basque country population and Balearic Island, however Canary Island show high potential of causing stratification. Principal component analysis performed using the top 1000 highest SNPs shows that 25% of variation separates Canary Island from the rest of the Spanish populations. Highly informative SNPs of population stratification detected in this first phase of the analysis will be subsequently genotyped in the complete Spanish DNA Bank to ensure the availability of appropriately matched controls for association studies through the genetic knowledge of each individual. Data are discussed in the context of population Genetics and association studies. This study provides a more comprehensive picture of the genetic composition in relation to geographic variation of the Spanish population.

Laderman, Stephen

High-Resolution Structure Of Human Copy Number Variant (CNV) Regions As Defined By Custom High-Density Oligonucleotide Microarrays

Amir Ben-Dor(1), George H. Perry(2), Alicia Scheffer(1), Nick Sampas(1), Stephanie Dallaire(2), Joelle Tchinda(2,3), Anya Tsalenko(1), Peter Tsang(1), Alice Yamada(1), Zohar Yakhini(1), Laurakay Bruhn(1), STEPHEN LADERMAN(1), Charles Lee(2,3)
(1) Agilent Technologies, Santa Clara, CA, USA; (2) Department of Pathology, Brigham and Women's Hospital, Boston, Massachusetts, USA; (3) Harvard Medical School, Boston, Massachusetts, USA

Copy Number Variants (CNVs) are highly prevalent in the human genome and may play an integral role in determining the diversity of human traits, including susceptibility to disease. Initial screens using a variety of technologies have identified thousands of CNV regions. However, many of these measurements provide limited resolution of the structure of the CNV regions, including their sizes and boundaries. To further explore the architecture of known CNVs, we have constructed a custom oligonucleotide array set containing 470,143 distinct 60mer probes, encompassing 2191 human CNV regions, annotated in the Database of Genomic Variants [http://www.tcag.ca/] as of November 30, 2006. The probes were selected with an approximate spacing of 1 kb within putative CNV regions and within 5 kb upstream and downstream of each CNV region. Probe density was progressively reduced for up to an additional 15 kb of flanking sequences.

Our initial study interrogated the genomic DNAs from 30 individuals of the 270 HapMap samples. To facilitate comparisons between studies, we used the same reference sample (i.e. the NA10851 HapMap individual) as in the Redon et al. study (Nature 444: 444-454, 2006). Across these 30 HapMap individuals, the number of CNV regions within which our array set detected variants ranged from 163 to 258. 93 CNV regions were identified in the Redon et al. study by both array platforms used there in at least one individual, and designated as CNVs of high confidence. With our custom oligonucleotide array platform, we observed copy number variation in all but one of these regions, demonstrating the robustness of our array set for detecting CNVs. Across the regions studied, we frequently observed CNVs with sizes that varied considerably among the 30 individuals, whereas the CNV boundaries (breakpoints) remained consistent between replicates of the same sample. This implies a richer structure of variation in many human CNV regions than previously reported.

These data improve our understanding of human CNV architecture and precede the design of a targeted, CNV-enriched array that may be useful as a tool in disease association studies aimed at identifying the role of CNVs in common diseases.

Laframboise, Thomas

SNP Allelic Inference In The Presence Of Germline Duplications And Deletions

Kothari, Moulik; Gould, Meetha; Macconaill, Laura; LAFRAMBOISE, THOMAS
Case Western Reserve University School of Medicine, Cleveland, OH
The Broad Institute of Harvard and MIT, Boston, MA

Genotyping single nucleotide polymorphisms (SNPs) in humans using microarrays has become increasingly high-throughput and cost-effective. The methods associated with these arrays generally assume two copies of each SNP per cell, for a diallelic genotype. Given the recent discovery of widespread copy number variation in the human genome, however, this assumption is no longer always valid. For example, if an A/G SNP is contained within a genomic region that is duplicated in a significant proportion of the human population, this SNP's genotype may be AAA, AAG, AGG, or GGG for some individuals. This 'generalized genotype' is unrestricted by the usual diallelic assumption that results solely in AA, AG, and GG genotypes, and succinctly provides both copy number and SNP allelic information. Indeed, besides these possible genotypes in a region of duplication, an individual may carry an A- or G- genotype in a region harboring a germline deletion, or even a "--"genotype if chromosomes harboring the deletion are inherited from both parents. Currently-available software would typically call an AAG genotype as AA or AG, an A- genotype as AA, and a -- genotype as "No Call". These erroneous calls can lead to incorrect phasing and apparent deviations from Hardy-Weinberg equilibrium. Moreover, the impending growth in genome-wide association studies will heavily rely on accurate SNP genotyping, whether the SNPs are used as markers or as putative causal variants.

We have developed methods to infer generalized genotypes from both the Affymetrix GeneChip and the Illumina BeadChip Arrays. Each of these platforms interrogates over 500,000 human SNPs across the genome. Our methods were developed using data from over 100 HapMap samples on each platform, enabling us to compare inferences from these independent technologies. Many of our generalized genotypes were verified using a variety of "wet lab" assays, and demonstrated a high level of accuracy for our *in silico* approach for both the Affymetrix and the Illumina platforms.

Phasing the generalized genotypes in regions of duplication is subject to the same difficulties as in the diallelic SNP setting. The mother-father-child trios in the HapMap data set allow us to determine phase unambiguously in thousands of cases, and the interrelationship between SNP allele and copy number variation provides insight into the history of the point mutation and duplication events that resulted in these variants. Our analysis of thousands of duplicated SNPs implies that the duplication is more recent than the point mutation in most, if not all, cases. Furthermore, the duplication events seem to be recurrent in human history in many cases.

Lange, Christoph

Replication Of The Association Of The ROBO1 Gene With Obesity: Timing Is Everything!

CHRISTOPH LANGE (1,2), Jessica Lasky-Su (2,3) Multiple collaborators from the following organizations: Framingham Heart Study, KORA-S4, Decode, Merck, Harvard Medical School, Children s Hospital, Channing Laboratories, the Broad Institute
(1) Harvard School of Public Health, Boston, MA; (2) Channing Laboratory, Brigham and Women's Hospital, Harvard Medical School, Boston, MA; (3) SUNY Upstate Medical University, Syracuse, NY

While the failure to replicate genetic association is most commonly believed to be attributable to insufficient statistical power (e.g. in false positives in an initial sample or false negatives in a replication study), population stratification, or various forms of between-study heterogeneity or environmental influences, we illustrate that age-dependence of the genetic association can also be a potential reason for non-replications. Using the 100K SNP scan of the Framingham Heart Study, we identify an age-dependent genome-wide association between a SNP in ROBO1 and obesity. This finding was followed-up in seven independent samples comprised of 12,838 genotyped probands. Overall, the original finding replicates in five of the seven studies (six of the eight total studies), showing an age-dependent relationship (one-sided combined p = 8.42x10-10). Furthermore, this study illustrates that commonly used genetic association designs, ascertainment conditions and analytical methods may not be able to detect age-dependent associations. If age/time-dependent genetic effects are not taken into account in the selection of follow-up samples, ascertainment conditions and in the statistical analysis, important genetic associations may be missed.

Lee, Charles

Evolutionary Population Genetics Of RHD Copy Number Variation And The Human RhD Negative Blood Type

CHARLES LEE(1,2), Arthur S. Lee(1), Yali Xue(3), Steven A. McCarroll(4,5), Pardis C. Sabeti(5), J. Omar Yanez(1), Anne C. Stone(6), Chris Tyler-Smith(3), and George H. Perry(1,6)
(1) Department of Pathology, Brigham and Women.s Hospital, Boston, MA, USA; (2) Harvard Medical School, Boston, MA, USA; (3) The Wellcome Trust Sanger Institute, The Wellcome Trust Genome Campus, Hinxton, Cambridge, UK; (4) Department of Molecular Biology, Massachusetts General Hospital, Boston, MA, USA; (5) Program in Medical and Population Genetics, Broad Institute of Harvard and Massachusetts Institute of Technology, Cambridge, MA, USA; (6) School of Human Evolution and Social Change, Arizona State University, Tempe, AZ, USA

Because of its clinical significance, the Rh blood group system is one of the most widely studied human phenotypes. Homozygous deletion of the entire RHD gene results in an RhD negative (-) blood type. RhD- mothers produce anti-RhD antibodies in response after exposure to blood from an RhD positive (+) fetus during pregnancy or childbirth. In such cases, subsequent RhD+ offspring may be affected by hemolytic disease of the newborn, having an adverse affect on the mother's reproductive fitness. Therefore, one might expect strong purifying selection to act against the RhD- phenotype. However, the RHD deletion allele is found at a high frequency in many European populations, as high as 60% in certain populations. Therefore, we hypothesized that the RHD deletion allele may confer some unknown fitness benefit, such that positive or balancing selection may explain this unexpected high frequency.

To evaluate this hypothesis, we have characterized patterns of linkage disequilibrium around the RHD gene by genotyping the deletion in the 270 HapMap individuals representing four populations, for whom genotypes for >3 million genome-wide single nucleotide polymorphisms (SNPs) have been obtained. We observed an RHD deletion allele frequency of 0.43 in the European-Americans, 0.19 in the Yoruba, and 0.06 among the Asian samples (Japanese + Chinese). Interestingly, we found a low frequency RHD duplication allele in the Yoruba and Asia samples. This was confirmed by extensive fiber FISH analyses. When RHD allele-specific copy number information (i.e., deletion versus no deletion) was integrated with phased SNP data, we observed high levels of linkage disequilibrium between the deletion allele and SNPs up to 200 kb upstream from the gene in European-Americans. This extensive linkage disequilibrium could reflect a recent and rapid frequency increase, which would be a signature of positive selection. We are currently resequencing flanking regions of the RHD gene in individuals from the four HapMap populations in order to more fully characterize the evolutionary history of this deletion.

Li, Ling-Hui

Genome-Wide SNP Scan Coupled With Copy Number Estimation And Allelic Imbalance Analysis Detects Submicroscopic Chromosomal Aberrations In Acute Lymphoblastic Leukemia

LING-HUI LI(1), Hsin-Chou Yang(2), Tzu-Po Chuang(1), Chun-Yu Wei(1), Sheng-Feng Ho(1), You-Chin Lin(3), Mitchell Diccianni(4), Yann-Jang Chen(5), Chien-Hsiun Chen(1), Kuo-Ching Jiang(1), Cathy S. J. Fann(1), Jer-Yuarn Wu(1), Yuan-Tsong Chen(1), Alice Lin-Tsing Yu(3,4)
(1) Institute of Biomedical Sciences, Academia Sinica, Taipei, Taiwan; (2) Institute of Statistical Science, Academia Sinica, Taipei, Taiwan; (3) Genomics Research Center, Academia Sinica, Taipei, Taiwan; (4) Department of Pediatrics/Hematology Oncology, University of California, San Diego, California, USA; (5) Life Sciences and Institute of Genome Sciences, National Yang-Ming University, Taiwan

Background: Cytogenetic analysis of childhood acute lymphoblastic leukemia (ALL) is difficult due to the low mitotic index and poor quality of the metaphases; this is especially true for T-ALL as limited chromosomal abnormalities are observed in only 39-75 % of T-ALL. Our purpose was to utilize high-density SNP array to investigate submicroscopic chromosomal aberrations in childhood ALL.

Methods: Fourteen ALL patients including 12 T-ALL and 2 B-ALL were subjected to array-based SNP genotyping. An analysis paradigm which combined copy number estimation and a new algorithm for allelic imbalance analysis was applied to genotyping data to detect submicroscopic chromosomal abnormalities and allelic alterations. The chromosomal aberrations were verified using real-time PCR and fluorescence in-situ hybridization.

Results: A total of 60 non-redundant cancer-specific regions with deletion or amplification were identified; among them, 10 regions were between 1 to 4 Mb, and 22 regions were d 1 Mb. Importantly, 9 regions in the 12 T-ALL samples were novel as they have never been reported in T-ALL, including amplification of the MYCN locus and deletion of the RUNX1 (AML1) locus. In addition, we observed frequent deletion of ~1 Mb within 14q11.2/TCRA locus, a deletion interval of 1.0 Mb within 7q34/TCRB locus and deletion or loss of heterozygosity (LOH) involving 9p21, where the CDKN2A and CDKN2B genes are located. With allelic imbalance analysis involved alleles were assigned, complex deletions and amplifications that might reflect clonal heterogeneity or cancer evolution were identified, and copy-neutral LOH was detected.

Conclusions: The results demonstrated that high-density genome-wide SNP genotyping coupled to copy number estimation and allelic imbalance analysis is useful for detecting submicroscopic chromosomal aberrations and allelic alterations in heterogeneous cancer samples and for defining the boundaries of chromosomal abnormalities and involved alleles. This will facilitate the identification of candidate genes involved in leukaemogenesis. Furthermore, the dissection of tumor heterogeneity at the molecular level will provide valuable insight into the complex process of tumor evolution and therapeutic targeting.

Lin, Yung-Feng

Mapping Tumor Suppressor Gene For Hepatocellular Carcinoma By Allele Retention Status

YUNG-FENG LIN(1,2), Ling-Hui Li(2), Jian-Chiuan Li(2), Mei-Hua Tsou(3), Ming-Tai Chuang(1), Tsai-Lien Liao(2), Ya-Hui Chang(2), Chih-Hung Li(2), Shiu-Feng Huang(2), Shih-Feng Tsai(1),(2)
(1) Institute of Genetics and Genome Research Center, National Yang-Ming University, Taipei (112), Taiwan; (2) Division of Molecular and Genomic Medicine, National Health Research Institutes, Zhunan, Miaoli 350, Taiwan; (3) Department of Pathology, Koo Fundation Sun Yat-Sen Cancer Center, Taipei (112), Taiwan

Hepatocellular carcinoma (HCC), the most common liver malignancy, is characterized by various genomic alterations at multiple chromosomal locations. Precise mapping of tumor-associated genes by loss of heterozygosity (LOH) analysis, hampered by high degree of genomic instability and variable changes of chromosomal DNA in HCC, has not been successful. We have taken a new approach by comparing single nucleotide polymorphism (SNP) between control and tumor tissues and systematically examined genes in the chromosome 4q21-25 region for haplotypes selectively retained in the tumors. From 144 genes in 4q21-25, UNC5C and DKK2 showed nonrandom distribution of SNP haplotypes in HCC. We demonstrated that DKK2 protein can act as tumor suppressor by antagonizing the WNT signaling pathway. Taking together the previously reported tumor suppressor function of UNC5C, we conclude that allele retention status can be used as an indicator for tumor suppressor gene mapping.

Lopez-Ridaura, Ruy

Genetic Variants In The Apo A1/C3/A4/A5 Gene Cluster Are Associated With Adiponectin Levels In Diabetic Men Independently Of The Lipid Profile

RUY LOPEZ-RIDAURA, Cuilin Zhang, Eric B. Rimm, Nader Rifai, Frank B. Hu
Cuernavaca, Mexico, Boston, MA

Background and Aims: Several studies showed genetic variations of Apo A1/C3/A4/A5 gene cluster associated with hyperlipidemia and atherosclerosis in the general and diabetic population. However, some findings suggest triglycerides or HDL levels did not explain the entire risk associated with these genetic variations. We aimed to explore the association of these genetic variants and other potential mediator of cardiovascular risk among diabetic men.

Methods: After 14 years of follow-up of men aged 45-70 y who participated in the Health Professionals Follow-up Study in 1986, we identified 765 diabetic men who returned blood samples by 1994 and were free of any cardiovascular condition at blood draw. We evaluated the association between 10 common single nucleotide polymorphisms (SNPs) in the Apo A1/C3/A4/A5 gene cluster and biomarker levels including adiponectin and inflammatory biomarkers.

Results: Only three out of the 10 genotyped SNP within the Apo A1/C3/A4/A5 cluster, two SNPs in the regulatory region of APOC3 gene (C_482 and C3_455) and one of the non synonymous SNPs in the ApoA4 gene (A4_347), were significantly associated with adiponectin levels, whereas inflammatory biomarkers were not associated with any of the SNPs in the cluster. Al minor alleles either as heterozygous or homozygous were associated with lower levels of adiponectin and HDL and tended to be associated with higher levels of fasting triglycerides. After adjusting for HDL and Triglycerides, only the APO4_347 SNP persisted significantly associated with adiponectin levels. Specifically the minor allele in the APO4_347 SNP (Serine instead of Threonin) present a 10 percent decrement in the adiponectin levels as compared with homozygous for the most common allele. Haplotype analyses suggest confirm the association with the APO4_347 SNP to be independent of other SNPs in the cluster.

Conclusions: Genetic variation at the apoA4 gene may affect adiponectin levels independently of other lipid abnormalities among men with type 2 diabetes.

Lorente, Belén

Tools For Automatic/Massive SNP Selecting

LORENTE,BELEN (1); Medina,Ignacio (3); Sangrós,Ricardo (1.6); Alegre,Josep (1.6); Morcillo,Carlos (1.6); Pita, Guillermo (5); Vellalta,Gemma (4); Malats,Nuria (4); Gonzalez-Pisano,David (5,6); Dopazo,Joaquín (3,6); Navarro,Arcadi (1,2,6)
(1) Unitat de Biologia Evolutiva. Departament de Ciències Experimentals i de la Salut. Universitat Pompeu Fabra. Barcelona, Catalonia, Spain; (2) Institució Catalana de Recerca i Estudis Avancats (ICREA) and Unitat de Biologia Evolutiva. Universitat Pompeu Fabra. Barcelona, Catalonia, Spain; (3) Centro de Investigación Príncipe Felipe (CIPF), Valencia,Spain; (4) Instituto Municipal de Investigaciones Médicas (IMIM), Barcelona, Spain; (5) Centro Nacional de Investigaciones Oncológicas (CNIO), Spain; (6) Instituto Nacional de Bioinformática (INB), Spain

Researchers studying the genomic basis of complex diseases or traits need to select optimal sets of Single Nucleotide Polymorphisms (SNPs) from the millions that have been validated in the human genome. This constitutes a formidable computational problem, since many different criteria have to be considered simultaneously. These criteria include functional properties of SNPs (such as whether they affect aminoacids, transcription factor binding sites or microRNAs); population-based properties of SNPs (such as whether they are tag-SNPs in the population from which samples come from), as well as other properties that vary depending on the genotyping technology (sets of SNPs that can be efficiently genotyped with a given platform may present high failure rates in others).

SYSNPs (Select Your SNPs) is the first web server implementing algorithms that allow for efficient and simultaneous consideration of all the relevant criteria in order to obtain optimal sets of SNPs. SYSNPs allows users to select SNPs for arbitrarily large sets of genes or genomic regions. Also, users can easily consider technological information and tagging information from their choice populations.

A preliminary version of SYSNPs has been successfully used to select SNPs in different studies, thus optimizing reducing selection and genotyping costs. For example, the time needed to select appropriate tag-SNPs to study a set of 100 genes that will be genotyped by means of the Illumina platform is reduced from weeks to a few minutes. In addition, the task is simplified from a partially automated search in several databases by means of several different (and very limited) tools to the easy use of a simple browser.

Lundmark, Per

Utility Of HapMap Data In European Populations

PER LUNDMARK(1), Ulrika Liljedahl(1), Dorret Boomsma(2), Heikki Mannila(3), Nick Martin(4), Aarno Palotie(5), Leena Peltonen(6,7), Markus Perola(6), Tim Spector(8), David Leon(9), Ann-Christine Syvänen(1)
(1) Department of Medical Sciences, Uppsala University, Uppsala, Sweden; (2) Department of Biological Psychology, Vrije Universiteit, Amsterdam, Netherlands; (3) HIIT Basic Research Unit, University of Helsinki, Helsinki, Finland; (4) Genetic Epidemiology Unit, Queensland Institute of Medical Research, Brisbane, Australia; (5) Finnish Genome Center, University of Helsinki, Helsinki, Finland; (6) Department of Molecular Medicine, National Public Health Institute, Helsinki, Finland; (7) Department of Medical Genetics, University of Helsinki, Helsinki, Finland; (8) Twin Research and Genetic Epidemiology Unit, St. Thomas's Hospital, London, UK; (9) London School of Hygiene and Tropical Medicine, London, UK.

Here we describe a study performed to evaluate how well the European CEPH samples used in the HapMap project represent European populations from different countries. The nuclear family samples included in the study originated from Swedish, Finnish, Dutch, British and Australian populations (with European ancestry). The number of samples from each population was similar to those in the HapMap sample sets. A region spanning 1.5 M base pairs on chromosome 4 was selected for genotyping. A panel of 186 SNPs with available HapMap genotype data was genotyped using the Beckman Coulter GenomeLab SNPStream system. Allele frequencies were compared for all combinations of samples including the HapMap CEU European data. Principal component analysis was used to create an overview of clustering of different populations regarding allele frequencies, as well as the markers responsible for observed variance. The only sample with detectable differences in overall allele frequencies was that from Kuusamo, Finland. This sample also separated from the others, including the other Finnish sample, in the PCA analysis.

A tagSNP set was defined based on the HapMap data and applied to our samples. Performance of the tags was good in all samples with results ranging from 95% of markers captured at r^2 0.8 in the Kuusamo sample, to 87% captured in the Australian sample. The excellent performance of the Kuusamo sample despite allele frequency differences was in part explained by the low number of native tags required to tag Kuusamo. The Kuusamo, HapMap and Australian samples needed 58, 63 and 74 native tags respectively.

In conclusion the HapMap CEU sample appears representative for the European samples investigated for the purpose of tagSNP selection, with some caution regarding estimation of allele frequencies in the Kuusamo Finnish sample and a slight reduction in tagging efficiency in the Australian sample.

The study is supported by the GenomeEUtwin project (QLG2-CT-2002-01254)

Makeev, Vsevolod

Relationship Of Micro- And Minisatellites In The Human Genome

Boeva, Valentina; Fridman, Marina; Regnier, Mireille; MAKEEV, VSEVOLOD
Ecole Polytechnique, France; GosNIIgenetika, Moscow, Russia; INRIA Rocquencourt, France;
Institute of Molecular Biology, Moscow, Russia

Micro- and minisatellites constitute an essential part of DNA with low sequence complexity and perform a number of important functions. We used the TandemSWAN program to search the human genome for tandem repeats with a length of a repeated unit to 200 bp, including repeats with a large number of nucleotide substitutions. Our goal was to get distributions of micro- and minisatellites along human chromosomes, to analyze their properties and to reveal some characteristic features of sets of minisatellite families in each chromosome.

We compared distribution of the minisatellites in the annotation of the human genome [version 17, provided by the Human Genome Browser at UCSC] with that obtained by the TandemSWAN program. It turned out that a significant part of minisatellites with periods from 25 to 200 bp, about 35%, were not previously masked as repeats. Thus, one of the results of our work was the creation of a more complete map of minisatellites in the human genome.

In addition to some well known minisatellites families, e.g. Alu repeats or the annotated 48 bp repeat family in the 5' subtelomeric region on chromosome 22, we have discovered several new families, such as 38 bp family on chromosome 19.

We revealed some characteristic features of repetitive structures in other eukaryotic genomes. In conclusion it can be said that each of explored genomes has its distinctive tandem repeat distribution. Such evolutionary close genomes as genomes of mouse and rat can dramatically vary in the distribution of minisatellites. This may be the result of the balance between minisatellites proliferation and their elimination due to selection on the genome compactness.

Exploring minisatellites in human genome we came out with an observation that, generally, the presence or absence of characteristics satellites in a given long region of a DNA sequence can be used to determine whether or not this sequence belongs to a given chromosome. It was also found that the density of minisatellite repeats in human sex chromosome X is higher than that in human autosomes. The total database of the found tandem repeats is available at http://www.bioinform.genetika.ru/.

At the next step, we analyzed the distributions of microsatellites and short (with a period to 23 bp) minisatellites in human chromosomes. Most minisatellites found had period lengths multiples of 4. We assume that this period distribution is explained by the origin of these minisatellite repeats from microsatellite repeats. The process can be the following. A microsatellite elongates by, e.g., replication slippage. This results in the formation of a microsatellite repeat with a large number of copies. In the sequence of this repeat, there are point substitutions, insertions, or deletions of nucleotides, which make the microsatellite incapable of self-elongation by replication slippage. Further, the repeat or its part multiplies (elongates) as a minisatellite by the minisatellite mechanism, e.g., unequal crossing over. Finally, there is a minisatellite for which one can determine a shorter sub-period with a more degenerate repeated motif.

To test this assumption, we made an additional search for shorter sub-periods in the tandem repeats that had already been found by the program. It turned out that most of the found minisatellites with periods up to 23 bp can be regarded as derived from microsatellites. In a considerable part of the found repeats with periods that are multiples of 4, a repeated motif of a length of 4 bp can be discerned. It is also of interest that, in the human genome, words that most often form tandem repeats constitute a rather limited set.

Martinez, Urko

Patterns Of Genetic Differentiation Between Human Populations In Selected And Disease Associated Loci

MARTINEZ URKO(1); Lao, Oscar(3); Casals, Ferran(1); Calafell, Francesc(1); Bosch, Elena(1); Morcillo, Carlos(1); Navarro, Arcadi(1,2)
(1) Unitat de Biologia Evolutiva. Departament de Ciències Experimentals i de la Salut. Universitat Pompeu Fabra. Barcelona, Catalonia, Spain; (2) Institució Catalana de Recerca i Estudis Avançats (ICREA), Catalonia, Spain; (3) Department of Forensic Molecular Biology, Erasmus University Medical Centre Rotterdam, The Netherlands.

Single Nucleotide Polymorphisms (SNPs) are choice markers for the study of human genome variability patterns. They are key in many aspects of the study of function in our genomes, from the analysis of the genetic structure of complex disease to the study of human evolution. In particular, many SNPs have been associated to complex diseases or have been used to infer selective processes in certain populations. It has been hypothesized that some of the effects of natural selection upon disease/selection associated SNPs might represent adaptation to local conditions or local susceptibility to certain diseases and, thus, may imply population-specific SNP variability patterns. To study this possibility, we used HapMap Project data and compared SNPs belonging to putatively selected and/or disease-associated loci to SNPs in the rest of the genome.

SNP frequency patterns differed for different classes of loci (disease, positively selected, highly conserved). For example, SNPs genes putatively under positive selection presented larger differences between populations than the genome-wide average. We will discuss these and other results showing that genetic differences in human populations vary depending on the evolutionary history of each loci, and that population-specific adaptation has helped configuring the extant patterns of human genomic variability. Also, our results show that genetic differences between populations may be a key point when trying to replicate associations between human polymorphisms and complex diseases in different populations.

Maslen, Cheryl

Does Copy Number Variation Contribute To The Cause Of Congenital Heart Defects?

MASLEN, CHERYL; Babcock, Darcie; Hickey, Raymond; Reshey, Benjamin
Oregon Health & Science University, Portland Oregon, USA

Congenital heart defects (CHD) are the most common form of birth defect, occurring in nearly 1:100 live births. There is clustering in families, a high concordance in monozygotic twins, and frequent occurrence in cytogenetic abnormalities, indicating a strong genetic influence. However, the majority of CHDs occur as sporadic traits of unknown etiology. One of the most severe forms of CHD is atrioventricular septal defect (AVSD). Families with appreciable inheritance of AVSD are exceedingly rare, virtually preventing gene discovery by linkage analysis. Breakpoint analysis in some cytogenetic disorders that manifest AVSD have implicated various regions of the genome, although little progress has been made in associating individual gene defects with this malformation. One exception came from analysis of the critical region identified by breakpoint analysis in patients with 3p- syndrome. This resulted in the identification of the CRELD1 gene as a candidate for non-syndromic AVSD (Rupp et al, 2002). Subsequent studies demonstrated that mutations in CRELD1 are associated with AVSD, but incomplete penetrance indicates that additional risk factors are required to produce this heart defect (Robinson et al, 2003; Maslen et al, 2006). Another AVSD locus has been mapped to chromosome 1p in a rare family with autosomal dominant AVSD, suggesting additional genetic heterogeneity, but the gene has not been identified. Individuals with Down syndrome (trisomy 21) are at a 2000-fold increased risk for AVSD compared to the euploid population, implicating the additional copy of chromosome 21 as a major susceptibility factor. However, no single gene or group of genes on chromosome 21 has been shown to be the contributing factor for the increased susceptibility for AVSD either in Down syndrome, or in individuals with sporadically occurring isolated AVSD in the euploid population. The high incidence of AVSD in the context of trisomy 21 suggests that copy number variation (CNV) may play a role in causing AVSD. To determine if CNV contributes to the pathogenesis of AVSD in the euploid population, we will compare the genomes of individuals with sporadically occurring AVSD with those of their parents and controls, using comparative genome hybridization. Apparent *de novo* CNV events in affected individuals that do not occur in a control population will be further investigated to confirm spontaneous copy number changes associated with AVSD.

Rupp, P.A., Fouad, G.T., Egelston, C.A., Reifsteck, C.A., Olson, S.B., Knosp, W.M., Glanville, R.W., Thornburg, K.L., Robinson, S.W., Maslen, C.L. (2002) Identification, genomic organization and mRNA expression of CRELD1, the founding member of a unique family of matricellular proteins. Gene 293:47-57.
Robinson, S.W., Morris, C.D., Goldmuntz, E., Reller, M.D., Jones, M.A., Steiner, R.D, Maslen, C.L. (2003) Missense mutations in CRELD1 are associated with cardiac atrioventricular septal defects. Am. J. Hum. Genet. 72: 1047-1052.
Maslen, C.L., Babcock, D., Robinson, S.W., Bean L.J.H., Willour, V., Sherman, S. (2006) CRELD1 Mutations Contribute to the Occurrence of Cardiac Atrioventricular Septal Defects in Down Syndrome. Am. J. Med. Genet. 140: 2501-2505.

McCaughan, Frank

Exploration Of The Dynamics Of Genomic Evolution Of Premalignant Bronchial Epithelial Lesions

FM MCCAUGHAN (1,3,4), AT Bankier (1), BA Konfortov (1), CA Read (3), PJ George (3), TH Rabbitts (2), PH Dear (1), PH Rabbitts (2)
(1) MRC Laboratory of Molecular Biology, Hills Road, Cambridge CB2 2QH, United Kingdom; (UK); (2) Leeds Institute of Molecular Medicine, Beckett Street, Leeds LS9 7TF; (3) Centre for Respiratory Research, University College London, WC1E 6JF, UK; (4) MRC supported Clinical Research Training Fellow.

Epithelial cancers are thought to develop in a multistage process from normal epithelium through increasingly abnormal histological stages until lesions become fully invasive. There is a drive to explore the genetic and genomic aberrations occuring early in this multistage process. The rationale is that if the early events driving the dysplastic progression can be defined, they may represent ideal molecular signals to use as biomarkers or potential targets for therapeutic intervention.

To date opportunities to study genomic changes in preinvasive human lesions have been limited because biopsies tend to be very small, and processed by fixation in formalin. In addition the biopsies inevitably comprise heterogeneous cell populations so that epithelial cells may represent only a fraction of the whole biopsy. DNA retrieved from these biopsies is therefore degraded and often in picogramme quantities. This makes it particularly difficult to gain a reliable signal from whole genome array CGH-based techniques.

We have addressed these problems by applying a single molecule counting approach called Molecular Copy-number Counting to assess regional variation in copy number, concentrating primarily on chromosome 3. The comparison of early and late preinvasive lesions has provided valuable and novel insights into the dynamics of chromosome 3 genomic instability in preinvasive lesions. These results will be presented along with a perspective on how to translate this type of work into the development of a clinically useful molecular biomarker.

Mercader, Josep-Maria

Combined Family Based And Case-Control Association Study Of Neurotrophin Signalling Genes In Four Eating Disorders European Populations

JOSEP MARIA MERCADER(1), Ester Saus(1), Mònica Gratacòs(1,2), Rafael de Cid(2), Anna Carreras(2), Anna Puig(2), Juan R. Gonzàlez(2), Mònica Bayés(2), Fernando Fernández-Aranda(3), Elena Cellini(4), Benedetta Nacmias(4), Johannes Hebebrand(5), Anke Hinneyh(5) Claudette Boni(6), Philip Gorwood(6) and Xavier Estivill(1,2,7)
(1) Center for Genomic Regulation, (2) National Genotyping Center (CeGen) , and CIBERESP, (3) Psychiatric Service, Ciutat Sanitaria Bellvitge, L Hospitalet; (4) Department of Neurology and Psychiatric Sciences, University of Florence, Florence; (5) Rheinische Kliniken, Department of Child and Adolescent Psychiatry, University of Duisburg-Essen, Essen; (6) Hospital Louis Mourier, Adult Psychiatric Department, Paris; and (7) Pompeu Fabra University, Barcelona

Both animal models and association studies propose BDNF and NTRK2, as a key regulator of eating behaviour. To shed light on the potential involvement of other neurotrophins as susceptibility genes for eating disorders (ED), we performed a family based and population based association study for several candidate genes: Nerve Growth Factor (NGF), Brain-Derived Neurotrophic Factor (BDNF), Neurotrophic Tyrosine Kinase, Receptor, type 1 (NTRK1), Neurotrophic Growth Factor Receptor (NGFR), Neurotrophin 5 (NT4/5), Tyrosine Kinase, Receptor, type 2 (NTRK2), Neurotrophin 3 (NTF3), Neurotrophic Tyrosine Kinase, Receptor, type 3 (NTRK3), Ciliary Neurotrophic Factor (CNTF) and Ciliary Neurotrophic Factor Receptor (CNTFR). The clinical samples include 420 trios, 408 index cases and 385 controls from four European countries: Spain, Italy, France and Germany. Tag SNPs, selected from the CEU HapMap project dataset, were genotyped using SNPlex technology. We used FBAT software for the family Based Association Studies and the SNPassoc software to analyze the effect of SNPs on minimum body mass index and to perform the case-control studies. When taking ED as a whole group, 14 nominal associated SNPs were found, in NGF, NTF3, NTRK3, P75, CNTFR and NTRK2. Interestingly, there were 6 nominal associated SNPs in NTK3, one of which was significantly associated after Bonferroni Correction (p = 9.6 E-5). These results suggest the involvement of other neurotrophin genes apart from BDNF and NTRK2 in the susceptibility to eating disorders.

Supported by the Spanish Ministry of Education and Sicence, Instituto de Salud Carlos III, Danone Institute and Generalitat de Catalunya.

Milà, Montserrat

X-Chromosome Tiling Path Array Detection Of Copy Number Variants In Patients With Chromosome X-Linked Mental Retardation

I Madrigal(1,5), L Rodríguez-Revenga(1,5), L Armengol2, E González(3), LA Pérez-Jurado(4,5), X Estivill(2), and M MILÀ(1,5)
(1) Biochemistry and Molecular Genetics Department, Hospital Clínic and IDIBAPS (Institut d'Investigacions Biomèdiques August Pi i Sunyer), Barcelona
(2) Genes and Disease Program. Center for Genomic Regulation (CRG-UPF), Barcelona.
(3) Microarray Laboratory, Bioinformatics and Genomics Program, (CRG-UPF),Barcelona
(4) Genetics Unit, Universitat Pompeu Fabra, Program in Molecular Medicine and Genetics, Hospital Vall d'Hebron, Barcelona, Spain.
(5) Centre for Biomedical Research on Rare Diseases (CIBERER), ISCIII, Barcelona, Spain

Aproximately 5-10% of cases of mental retardation in males are due to copy number variations (CNV) on the X chromosome. Novel technologies, such as array comparative genomic hybridization (aCGH), may help to uncover cryptic rearrangements in X-linked mental retardation (XLMR) patients. We have constructed an X-chromosome tiling path array using bacterial artificial chromosomes (BACs) and validated it using samples with cytogenetically defined copy number changes. We have studied 54 patients with idiopathic mental retardation and 20 controls subjects. Known genomic aberrations were reliably detected on the array and eight novel submicroscopic imbalances, likely causative for the mental retardation (MR) phenotype, were detected. Putatively pathogenic rearrangements included three deletions and five duplications (ranging between 82 kb to one Mb), all but two affecting genes previously known to be responsible for XLMR. Additionally, we describe different CNV regions with significant different frequencies in XLMR and control subjects (44% vs. 20%). This tiling path array of the human X chromosome has proven successful for the detection and characterization of known rearrangements and novel CNVs in XLMR patients.

Monos, Dimitri

Long-Range Computational Haplotyping Of The Human Major Histocompatibility Complex Reveals Insufficiency Of Current Computational Methods For Accurate Haplotype Reconstruction

Kanterakis, Stathis(1); Roeder, Kathryn(2); Shen, Kui(3), Baldassano, Robert(1), Grant, Struan(4); Bradfield, Jonathan(4); Hakonarson, Hakon(4); Polychronakos, Constantin(5); MONOS, DIMITRI(1)
(1) The Children s Hospital of Philadelphia and Department of Pediatrics, University of Pennsylvania, School of Medicine; (2) Carnegie Mellon University, Department of Statistics; (3) Department of Computational Biology, University of Pittsburgh; (4) The Children s Hospital of Philadelphia, Center for Applied Genomics; (5) Departments of Pediatrics and Human Genetics, McGill University, Montreal, Québec, Canada.

Knowledge of haplotype phase can potentially provide valuable information on determining key haplotypic regions, as well as linkage disequilibrium in disease association studies. Numerous attempts have been made to derive haplotype phase computationally, most of which are highly successful for short and relatively sparse genomic sequences (up to 200 SNPs per 1Mb)[6]. In the present study, we attempted to use current software to computationally derive contiguous, long-range haplotypes of the Human Major Histocompatibility Complex (MHC), a genetically dense and highly polymorphic region of chromosome 6, including a large number of genes associated with the immune response. For this, 578 Caucasoid trios were SNP genotyped using the Illumina InfinumTM HumanHap500 BeadChip. True haplotypes were derived from the trio genotype information. fastPHASE[7] was subsequently used to infer haplotype phase from unphased genotypes in our population, for the MHC region (1800 SNPs). Inferred haplotypes were then compared to true haplotypes to assess the performance of haplotype inference of the MHC. We discovered that error rates were worse than reported in similar whole-genome phasing studies[6] and that phasing regions of more than 200 SNPs will not produce confident contiguous haplotypes (Incorrect Genotype Percentage (IGP) error > 15%). When reconstructing phase from unrelated individuals a crucial type of mistake is the switch error, the computational equivalent of a recombination. We tried to pinpoint where switch errors were occurring with greater frequency. We reveal a striking association between switch error positions and previously reported hotspots of recombination, within the MHC[8]. We conclude that inferring true, long-range, contiguous haplotypes of the MHC (1800 SNPs in 4Mb in our case) cannot be accurately done. However, recombination rate information from publicly available sources (such as the HapMap project) can be a useful tool in determining the likelihood that a given inferred haplotype region is accurately predicted. Molecular methods can then be used to supplement computational derivation in regions of high recombination.

6. Jonathan Marchini, David Cutler, Nick Patterson, Matthew Stephens, Eleazar Eskin, Eran Halperin, Shin Lin, Zhaohui S. Qin, Heather M. Munro, Gonc¸alo R. Abecasis, and Peter Donnelly, for the International HapMap Consortium. A Comparison of Phasing Algorithms for Trios and Unrelated Individuals, Am. J. Hum. Genet. 78:437-450, 2006.

7. Scheet, P. and M. Stephens. A fast and flexible statistical model for large-scale population genotype data: Applications to inferring missing genotypes and haplotypic phase. American Journal of Human Genetics 78:629-644, 2006.

8. Michael Cullen, Stephen P. Perfetto, William Klitz, George Nelson, and Mary Carrington. High-Resolution Patterns of Meiotic Recombination across the Human Major Histocompatibility Complex, Am. J. Hum. Genet. 71:759-776, 2002.

Morán Moguel, María-Cristina

Insertion/Deletion (I/D) Polymorphism In Angiotensin Converting Enzyme (ACE) In Mexican Women With Breast Cancer

MORÁN MOGUEL, MARÍA CRISTINA; Castro, Xochitl; Gutierrez, Susan; Mendizábal, Adriana; Vásquez, José G; Sánchez Corona, José.
División de Medicina Molecular. Centro de Investigación Biomédica de Occidente. Unidad de Anatomía Patológica. Hospital de Especialidades. Centro Médico Nacional de Occidente. Instituto Mexicano del Seguro Social. Guadalajara, Jalisco. México.

Introduction: The renin angiotensin system plays an important role in homeostasis. The conventional actions of its main effector angiotensin II are in the control of the arterial pressure, through two receptors AT1R and AT2R. In other nonclassic routes angiotensin II interacts through its receptors with diverse molecules in other complex processes like inflammation, apoptosis, angiogenesis, migration and cellular proliferation implied in cancer development. Lately, angiotensin II, has been attributed with angiogenic, mitogenic and growth factor actions in breast tissue, human cell lines and animal models of breast cancer [1]. Since 1998, several epidemiological studies have examined cancer risk in users of ACE inhibitors. Lever et al. observed lower breast cancer incidence in female users of ACE inhibitors relative to nonusers with comparable cardiovascular conditions [2]. The use of ACE inhibitors and the ACE I/D polymorphism may be linked to breast cancer. The polymorphism in intron 16 of ACE gene located in 17q23, is a 287 base pair insertion or deletion. The polymorphism accounts for the variability of ACE plasma concentrations [3].

Objective: The aim of the present study was to investigate the frequencie and evaluate the relationship of I/D polymorphism with breast cancer risk in Mexican postmenopausal women.

Methods: Ninety four paraffin-embedded tissue blocks were recovered from the pathology archive of Centro Médico Nacional de Occidente, Instituto Mexicano del Seguro Social. Blocks initially suspected of harboring breast cancer tissue were classified into two groups after histopathological assessment (cases and reference groups). We obtained 186 blood samples from general population to establish polymorphic frequencies and Hardy Weinberg equilibrium. The ACE I/D polymorphism was genotyped by PCR with specific primers [3].

Results: The Mexican population is in Hardy-Weinberg equilibrium with respect this polymorphism. The genotype frequencies in cases were II: 0.12, ID: 0.09 and DD: 0.78 and in controls were II: 0.03 ID: 0.1 and DD: 0.87. The DD carriers showed a significantly increased risk of developing breast cancer when compared with the I/D and I/I carriers (Odds ratio, 3.360; 95% CI, 1.437-7.859; p= 0.00353).

Conclusion: Our results suggest that the ACE I/D polymorphism plays an important role in breast cancer and benign breast disease risk in Mexican women. As the angiogenesis actions are triggered by angiotensina II, the possibility that in addition to ACE, the polymorphisms of other genes of Renin Angiotensin System are implied in cancer exist. Further study on these genotypes and other polymorphisms in RAS genes is required to analyze their association with breast cancer risk.

References.

1. Tahmasebi M, Barker S, Puddefoot JR, Vinson GP. Localisation of renin-angiotensin system (RAS) components in breast. Br J Cancer. 2006 Jul 3;95(1):67-74. Epub 2006 Jun 6.
2. Lever A. F., Hole D. J., Gillis C. R., McCallum I. R., McInnes G. T., MacKinnon P. L., Meredith P. A., Murray L. S., Reid J. L. and Robertson J. W. Do inhibitors of angiotensin-I-converting enzyme protect against risk of cancer? Lancet, 352:179-184,1998.
3. Rigat B., Hubert C., Alhenc-Gelas F., Cambien F., Corvol P. and Soubrier, F. An insertion/deletion polymorphism in the angiotensin I-converting enzyme gene accounting for half the variance of serum enzyme levels. J. Clin. Investig., 86: 1343-1346, 1990.

Mudge, Jonathan

The Importance Of Manual Annotation In Characterising SNPs And CNV In The Human Genome: Lessons Learned From The Vega Project

MUDGE, JONATHAN; Gibson, Richard; Gilbert, James; Wilming, Laurens; Harrow, Jennifer; Hubbard, Tim
Wellcome Trust Sanger Institute, Hinxton, Cambridge, UK

As the human genome project progresses from the initial sequencing phase into the identification of human variation, annotation projects must adapt to address incoming single nucleotide polymorphism (SNP) and copy number variation (CNV) data. The manual genome annotation provided by the HAVANA group has a number of advantages over automated projects such as ENSEMBL, all of which ultimately relate to the increase in accuracy afforded by human judgement. This judgement is of paramount importance in the comparative annotation of DNA from different humans, as illustrated by our ongoing work on human MHC haplotypes. Whilst SNPs within a new MHC haplotype are readily identified by alignment against the reference genome, the consequences of these substitutions frequently require in depth consideration. For example, we have classified loci containing changes within splicing signals, predicted to hinder efficient transcription. We also observe SNPs which affect protein structures beyond basic amino acid substitutions: both genes with missing or premature STOP codons and genes with variant START codons. In such cases our knowledge of processes such as nonsense-mediated decay and translational initiation, alongside consideration of protein domain structures and publications specific to the locus, allow us to judge the likelihood that an unorthodox protein is (a) translated and (b) functional.

Similarly, accuracy is essential in annotating the CNV common in MHC haplotypes. Here, we believe lessons can be learned from our parallel annotation on the mouse genome. Unlike humans, mice can be inbred towards a fully homozygous state, thus genome sequencing complications arising from heterozygosity within highly unstable regions such as MHC are largely avoided. We have recently sequenced the highly dynamic Major Urinary Protein (MUP) cluster from two divergent strains of laboratory mice, with each region believed to provide a distinct 'snap-shot' of the SNPs and CNV which exists in wild populations in a manner analogous to a pair of haplotypes. It is clear that MUP loci like MHC loci - are subjected to both copy gain and loss via the process of birth-and-death evolution. Again, it is clear that algorithms frequently fail to predict the functional consequences of nucleotide differences between duplicant loci. In particular, manual annotation is typically required to differentiate between predictions of pseudogenisation and subfunctionalisation / adaptation. Finally, our experiences with CNV annotation demonstrates the need for a coherent system of nomenclature for gene loci in order to avoid confusion when comparing orthologs between species or strains and paralogs between haplotypes.

The HAVANA annotation of the MHC haplotype regions and MUP clusters are available for comparison within the Vega web browser <www.vega.ac.uk>, specifically designed to display manually annotated genome sequence. Our detailed predictions regarding the functionality of loci, with a particular emphasis on splice variants, are reflected in our sophisticated gene classification system, providing users with a significant increase in information content over other genome browsers.

Muiños-Gimeno, Margarita

Patterns Of Variation And Linkage Disequilibrium In miRNA Genomic Regions: Implication For Association Studies

Y. Espinosa-Parrilla(1), M. MUIÑOS-GIMENO(1), M. Montfort(1), M. Bayés(1), X. Estivill(1,2)
(1) Genes and Disease Program, CeGen and CIBERESP, Center for Genomic Regulation (CRG-UPF), Barcelona; and (2) Pompeu Fabra University, Barcelona, Catalonia, Spain

MicroRNAs (miRNAs) have a crucial role as posttranscriptional regulators of genes, being involved in the regulation of the expression of at least a third of mammalian genes. Polymorphisms and mutations involving miRNAs sequences or their regulatory machinery are likely to represent an important source of variation in quantitative traits and contribute to the genetic susceptibility to many complex disorders. Association studies using single nucleotide polymorphisms (SNPs) in genomic regions containing miRNAs might help to evaluate functional miRNA allele variants with respect to disease. We constructed a panel of SNPs covering miRNA regions and studied their pattern of variation in the population. We first analyzed the organization of the whole collection of miRNAs at the genomic level and have defined 164 different regions spanning 2 Mb of genomic DNA and containing 326 known human miRNAs (MiRBase release 7.1), including the precursor sequence as well as at least 5 kb upstream and downstream of the miRNA. Forty-nine clusters containing 192 miRNAs were defined at a given inter-miRNA distance of 2 kb with two large clusters in chromosome 14 and 19, containing 23 and 44 miRNAs, respectively. Considering the SNP coverage of HapMap data (Rel 19/phase II), the SNP density of miRNA regions was considerably lower than in the average of the genome, with only 8 SNPs in miRNA precursor sequences (0.3 SNPs/kb). In order to achieve an optimal selection of informative SNPs in these regions, we combined tagSNP and random SNP selection methods to design a panel of 768 SNPs. Only 18 out of the 768 SNPs were located in premiRNAs sequences (8 SNPs from HapMap and 10 non typed SNPs from dbSNP). Genotypes for those SNPs were generated in 340 Spanish unrelated individuals using a custom Golden Gate assay from Illumina. The analysis of allele frequencies of SNPs located in the premiRNA showed that half of them are monomorphic in the studied population, which is suggestive of strong selective constraint on human miRNA sequences. Furthermore, we analyzed allele frequencies of all 768 SNPs and linkage disequilibrium blocks of the miRNA clusters in the Spanish control population and compared the data with the CEU population of HapMap. The analysis indicated similar allele frequencies and linkage disequilibrium patterns in the genomic regions of miRNAs in the Spanish population and the HapMap CEU sample confirming the applicability of our SNP panel to the study the association of miRNAs in complex disorders and common traits.

Supported by the Spanish Government (FI05/00061 and R&C program).

Munro, Robin

A Workflow Based Approach To Genome Wide Association Studies, From Large Data Set Quality Control, Through Analysis To Interpretation

MUNRO, ROBIN; Kalaitzopoulos, Dimitris; Zhang, Chi; Clark, Jim; Newell, William
InforSense Ltd, London, UK

We describe features of a newly developed software system for comprehensive analysis and interpretation of Genome-Wide Association (GWA) studies. InforSense GenSense provides methods for transforming raw data from high throughput genotyping platforms to a fully annotated list of genes associated with a measured phenotype. These include reading of large data sets (104 samples x 106 variables) from all major platforms. Using an internal data representation by removing sample name and locus name redundancies and a binary representation of genotype data allows fast in memory statistical calculations on complete GWA data sets. This optimized representation of genotype data can then be assessed for quality control; statistical analysis to identify significantly associated genetic markers and derive haplotypes; full annotation and visualisation of these markers using public domain genome databases. In addition, the system integrates access to third-party tools, such as HaploView and the UCSC Genome Browser. Due to the underlying workflow based approach to the analysis the system is highly configurable and extensible; allowing users to define their own customised analyses. Examples of this analytical workflow based approach are described here for data quality control, case control statistics and ways for further annotating SNP s in the context of biological systems.

Muramatsu, Masaaki

Nicotinamide N-Methyltransferase Gene Polymorphism Is A Novel Modifier Of Plasma Homocysteine Concentration In Healthy Japanese Men

MASAAKI MURAMATSU, Ling Zhang, Jungo Araki, Koichi Miyaki
Dept. Molecular Epidemiology, Medical Research Institute, Tokyo Medical and Dental University, Tokyo, Japan

Elevation of plasma total homocysteine concentration (tHcy) is a common and confirmed risk factor for atherosclerotic arterial diseases and venous thrombosis. Plasma tHcy concentration is determined by genetic and nutritional factors. Elevation of tHcy concentration is influenced by factors such as sex, age, renal function, vitamin intake and inheritance. Several genetic polymorphisms are reported to associate with tHcy concentration. MTHFR C677T polymorphism is the most well studied and has recurrently been shown to associate with hyperhomocystenemia in different populations. Others gene polymorphisms such as MTHFR A1298C, methionine synthase (MS) D919G, and methionine synthase reductase (MTRR) 66A/G are also reported to associate with plasma tHcy level. All of these genes encode enzymes involved in the homocysteine metabolism. Recently, the nicotinamide N-methyltransferase (NNMT) gene was identified as a novel genetic determinant for tHcy concentration through a family study of venous thrombosis in Spain. The objective of the current study is to determine whether the reported A/G polymorphism of NNMT gene (dbSNP: rs694539) associates with plasma tHcy concentration in healthy Japanese men.

A cross-sectional study was conducted among 313 unrelated Japanese male workers (age 45.8 ± 11.7; mean ± SD). The NNMT A/G and MTHFR C677T polymorphisms were determined by melting curve analysis. Clinical parameters including plasma tHcy, folate, and vitamin B12 concentrations were determined for each individual.
Overall, there was no association between the NNMT genotype and tHcy concentration (AA+AG vs. GG; 9.5 umol/L vs. 9.9 umol/L, p=0.254). When confounding factors were taken into account, the GG genotype associated with hyperhomocystenemia in subjects with high plasma tHcy, or those with low plasma folate, or in the elder (e40 y) group. An interaction with the MTHFR C677T genotype was also detected in low plasma folate group.

We conclude that the NNMT A/G polymorphism is not a strong determinant factor as the C677T polymorphism, but may have a role in modifying the tHcy concentration, especially in the at-risk groups in healthy Japanese men.

Mägi, Reedik

Revised Human Genome Sequence

REEDIK MÄGI, Reidar Andreson, Tarmo Puurand and Maido Remm
Department of Bioinformatics, Institute of Molecular and Cell Biology, University of Tartu, ESTONIA.

Comparing the human genome sequence with the HapMap data we observed that approximately 1,250,000 SNP positions in the current human genome sequence (NCBI Build 36) represent the minor allele in at least one HapMap panel (aka HapMap population). Approximately 42% (ca 521 000) of those minor allele positions are minor alleles in all three HapMap panels. The positions with minor alleles may influence comparative genomics studies, primer design or other studies involving genomic sequences. Therefore we created software for masking and/or replacing the SNP positions in genomic sequence (http://bioinfo.ut.ee/SNPmasker/). The software was used to generate a modified human genome sequence. In this modified genome sequence 521 000 SNP nucleotides that were represented by minor allele were replaced by major alleles. This sequence and HapMap genomic sequences fro three HapMap panels (CEU, CHB+JPT, YRI) are available for download from http://bioinfo.ut.ee/HMgenome/.

Naeem, Muhammad

A Mutation In The Hair Matrix And Cuticle Keratin KRTHB5 Gene Causes Ectodermal Dysplasia Of Hair And Nail Type

MUHAMMAD, NAEEM; Muhammad, Wajid; Suzanne, M, Leal; Wasim, Ahmad
Department of Biochemistry & Molecular Biology, Army Medical College, National University of Sciences & Technology (NUST), Rawalpindi, Pakistan

Ectodermal dysplasias are developmental disorders affecting tissues of ectodermal origin including hair, nails, teeth and sweat glands. To date, four different types of ectodermal dysplasia involving only hair and nails have been described, the molecular bases of which are entirely unknown. In an effort to identify the gene underlying this form of ectodermal dysplasia, two Pakistani consanguineous families (A & B) with multiple affected individuals have been ascertained. Microsatellite markers were genotyped in candidate regions and two point and multipoint parametric linkage analysis was carried out. The disease locus was mapped to chromosome 12q13.13 (Zmax=8.2 for family A; Zmax=3.1 for family B), which harbour six type II hair keratin genes. DNA sequence analysis of the coding exons and splice sites of six hair keratin genes in family A revealed a homozygous missense mutation in the hair matrix and cuticle keratin KRTHB5, leading to histidine substitution of a conserved arginine residue (R78H) located in the head domain. In family B, no pathogenic sequence aberration was identified, which suggests that mutation in this family either lies in the regulatory region of the keratin genes or in another unknown gene, located in the linkage interval, with a possible role in the development of ectodermal morphogenesis. Furthermore, several new polymorphisms were revealed during sequencing of hair keratin genes. This report provides the first direct evidence relating to the molecular pathogenesis of pure hair nail ectodermal dysplasia.

Note: The work presented in this abstract has been published in Journal of Medical Genetics (family A) and accepted for publication in Clinical and Experimental Dermatology (family B).

Naito, Kimitoshi

DVAR (DNA Variation And Phenotype Data Model) Is The Standard Description Format For Interoperability On DNA Variation And Phenotype Data

KIMITOSHI NAITO(1), Yasumasa Shigemoto(2), Takeshi Tomiki(3), Keiichiro Takahashi(4), Takashige Oroguchi(1), Akihiko Konagaya(5), Toshio Kojima(5), Anthony J. Brookes(6), Heikki Lehväslaiho(7), Juha Muilu(8), Martin Senger(9), Albert V. Smith(10), David Fredman(11), Debasis Dash(12), Haseena Rajeevan(13), Mathew Darlison(14), Mark Woon(15), Peter Rice(9), Ituro Inoue(16), Katsushi Tokunaga(17), Masako Kuroda(18), Hiroshi Mizushima(19), Hideaki Sugawara(20)
(1) Japan Biological Informatics Consortium, (2) Fujitsu Limited, (3) NEC Soft, Ltd, (4) Novus Gene Inc, (5) RIKEN GSC, (6) University of Leicester, (7) South African National Bioinformatics Institute, (8) Institute for Molecular Medicine Finland, University of Helsinki, (9) European Bioinformatics Institute, (10) Cold Spring Harbor Laboratory, (11) Bergen Center for Computational Science, (12) Institute of Genomic & Integrative Biology, (13) Yale University, (14) University College London, (15) Stanford University, (16) School of Medicine, Tokai University, (17) Department of Human Genetics, Graduate School of Medicine, University of Tokyo, (18) Japan Science and Technology Agency, Bioinformatics Division, (19) Information Center for Medical Sciences, Tokyo Medical and Dental University, (20) National Institute of Genetics

Genome sequence variation such as SNPs (Single Nucleotide Polymorphism) is deeply related to the phenotypic diversity. It can change individual responses to stimuli such as drugs and also can cause serious disease. Thus, many laboratories and consortiums all over the world have been carrying out a large-scale survey on correlation between genome sequence variation and phenotypic diversity.

In the meantime, databases are available through web such as JSNP (http://snp.ims.u-tokyo.ac.jp/), HGVbase (http://hgvbase.cgb.ki.se/), dbSNP (http://www.ncbi.nlm.nih.gov/SNP/) and TSC (http://snp.cshl.org/), and so forth. However, their data description formats are heterogeneous and it makes access to the database difficult. Therefore, we need the standard data description format.

Since 2003, we have developed standard description format of genome sequence variation data named PML (Polymorphism Markup Language) based on XML (Extensible Markup Language). In June 2005, PML was approved as international standard of genome sequence variation data description format by Life Science Research (LSR) of Object Management Group (OMG).

In September 2005, we held the 3rd International Bio-data Interoperability Conference (IBIC) in Tokyo, and discussed expansion of PML to phenotype data description named DVAR (DNA Variation and Phenotype Data Model). In October 2006, we held the 4th IBIC in Tokyo. It was an outcome of the conference that we proposed the initial submission of DVAR to LSR of OMG. DVAR was approved at OMG Technical Meeting in December 2006. In December 2007, we will execute the revised submission of DVAR to LSR of OMG.

DVAR is constituted of PML original classes and new classes for phenotype description. New classes are Phenotype class, Measurable _ feature class, Observable class, Observation class, Observation _ method class, Environment class, and Phenotype _ Category class. They are connected mutually and also they are connected to PML original classes.

Modeling of DVAR is composed of two phases same as PML; Platform Independent Model (PIM) and Platform Specific Model (PSM). PIM is a model independent of implement platform and is expressed in Unified Modeling Language (UML). We built best-suited PIM in consideration of the use case of exchanging genome sequence variation data. Based on the model described by UML, we developed PML which is a model depending on the platform of XML as PSM. The platform specific model for XML derived its architecture according to the XML schema specified by W3C documents: "XML Schema Part0: Primer (http://www.w3.org/tr/xmlschema-0/)," "XML Schema Part1: Structures (http://www.w3.org/tr/xmlschema-1/)," and "XML Schema Part2: Datatypes (http://www.w3.org/tr/xmlschema-2/)." Mapping from PIM to PSM is performed according to the framework designated by OMG standard "Model Driven Architecture (MDA)."

We have applied DVAR format to existing Phenotype and Genotype Database and proved that the existing databases can be expressed in the DVAR format. We are going to extend DVAR with a view to expand its application in healthcare and pharmaceutical industry.

Navarro, Arcadi

Pervasive Allele-Specific Gene Expression In The Human Genome

NAVARRO, ARCADI (1); Palacios, R. (2); Goñi, J. (2).; Piedrafita G. (2); Fernando, O. (3, 4); Gazave, E. (3), and Villoslada, P (2).
(1) ICREA and Unitat de Biologia Evolutiva. Universitat Pompeu Fabra, Barcelona, Spain; (2) Neuroimmunology Laboratory, Center for Applied Medical Research (CIMA), University of Navarra, Pamplona, Spain; (3) Unitat de Biologia Evolutiva. Universitat Pompeu Fabra, Barcelona, Spain; (4) Instituto de Tecnologia Química e Biológica (ITQB), Universidade Nova de Lisboa, Portugal.

Differential allelic gene expression appears to be an important factor in human phenotypic variability and, as a consequence, in the development of complex traits and diseases. Still, the magnitude, distribution and regulation of differential allelic gene expression remain largely unknown. In order to study allele-specific gene expression across the human genome, we have coupled genotyping with an analysis of allele-specific gene expression by screening 11,500 SNPs using the Mapping 10K Array to identify differential allelic expression. We found that 63% of the SNPs were subject to differential allelic expression, and were equally distributed along human chromosomes and biological processes. In addition, we have identified a regulatory effect of segmental duplications on allele specific gene expression of genes close by.

Nikopensius, Tiit

Comparison Of Allele And Haplotype Diversity Across 25 Genomic Regions In Three Eastern European Populations (Estonians And Russians)

NIKOPENSIUS, TIIT(1,3); Khrunin, Andrey(2); Mihailov, Evelin(1,3); Krjutakov, Kaarel(1,3); Limborska, Svetlana(2); Metspalu, Andres(1,3,4)
(1) Institute of Molecular and Cell Biology, University of Tartu, Tartu, Estonia; (2) Human Molecular Genetics Department, Institute of Molecular Genetics of Russian Academy of Sciences, Moscow, Russia; (3) Estonian Biocentre, Tartu, Estonia; (4) Estonian Genome Project, University of Tartu, Tartu, Estonia

The patterns of linkage disequlibrium (LD) and haplotype variation present crucial information for designing association mapping studies, searching common genetic variants contributing susceptibility to multifactorial diseases. Patterns of LD are also informative about population histories and human migrations. However, current studies have shown that patterns of LD may vary between populations and ethnic groups, even among populations of similar geographic origins (Sawyer et al., 2005). The HapMap study provides dense, genome-wide information on LD based on common markers in only a few populations. Although it has been demonstrated that HapMap data on CEPH samples are generally valid in other European populations (e.g. Montpetit et al., 2006), there are still population-based SNPs which are less frequent or are truly population-specific. We have carried out a genotyping study with 452 SNP markers from 25 genomic regions on different chromosomes in 140 Estonians and 207 Russian individuals from the Northern (Mezen) and Western (Andreapol) regions from the European part of Russia. Results from the present study have demonstrated highly consistent allele frequency distributions between three populations studied (R>0.91 for all pairwise comparisons). The overall frequency variation among populations was quite low (Fst=0.0054). The number of SNPs with high-range Fst values (0.02-0.09) was most prominent for MC5R gene region. Haplotype heterogeneity among populations was quite low (Fst values within 0.001-0.010, with exception for haploblocks in ADIPOR2 and MC5R regions). Finally, the interpopulation proximity was also evaluated through haplotype diversity. The higher concordance was observed between Estonians and Russians from Andreapol (North-West from Moscow, Tver oblast) (R=0.94; P<10-6) compared with other pairwise comparisons (R=0.83 for Andreapol vs Mezen and 0.85 for Estonians vs Mezen), that might be due to specific settlement events during ethnogenesis at these territories.

Nordgard, Silje

Deletion Of 16q In Breast Cancer Patients Is Associated With Survival And Predicted By Haplotypes On Chromosome 10

SILJE H. NORDGARD(1,2), Fredrik E. Johansen(1), Grethe I.G. Alnæs(1), Elmar Bucher(3), Bjørn Naume(4), Anne-Lise Børresen-Dale(1,2) and Vessela N. Kristensen(1,2)
(1) Department of Genetics, Institute of Cancer Research, Rikshospitalet-Radiumhospitalet Medical Center, Oslo, Norway, (2) Medical Faculty, University of Oslo, Oslo, Norway, (3) Medical Biotechnology, VTT Technical Research Center of Finland, Turku, Finland, (4) The Cancer Clinic, Rikshospitalet-Radiumhospitalet Medical Center, Oslo, Norway

Introduction: Deletion of chr 16q is frequent event in Breast Cancer (BC), suggesting the existence of a tumor suppressor gene on 16q. Breast cancer patients can be divided into 5 distinct subtypes depending their expression pattern of 561 transcripts (1). These five subgroups, ErbB2+, Basal-like, Normal-like, Luminal A and Luminal B, are associated with different clinical outcomes (2).

Method: Blood and tumor DNA from 112 patients with early breast cancer were genotyped using the Human-1 109K SNPs array from Illumina. The tumor genome profiles of the log R ratios were visualized using CGH explorer (3), and DNA gains and losses were called using the Analysis of Copy Errors (ACE) with a False Discovery Rate (FDR) <0.0001 implemented in the tool. Deletion of 16q was scored as the presence of at least one out of the 1886 deleted probes on chromosome 16q. Whole genome mRNA expression was analyzed in breast tumors with 42K Stanford cDNA arrays (4). We used both SAM and WGA (5) for the association analysis performed.

Results: Deletion of 16q was significantly associated with better survival (p=.0007). Patients with no aberration calls on 16q or with amplification had worse prognosis (p=.037). The most significant association to survival was mapped to an area conserved in Caucasians, Yoruba, Japanese and Han Chinese residing on 16q22.2 upstream of the ATBF1 gene. Studying the distribution of patients with 16q deletion among the different BC subtypes revealed an overrepresentation in tumors from patients grouped to the Luminal A subtype (83%) compared to the Luminal B (64%), ErbB2+ (267%), Basal-like (67%) and Normal-like (54%), p=.001. SNPs in CHST3 and SPOCK2 on chromosome 10q were associated to the 16q deletion status (q-value= 7.35e-05). Phasing of the genotype data showed the existence of distinct Haplotypes both in CHST3 and SPOCK2 that were associated to the deletion status (p=5.0e10-4 and p=.0018, respectively). CHST3 and SPOCK2 are both extracellular matrix proteins.

Conclusion: There may be a selection in early tumor cells towards the deletion of the tumor suppressor gene ATBF1, and during this selection process, usually, most of the q arm of chromosome 16 is lost. This event results amongst others in deletion of the P-cadherin gene, CDH3. The CDH3 maps in close proximity to ATBF1. CDH3 is one of the 561 transcripts whose expression classifies the 5 BC subgroups (1). One may speculate that reduced expression of this gene inhibits the tumor cell from developing into more advanced stages of cancer. The association between Haplotypes in CHST3 and SPOCK2 on chr 10 and the presence of deletion of 16q may indicate some gene-gene interaction within the extracellular matrix gene family.

1. Perou et al. Nature 2001
2. Sørlie et al. PNAS 2001
3.Lingjaerde et al. 821-22
4. TS in press in Molecular Oncology
5. Gonzalez et al. Bioinformatics 2007

Nuytemans, Karen

Estimation Of The Frequency Of Patient-Related Copy Number Variations In Causal PD Genes In The Belgian Population

K. NUYTEMANS(1,2,5), N. Brouwers(1,2,5), P. Pals(1,3,5), S. Engelborghs(4,5,6), B. Pickut(6), V. Bogaerts(1,2,5), E. Corsmit(1,2,5), M. Van den Broeck(1,2,5), P. P. De Deyn(4,5,6), J. Theuns(1,2,5), C. Van Broeckhoven(1,2,5)
(1) Neurodegenerative Brain Diseases Group, Department of Molecular Genetics; (2) Laboratory of Neurogenetics, (3) Laboratory of Neurobiology and (4) Laboratory of Neurochemistry and Behaviour, Institute Born-Bunge; (5) University of Antwerp, (6) Department of Neurology, Middelheim General Hospital; Antwerp; Belgium.

Recent genetic studies have provided evidence that the spectrum of mutations causing complex neurodegenerative disorders is more complex than simple missense mutations. Particularly in Parkinson s disease (PD) the role of gene dosage became more evident. So far, four out of five of the genes in which missense mutations lead to familial PD have also been reported to have PD associated dosage mutations (SNCA, PARK2, DJ-1 and PINK1). It is conceivable that copy number variations (CNVs) in these genes are currently underestimated since most mutation analyses did not include an extensive search for CNVs due to technical difficulties of the screening techniques. Accurate frequency estimates, however, are essential to determine their contribution and role in PD etiology.

We determined the frequency of CNVs in the four reported dosage sensitive PD genes, SNCA, PARK2, DJ-1 and PINK1, in 250 Belgian PD patients. Using a combination of TaqMan-MGB and SybrGreen real-time PCR and multiplex amplicon quantification (MAQ) assays we screened SNCA and PINK1 for the presence of whole gene multiplications and PARK2 and DJ-1 for single exon or promoter deletions or duplications. We did not identify multiplications of SNCA in the Belgian PD patients, which is consistent with the low frequency of SNCA CNVs in other PD populations reported so far. On the other hand we did identify 11 Belgian patients with PARK2 exon deletions or duplications demonstrating that also in the Belgian population (13 patients with PARK2 base substitutions) approximately 50% of the parkin mutations are copy number variations. We did not observe any deletion or duplication in DJ-1 or PINK1 in the Belgian PD population. Currently we are assessing the frequency of CNVs in PD genes in healthy control individuals and comparing the frequency of patient specific CNVs with pathogenic missense mutations. We can conclude from this study that CNV analyses should be included in standard mutation screenings of known and novel PD genes. In particular PARK2 dosage studies should be included in further genetic testing of PD patients and control individuals worldwide. Moreover, further genetic and extensive functional analysis of these CNV s are mandatory to estimate their biological relevance in PD pathogenesis.

Orr-Urtreger, Avi

High-Throughput Detection Of Genetic Variations Associated With Parkinson's Disease

ORR-URTREGER, AVI, Bar-Shira, Anat, Kedmi, Merav, Shifrin, Chen, Gan-Or, Ziv, Rozovski, Uri, Rosner, Serena, Gurevich, Tania, Giladi, Nir.
Genetic Institute & Movement Disorders Unit, Parkinson Center, Tel-Aviv Sourasky Medical Center, Sackler Faculty of Medicine, Tel-Aviv University, Israel.

The unique structure and origin of the relatively homogeneous Ashkenazi population has been valuable for the study of carcinogenesis and genetic syndromes, and founder mutations detected in Jews were identified in genes associated with disease in the population-at-large. In recent years, our group has successfully implemented a wide variety of genetic tools to study the possible roles of candidate genes in prostate cancer in Ashkenazi Jews[1-4]. Additionally, we have demonstrated that a combined research approach including array-CGH, expression-microarrays and bioinformatic analyses successfully revealed genes associated with prostate cancer pathogenesis and metastasis[5]. Similarly, we believe that the study of the Ashkenazi Jewish population will be valuable for the elucidation of genetic factors underlying Parkinson's disease (PD). PD is the second most common neurodegenerative disorder affecting 1-4% of the worldwide population over 60 years of age. While environmental factors have traditionally been implicated in disease development, substantial evidence in the last decade suggests that the contribution of genetics is greater than previously appreciated. Linkage studies have identified 11 PD-linked chromosomal loci, and causal mutations have been identified in six genes (SNCA, parkin, UCHL1, DJ1, PINK1, and LRRK2). We have established an extensive cohort of Jewish Ashkenazi PD patients and controls as well as a database of medical and lifestyle information, environmental risk factors and family history, and a biobank consisting of DNA and RNA from peripheral blood leukocytes. Recently, we screened our cohort for mutations in the LRRK2 gene, the most common genetic determinant of PD identified to date, and for mutations in GBA, the gene underlying Gaucher disease. Among the Ashkenazi PD patients, we identified 14.8% LRRK2 G2019S6 and 19.3% GBA mutation carriers, compared to 2.4% and 6.3% carriage in controls, respectively, suggesting a significant role for these mutations in PD risk. The unusually high percentage of mutation carriers for a complex inherited disease such as PD in our homogeneous Ashkenazi population, prompted us to search for additional genetic variations that might be relevant to PD pathogenesis, and specifically, for disease development in LRRK2 and GBA mutation carriers. Since gene expression changes in peripheral blood leukocytes were shown to be relevant for neurological diseases including PD, we have initiated global gene expression analysis to determine PD transcriptional profiles and to detect abnormalities in gene regulation and splicing in 120 PD patients and controls, using the Affymetrix human exon array. Simultaneously, DNA samples from 450 PD patients and controls will be SNP genotyped using the Whole-genome 500K SNP Array. We expect that the combination of these two high-throughput genetic approaches followed by extensive bioinformatic analysis and validation of candidate genes, and the correlation with clinical variables in our well characterized homogeneous Ashkenazi patients population will delineate novel genes and markers associated with PD risk and disease phenotype. Finally, our goal is to identify a set of biomarkers for presymptomatic identification of high risk individuals, to allow early intervention and the development of new therapeutic approaches.

1. Am J Hum Genet 71:981-4, 2002
2. Cancer Epidemiol Biomarkers Prev 15:474-9, 2006
3. Prostate 66:1052-60, 2006
4. Prostate 67:8-13, 2007
5. Cancer Research 62:6803-7, 2002
6. Neurology in press, 2007

Pappaioannou, Dimitri

A Novel Approach To Whole Genome Amplification And Labeling Of DNA Samples For Copy Number Variation Detection On BAC Microarrays

D. PAPPAIOANNOU(1), G. Schock(2), S. Schoenmakers(1), D. Postma(1), EJ. Klok(1) and F. Opdam(1)
(1) KREATECH Biotechnology B.V., Amsterdam, The Netherlands.
(2) Qiagen GmbH, Hilden, Germany

Over the past decades comparative genomic hybridization has proved to be a powerful tool when it comes to analyzing DNA copy number changes. With the advent of BAC microarrays, an unprecedented high resolution for whole genome scanning within one single experiment has now become feasible. BAC array based CGH analysis has become 'the' tool of choice for detection of copy number changes in tumors and genetic disorders.

The Universal Linkage System (ULS™) is technology that allows direct (non-enzymatic) labeling of DNA with fluorescent dyes for Microarray analysis. For BAC arrayCGH applications the ULS system demonstrates one of its key advantages by the direct labeling of genomic DNA samples isolated from both fresh and archival samples. For instance, ULS technology makes it easier to obtain high-quality BAC arrayCGH data from formalin fixed paraffin embedded (FFPE) material.

In addition, many arrayCGH applications will require significant amounts of genomic DNA from each sample whereas the available amount of DNA can be limiting. For these samples whole-genome amplification (WGA) is a necessity prior to labeling and hybridization. The WGA amplification technology uses the REPLI-g ® technology from QIAGEN which gives higher yields and improved uniformity of amplified DNA in comparison with Random Prime methods using modified nucleotides. Subsequently, the ULS technology provides a flexible way to label the amplified DNA without a second enzymatic step. This results in a very robust amplification and labeling procedure with less introduction of bias compared to conventional technologies.

Here we will report data on the performance of the ULS technology in BAC arrayCGH experiments on genomic DNA, FFPE stored DNA and REPLI-g ® amplified DNA.

Paquin, Bruno

Genome-Wide Association Studies For Schizophrenia And Endometriosis Using Samples From The Quebec Founder Population

PAQUIN, BRUNO; Raelson, John; Van Eerdewegh, Paul; Paquin, Nouzha; Briand, Sandie; Croteau, Pascal; Lapalme, Micheline; Hooper, John; Belouchi, Abdelmajid; Keith, Tim
Genizon Biosciences, Ville-St-Laurent, QC, Canada

Genizon is involved in the discovery of disease susceptibility genes in more than 20 complex disease areas using samples from the Quebec founder population (QFP). To date, we have successfully completed ten genome-wide association studies (GWAS) for various complex diseases. Here, we present an overview of our recent scans for schizophrenia and endometriosis. Both scans were carried out with similar sample sizes, 516 cases and 516 controls for schizophrenia, and 511 cases and 511 controls for endometriosis. The marker map comprised 374,187 SNPs, consisting of the Illumina HAP300 chip (317,504 tag SNPs) supplemented with 56,683 SNPs tailored to the LD structure of the QFP (a combination of tag SNPs and LD spacing with higher density of SNPs in low-LD regions). We ran in parallel two case/control association analyses, one based on haploypes (window sizes of 1, 3, 5, 7 and 9 markers) and one based exclusively on single markers. Genome-wide significance of the obtained P values was assessed using a permutation-based approach, following the same methodology as for the original genome-wide scan. Signals with P values that met the criteria for genome-wide significance were observed from both the haplotype and single-marker analyses for schizophrenia but only from single-marker association tests for endometriosis. For schizophrenia, we observed 16 regions with haplotype P values < 10-6, including one with P values < 10-8, whereas single-marker association identified 6 regions with P values < 10-5 including 2 with p values < 10-6. In comparison, for endometriosis, we also observed 6 of regions with P values < 10-5, including 1 with P value < 10-6. Additional regions were identified from gender-specific (schizophrenia study only), sub-phenotype and conditional analyses. The latter allows detection of gene-gene interactions such as epistasis and genetic heterogeneity. The statistical significance of the signals observed in each additional analysis is also assessed from a permutation-based approach following a similar principle as for the full sample GWAS. The identified candidate regions from both studies are genetically well resolved with many of them containing only 1 or 2 genes. The identified genes from each study were used to build GeneMaps, networks of interacting disease susceptibility genes and their biological pathways. The GeneMaps provide a comprehensive understanding of the genetic etiology of the diseases and a powerful tool for the development of diagnostics and novel therapeutics. For the two studies, the GeneMaps include biologically relevant pathways as well as novel pathways not previously known to be associated with their respective diseases.

Parnell, Laurence

Nutrigenomics And Gene Expression. The Influence Of SNPs And The Response To Dietary Intake

LAURENCE D PARNELL, Jacqueline Lane, Jian Shen, Jose Carlos Rodriguez Rey*, Chao-Qiang Lai, Jose M Ordovas
Nutrition and Genomics Laboratory, Human Nutrition Research Center on Aging, Agriculture Research Service, US Dept of Agriculture, Tufts University, Boston, MA, USA; *Department of Molecular Biology, University of Cantabria, SPAIN

Metabolic syndrome can be defined as central adiposity coupled with some or all of the following: elevated triglycerides, reduced HDL-cholesterol (HDL-C), raised blood pressure and raised fasting plasma glucose. Persistence in this state leads to long-term health effects of obesity, diabetes, atherosclerosis, cardiovascular disease and stroke. Nutrition has a profound influence on the emergence of metabolic syndrome and its transition to more serious health conditions. Genome variation also influences an individual's health status. Thus, the fields of nutrigenomics and nutrigenetics seek to uncover the relationships between diet, health and the human genome. Examples of gene-diet interactions include the LIPC (hepatic lipase) -514T allele, which associates with greater HDL-C only when diet is <30% fat by energy in whites, and the variation at -75 of APOA1 (apolipoprotein A-I), where the A allele in women who have higher polyunsaturated fatty acids (PUFA) intake associates with elevated HDL-C and where G/G individuals showed lower HDL-C only when intake of PUFA was higher. Additionally, we have reported that the -1123C allele of APOA5 (apolipoprotein A-V), affecting a binding site for hepatocyte nuclear factor 4 (HNF-4), associated with increased triglycerides only when n-6 PUFA intake is elevated.

We believe that these and numerous other polymorphisms within gene expression control regions drive many phenotypic differences important to response to the environment, in this case response to diet. Therefore, in order to focus our genotyping to SNPs that are functional with respect to affecting transcription, we have identified putative transcription factor binding sites both upstream and within genes pertinent to metabolic syndrome. We then correlated those data with known and proprietary SNP data and have identified several polymorphisms altering putative transcription factor binding sites in genes such as APOA2, CRP, LPL, MTTP, PBEF1, PPARGC1A and others. Association studies with these SNPs in at least three large populations have revealed several interesting results. For example, we have extended the findings of variation at -265 of APOA2, demonstrating that the minor allele, with its concomitant loss of the predicted C/EBP binding site, associates with increased values of BMI and intakes of fat and total energy. A SNP within intron 1 of CRP (C-reactive protein), a marker of inflammation, has a minor T allele that abolishes a predicted binding site for GFI1 (growth factor independent 1, a zinc finger protein involved in immunosuppression) and associates with reduced levels of CRP in serum and elevated amounts of HDL-C. Gel-shift assays confirm the allele-specific nature of the functionality of many of these sites. In order to discover new genetic contributors to metabolic syndrome and associated phenotypes, we have utilized recent data uncovering the genetic variation in gene expression (Stranger et al., 2007 Science 315:848.), mining those data for both genes with a potential role in metabolic syndrome and for SNPs with allele-specific binding sites for transcription factors pertinent to the response to diet. This approach has revealed a number of new candidate metabolic syndrome alleles. By bringing together genome variation as it affects gene expression and phenotypic data relevant to metabolic syndrome, we are beginning to be able to draw inferences that explain an individual's response to diet and the resulting health effects.

Pawlak, Andrzej

The Combinations CC/LM And CT/LM Of PON1 -108 C>T; L55M SNPs Are More Prone To The Aneurysmal As Compared To The Occlusive Type Of The Arterial Diseases

Strauss Ewa(1), Waliszewski Krzysztof(2), Majewski Waclaw(2), Gluszek Jerzy(2), PAWLAK ANDRZEJ(1)
(1) Institute of Human Genetics, Polish Academy of Sciences, Poznan, Poland; (2) Medical University, Clinics of Vascular Surgery and Arterial Hypertension Poznan, Poland

Studied groups included 453 arterial disease patients and 202 persons without symptoms of vascular disease. The group of patients comprised 238 coronary artery disease (CAD), 109 aorto iliac occlusive disease (AOID) considered as the occlusive/arteriosclerotic type of arterial disease and 106 abdominal aorta aneurysms (AAA) cases.

Paraoxonase (homocysteine thiolactone hydrolase, Jakubowski: J Nutr 136: 1741S-1749S, 2006) was considered as an important and relatively specific factor in pathogenesis of arterial diseases. Therefore the genetic variants of the PON1 gene were assumed as a possible factors influencing the risk of the arterial diseases of the occlusive/arteriosclerotic vs aneurysmal types.

The PON1 -108 C>T; L55M SNPs were determined by PCR RLFP method. The frequency of PON1 -108 C>T; L55M CT/LM combination was 35,8% in the AAA patients group as compared to 20,6% in the matched control group (n=97) (different at p=0,02; OR=2,0 (0,9-4,5) referred to the homozygotes CC/AA). Significant differences were noted in frequency of CC/LM and CT/LM between the aneurysmal arterial diseases group (AAA patients) and the occlusive/arteriosclerotic group (combined AOID and CAD groups). The combinations of PON1 -108 C>T; L55M CC/LM and CT/LM conferred greater risk (OR) of AAA by 2,6 (p=0,003) and 1,6 (p=0,05) in relation to the risk of occlusive/arteriosclerotic arterial diseases. In the multivariate analysis the effect of PON1 genotype was independent of the other risk factors.

The analysis of the age dependency of the studied differences in PON1 marker genotype combinations shows the differences increasing in the older patients. The earlier observation that the frequency of PON1 -108T allele is lower in the older persons in the control group should be considered in interpretation of the age effects in the patients groups (Strauss, J Phys.Pharm. 2005, 56, suppl.2, 65-75).

Supported by grants from Ministry of Education PO5C 03828 and N402 081 31/2499 and from State Committee for Scientific Research no 3 P05A 121 24

Perez-Jurado, Luis

Copy Number Variation At The 7q11.23 Segmental Duplications Is A Susceptibility Factor For The Williams-Beuren Syndrome Deletion

Cuscó I(1) Corominas R(1,2) Bayés M(3) Flores R(1) Rivera N(1) Campuzano V(1) PÉREZ JURADO LA(1,4)

(1) Genetics Unit, Universitat Pompeu Fabra and CIBERER, Barcelona, Spain
(2) Research Group in Neurometabolic Disorders, Hospital Vall d'Hebron, Barcelona, Spain
(3) CEGEN, Centre for Genomic Regulation, Barcelona, Spain
(4) Program in Molecular Medicine and Genetics, Hospital Vall d'Hebron, Barcelona, Spain

Large copy number variants (CNVs) have been recently found as structural polymorphisms of the human genome of still unknown biological significance. CNVs are significantly enriched in regions with segmental duplications or low-copy repeats (LCRs). Williams-Beuren syndrome (WBS) is a neurodevelopmental disorder caused by a heterozygous deletion of contiguous genes at 7q11.23 mediated by non allelic-homologous recombination (NAHR) between large flanking LCRs and facilitated by a structural variant of the region, a ~2Mb paracentric inversion present in ~25% of WBS transmitting progenitors. We have found that 7 out of 160 (4.37%) WBS transmitting progenitors are carriers of a large deletion of LCRs. The prevalence of this deletion type CNV among control individuals and non-transmitting progenitors is much lower (1%, n=500), thus indicating that it is a predisposing factor for the WBS deletion (odds ratio 4.5 fold, P=0,013). LCR duplications were found in 2.5% of WBS transmitting progenitors but also in 1.4% of controls, what implies a non-statistically significant increase in WBS transmitting progenitors. We have characterized the organization and breakpoints of these CNVs, encompassing ~400 kb of genomic DNA and containing several pseudogenes but no functional genes. Additional structural variants of the region have also been defined, all generated by NAHR between different blocks of segmental duplications. Our data further illustrate the highly dynamic structure of regions rich in segmental duplications such as the WBS locus, and indicate that large CNVs can act as susceptibility alleles for disease-associated genomic rearrangements in the progeny.

Prathap Naidu, Boode

Implication On The Genetic Origins Of Maharashtra (India) Tribal Population: Inference From High-Resolution Mitochodrial DNA And Y Chromosome Studies

B. PRATHAP NAIDU(1), Kumarasamy Thangaraj(1), Gyaneshwer Chaubey(1,2), S. R. Walimbe(3), Shashank Upadhyay(1), Alla. G. Reddy(1) and Lalji Singh(1)
(1) Centre for Cellular and Molecular Biology, Hyderabad-500 007; (2) Estonian Biocentre, Riia, 23, Tartu- 51010, Estonia; (3) Deccan College Post-Graduate and Research Institute, Pune-411 006.

The Indian subcontinent is legendary for the cultural, linguistic and genetic diversity of its inhabitants. As many castes and tribes inhabit Indian subcontinent, it is tacit that association between these various populations may define the present genetic landscape of India. Taking this assumption, geographical and ethnic diversity into account and to answer the question of the origin of Indian maternal and paternal lineages further, we analyzed 14000bp of the mitochondrial DNA in 181 samples, three complete mtDNA sequences and 13 biallelic Y-chromosome SNPs and 17 STRs in two tribal groups from Maharashtra state (Central India) and compared the results with available data from the Indian sub continent. Our current study suggests, presence of twenty sub haplogroups of M including one previously uncharacterized haplogroup, which we named it as M48. Territories of Maharashtra state (Central India) played pivotal role in the expansion of haplogroup U7, as it is evident from significant frequencies of haplogroup U7 in the studied populations. High frequency (%) of haplogroup U in studied tribal populations using high-resolution studies of mtDNA strongly supports an extensive deep late Pleistocene genetic link between contemporary Europeans and Indians. The possibility for the North East origin of haplogroup M33 can now be ruled out, as it is observed in significant frequency in the studied tribal populations. Further more, qualitative and quantitative mtDNA studies are needed from Maharashtra state (Central India) to disturb the North East origin of haplogroup M33. On the other hand, Y chromosome studies strongly supports the Indian origin of R1a-M17, as it is apparent from the significant frequencies of this haplogroup in the studied populations and also from the chenchu tribe. In addition, the Y chromosome of lower caste population shows significant homology with the tribal population and hence supports the tribal origin of the lower caste. Finally, significant frequencies of H-M52 and F-M89 are observed both in the tribes and in the lower castes, hence both of them can now be considered as a tribal/lower caste marker rather than tribal marker alone.

Puurand, Tarmo

Finding Deletions And Inversions From The Human Genome

PUURAND, TARMO; Remm, Maido
University of Tartu, Tartu, Estonia

We have implemented algorithm for finding deletions and inversions from the entire human genome.

The algorithm is based on comparison of trace sequences and the finished genome sequence.

Key steps of the algorithm are the following. 1. Location of all available (currently 90 000 000) trace sequences is identified by BLAST searches against the human genome. 2. The traces that have discontinuous location (different parts of the trace show different locations in genome) are filtered out and analysed further. 3. Traces that show discontinuous locations on opposite strands of the genome are classified as inversions, traces that show discontinuous locations on same strand of the genome are classified as deletions.

We used a known inversion region from the chr17 (Stefansson et al., Nature Genetics, 2005) as a positive control to test the algorithm. Our algorithm identified the published inversion and several deletions at the same location.

We will present data about identified inversions from the entire genome and supporting evidence for inversions.

Raza, Tasleem

Mutation Of Exon 4 Region Of FVIII Gene In Indian Hemophiliacs

SYED TASLEEM RAZA, Nuzhat Husain, Farzana Mahdi
Genetics Unit, Post Graduate Department of Pathology King George.s Medical University, Lucknow, India & Era.s Lucknow Medical College and Hospital Lucknow, India

BACKGROUND: Hemophilia A is a X linked bleeding disorder caused by deficiency of factor FVIII. The disease affects one in 10,000 males world wide. Prevention of birth of affected fetus is the best current day option to decrease the disease burden. The exon 4 of the factor VIII gene is a frequently mutated site in cases with Hemophilia A and in a preliminary study of all 26 exons in 22 cases we have found that exon 4 mutation in 20/100 cases with mutation.

AIMS & OBJECTIVES: To analyze small mutational changes in Exon 4 region of FVIII gene in the Indian cases with Hemophilia A Indian population.

MATERIAL & METHOD: The study group included 100 cases with Hemophilia A. On the basis of the FVIII levels 54 of 100 cases were categorized as severe hemophilia (<1% FVIIIC), 42 cases had moderate severity of disease (1-5% FVIIIC) and 4 cases of mild disease (5-30% FVIIIC). DNA was extracted by using standard phenol chloroform method (Kunkel et al) PCR amplification of genomic DNA from patients was performed with thermostable DNA polymerase using primer sequences 5'-GCCGATCCAGAAAGGACAATTTTAT-3' and 5'-TTGGATCCTGCTTATTTCATCTCA-3'. Single Stranded Conformational Polymrphism was performed according to (Lin and Shen 1993) with modifications. 15µL of amplified sample heated with 94°C for 6 minute with an equal volume of loading buffer (0.055µL bromophenol blue and 955µL form amide), the PCR products were snap-cooled and immediately loaded on to the 8% polyacrylamide gel and run overnight at150V.DNA fragments showing altered position were eluted and sequenced form Lab India Instruments India Ltd. Automatic nucleotide sequencing was done to confirm the SSCP results. Nucleotide sequence were translated to amino acid using online tool available at http://www.justbio.com.The nucleotide sequences and translated amino acids were aligned with blast program and gene bank database to compare with corresponding sequences of Human FVIII gene sequence, accession number AE 52420 Amino acid sequence CAD97566 and nucleotide sequence AH002692 have been used for sequence alignment using BLAST and Bioedit programs.
.
RESULTS: This study reports the result of mutation analysis of exon 4 of FVIII gene in a consecutive series of 100 male hemophiliacs. A positive family history of disease was identified in 48/100 (48%). Twenty of the hundred cases had total 37 mutations in the exon 4. The types of mutations included 30 missense, and 7 deletions. Thirty six novel mutations have been observed .The sequence was submitted to gene bank with the accession numbers DQ910174, DQ9101745, DQ910176, DQ910177, EF064778, EF064779, EF064780, EF064781, EF064782, EF064783, and EF064784.

CONCLUSION: Mutation in exon 4 region appears to be common in Indian population. Since direct mutation detection involves individual/nested amplification of all 26 exonic regions. It is important to device an approach in different populations depending on the frequency of mutations at different exonic sites.

REFERENCES:
1. Kunkel LM, Smith KD, Boyer SH, et al. Analysis of human Y-chromosome-specific reiterated DNA in chromosome variants. Proc Natl Acad Sci USA 1977 Mar; 74(3): 1245-49
2. Lin,S.W., and ShenM.C. (1993).Genetic basis and carrier detection of Hemophilia B of Chinese origin.Thromb.haemostasis 69:247-252.

Ribasés-Haro, Marta

Association Of Childhood And Adulthood Attention-Deficit/Hyperactivity Disorder And Neurotrophic Factors: Contribution Of NTF3, CNTFR and NTRK2

MARTA RIBASÉS(1), Amaya Hervás(2), Josep Antoni Ramos-Quiroga(1,3), Rosa Bosch(1), Anna Bielsa(1), Xavier Gastaminza(1), Silvia Rodriguez-Ben(2), Mònica Gratacòs(4,5,6), Xavier Estivill(4,5,6), Miquel Casas(1,3), Bru Cormand(7,8,9) and Mònica Bayés(4,5,6)
(1) Department of Psychiatry, Hospital Universitari Vall d Hebron, Barcelona, Spain; (2) Child and Adolescent Mental Health Unit, Hospital Mútua de Terrassa, Barcelona, Spain; (3) Department of Psychiatry and Legal Medicine, Universitat Autónoma de Barcelona, Spain; (4) Genes and Disease Program, Center for Genomic Regulation (CRG), UPF, Barcelona, Spain; (5) Centro Nacional de Genotipado (CeGen), Barcelona, Spain; (6) CIBER Epidemiología y Salud Pública, Instituto de Salud Carlos III (CRG), Barcelona, Spain; (7) Departament de Genètica, Facultat de Biologia, Universitat de Barcelona, Spain; (8) CIBER Enfermedades Raras, Instituto de Salud Carlos III, Barcelona, Spain; (9) Institut de Biomedicina de la Universitat de Barcelona (IBUB), Catalonia, Spain

Introduction
Attention-deficit/hyperactivity disorder (ADHD) is a common psychiatric disorder that results from the interaction of different genetic and environmental susceptibility factors. Animal, genetic, and pharmacological studies support that neurotrophic factors and their receptors, which play an essential role in synaptic efficiency and neuronal survival, differentiation and plasticity, may participate in the etiology of ADHD.

Methodology
To evaluate their contribution to this complex disorder we aimed to analyze 182 SNPs across 10 genes that encode neurotrophic factors (NGF, BDNF, NTF3, NTF4/5, and CNTF), and their receptors (NTRK1, NTRK2, NTRK3, NGFR and CNTFR) in a clinical sample of 546 ADHD patients (330 children and 216 adults) and 546 controls by a case-control association study.

Results
After correcting for multiple testing, the multiple marker analysis showed a strong association between the Cyliary Neurotrophic Factor Receptor (CNTFR) and both child (P=9.1e-04; OR=1.4) and adult ADHD (P=0.0077; OR=1.38), and provided evidence of a childhood-specific contribution of Neurotrophin 3 (NTF3; P=3.0e-04; OR= 1.48) and the Neurotrophic Tyrosine Kinase Receptor Type 2 (NTRK2; P=0.0084; OR=1.52) when all ADHD clinical subtypes were taken into account, as well as when the combined or inattentive clinical subgroups were independently analysed.

Discussion
Although independent replication in other sets of patients and family trios are required, the results of our study support the involvement of neurotrophic factors and their receptors in the predisposition to both childhood and adulthood ADHD.

Ring, Susan

Generation Of Lymphoblastoid Cell Line Banks For Genetic Epidemiology.

Karen Ho(1), Patricia Holley(1), Wendy McArdle(1), David Strachan(2), Marcus Pembrey(3,4) and SUSAN RING(1).
(1) Department of Social Medicine, University of Bristol, UK; (2) Division of Community Health Sciences, St George s Hospital Medical School, London, UK; (3) University of Bristol, UK; (4) Clinical and Molecular Genetics Unit, Institute of Child Health, University College London, UK

Recent advances in high throughput genotyping have placed demands on genetic epidemiology studies to supply large amounts of high quality DNA. Lymphoblastoid cell lines (LCL's) are a good source of such DNA since an infinite number of cells can be generated to provide the required yields. DNA quality is also more easily controlled since cell lines provide a uniform starting material for DNA extraction.

In addition LCL's are a valuable phenotypic resource for genetic epidemiology studies. Cells can be grown under controlled conditions and accurate cellular phenotypes and expression profiles generated.

We have created a lymphoblastoid cell line bank as a source of DNA for the 1958 British Birth Cohort (http://www.cls.ioe.ac.uk/Ncds/mainncds.htm and the are in the process of creating a similar resource for the Avon Longitudinal Study of Parents and Children (www.alspac.bris.ac.uk).

Over 25,000 cell lines will be generated and we have determined the optimum sample preparation conditions for establishing cultures and compared manual and robotic feeding techniques. We have also explored the effect on transformation success and cell growth rate of a number of characteristics of the cell donors such as age of individual, sex, and some genotypes.

Rios, Daniel

Extending Ensembl Annotation Data To Include Disease Related Variations

RIOS, DANIEL; Chen, Yuan; Birney, Ewan; Flicek, Paul
European Bioinformatics Institute, Wellcome Trust Genome Campus, Hinxton, Cambridge, CB10 1SD, United Kingdom

The Ensembl (http://www.ensembl.org/) database project provides a bioinformatics framework to organise biology around the sequences of large genomes. It is a comprehensive and integrated source of automatic annotation of large genome sequences, available as either an interactive website or as flat files. As well as being one of the leading sources of genome annotation, Ensembl is an open source software engineering project to develop a portable system able to handle very large genomes and associated requirements.

Technological advances are leading to the widespread availability of multi-species variation data, dense genotype data, and large-scale resequencing projects. The study of human variation has significantly advanced through resources already available from projects such as the HapMap. These data are challenging the conventional genome-wide bioinformatics resources previously used for storage and manipulation of variation data.

To address these challenges within Ensembl, we have designed and tested a database solution and software library designed to support variation data, dense genotyping and resequencing data from thousands of individual genome sequences. These tools include several unique features compared to other systems for variation bioinformatics such as a full-featured API and a novel compression scheme for genotype data.

We demonstrate the power of these resources to enable a deeper understanding of the genotype-phenotype relationship by providing our users with disease-related information that is integrated with other Ensembl resources. We have developed a software infrastructure to import the gene specific mutation data currently available from various locus-specific mutation databases worldwide. The challenges associated with including this data includes reconciling coordinate systems with the human genome reference assembly and supporting multiple LSDB formats. Another set of disease related data we have recently incorporated into the Ensembl variation database is the publicly available version of the Decipher database (http://decipher.sanger.ac.uk/), containing structural variation data mapped to the human genome trying to facilitate the study of the associated phenotype.

Ensembl provides connections from specific gene or disease data to the other publicly available genotypic and functional data resources and provides visualisation routines to help researchers to browse genes and disease related data. For researchers requiring the in-depth data that a specialised database provides, we include links back to the relevant database.

Robledo, Mercedes

Association Study Of 97 Genes Identifies Low Penetrance Loci In Sporadic Papillary Thyroid Carcinoma

Landa, Iñigo(1); Montero-Conde, Cristina(1); Milne, Roger(2); Letón, Rocío(1); Cascón, Alberto(1); Rodríguez-Antona, Cristina(1), Leskelä, Susanna(1); López-Jiménez, Elena(1) ; Ruiz-Llorente, Sergio(3); Santisteban, Pilar(3); González-Neira, Anna(2); Viglietto, Giuseppe(4); ROBLEDO, MERCEDES(1)
(1) Hereditary Endocrine Cancer Group and (2) Genotyping Unit, Human Cancer Genetics Programme, Spanish National Cancer Centre (CNIO), Madrid, Spain; (3) Molecular Endocrinology Department, Biomedical Research Institute (IIB), Madrid, Spain; (4) Dipartimento di Medicina Sperimentale e Clinica .G Salvatore, Università Magna Graecia, Catanzaro, Italy

Papillary Thyroid Carcinoma (PTC), originated from follicular thyroid cells, is the most common malignancy (80%) among thyroid neoplasias. PTC has a sporadic behaviour in approximately 95% of the cases (sPTC), while the remaining 5% has a familial nature. To date, there are four somatic molecular lesions associated with these sporadic tumours: chromosomal rearrangements that affect the RET or TRKA tyrosine kinase receptors and activating mutations of BRAF or RAS oncogenes. All of these genetic alterations activate the MAPkinase signalling pathway and they are mutually excluding.

In spite of this knowledge, PTC still behaves as a complex disease where the individual susceptibility probably is determined by multiple common genetic variants located in different 'low penetrance genes' (LPG). Hence, our purpose was to identify LPG related to PTC susceptibility by a case-control study with representative SNPs of the candidate genes.

We have performed a two-stage case-control study in two European populations using medium-throughput genotyping by using an Illumina platform. We selected a total of 97 candidate genes to analyze: 58 related to thyroid metabolism and 39 due to be differentially expressed by using cDNA arrays when comparing PTC versus normal tissue, follicular, anaplastic and poorly differentiated thyroid carcinomas. The 97 genes selected were well characterized by 768 single nucleotide polymorphisms (SNPs). The SNPs were chosen through NCBI, Ensembl and HapMap databases and *in silico* tools (PupaSuite) to include Tag SNPs and putative functional SNPs when possible.

These SNPs were initially studied in a series of sPTC cases (N=300) and controls (N=300). Associations were assessed by and exhaustive analysis of individual SNPs, haplotypes (considering linkage disequilibrium blocks defined by HapMap Project) and dyplotypes to define PTC susceptibility loci. In stage 2, an independent series of up to 250 sPTC patients and 250 controls was studied to validate the significant results obtained in stage 1.

The results will be presented in the meeting.

Rosenow, Carsten

Construction Of A Comprehensive Whole Genome Analysis Panel Containing Over 1M Variations

C. ROSENOW, K. A. Viaud, L.M. Galver, L. Zhou,, M. Eberle, D. Peiffer, K. Kuhn, R. Shen, and S.S. Murray.
Illumina, Inc., San Diego, CA

We have constructed a whole-genome genotyping panel containing over 1 million SNPs selected to maximize genomic coverage for whole-genome and copy number variation (CNV) studies. This panel utilizes the Infinium® assay that allows interrogation of large number of SNPs and non-polymorphic probes efficiently and accurately on a single slide (e.g. Steemers et al., 2006). The content of this panel focuses primarily on genes, CNV regions, and tag SNPs. Approximately 400,000 SNPs map within 10kb of a gene region covering >99% of RefSeq genes at an average density of 6 SNPs per 10kb. More than 99% of the 2,714 regions of known CNV published in the Database of Genomic Variants are covered by 232,547 SNPs at an average density of 5 SNPs per 10kb. Approximately 38,000 SNPs and 18,000 non-polymorphic probes were added to cover 13,000 novel CNV regions. Tag SNPs on this panel were chosen from all HapMap populations and capture approximately 94%, 93% and 73% of HapMap Phase II variation at r2 e 0.8 in the Caucasian (CEU), Han Chinese/Japanese (CHB/JPT) and Yoruba (YRI) populations, respectively. Additionally 6,000 SNPs and indels were chosen from a high-density MHC map (deBakker et al., 2006), 25,000 nsSNPs were included and 10,000 SNPs selected to cover 200 known ADME genes. Average spacing of SNPs and probes is approximately 2.4 kb across the genome with <5,000 gaps greater than 10kb. This panel of over 1M variants provides a powerful tool for whole genome analysis utilizing both SNPs and structural variation throughout the genome.

Saarela, Janna

A Complex Segmental Duplication Superstructure On Human Chromosome 17q Arm

Daniel Chen(1), Virpi Leppä(2), Timo Miettinen(3), Aarno Palotie(3,4), Leena Peltonen(2,4) and JANNA SAARELA(2)
(1) Department of Human Genetics, David Geffen School of Medicine at UCLA, Los Angeles, California, US; (2) National Public Health Institute, Helsinki, Finland; (3) Finnish Genome Center, University of Helsinki, Helsinki, Finland; (4) The Broad Institute of MIT and Harvard, Cambridge, MA, US

Human Chromosome 17 is implicated in many forms of human neoplasia and genetic diseases caused by chromosomal abnormality, but it is also rich in polymorphic structural variation. Chromosomal instability is one underlying cause of these manifestations. A major mechanism resulting in chromosomal instability is the non-allelic homologous recombination of two highly similar regions. We and others have shown that the chromosome 17 is significantly enriched with inter- and intra-chromosomal segmental duplications.

We conducted a genome-wide sequence alignment using expressed transcripts mapping within the previously identified duplicated domains on 17q22-q24 to determine the full extent and architecture of the duplicated segments on chromosome 17q. This analysis identified a duplication superstructure (SDS) on 17q arm, consisting of 13 discrete domains, which share sequence homology. Four of the duplication domains contain sequence elements homologous to every other domain of the superstructure. A similar duplication structure was found on the syntenic chimpanzee chromosome. We further identified retrotransposable sequence elements to be highly associated with the duplication superstructure with density of over 100 sequence copies per 1Mb within the duplication superstructure compared to 11 sequences per 1Mb on non-duplicated parts of 17q.

We listed all retrotransposable mRNAs (n=257) on chromosome 17q and mapped them electronically to the human genome. A total of 781 unique positions on chromosome 17q arm were observed to contain full or partial copies of these elements. The average number of copies per retrotransposable mRNA was three, but the 10% most highly copied mRNAs had on average 13 copies within 17q (n=7-28). These highly-copied elements could be divided into two groups: A. Fourteen of these elements were significantly associated with the duplication superstructure, and mapped almost exclusively within the SDS; B. Eleven elements mapped mostly outside the superstructure. The group A elements (n=14) were associated also with the chimpanzee 19q duplication superstucture, and we observed at least two partial copies of them at two discrete positions in the syntenic dog chromosome. We further studied the expression of three of the SDS-associated retrotransposable mRNAs (group A: AK125814, AK000982 and AF119889) in human and chimp PBMC samples using specific primers for each retrotransposable element. The RT-PCR and subsequent sequence analyses revealed expression of multiple discrete copies, with differences in the expression between individuals.

To summarize, this analysis identified a duplication superstructure on 17q arm, consisting of 13 discrete domains, which all share sequence homology. A parallel superstructure was found on the syntenic chimpanzee chromosome. Such a superstructure may predispose to the chromosomal instability observed on chromosome 17q: the 13 homologous domains create a high number of potential sequence annealing sites for non-allelic homologous recombination events. We further identified actively transcribed retrotransposable sequence elements to be highly associated with the duplication superstructure on human chromosome 17q and the syntenic chimpanzee chromosome. These observations suggest a potential link between transcription, duplication and rearrangements.

Sasvari-Szekely, Maria

Unbalanced Gene Copy Number Of Complement C4A And C4B: Medical Aspects

SASVARI-SZEKELY, MARIA(1); Szantai, Eszter(1); Janka, Zoltan(2) and Fust, George(3).
(1) Inst. Medical Chemistry, Molecular Biology and Pathobiochemistry and 3 The 3rd Dept. of Internal Medicine, Semmelweis University, Budapest; (2) Dept. of Psychiatry, Univ. of Szeged, Hungary

Human complement C4 gene is a part of the RCCX module in the class III region of the Major Histocompatibility Complex (MHC) on chromosome 6 with a gene copy number of 2 to 6. In addition to the gene copy number variation, the C4 gene has two isoforms (C4A and C4B) encoding for slightly different proteins. Any of the C4 gene copies can be either C4A or C4B, however, the most frequent haplotypes of the RCCX module possesses one copy of C4A and C4B genes on each of the homologous chromosomes. Unbalanced ratio of the isoforms have been shown to result in unbalanced expression of the complement isoforms and was associated with a variety of diseases, as well as a shorter life-span both in a Hungarian population[1] and on Iceland[2] .

We previously developed a novel RT-PCR method for quantization of C4A and C4B genes in the human population as a substitution of the classic Southern-blot method. Application of the novel methodology helped us to reveal further behavioural and medical aspects of the unbalanced C4A/B gene copy number. Lower C4B than C4A gene dosage is traditionally labelled as C4B*Q0. Our recent results suggested that the decreasing percentage of the C4B*Q0 carriers in healthy elderly population might be the consequence of a higher risk of chronic disabling diseases in interaction with smoking[3,4]. Moreover, we recently found an association between C4AQ0 (lower C4A gene copy number) and the risk of schizophrenia in a case-control study.

As the studied gene region is a part of an extended haplotype within the MHC, involving numerous genes in a strong linkage disequilibrium, the above enlisted risk factors might also be related with other gene variants in this region, rather that to the C4 gene polymorphism itself. Studies are in progress to define a more precise haplotype structure of the RCCX module by RT-PCR, as well as to identify further polymorphic genes in this region and their relation to risk factors of various diseases.

References:
1. Kramer J, Fulop T, Rajczy K, Nguyen AT, Fust G. A marked drop in the incidence of the null allele of the B gene of the fourth component of complement (C4B*Q0) in elderly subjects: C4B*Q0 as a probable negative selection factor for survival. Hum Genet. 1991 Apr;86(6):595-8.
2. Arason GJ, Bodvarsson S, Sigurdarson ST, Sigurdsson G, Thorgeirsson G, Gudmundsson S, Kramer J, Fust G. An age-associated decrease in the frequency of C4B*Q0 indicates that null alleles of complement may affect health or survival. Ann N Y Acad Sci. 2003 Dec;1010:496-9.
3. Szilagyi A, Blasko B, Szilassy D, Fust G, Sasvari-Szekely M, Ronai Z. Real-time PCR quantification of human complement C4A and C4B genes. BMC Genet. 2006 Jan 10;7:1.
4. Blasko B, Szeplaki G, Varga L, Ronai Z, Prohaszka Z, Sasvari-Szekely M, Visy B, Farkas H, Fust G. Relationship between copy number of genes (C4A, C4B) encoding the fourth component of complement and the clinical course of hereditary angioedema (HAE).
Mol Immunol. 2007 Apr;44(10):2667-74.
5. Arason GJ, Kramer J, Blasko B, Kolka R, Thorbjornsdottir P, Einarsdottir K, Sigfusdottir A, Siguretharson ST, Sigurethsson G, Ronai Z, Prohaszka Z, Sasvari-Szekely M, Boethvarsson S, Thorgeirsson G, Fust G. Smoking and a complement gene polymorphism interact in promoting cardiovascular disease morbidity and mortality. Clin Exp Immunol. 2007 Apr 11; [Epub ahead of print]

Saus, Ester

Comprehensive Copy Number Variant (CNV) Analysis Of Neuronal Pathways Genes In Psychiatric Disorders

ESTER SAUS(1), Anna Brunet(1), Mònica Gratacòs(1,2), Juan Ramón González(1), Lluís Armengol(1) and Xavier Estivill(1,2,3) on behalf of the Psychiatric Genetics Consortium
(1) Genes and Disease Program and (2) CIBERESP, Center for Genomic Regulation (CRG); (3) Pompeu Fabra University, Barcelona, Catalonia

A Copy Number Variation (CNV) is defined a DNA segment that is one kilobase or larger and present at variable copy number in comparison with a reference genome. Because CNVs can confer risk to complex disease traits, their study in psychiatric disorders is of great interest. The aim of this work is to perform a comprehensive screening of CNVs in different groups of psychiatric patients. The sample analyzed consisted of 170 patients of each group: affective disorders, eating disorder, anxiety disorders and schizophrenia, as well as 170 control individuals. Based on the Central Nervous System transmitter systems, we selected 364 genes involved in neuronal pathways, including metabolyzing enzymes, receptors, transporters, proteins interacting with the transmitter receptors and proteins involved in its signal transduction. We used the Database of Genomic Variants to identify genes predicted to be in CNVs. We designed four Multiple Ligation Probe Amplification (MLPA) assays to detect variations in copy number between patients and controls. The results were analyzed with MLPAstats, a new package from R software, developed by our group.

Seventy-five genes were included in the analysis. CNVs were detected for forty-eight genes. We did not find significant differences between case and control samples when single genes were analyzed. When comparing the total number of gains and losses in psychiatric patients vs. control subjects, we found that controls tend to carry a higher number of CNVs than psychiatric patients.

These results indicate that a considerable proportion of neuronal pathways genes contain CNVs. Although the involvement of these CNVs in psychiatric disorders is unclear, the analysis of a larger sample and a wide range of psychiatric phenotypes should permit to define their potential role in these complex disorders.

Schott, Jean-Jacques

Long QT Syndrome Caused By Large Deletions Including The KCNH2 (HERG) Gene Undetectable By Current Polymerase Chain Reaction-Based Exon-Scanning Methodologies

Schmitt Sébastien(1,2), Le Cunff Martine(1), Vieyres Claude(3), Cédric Le Caignec(1,2), Kyndt Florence(1,2), Hervé Le Marec(1,4), Probst Vincent(1,4), SCHOTT JEAN-JACQUES(1,2)
(1) Inserm U533, l'institut du thorax, Faculté de médecine Nantes, France
(2) Laboratoire de génétique moléculaire, CHU de Nantes, France
(3) Cabinet de cardiologie Angoulême, France
(4) Clinique cardiologique, l'institut du thorax, CHU de Nantes, France

Long-QT syndrome (LQTS) is an inherited cardiac arrhythmia characterized by a prolonged QT interval on the surface ECG associated with syncope and sudden death from torsades de pointes polymorphic ventricular tachycardia. It is estimated to affect 1 in 5000 individuals. LQTS may be caused by mutations in 4 major genes encoding K+ channels pore forming (KCNQ1, KCNH2) and auxiliary (KCNE1, KCNE2) subunits and in the gene encoding the cardiac specific Na+ channel SCN5A. Genetic testing for congenital LQTS has been performed in research laboratories for the past decade. Approximately 75% of patients diagnosed with LQTS have mutations in one of five LQTS genes. Possible explanations for the remaining genotype-negative cases include LQTS mimickers, novel LQTS-causing genes, unexplored regions of the known genes, and genetic testing detection failures.

The purpose of this study was to determine the relative copy number in the 5 major LQTS genes in a series of 100 mutation-negative LQTS probands. A multiplex ligation-dependent probe amplification (MLPA) approach was used using SALSA P114 MLPA kit (MRC Holland, Amsterdam, The Netherlands). Aberrant exon copy numbers were confirmed by Quantitative Multiplex PCR of Short Fluorescent Fragment (QMPSF). Array based CGH analysis was performed using Agilent Human Genome 244K Micro arrays to further map the genomic rearrangements. Whenever possible, familial investigation was performed to test segregation of the copy number variation.

This study identified two large deletions in KCNH2 gene in 2 probands. A KCNH2 deletion spanning exon 4 to 14 was identified in a 20 years old symptomatic woman with QTc > 554 milliseconds. QMPSF analysis of exon 15 identified a deletion of the last KCNH2 exon. CGH analysis identified a much larger genomic deletion of 650 Kb deletion including KCNH2 exon 4 to 15 but also 19 additional genes downstream of KCNH2 including the amiloride binding protein 1 gene (ABP1) and a cluster of genes encoding immunity-associated proteins (GIMAPs). Familial and clinical investigations identified 3 additional affected individuals with a KCNH2 deletion. A second large deletion was identified in a second proband with a QTc= 478 milliseconds who experienced syncope after emotional stress. MLPA analysis identified the deletion from exon 1 to 14 and exon 15 QMPSF analysis confirmed full length deletion of KCNH2 gene. Using Agilent array CGH 244 K, the size of the deletion was estimated to be 145 Kb. 5' breakpoint maps between KCNH2 and NOS3 genes intergenic region whereas 3' breakpoint maps after the ABP1 gene. No familial investigation was possible since the proband was adopted. Both genomic deletions were not found in 100 controls and no deletions or duplications were identified in the KCNQ1, KCNE1, KCNE2 or SCN5A genes. Both deletions are expected to be non functional thereby decreasing IKr current in ventricular cardiomyocytes suggesting haploinsufficiency as the most likely mechanism leading to LQTS in both probands and relatives. Further clinical investigations are currently underway to investigate for additional phenotypes in all patients.

Despite a quit limited number of LQTS probands screened in this study, the identification of 2 large deletions in LQTS genes strongly suggests screening for copy number variant in mutation negative LQTS probands.

Sethumadhavan, Rao

Identification And In-Silico Analysis Of Functional SNPs Of BRCA1 Gene

Rajasekaran. R, Sudandiradoss. C, George. C, RAO SETHUMADHAVAN
School of Biotechnology, Chemical and Biomedical Engineering, Vellore Institute of Technology University, Vellore 632014, Tamil Nadu, India

Single nucleotide polymorphism (SNPs) play a major role in understanding the genetic basis of many complex human diseases. Also, genetics of the human phenotype variation could be understood by knowing the functions of these SNPs. It is still a major challenge to identify the functional SNP's in a disease related gene. We have reported the results breast cancer of BRCA1 gene functional SNPs in this work. Out of the total 477 SNPs, 65 were found to be non synonymous (nsSNPs). Among the14 SNPs, 4 SNPs were found in 5' and 10 SNPs were found in 3' un-translated regions (UTR). It was found that 16.9 % nsSNPs were found to be damaging by both SIFT and PolyPhen server. 50 % and 30 % of 5' and 3' UTR regions were found to be deleterious by our study. We identified that major mutations were of proline to serine at the position 1776 and 1812 of the native protein of BRCA1 gene. We have also modeled the structure for both the mutant proteins and have compared with the native protein.

From a comparison of the stabilizing residues of the native and mutant protein, we propose that the mutation at position 1812 (nsSNP rs1800757) could be a possible mutation candidate responsible for the cause of breast cancer by BRCA1 gene.

Sigurdsson, Snaevar

Polymorphisms In The Interferon Regulatory Factor 5 Gene Are Associated With Systemic Lupus Erythematosus And Rheumatoid Arthritis

SIGURDSSON, SNAEVAR(1); Padyukov, Leonid(3); Klareskog, Lars(3); Alm, Gunnar(2); Rönnblom, Lars(2); Syvänen, Ann-Christine(1)
(1) Molecular Medicine and (2) Section of Rheumatology, Department of Medical Sciences, Uppsala University, Uppsala, Sweden; (3) Rheumatology Unit, Department of Medicine, Karolinska University Hospital, Stockholm, Sweden.

The type I interferon (IFN) system is activated when self-tolerance is broken and autoimmune reactions develop. It may play a key role for the development of autoimmune diseases, such as Systemic Lupus Erythematosus (SLE), which is considered to be the prototype autoimmune disease. The IRF5 gene is a transcription factor that is activated by the Toll-like receptors 7, 8 and 9, which are involved in the induction of IFN-± in human cells. We have previously identified SNPs in the promoter and first intron of the interferon regulatory factor 5 (IRF5) gene that are associated with SLE in patients from the Nordic countries (Sigurdsson et al. Am J Hum Genet 76:528, 2005). This finding has been convincingly replicated in other populations (Graham R et al. 2006, Nat Genet 38, 550-555.) We have performed an association study of these SNPs in a set of Swedish samples from 1530 Rheumatoid Arthritis (RA) patients and 881 age, matched controls from (EIRA) collection (Padyukov et al. Arthritis Rheum 50:30854, 2004). We found that the same SNPs in the IRF5 gene that are associated with SLE, also are associated with RA, with the strongest significance with anti-cyclic citrullinated peptide (anti-CCP) antibody-negative RA. The same IRF5 haplotype formed by the minor alleles of the IRF5 SNPs appear to be protective against the disease both in SLE and RA (Sigurdsson et al. 2007, Arthritis Rheum. 56).
For a complete screen of genetic variants of the IRF5 gene, we sequenced all exons, introns and the promoter region in SLE patients and controls. We identified several new SNPs and indels that define strong risk and protective haplotypes in SLE patients. Three different potential functional variants have been identified. The risk haplotype contains the allele of rs2004640 that creates splice site for alternative exon 1, a common 30 bp insertion in exon 6, and the allele of rs10954213 that increases the expression of the IRF5 gene. (Graham R. et al 2007, PNAS 104:6758). This pattern of multiple common causal alleles may have general implication for genetic studies of complex diseases.

Sikora, Martin

Evolutionary Analysis Of Genes Involved In The Biosynthesis Of Chondroitin Sulfate And Hyaluronic Acid For Their Possible Implication In Malaria

MARTIN SIKORA, Ferran Casals, Anna Ferrer, Jaume Bertranpetit Unitat de Biologia Evolutiva, Universitat Pompeu Fabra, Barcelona, Catalonia, Spain

Recent times have seen a new interest in the study of natural selection in human populations, with a range of new methods available to detect signatures of selection in the human genome. We present here the results of a study employing this methodology to identify functional variation in 24 genes in the biosynthesis pathway of two receptor molecules thought to be involved in human-pathogen interaction, chondroitin sulfate and hyaluronic acid. Analysis of publicly available polymorphism data from the International HapMap project for three human populations resulted in the identification of three candidate genes showing signatures of selection. One of those candidates, XYLT1, shows particular suggestive evidence in each of the applied analysis. We demonstrate the utility of this approach to identify regions of interest in the study of human infectious disease.

Skol, Andrew

A Comparison Of Allele Frequencies Estimates In 6,174 SNPs Genotyped In HapMap And Seattle SNPs

ANDREW D SKOL(1) and Michael L. Feolo(2)
(1) Section of Genetic Medicine, Department of Medicine, University of Chicago; (2) National Center for Biotechnology Information, National Library of Medicine, National Institutes of Health

We examined the fidelity of genotype data obtained through sequencing by comparing allele frequencies for a set of 6,174 SNPs genotyped by both Seattle SNPs (SS) and HapMap (HM). Seattle SNPs genotypes were obtained by targeted sequencing of genic regions in 22 or 23 individuals of European decent (CEPH Utah individuals). HapMap genotype data was generated from probe-based genotyping of the 60 CEPH Utah founders. A subset of the subjects was genotyped in both groups on 5,128 SNP, allowing us to directly test for genotyping inconsistencies. The number of overlapping subjects varied, but ranged between 4 and 23 individuals depending on the panel genotyped by SS.

Of the 6,174 SNPs, 2,932 were polymorphic in at least one of the sample. We identified 103 SNPs (1.7%) for which at least one individual's genotype was called differently by HM and SS. Of these, 68 SNPs have a single discrepant subject, 19 SNP have two discrepant subjects, and 16 have three or more discrepancies. Of the 38,458 opportunities to detect discrepancies, we found only 258 discrepant genotypes (0.7%). We tested for allele frequency differences between the SS and HM samples using a standard Chi-squared test of independence. After looking at the distribution of p-values, which we expect to be uniformly distributed between 0 and 1, we found an excess of significant results at the .0001 and .001 levels.

We also discovered that of the 103 SNP with at least one discrepant genotype, 24 are monomorphic in HM, but have at least one heterozygous SS individual. The converse occurs only 5 times. When there are no genotype discrepancies this imbalance is not observed. We determine empirically if heterozygotes are being overcalled by sequencing or undercalled by conventional genotyping by examining the linkage disequilibrium pattern between the SNP in question and those in the surrounding region.

In summary, we have found that the overall data quality of both sequence data from Seattle SNPs and genotype data from HapMap is of very high quality. A small proportion of polymorphic SNPs may appear monomorphic when using conventional genotyping products due to the presence of a SNP in the primer sequence or due to technical issues in the genotype calling algorithm. As more genes are resequenced such SNPs can be identified, allowing either the redesign of primers or substitution of a SNP in linkage disequilibrium with the problem SNP.

Sobrino, Beatriz

Genetic Basis Of Age-Related Macular Degeneration (AMD)

Brión M(1), de la Fuente M(2), SOBRINO B(1), Carracedo A(1), Sánchez-Salorio M(2), Multi-centric group of AMD.
(1) Grupo de Medicina Xenomica, CEGEN-IML, University of Santiago de Compostela, Spain; (2) Instituto Galego de Oftalmoloxía (INGO), Santiago de Compostela, Spain

Age-related macular degeneration (AMD) is the most frequent cause of lost of vision in the elderly in developer countries. The molecular basis of the disease is not well known and the diagnosis of both types of AMD, 'geographic atrophy' (GA) and choroidal neovascularization (CNV), has been made based on the presence of phenotypic signs related to the disease, which are also used to characterize its severity and to tailor treatment for advanced stages of the disease. The prevailing view is that AMD is a complex disorder stemming from the interaction of multiple genetic and environmental risk factors. Familial aggregation studies indicate that a genetic contribution can be identified in up to 25% of the cases.

To examine the genetic basis of AMD we conducted an association study in 360 index cases and 360 controls. Family and medical history was obtained from index cases, and samples were recovered with uniform geographic criteria, by the multi-centric group of AMD. A total of 387 SNPs distributed among 55 candidate genes were analysed. Genes have been carefully selected from the literature, including genes that have been previously associated with the disease by other groups and new candidate genes not analysed before, but prone to be implicated in the development of the disease.

Genotyping was performed with SNPlex technology (Applied Biosystems) and results were processed to calculate basic statistical parameters and to test for possible genetic association with any of the different forms of the disease.

As a result, we have replicated the positive association with CFH and LOC387715; but in addition, FGF2 also appear as candidate gene responsible for AMD, and particularly for Athrophic AMD.

Song, Kyuyoung

Association Of TNFSF15 With Crohn's Disease In Koreans

Suk-Kyun Yang(1); Jiyoung Lim(2); Hye-Sook Chang(1); Inchul Lee(3); Li Yuqing(4); Jianjun Liu(4); KYUYOUNG SONG(2)
Departments of (1)Internal Medicine, (2)Biochemistry and Molecular Biology, (3)Pathology, Asan Medical Center, University of Ulsan College of Medicine, Seoul, Korea; (4) Human Genetics Group, Genome Institute of Singapore, Singapore

Recent genome-wide association studies identified additional IBD genes including TNFSF15 (Yamazaki et al. 2005) and IL23R (Duerr et al. 2006). Of these, the TNFSF15 variants contributed to the susceptibility to CD not only in European populations but also in Japanese. Based on the report of differences in genetic backgrounds for IBD between Asian and Western countries, we set out to test whether the TNFSF15 gene is associated with susceptibility to CD in a closely related population. Four SNPs across TNFSF15 were genotyped in 380 patients with CD and 380 healthy controls. Carriers of three polymorphisms including rs3810936, rs6478108, rs7848647 showed statistically significant association with CD (aOR = 2.81, 95 % CI = 1.94-4.07, P = 4.4 x 10-8, aOR = 3.49, 95 % CI = 2.42-5.04, P = 2.7 x 10-11, aOR = 3.49, 95 % CI = 2.42-5.03, P = 2.2 x 10-11 respectively). Following haplotype analysis, homozygotes carrying two copies of the haplotype consisted of the risk alleles of those three SNPs showed statistically significant association with CD (adjusted OR = 5.39, 95 % CI = 3.19-9.10, P = 3.07 x 10-10). Our data supports the hypothesis that the TNFSF15 genotypes play an important role in the pathogenesis of CD in Koreans.

Spielman, Richard

Linkage And Association Between Polycystic Ovary Syndrome And Markers On Chromosome 19p13.2

R.S. SPIELMAN(1), D.R. Stewart(2), B.A Dombroski(1), W. Ankener(1), M. Urbanek(3), K.G. Ewens(1), R.S. Legro(4), J.F. Strauss III(5), A. Dunaif (3)

(1) Department of Genetics, University of Pennsylvania School of Medicine, Philadelphia, Pennsylvania 19104; (2) NHGRI, NIH, Bldg 49, Bethesda, Maryland 20892-4472;
(3) Division of Endocrinology, Metabolism, and Molecular Medicine, Northwestern University Medical School, Chicago, Illinois 60611;
(4) Department of Obstetrics and Gynecology, Pennsylvania State University, Hershey, Pennsylvania 17033;
(5) Dean, School of Medicine, Virginia Commonwealth University, Richmond, Virginia 23298

Polycystic ovary syndrome (PCOS) is an endocrine disorder that occurs in 4-5% of reproductive-age women, and shows familial aggregation. Using the transmission/disequilibrium test (TDT), we found strong evidence for linkage and association with a microsatellite marker (D19S884) located approximately 1 Mb from the insulin receptor gene (INSR) on chromosome 19p13.2. We have since added two independent samples that extend the original findings. In the three sets of families (2100 individuals in 465 families), the transmission ratio of the associated allele is practically identical (0.61, 0.61, 0.64), and the P-value for the combined material is $P < 0.00007$.

Preliminary fine mapping of the region was not able to specify the genetic element or gene that is responsible for PCOS, and it is not clear how susceptibility is conferred. In order to resolve these issues, we have carried out reporter assays to test for regulation of gene expression by DNA elements in the D19S884 region. We first constructed an 800-bp fragment containing the region of interest and evaluated its function with standard luciferase assays adapted to use in immortalized B-lymphocytes (LCLs). In these experiments, we found that: 1. The fragment containing D19S884 has substantial, reproducible, promoter activity, equal to approximately 20% of SV40 promoter activity in this same system. 2. There is no significant difference between the luciferase activity conferred by predisposing (PCOS-associated) and non-predisposing alleles of the marker. In follow-up, we tested 22 SNPs in the region, and considered the association with haplotype blocks. Although these blocks refine the association, D19S884 remains the marker with the most significant evidence for association with PCOS. It is likely that variation at D19S884 affects expression in vivo of some closely linked gene(s) in appropriate tissues.

Stankovic, Marija

Association Of GSTM1 And MMP9 Gene Variants As A Risk Factor For COPD Development

MARIJA STANKOVIC(1), Ljiljana Rakicevic(1), Andrija Tomovic(2), Natasa Petrovic-Stanojevic(3), Marina Andjelic(3), Vesna Dopudja-Pantic(3), Dragica Radojkovic(1)
(1) Institute of Molecular Genetics and Genetic Engineering, Belgrade, Serbia; (2) Friedrich Miescher Institute for Biomedical Research, Basel, Switzerland; (3)Zvezdara University Medical Center, Belgrade, Serbia

Chronic Obstructive Pulmonary Disease (COPD) is characterized by the progressive development of airflow limitation that is not fully reversible. Smoking is regarded as the most important causal factor, but only minority of smokers develop symptomatic COPD, which indicates that a difference in susceptibility to tobacco smoke injury might be related to genetic factors. It is likely that multiple genetic factors interact with each other and with environmental factors resulting in pathophysiological heterogeneity observed in COPD.

There are two major hypothesis in the smoking related COPD. One is oxidant-antioxidant hypothesis which proposes that oxidant stress, resulting from oxidant-antioxidant imbalance, have important consequences for the pathogenesis of COPD [1]. The glutathione S-transferase (GST) supergene family encodes isoenzymes that appear to be critical in protection against oxidative stress by detoxifying various toxic substrates in tobacco smoke. The other hypothesis is the proteinase-antiproteinase which states that various proteinases break down connective-tissue components, which influences lung parenchyma destruction and inflammation in COPD [2]. Matrix metalloproteinases (MMPs) are a family of proteolytic enzymes, which play an essential role in tissue remodeling and repair.

Different genetic variants of genes that are involved in metabolism of toxic substances from cigarette smoke and/or extra-cellular matrix remodeling may alter or modulate processes that could lead to initiation and progression of COPD pathogenesis. In order to examine a role of functional gene variants of GSTM1, GSTT1, GSTP1, MMP1, MMP9 and MMP12 genes in COPD development an association case-control study was designed.

Genotyping of 86 COPD patients and 102 control subjects was performed using: multiplex polymerase chain reaction for GSTM1 null and GSTT1 null genotype, restriction fragment length polymorphism for GSTP1 Ile105/Val105, MMP9 C-1562T, MMP12 A-82G polymorphisms, while MMP1 G-1607GG gene variant was detected by conformation sensitive gel electrophoresis. Odds Ratios (ORs) and 95% Confidence Intervals (95%CIs) for associations between genotypes and COPD status were calculated by binary logistic regression model. A p-value of less than 0.05 was considered significant. Statistical analysis was performed using SPSS software.

None of tested polymorphisms had statistical significance between COPD patients and controls when studied separately. Statistical significance was observed when tested combination of homozygote for GSTM1 null and heterozygote for MMP9 C-1562T variant in COPD group in comparison with control subjects (24.4% versus 11.7%, OR= 2.4, p=0.026).

The MMP9 -1562T promotor polymorphism is connected with higher transcriptional activity and consequently increased extracellular matrix protein degradation. It has been shown that oxidant stress may increase MMP9 activation, which in case of GSTM1 deficiency may present an additional mechanism for even higher expression of MMP9 than that -1562T allele would have by itself.

According to our results association of GSTM1 null/null and MMP9 -1562CT genotypes might be involved in smoking-related COPD development. We also demonstrated the importance of association analysis of xenobiotic metabolism and extracellular matrix remodeling gene polymorphisms. That could result in increased oxidative stress and lung parenchyma destruction, which are crucial characteristics of COPD pathogenesis.

1. He J, Ruan J, Connett JE, Anthonisen NR, Paré PD, Sandford AJ. Antioxidant gene polymorphisms and susceptibility to a rapid decline in lung function in smokers. Am J Respir Crit Care Med. 2002;166:323-328.
2. Joos L, He J, Shepherdson MB, Connett JE, Anthonisen NR, Paré PD, Sandford AJ. The role of matrix metalloproteinase polymorphisms in the rate of decline in lung function. Hum Mol Genet. 2002;11:569-576.

Stepanov, Vadim

Genetic Variability Of Inflammatory Immune Response Genes In North Eurasia: The Climatic Influence

V. A. STEPANOV(1), S. Khoo(2), J. Bizzintino(2), C. McLean(2), H. Clifford(2), I. Laing(2,3), C. Hayden(2), P. Candelaria(2), J. Goldblatt(2), A. Bittles(4), M. Black(4), V. Puzyrev(1), P. N. LeSouef(2,3);
(1) Institute for Medical Genetics, Tomsk, Russian Federation; (2) School of Paediatrics and Child Health, University of Western Australia, Princess Margaret Hospital, Perth, Australia; (3) Institute for Child Health Research, Subiaco, Australia; (4) Centre for Comparative Genomics, Murdoch University, Perth, Australia.

Inflammatory immune responses play a crucial role in the interaction of humans with the environment, and genes which mediate immunity in humans are a possible target for natural selection during human evolution and migrations. The aim of this study was to estimate the genetic diversity of immune response-related genes in populations living under either moderate or Arctic climatic conditions in North Eurasia and to compare it with genetic variability in populations from tropical and subtropical regions. Ten populations, 3 Eastern European (2 Russian, 1 Komis), 2 Central Asian (Kirghiz), 5 Siberian (2 Buryat, 2 Altay, 1 Khant) were genotyped for SNPs in genes involved in Th2 response (CD14 C-159T, SCGB1A1 A38G, CMA1 A-1903G, ADRB2 Arg16Gly, ADRB2 Glu27Gln).

Allele frequencies of the CD14 and CMA1 genes showed significant departure from selective neutrality expectations in most of study populations. A high total level of genetic diversity in inflammatory immune response genes was found in the North Eurasian populations. Irrespective of ethnic and linguistic affiliation and geographical location, 9 of the 10 populations were characterized by high average expected heterozygosity (0.45-0.49). The degree of genetic differentiation of North Eurasian populations exhibited by Th2-related genes was relatively low (Fst=2.4%) compared to ‘neutral’ markers (autosomal Alu Fst=6%, Y-chromosome Fst=18%). The patterns of genetic relationships between populations revealed by phylogenetic analysis seemed not to correspond with those obtained for 'neutral' genetic systems (Alus, Y-chromosome, mtDNA). In general, a significant correlation of 'proinflammatory' allele frequency and total genetic diversity with geography and climate was revealed. The consistent picture of decreasing of 'proinflammatory' allele frequency and increasing genetic diversity with increasing severity of climate was found.

These data may indicate for selective advantage of 'proinflammatory' allele in tropical climate due to increased genetic capacity for stronger and more intense Th2 immune responses, and the change of genetic profile with respect to immune system during human settling and adaptation to temperate and Arctic climate regions.

Sultan, Marc

Gene Expression Variation In Down Syndrome Mice Allows To Prioritize Candidate Genes

MARC SULTAN(1), Ilaria Piccini(1), Daniela Balzereit(1), Ralf Herwig(1) Nidhi G. Saran(2), Hans Lehrach(1), Roger H. Reeves(2), Marie-Laure Yaspo(1).
(1) Max Planck Institute for Molecular Genetics, Berlin, Germany; (2) Johns Hopkins Univ. School of Medicine, Baltimore. USA.

Down syndrome (DS), or trisomy 21 affects ca. 1/700 newborns, and is a highly complex developmental disorder showing a constellation of clinical signs with various occurrence and severity among patients. The identification of molecular mechanisms responsible for DS has thus been a high priority for genome research, motivating a large fraction of the molecular analysis of chromosome 21. Until now, the hunt for DS candidate genes has not been conclusive. We argue here that normal variation of gene expression in the population contributes to the heterogeneous DS clinical picture, and we estimated the amplitude of this variation for trisomic genes in Ts65Dn, a mouse model of DS. We analyzed the expression levels of 50 mouse orthologs of chromosome 21 genes in brain regions of eight Ts65Dn and eight euploid mice by real-time PCR. On pooled RNAs, we confirmed previous studies reporting that trisomic vs. euploid (Ts/Eu) gene expression ratios were close to 1.5 fold. However, we observed that inter-individual gene expression levels spanned a broad range of values helping to identify three categories of genes in the cerebellum, cortex and midbrain: 1) genes with expression levels consistently higher in Ts65Dn than in euploids (9, 17 and 7 genes, respectively) 2) genes whose expression levels partially overlap between the two groups (10, 9, and 14 genes respectively) and 3) genes with intermingled expression precluding to differentiate trisomics from euploids (12, 5 and 9 genes respectively). Data show that three genes from the first category (App, Cbr1 and Mrps6) are common to the three tissues highlighting them as strong candidates for the constant trisomy phenotypes. This is the first analysis addressing inter-individual gene expression levels as a function of trisomy. Based on this information we proposed a stategy allowing the discrimination between genes that are candidates for the constant features of DS and those which are likely to contribute for the partially penetrant signs of the disease.

Tam, Paul

Genome-Wide Association Analysis Of Hirschsprung's Disease

PAUL, TAM(1); Clara, Tang(2); Maria-Mercè, Garcia-Barcelo(1); Stacey S, Cherny(2,3); Pak C, Sham(2,3)
(1) Department of Surgery, (2) Department of Psychiatry, (3) Genome Research Centre, Li Ka Shing Faculty of Medicine, The University of Hong Kong, Hong Kong SAR, China.

Hirschsprung's disease (HSCR, aganglionic megacolon) is a developmental disorder characterised by the absence of the enteric ganglia along a variable length of the intestine. HSCR exhibits significant clinical and genetic heterogeneity and has greater prevalence in males. Its incidence varies among populations, being more frequent in Asians (2.8 per 10,000 life births). HSCR mostly presents sporadically although it can be familial (H20%). The gene encoding a receptor tyrosine-kinase (RET) is the major HSCR gene although coding sequence mutations (CDS) only account for 7%-35% of the sporadic and up to 50% of the familial HSCR cases. Reduced penetrance of RET CDS mutations, lack of genotype-phenotype correlation, and a highly frequent low penetrance locus in RET intron 1, indicates that the disease likely results from the interaction of several yet unknown susceptibility loci. A major feature of phenotypes with a complex pattern of inheritance is the segregation of multiple predisposing loci, with cumulative effects. HSCR is as an oligogenic entity being genetically dissected and used as a paradigm for the study of polygenic/complex diseases. To find additional HSCR loci, two independent genome-wide screenings on Caucasian and Chinese populations are being carried out on the International HSCR Consortium set of patients. Initial data obtained from genotyping 72 Chinese HSCR trios using the GeneChipMapping 500K Set (Affymetrix) revealed suggestive susceptibility loci on 7q31.2, 5q34, 18q12.2, and Xq27.3 chromosomal regions in addition to 10q11.2 (RET). Analyses of interactions among these loci and a follow up on the 240 most significant SNPs on 192 HSCR cases and 192 will be presented and discussed.

Taudien, Stefan

Beta-Defensin Genes On Human 8p23.1 Show High Level Of Concordance In Their Copy Numbers

Marco Groth(1), STEFAN TAUDIEN(1), Anders O.H. Nygren(2), Jan P. Schouten(2), Klaus Huse(1) and Matthias Platzer(1)
(1) Genome Analysis, Leibniz Institute for Age Research - Fritz Lipmann Institute, Jena, Germany; (2) MRC Holland b.v. Amsterdam, Netherlands

Copy number variations as an important feature of genome's dynamics pose an exceptional challenge to the availability and reliability of individual copy number estimation methods. Among these, realtime PCR approaches [1] and paralogous sequence quantifications [2-4] are the most commonly used techniques.
Up to now, at the beta defensin (DEF) gene cluster locus on human chromosome 8p23.1, encompassing ten different genes and pseudogenes, individual copy numbers of DEFB4, DEFB103A, DEFB104 and HSPDP3, respectively, were independently determined [1,3-8]. These copy numbers are generally taken as representative for the copy numbers of the entire cluster, spanning at least 185 kb, i.e. assuming concordance of copy numbers for all genes in this region. However, copy number estimation of selected genes within a copy number variable gene cluster by the mentioned methods in the strictly sense do not provide reliable information whether and to which extent the whole cluster is multiplied. E.g., there is no correlation of the presence or absence of the DEFA3 gene within the nearby cluster of alpha defensin genes and the copy number of DEFA1. Furthermore, it has been described that the number of individual beta defensin genes vary within one individual genome [7].
Thus, as a check for concordance, we applied the Multiplex Ligation-Dependent Probe Amplification method (MLPA) [9] to ten DEF cluster genes of 22 individual DNAs. We compared the MLPA probe signals of the DEF cluster genes to those of 13 single copy genes genes flanking the cluster at both 8p23.2 and 8p22.
For every DEF cluster gene, we calculated the ratio of its signal intensity to the average of those from the flanking genes. These ratios were found to be invariant for the same individual DNA (SD 0.11), clearly indicating concordance for the signals and therewith for all of the genes in the DEF cluster. Furthermore, the ratio varies between different individuals, reflecting the copy number differences of the gene cluster. Although we can not directly deduce the absolute individual copy number, we noticed that in 11 tested individual genomes the ratios range from 1 to 4 in steps of 0.5, suggesting to represent 2 to 8 DEF gene cluster copies per diploid genome.

Our results evidence that - as commonly postulated - the beta defensin genes at 8p23.1 are collectively duplicated in one cluster, although individual clusters may exhibit genetic differences such as MSVs [6,10].
Further work is in progress to establish a reliable estimation of individual DEF cluster copies by MLPA.

[1] Linzmeier, R. et al. (2005) Genomics 86, 423-430
[2] Deutsch, S. et al. (2004) J.Med.Genet. 41, 908-915
[3] Taudien, S. et al., (2006) poster at the HGV meeting HongKong
[4] Armour, J.A.L. et al. (2007) Nucleic Acids Res.35(3):e19. Epub 2006 Dec 14.
[5] Hollox, E. et al. (2003) Am.J.Hum.Genet. 73, 591-600
[6] Taudien, S. et al., (2004) BMC Genomics 5:92
[7] Chen, Q. et al. (2006) J.Immunol.Meth. 308, 231-240
[8] Fellermann, K. et al. (2006) Am.J.Hum.Genet. 79, 439
[9] Schouten, J.P. et al. (2002) Nucleic Acids Res.30(12), e57
[10] Groth et al. (2006) Anal.Biochem.356(2), 194-201

Tenesa, Albert

Genome-Wide Association Mapping Of BMI

TENESA A(1,2), Farrington SM(1,2), Hayward C(2), Knott SA(3), Rudan I(4,5), Wright A(2), Hastie N(2), Dunlop MG(1,2), Campbell H(1,5)

(1) Colon Cancer Genetics Group, University of Edinburgh.
(2) MRC Human Genetics Unit, Edinburgh
(3) Institute of Evolutionary Biology, University of Edinburgh
(4) Faculty of Medicine, Zagred, Croatia
(5) Public Health Sciences, University of Edinburgh

Body mass index (BMI) is a measure of body fat based on height and weight that applies to both adult men and women. It is calculated as weight/height2 and usually measured in kg/m2. BMI is used as a medical diagnostic tool for human eating disorders such as obesity or anorexia. Ideal BMI ranges from 18.5 to 25. Individuals with a BMI less than 17.5 are considered anorexic and individuals with a BMI greater than 40 are considered morbidly obese.

Being overweight (BMI > 25) increases the risk of developing heart disease, type II diabetes and a number of different cancers. For example, a BMI greater than 30 is associated with approximately a 2-fold increased risk of developing colorectal cancer.

BMI is a complex trait that is determined by genetic and environmental factors. Its heritability is ~0.5 for different human populations.

A genome-wide association study for self-reported BMI was performed using Illumina HumanHap500 on 494 unrelated colon cancer cases (aged d 56) and 774 unrelated controls from the general population of Scotland. As the replication set, we used a sample of 483 unrelated Croatians with measures of BMI and typed for Illumina HumanHap300.

Least squares were used to test for marker-trait association. The fit of a model containing gender and age was compared to that of a model containing gender, age and SNP genotype assuming an additive model.

SNPs that replicated at the 5% nominal level across all three datasets -colon cancer Scottish patients, Scottish controls and Croatians- will be typed on additional samples from the general population of Scotland.

Tsai, Shih-Feng

Genomic Instability In Adenocarcinoma Of Lung

SHIH-FENG TSAI; Ya-Hui Chang; Yung-Fung Lin, Shiu-Feng Huang
National Health Research Institutes, Zhunan, Miaoli 350, Taiwan

Adenocarcinoma of lung is a deadly disease, which is a leading cause of cancer mortality. We previously reported that EGFR mutation rate is more than 50% in Taiwanese patients affected with lung adenocarcinoma, and that EGFR mutation is associated with clinical response to gefitinib (Iressa) tyrosine kinase inhibitor treatment. To investigate the genomic instability in lung adenocarcinoma, we have taken clinical samples with or without EGFR mutation and conducted genotyping with STR and SNP markers, comparative genomic hybridization on DNA chips, and gene expression profiling by microrarray. Our data indicated that there is high proportion of 20-repeat EGFR intron 1 sAVH3 allele in Asians, also there is no difference between lung cancer and control in the intron 1 marker allelic distribution. Moreover, there is random loss of short or long allele in the tumor samples, even though the short allele has previously been reported to be linked to a higher promoter activity. EGFR mutation in lung cancer is associated with EGFR allelic imbalance but, in EGFR mutation (+) cases, genomic instability (as revealed by allelic imbalance) is not limited to the EGFR locus. We proposed that genomic instability might be a critical pathogenic event in lung adenocarcinoma with EGFR mutation.

Tuefferd, Marianne

Analyzing 500k SNP Arrays For Copy Number Variations In Partially Degraded Samples

TUEFFERD MARIANNE*(2); De Bondt, An*(1); Van Den Wyngaert, Ilse(1); Talloen, Willem(1); Raghavan, Nandini(3); Amaratunga, Dhammika(3); Broët, Philippe(2); Camilleri-Broët, Sophie(2); Göhlmann, Hinrich(1) [* These authors contributed equally to this work]
(1) Functional genomics, Johnson & Johnson Pharmaceutical Research & Development. A Division of Janssen Pharmaceutica, B-2340, Beerse, Belgium ; (2) JE2492, Faculte de Medecine Paris-Sud, France ; (3) Non-Clinical Biostatistics, Johnson & Johnson Pharmaceutical Research & Development. Raritan, NJ 08869, USA.

SNP microarrays are becoming a reference technology for genome-wide analysis allowing in one experiment detection of SNP status, loss of heterozygosity, and copy number variation (CNV). To gain insight into the CNV on disease susceptibility main limitation is population selection. For most large clinical studies biological samples are retrospectively collected as formalin-fixed and paraffin-embedded (FFPE) material. Genomic imbalance analyses are difficult to perform on this material as extracted DNA is partially degraded. Adapted protocols have been proposed for FFPE specific studies improving hybridization quality, and opening access to large cohorts and genomic information.

Based on four lung tumors from which fresh frozen and corresponding FFPE material was available, extracted DNA was hybridized to 500K SNParrays, and we constructed an analysis workflow for CNV identification that could be applied to FFPE samples. To normalize and summarize the raw data into one unique signal per SNP, performances of the dChip standalone software and the Oligo package (from Bioconductor) were compared and some performance-enhancing modifications suggested. Different graphs emphasizing DNA integrity are suggested. After standardizing and smoothing signal intensities, their allocation to a genomic imbalance state (amplified, deleted or non-modified) is determined using spatially correlated mixture CGHmix model. By this approach, each SNP intensity is associated to a probability to belong to amplified and deleted state. Fixing a threshold for each sample allows defining a discrete allocation variable. A conservative 5% False Discovery Rate was determined, taking into account differences between samples in signal variability.

To validate our SNParray approach, fresh frozen extracted DNA was hybridized to BACarrays. Using CGHmix, the two different techniques gave comparable regions of CNV for the four fresh frozen tumor samples, confirming the use of SNParrays for copy number analyses. In order to evaluate ability to extract specific signal from degraded samples FFPE SNParray results were compared with fresh frozen ones. Larger overall variance was observed for partially degraded FFPE distributions. One FFPE sample showed non specific hybridization characteristics and was considered as highly degraded. Within the non degraded fresh frozen material 19 to 55% of genomic regions were detected as modified, while 11 to 25% of the SNPs were detected as modified within FFPE material. Around 80% of detected modifications in FFPE were identified in corresponding fresh frozen material for two samples showing large genomic modifications. For a less contrasted sample, this was reduced to 40%. Considering only probes hybridizing short DNA fragments (<700bp) did not improve concordance between fresh frozen and FFPE samples. Allocation results for FFPE material were validated by FISH technology for EGFR and c-MYC genes. These results confirm that relevant biological information could be extracted from partially degraded DNA hybridized on SNParray and that analysis procedure stringency should be adjusted depending on DNA quality and overall genomic imbalances observed in tumor.

Varzari, Alexander

Alu Polymorphisms And Y-Chromosome Variation In The Dniester-Carpathian Region

ALEXANDER VARZARI(1,2,3); Vladimir Kharkov(2); Wolfgang Stephan(1); Florina Raicu(4); Valentin Dergachev(5); Valerey Puzyrev(2); Vadim Stepanov(2); Elisabeth Weiss(1)
(1) Biocenter, Ludwig-Maximilians University Munich, Germany; (2) Research Institute of Medical Genetics, Russian Academy of Medical Sciences, Tomsk, Russia; (3) National Center of Reproductive Health and Medical Genetics, Kishinev, Moldova; (4) Anthropological research Center Fr. Rainer, Bucharest, Romania; (5) Institute of Archaeology and Ethnography, Academy of Sciences of Moldova, Kishinev, Moldova

The area between the Dniester and the eastern Carpathian mountain range represents an important geographical link between Eastern Europe and the Balkans. To gain insights into the genetics of the Dniester-Carpathian region, we characterized 12 polymorphic Alu markers and Y chromosome haplotypes constructed on the basis of 32 binary and 7 STR markers in six autochthonous population groups: two Moldavian, one Romanian, one Ukrainian and two Gagauz populations. A total of 513 unrelated healthy people were studied for autosomal Alu markers and 322 male individuals of them were analyzed for Y chromosome variation. The data were compared with published data from populations of western Eurasia. The analysis of autosomal Alu polymorphisms showed a high degree of homogeneity among southeastern European populations (Gst=0.0161) and, in particular, those of the Dniester-Carpathian region (Gst=0.0038). Despite this low level of differentiation, tree reconstruction and principle component analyses allowed a distinction between Balkan-Carpathian (Macedonians, Romanians, Moldavians, Ukrainians and Gagauzes) and eastern Mediterranean (Turks, Greeks and Albanians) population groups. These findings are consistent with those from classical and DNA markers (Cavalli-Sforza et al. 1994; Malaspina et al. 2000) and are also compatible with archaeological and paleoanthropological data (Velikanova 1975; Renfrew 1987). The analysis of Y-chromosome polymorphism revealed a high level of variability within the Dniester-Carpathian male gene pool and a high level of population differentiation in this geographically relatively small region (Fst=0.0183 for binary haplogroups; Rst=0.0205 for STR haplotypes). The major haplogroups (R1a1, I1b, R1b3, G, E3b1 and J2, totaling 83.6%) are shared with neighboring southeastern and eastern European populations. Both gene frequency and admixture analyses showed that Ukrainians and southeastern Moldavians harbor a conspicuous proportion of Y-chromosomes of eastern European origin; Romanians and northern Moldavians share a large fraction of Y-chromosomes characteristic of the western Balkans; and the Gagauzes have substantial proportions of lineages of Near Eastern origin, comparable to those in Balkan populations. An intriguing finding of this study was that the transcontinental genetic barrier, separating Eastern Europe from the rest of the continent, passes through the Dniester-Carpathian territory. None of the genetic marker systems used in this study revealed a correspondence between genetic and linguistic patterns in the Dniester-Carpathian region or in southeastern Europe, which suggests either that the ethnic differentiation in these regions was very recent or that the linguistic and other social barriers were not strong enough to prevent gene flow between populations. In particular, Gagauzes, a Turkic speaking population, show no closer affinities to other Turkic peoples than to their geographical neighbors.

Vibhuti, Arpana

ACE, NOS3, GSTP1 And mEPHX Variants And Oxidative Stress Markers Associate With COPD

ARPANA VIBHUTI; Ehtesham Arif; Desh Deepak*; Bhawani Singh*; M A Qadar Pasha
Functional Genomics Unit, Institute of Genomics and Integrative Biology, Delhi, India; *Respiratory Unit, Dr Ram Manohar Lohia Hospital, New Delhi, India

Chronic obstructive pulmonary disease (COPD) is characterized by irreversible airflow limitation, abnormal permanent distal air-space enlargement and emphysema in the lungs. Oxidative stress plays a major role in the pathogenesis of COPD. Cigarette smoking, although, is the most important risk factor, only 10% of the chronic heavy smokers develop symptomatic COPD suggesting that there must be some genetic predisposing risk factors contributing to the susceptibility to this disease. The genetic susceptibility might depend on variations in pulmonary hypertension associated genes, and detoxifying genes that activate and detoxify cigarette smoke products, which otherwise generate oxidative stress.

In a case-control study we investigated the angiotensin I converting enzyme (peptidyl-dipeptidase A) 1 (ACE) I/D (GenBank accession no X62855), G894T (rs1799983) and CA-repeat (Ensembl Gene ID-ENSG00000164867) of nitric oxide synthase 3 (endothelial cell) (NOS3), I105V (rs1695), A114V (rs1138272) of Glutathione-S-transferase P 1 (GSTP1) and Y113H (rs1051740), H139R (rs2234922) of microsomal epoxide hydrolase (mEPHX) along with oxidative stress markers such as malondialdehyde (MDA), reduced glutathione (GSH) and glutathione peroxidase (GPx), ACE and NO levels. We examined the association of ACE, NOS3, GSTP1 and mEPHX variants individually or in combination with disease and their contribution to ACE, NO, MDA, GSH and GPx.

The distribution of I/D of ACE and G894T and CA repeat of NOS3 did not differ significantly between the two groups. However, the patients were over-represented by the alleles 105V, 114V of GSTP1 and 113H, 139H of mEPHX (2=12.24, p=0.002; 2=13.07, p=0.001; 2=10.68, p=0.004 and 2=7.33, p=0.025, respectively) and the haplotypes of same alleles i.e. 105V-114V and 113H-139H (?2=14.58, p<0.001 and ?2=23.14, p<0.001), whereas, wild-type haplotype 105I-114A of GSTP1 and 113Y-139H of mEPHX were significantly over-represented in the controls (?2=16.06, p<0.0001 and ?2=7.548, p<0.0001). Moreover, there was marked over-representation of combination of genotypes, I105I+A114A of GSTP1 (53% vs. 36%) in controls; whereas, the combinations with 105V/114V alleles (64% vs. 47%) of GSTP1 (OR=1.99; 95% CI=1.28-3.09; p=0.002) and the homozygotes H113H+H139H (27% vs.10%) of mEPHX (OR=3.26; 95% CI=1.73-6.15; p=0.0001) in patients. Furthermore, the combination of II+GG genotypes of ACE and NOS3 was significantly greater (30% vs. 15%, OR=2.43, 95% CI=1.21-4.87; P=0.01) in the controls as compared to the patients. The patients had significantly elevated ACE (p=0.05), MDA levels (p<0.001) and decreased GSH level (p<0.001) and GPx activity (p=0.035), respectively. Of note, Subjects having the combination of II+GG genotypes had lowest ACE activity and highest NO level. The changeover from II to ID and then to DD resulted into increased ACE activity, whereas the conversion from GG to GT and then to TT decreased NO levels in both groups. Furthermore, the genotypes, I105V/V105V, A114V/V114V of GSTP1 and Y113H/H113H of mEPHX associated with increased MDA level (p=0.04, p=0.03 and p=0.003) and decreased GSH level (p=0.019, p=0.007 and p=0.0006), respectively, in the patients; so was the correlation of these biomarkers with the combinations of the genotypes.

In conclusion, genetic predisposition to lower circulating ACE and higher NO levels by virtue of the interaction between I and G alleles, respectively, appear to cause less vasoconstriction and increased vasodilatation that may be advantageous to the patients. Furthermore, slow enzyme activity associated alleles 105V, 114V of GSTP1 and 113H, 139H of mEPHX and haplotypes of the same alleles were over-represented in the patients; the same alleles associated with airflow obstruction, imbalanced oxidative stress such as elevated MDA level and lower GSH level, GPx activity. Moreover, the genotype combinations and their correlations with respective biomarkers further strengthened our findings. It is evident from these findings that gene polymorphisms affecting the function of proteins causes imbalance of oxidative stress thereby contributing to the pathogenesis.

Wakil, Salma

Localization Of Familial Juvenile Idiopathic Arthritis

SALMA M WAKIL(2), Sulaiman Al Mayouf(1), Rana Al-Amr(2), Osama Alsmadi, Dorota Monies(2) and Brian F Meyer(2).
(1) Department of Pediatrics, King Faisal Specialist Hospital and Research Centre, Riyadh, Saudi Arabia; (2) Arabian Diagnostic Laboratory, Dept. of Genetics, Research Centre, King Faisal Specialist Hospital and Research Centre, Riyadh, Saudi Arabia.

Juvenile idiopathic arthritis represents the most common chronic rheumatic inflammatory conditions of childhood. Children with familial juvenile idiopathic arthritis (JIA) are clinically evaluated. All included patients fulfilled the accepted definition of JIA using the ILAR criteria. We defined familial JIA patients as belonging to a family with more than one sibling diagnosed with JIA. Familial autoinflammatory diseases including certain subgroups of JIA (i.e. spondyloarthropathies), familial Mediterranean fever or known syndromes associated with articular manifestations were excluded. All patients were assessed with respect to: demographic information, age of onset of JIA, disease activity, disease damage, laboratory variables and functional class. Eleven affected siblings (9 female/ 3 male) with JIA belonged to 4 apparently unrelated families. And all the patients were from the same geographical area. The mean age at onset was 2.4 years, and mean age at diagnosis was 3.5 years. And the mean duration of follow up was 6.4 years. All patients presented with multiple joint involvement at diagnosis. One third of the patients had a poly-articular onset subtype, and the remainder had a systemic onset subtype presenting with typical quotidian fever and the classic associated skin rash. All patients in this group had elevated inflammatory markers. Rheumatoid factor was positive in 38% and the antinuclear antibody was positive in 43% of the patients. Radiological evaluation revealed significant osteopenia, joint space narrowing and erosions.

A whole genome scan was performed using the Affymetrix GeneChip Mapping10K 2.0 Array for linkage analysis for the four consanguineous families. Genome-wide parametric linkage analysis of the four families using an autosomal recessive model of inheritance localized the disease to an ~4cM region of HSA 13q with a combined multipoint LOD score of 9.87. Fine mapping and a positional candidate approach are being used to identify the underlying gene.

Familial JIA has similar clinical features to the more common sporadic JIA. Localization of familial JIA to HSA 13q and subsequently, cloning of the underlying gene may provide further insight into the immune cascade and tissue destruction of this disorder. Thereby, opportunities for earlier diagnosis and better management of the disease including the detection of carrier status may provide the basis for reduced morbidity and disease prevention.

References:
1. Tom H Lindner, K Hoffmann. (2005) easy Linkage: a PERL script for easy and automated two-/multi-point linkage. Bioinformatics Feb 1;21 (3):405-7

Weiss, Lauren

Genome-Wide Analysis Of Copy Number Variation In Psychiatric Disease

LAUREN A. WEISS(1,2), Manuel Ferreira(1,2), Joshua Korn(1,2), Finny Kuruvilla(1,2), Steven McCarroll(1,2), Stephen Faraone(3), Hugh Gurling(4), Nan Laird(5), Matt McQueen(6), Andrew McQuillin(4), Vishwajit Nimgaonkar(7), Jordan Smoller(1), Jacob Lawrence(4), Ana Pereira(4), Nick Bass(4), Michelle Robinson(4), Mark Daly(1,2), Shaun Purcell(1,2), Pamela Sklar(1,2)
(1) Center for Human Genetic Research, Massachusetts General Hospital, Boston MA; (2) Broad Institute of Harvard and MIT, Cambridge MA; (3) Psychiatry and Neuroscience & Physiology, SUNY Upstate Medical University, Syracuse, NY; (4) Departmental of Mental Health Sciences, University College London, Windeyer Institute, London, UK; (5) Department of Biostatistics, Harvard School of Public Health, Boston MA; (6) Institute for Behavioral Genetics, Boulder CO; (7) Psychiatry and Human Genetics, University of Pittsburgh School of Medicine & Graduate School of Public Health, Pittsburgh PA

It is increasingly being recognized that copy number polymorphism and *de novo* mutation could have a major contribution to common, complex genetic disease, including psychiatric disease like schizophrenia, autism, and bipolar disorder. Well-powered association studies are being designed to collect genome-wide SNP data for thousands of samples in order to dissect the genetic architecture of these disorders. Although intensity data from SNP probes has been used in small datasets to assess copy number across the genome, currently available methods are difficult to scale to studies with thousands of samples, therefore we have developed a novel method to estimate copy number from raw intensity SNP probe data generated on the Affymetrix platform. Our aims were to design a method to test for association between common copy number polymorphism and disease, as well as detect *de novo* or rare copy number changes at an individual level in large genome-wide SNP datasets.

We utilize the intensity normalization and probe summarization implemented in BRLMM, which gives raw intensity data for each allele for each SNP. We further normalize this data in a plate-wise manner for each SNP across the sample set, eliminating probe-specific differences that bias locus intensity by genotype class. After our normalization process, we have a quantitative estimate of allele-specific copy number at each SNP, to which we apply a correction factor on an individual basis to account for genome-wide differences in intensity due to unmeasured sources of systematic bias (e.g. DNA quality). We then categorize these quantitative calls into integer copy number calls for each allele, which we can sum to obtain a SNP-specific copy number estimate. We consider the information provided by neighboring SNPs (which is technically independent, but potentially biologically correlated for copy number) to identify regions of copy number variation on a population level. Additionally, we can assess copy number estimates per individual across a chromosome to identify *de novo* or rare events. In preliminary application of this novel method to chromosome 22 in 3800 individuals, including controls from the NIMH repository and cases with Bipolar Disorder, we identified several common copy number polymorphisms in regions where copy number variation had previously been reported and detected multiple rare duplication events. Validation of these results is ongoing. We will apply this method to genome-wide data for Bipolar Disorder, Autism and Schizophrenia and analyze regions of copy number variation for clinical significance.

White, Phoebe

Copy Number Variation Analysis Using Quantitative Taqman® Copy Number Assays

Kelly Li(1), Adam J. Broomer(1), Yu Wang(1), Chunlin Xiao(1), Catalin Barbacioru(1), Iris R. Casuga(1), Andrew J Sharp(2), Evan E. Eichler(2), Meri L. Bozzini(1), PHOEBE A. WHITE(1) and Caifu Chen(1)

(1) SDS/Array R&D, Molecular Cell Biology Division, Applied Biosystems, 850 Lincoln Centre Dr., Foster City, CA 94404, USA
(2) Department of Genomic Sciences and Howard Hughes Medical Institute, University of Washington School of Medicine, 1705 NE Pacific St., Seattle, WA 98195, USA

Recent whole-genome studies have identified 1447 CNV regions (CNVRs) that cover about 12% of the human genome. Some of CNVR may contain disease loci/genes, whose copy number changes could impact gene activity and disease susceptibility. Copy number changes are also detected in microdeletion/microduplication syndromes, which are associated with genomic disorders. Although array-based technologies are powerful for large-scale CNV discoveries and microdeletion/microduplication syndrome screening, more quantitative technologies with higher sample throughput are required to validate newly identified CNVs and to detect deletions/duplications for a large sample size in candidate regions/genes. To meet these challenges and demands, Applied Biosystems has developed TaqMan® based real-time quantitative copy number assays. Here, we report the development of the TaqMan® copy number assay design pipeline and validation of TaqMan® copy number assays. We used this proprietary pipeline to design assays targeting the chromosomal regions associated with genomic disorders and CNV-associated OMIM genes. The assays were tested with DNA sets for validation, HAPMAP DNA collection as well as samples with known deletions/duplications. The TaqMan® copy number assay is a duplex reaction with a FAM™-assay targeting the gene of interest and a VIC®-assay targeting the reference gene (two copies per diploid genome) in the same well. The copy number is determined by relative quantification using a reference sample known to have two copies of the gene of interest. Our validation data demonstrate a high success rate of assay design and excellent assay performance. TaqMan® copy number assays are quantitative and robust, with high reproducibility, specificity, and sample throughput.

Wilhelmsen, Kirk

Convergent Haplotype Association Tagging (CHATing): Detection of LRRK2 In The Ashkenazi With Parkinson's Disease Which Can Not Be Readily Detected By Standard Case-Control GWA Methods.

WILHELMSEN, KIRK; Webb Amy, Chasse S
University of North Carolina-Chapel Hill

GWA analysis using standard contingency analysis is most powerful for detecting loci when the linked marker allele has a similar frequency and is in disequilibrium with the disease causing allele. Because the genotyping for GWA is usually biased towards polymorphisms with minor allele frequencies greater than 1% and it is unlikely that a rare variant will happen to be typed that is in strong LD with a rare disease allele, standard contingency analysis is unlikely to detect rare variants that cause disease. Power is further decreased when there is allelic heterogeneity. Gene based haplotype methods may perform better but long range haplotype sharing may provide stronger evidence that subsets of affected individuals have inherited a disease allele from a common ancestor. CHATing is a computational strategy for screening the genome for subsets of individuals that share a common ancestral haplotype. The algorithm deduces the shared haplotype, called a CHAT. The minimal region shared by all individuals with part of the CHAT provides localizing information for the disease causing allele.

To test this approach, a dataset was constructed with a known disease associated allele which was unlikely to be detected by simple marker by marker or gene by gene association analysis. A rare variant of the LRRK2 gene is strongly associated with Parkinson's disease (PD) in the Ashkenazi and appears to be responsible for disease in a small fraction of patients with PD. GWA data (with 500K Affymetrix chips) was collected for 96 each of LRRK2 negative patients with PD and controls. An additional 20 subjects with PD with the same LRRK2 mutation were also genotyped. Each of the participants had 4 grandparents that are self described as Ashkenazi. When conventional GWA analysis was performed there was not an obvious signal near LRRK2 when genotype frequencies of PD cases (+/- LLRK2 mutation) were together compared with controls. CHATs were detected at LRRK2 (the strongest signal in the genome) as well as two other loci. This suggests that CHAT can be used to detect relatively common rare variants with shared haplotypes using standard GWA data.

Yang, Hsin-Chou

Detection Of Allelic Imbalance Using Accurate Allele Frequency Estimation

HSIN-CHOU YANG(1), Ling-Hui Li(2), Mei-Chu Huang(1), Alice L. T. Yu(4), Mitchell B. Diccianni(5), Chien-Hsin Lin(3), Jer-Yuan Wu(2), Yuan-Tsong Chen(2), Cathy S. J. Fann(2)
(1) Institute of Statistical Science, Academia Sinica, Taipei, Taiwan; (2) Institute of Biomedical Sciences, Academia Sinica, Taipei, Taiwan; (3) Institute of Genome Sciences, Yang-Ming University, Taipei, Taiwan; (4) Genomics Research Center, Academia Sinica, Taipei, Taiwan; (5) Department of Pediatrics/Hematology Oncology, University of California, San Diego, USA

A recent important application of the array-based SNP genotyping technique is detection of chromosomal abnormalities and allelic imbalance (AI) including loss of heterozygosity (LOH). These genomic aberrations may change gene function/dosage/expression which results in increased disease susceptibility or tumor development. Identification of the abnormalities not only helps to locate disease susceptibility genes, oncogenes, and tumor suppressor genes, but also contributes to decipher the underlying mechanisms of disease/cancer etiology.

To detect chromosomal regions with AI, we developed single-point and multipoint detection methods based on accurate allele frequency (AF) estimation in a single sample by incorporating coefficient of preferential amplification/hybridization. The proposed single-point detection method examines whether the estimated AF of SNPs from an individual was located within one of the three genotype-specific prediction bands constructed from a normal population. SNPs with AF deviated from the standards were designated AI SNPs. We then developed cumulative-sum multipoint methods by incorporating information of neighbouring SNP markers. Positive scores were assigned to AI SNPs and negative scores were assigned to non-AI SNPs based on the results of the single-point detection. Scores for ordered SNP loci were accumulated from the starting SNP on each chromosome for identifying AI regions. A second multipoint statistic focused on detecting regions with significant LOH. In this method, all SNPs were divided into heterozygous and non-heterozygous SNPs. If the estimated AF of a SNP was located within the prediction band of heterozygous calls, the SNP was classified as a heterozygous SNP; otherwise it was classified as a non-heterozygous SNP. Positive scores were assigned to non-heterozygous SNPs and negative scores were assigned to heterozygous SNPs, and then scores for ordered SNP points were accumulated from the starting SNP on each chromosome for identifying LOH regions.

We have incorporated the above methods and normal reference database into user-friendly software named MPDA (http://www.stat.sinica.edu.tw/hsinchou/genetics/pooledDNA/mpda.htm). The proposed method and software have been successfully applied to scan chromosomal aberrations in acute lymphoblastic leukemia. Both AI-induced copy number change and copy-neutral LOH were identified with this newly developed AI detection method. The results indicate that accurate AF estimation is a sensitive way to detect genomic regions with allelic aberrations at individual sample level. This will greatly facilitate the identification of disease genes.

Yeager, Meredith

Common Variation At 8q24 And Prostate Cancer Risk

MEREDITH YEAGER(1,2), Nick Orr(3), Kevin Jacobs(2,4), Robert Welch(1,2), Andrew Crenshaw(1,2), Laura Burdett(1,2), Nick Xiang(1,2), David Hunter(2,5), Robert Hoover(2), Joseph F. Fraumeni, Jr.(2), Gilles Thomas(2), Stephen J. Chanock(2,3)
(1) Advanced Technology Program, SAIC-Frederick, NCI-FCRDC, Frederick, MD; (2) Division of Cancer Epidemiology and Genetics, National Cancer Institute, National Institutes of Health, Department of Health and Human Services, Rockville, MD; (3) Pediatric Oncology Branch, Center for Cancer Research, NCI, NIH, DHHS, Bethesda, MD; (4) Bioinformed Consulting Services, Gaithersburg, MD; (5) Program in Molecular and Genetic Epidemiology, Department of Epidemiology, Harvard School of Public Health, Boston, Massachusetts

Recently, several groups have reported strong associations between common DNA polymorphisms that span a segment of chromosome 8q24 and the risk of prostate cancer. There is evidence that at least three regions of this segment (chromosome 8: 126501167-128998553) are independently associated with risk and are also dependent on the ethnic origin of prostate cancer cases. We have extensively characterized common genetic polymorphisms present in individuals of European origin for two of these regions by re-sequence analysis (chromosome 8: 128470954-128619305) in the Cancer Genetic Markers of Susceptibility project (http://cgems.cancer.gov). In preliminary association studies of more than 4000 cases and 4000 controls drawn from population-based studies, we have identified haplotypes on which disease-contributory mutations most likely exist. These data may be used to more rapidly identify these mutations so that they can be investigated for functional significance. There is growing evidence that these regions are also implicated in other cancer types; these observations underscore the importance of characterizing common genetic variation at 8q24.

Zeng, Changqing

A Six Nucleotide Indel Of CASP8 Promoter Correlates To Cancer Susceptibility

Yang Gao(1), Tong Sun(2), Sufang Ma(1), Wen Tan(2), Dongxin Lin(2), and CHANGQING ZENG(1)
(1) Beijing Genomics Institute, The Chinese Academy of Sciences; Beijing Airport Industrial Zone, B6, Beijing, 101300, China; (2) Cancer Institute and Hospital, Chinese Academy of Medical Sciences and Peking Union Medical College; 17 Panjiayuan Nanli, Beijing, 100021, China

Caspases have been shown to be related to cancers. In this study we attempted to investigate potential impacts of the polymorphisms within the gene cluster of CASP8-CASP10-CFLAR on the risk of developing lung cancer. Using HapMap Phase I data, tag SNPs were selected in this 200 kb region at chromosome 2q33. In total, 18 SNPs were genotyped in 923 case and 888 control individuals. A significant association with lung cancer
was observed in a 25-kb region containing CASP8 promoter and 5.UTR (rs6747918, odds ratio, 2.16; $P = 1.93x10^{-13}$). We then resequenced this region and identified 3 common variants (heterozygosity >0.05) as CASP8-G 856C (rs3729647), CASP8-652 AGTAAG ins/del (rs3834129), and CASP8-G614A (rs6747918). Among them CASP8-652 6N ins creates an additional binding site for transcription factor Sp1, suggesting possible correlation of CASPASE-8 activity to this indel polymorphism, as well as the association of CASP8 expression with the cancer susceptibility. A series of expression and enzyme activity assays were then conducted to verify these possibilities. T lymphocytes with the CASP8-652 6N ins were shown to have increased levels of both CASPASE-8 activity and activation-induced cell death upon stimulated with cancer cell antigens. Further sequencing analysis in enlarged samples demonstrated this genetic variant is associated with susceptibility to multiple cancer types including lung, esophageal, gastric, colorectal, cervical, and breast caners. These results support the hypothesis that genetic variant influencing individual's immune status may confer cancer susceptibility.

Zhao, Chunyan

Functional Characterization Of A Novel Variant Of Estrogen Receptor Beta Identified In Screening Of DNA Derived From African Americans

CHUNYAN ZHAO, Jan-Åke Gustafsson and Karin Dahlman-Wright
Department of Biosciences and Nutrition, Novum, Karolinska Institutet, S-141 57 Huddinge, Sweden

Estrogen signaling in the cells is mediated by two forms of estrogen receptors (ERs), ERalpha and the more recently discovered ERbeta (1). These receptors belong to the nuclear receptor hormone superfamily and are ligand-inducible transcription factors. Polymorphisms in ER genes have been reported to be associated with some endocrine related disorders. For example, polymorphic alterations of ERalpha have been associated with breast cancer, endometrial cancer, menstrual disorder, Alzheimer's disease, osteoporosis and coronary artery disease. Polymorphisms in ERbeta have been correlated to ovulatory dysfunctions, hypertension, bone mineral density, bulimic disease and androgen levels. However, no information on single nucleotide polymorphisms in the ER genes is available for the African American population. In this study, we systematically screened the coding and flanking intron regions of the ERbeta gene in 49 healthy African American individuals. Ten sequence variants were identified. Among them, two variants (1082G/A and 1730G/A) have been reported in Caucasians and an African population, and four variants (105A/G, 143C/T, 566A/T and 1100T/C) have previously been reported in an African population. Additionally, we found four novel polymorphisms in exon sequences (728C/T, 963T/C, 1073C/T and 1109G/A) in this African American population. Interestingly, one of these novel variants (963T/C) resulted in amino acid change from phenylalanine to leucine at position 289, referred to as ERbeta-F289L. This receptor variant was further characterized in vitro for transcriptional activity and ligand-binding. These studies revealed that ERbeta-F289L had reduced estrogen binding affinity and impaired response to 17beta-estradiol induced transactivation compared to the wild type ERbeta. This novel variant might confer genetic susceptibility to certain endocrine related diseases in African Americans. Further association studies are required to determine whether this polymorphism is involved in the increased incidence of prostate and breast cancers in African Americans.

(1) Kuiper GG, Enmark E, Pelto-Huikko M, Nilsson S, Gustafsson J-Å. Cloning of a novel receptor expressed in rat prostate and ovary. Proc Natl Acad Sci U S A 1996; 93:5925-5930.

List of Presenting Participants

NAME	FORMAT	PAGE (poster No)
Irina Abnizova	Poster	39
Tomasz Adamusiak	Poster	40
Lidia Agueda	Poster	41
Joo Wook Ahn	Poster	42
Peter Aka	Poster	43
Noora Alakulppi	Poster	44
Thomas Albert	Poster	45
Anders Albrechtsen	Poster	46
Rosa Aledo	Poster	47
Signe Altmäe	Poster	48
Lluis Armengol	Oral	1
John Armour	Oral	2
Antonio Barbadilla	Poster	49
John Barber	Poster	50
Mònica Bayés	Poster	51
Olivia Belbin	Poster	52
Jennifer Below	Poster	53
Jaume Bertranpetit	Oral	3
Anshu Bhardwaj	Poster	54
Sanjeev Bhaskar	Oral	4
Ewan Birney	Oral	5
Janusz Blasiak	Poster	55
Elena Bosch	Poster	56
Nina Bosch	Poster	57
Maria Brión	Poster	58
Anthony J Brookes	Oral	6
Jasper Brugts	Poster	59
Anna Brunet	Poster	60
Tiffany Brunson	Poster	61
Mariona Bustamante	Poster	62
Anton Buzdin	Poster	63
Patrick Cahan	Poster	64
Francesc Calafell	Poster	65
Anne Cambon-Thomsen	Oral	7
Danielle Carpenter	Poster	66
Angel Carracedo	Oral	8
Christopher Carter	Poster	67
Ferran Casals	Poster	68
Isfahan Chambers	Poster	69
TingFung Chan	Poster	70
Stephen Chanock	Oral	9
Fadi Charchar	Poster	71
Carole Charlier	Oral	10
Vivian Cheung	Oral	11
George Church	Oral	12
Roger Colobran	Poster	72
Donald F Conrad	Oral	13
Nancy Cox	Oral	14
Pio d'Adamo	Poster	73
Mauro D'Amato	Poster	74

NAME	FORMAT	PAGE (poster No)
Johannes Dapprich	Poster	75
Ariel Darvasi	Poster	76
Subhajyoti De	Poster	77
Francisco De La Vega	Poster	78
Paul Dear	Poster	79
Emmanouil Dermitzakis	Oral	15
Andrew Devereau	Oral	16
Igor Deyneko	Poster	80
Hernan Dopazo	Poster	81
Peg Eis	Poster	82
Xavier Estivill	Oral	17
Israel Fernandez	Poster	83
Olga Fernando	Poster	84
Lars Feuk	Oral	18
Elodie Gazave	Poster	85
Ilse Gijselinck	Poster	86
Juan Gonzalez	Poster	87
Esteban González-Burchard	Oral	19
Anna González-Neira	Poster	88
Jenny Göransson	Poster	89
Carolin Gräbsch	Poster	90
Monica Gratacos	Poster	91
Hákon Guðbjartsson	Poster	92
Jeff Gulcher	Oral	20
Ivo Gut	Oral	21
Hakon Hakonarson	Poster	93
Ira Hall	Poster	94
Pille Hallast	Poster	95
Thomas Hansen	Poster	96
Charlotte N. Henrichsen	Poster	97
Stephanie Hesselson	Poster	98
Kirk Hogan	Poster	99
Ed Hollox	Poster	100
Matthew Hurles	Oral	22
Michael Inouye	Poster	101
Iuliana Ionita	Oral	23
Michael James	Poster	102
Aslaug Jonasdottir	Poster	103
Maria Kapasa	Poster	104
Masaru Katoh	Poster	105
John Kauwe	Poster	106
Namik Kaya	Poster	107
Jong-Won Kim	Poster	108
Toshio Kojima	Poster	109
Eloise Kok	Poster	110
Jussi Kollin	Poster	111
Jan Korbel	Poster	112
Patricia Kramer	Poster	113
Gregory Kryukov	Poster	114
Pui-Yan Kwok	Oral	24

NAME	FORMAT	PAGE (poster no)
Maris Laan	Poster	115
Hafid Laayouni	Poster	116
Stephen Laderman	Poster	117
Thomas LaFramboise	Poster	118
Christoph Lange	Poster	119
Charles Lee	Poster	120
Heikki Lehvaslaiho	Oral	25
Samuel Levy	Oral	26
Ling-Hui Li	Poster	121
Yung-Feng Lin	Poster	122
Ruy Lopez-Ridaura	Poster	123
Belén Lorente	Poster	124
Per Lundmark	Poster	125
Reedik Mägi	Poster	126
Vsevolod Makeev	Poster	127
Urko Martinez	Poster	128
Cheryl Maslen	Poster	129
Frank McCaughan	Poster	130
Josep-Maria Mercader	Poster	131
Montserrat Milà	Poster	132
Dimitri Monos	Poster	133
María-Cristina Morán Moguel	Poster	134
Jonathan Mudge	Poster	135
Margarita Muiños-Gimeno	Poster	136
Robin Munro	Poster	137
Masaaki Muramatsu	Poster	138
Muhammad Naeem	Poster	139
Kimitoshi Naito	Poster	140
Arcadi Navarro	Poster	141
Debbie Nickerson	Oral	27
Tiit Nikopensius	Poster	142
Silje Nordgard	Poster	143
Karen Nuytemans	Poster	144
Avi Orr-Urtreger	Poster	145
James Ostell	Oral	28
Dimitri Pappaioannou	Poster	146
Bruno Paquin	Poster	147
Laurence Parnell	Poster	148
Andrzej Pawlak	Poster	149
Luis Perez-Jurado	Poster	150
George Perry	Oral	29
Chris Ponting	Oral	30
Boode Prathap Naidu	Poster	151
Tarmo Puurand	Poster	152
Tasleem Raza	Poster	153
Marta Ribasés-Haro	Poster	154
Susan Ring	Poster	155
Daniel Rios	Poster	156
Mercedes Robledo	Poster	157
Carsten Rosenow	Poster	158

NAME	FORMAT	PAGE (poster No)
Allen Roses	Oral	31
Janna Saarela	Poster	159
Maria Sasvari-Szekely	Poster	160
Ester Saus	Poster	161
Stephen Scherer	Oral	32
Jean-Jacques Schott	Poster	162
Rao Sethumadhavan	Poster	163
Snaevar Sigurdsson	Poster	164
Martin Sikora	Poster	165
Andrew Skol	Poster	166
Beatriz Sobrino	Poster	167
Kyuyoung Song	Poster	168
Richard Spielman	Poster	169
Marija Stankovic	Poster	170
Lincoln Stein	Oral	33
Vadim Stepanov	Poster	171
Marc Sultan	Poster	172
Shamil Sunyaev	Oral	34
Paul Tam	Poster	173
Stefan Taudien	Poster	174
Albert Tenesa	Poster	175
Gilles Thomas	Oral	35
Barbara Trask	Oral	36
Shih-Feng Tsai	Poster	176
Marianne Tuefferd	Poster	177
Alexander Varzari	Poster	178
Joris Veltman	Oral	37
Arpana Vibhuti	Poster	179
Salma Wakil	Poster	180
Lauren Weiss	Poster	181
Phoebe White	Poster	182
Kirk Wilhelmsen	Poster	183
Hsin-Chou Yang	Poster	184
Meredith Yeager	Poster	185
Yum Lina Yip	Oral	38
Changqing Zeng	Poster	186
Chunyan Zhao	Poster	187

Participant Contact Details

Irina Abnizova
Institute of Public health
MRC Biostatistics Unit
Robinson Way
Cambridge, CB2 2SR
UK
Phone: +44 1223 330385
Fax: +44 1223 330366
Email: irina.abnizova@mrc-bsu.cam.ac.uk

Tomasz Adamusiak
Medical University of Lodz
Department of Immunopathology
Faculty of Postgraduate Medical Education
251, Pomorska str., bldg C5 Rm 46
92-213 Lodz
Poland
Phone: +48 604 235000
Fax: +48 42 6782292
Email: tomasz.adamusiak@gmail.com

Lidia Agueda
University of Barcelona
Avda. Diagonal 645, ed. annex, 3ª
Spain
Phone: +34 93 4021794
Fax: +34 93 4034420
Email: lagueda@ub.edu

Joo Wook Ahn
Guy's and St Thomas' NHS Foundation Trust
Guy's Hospital
London
UK
Phone: +44 20 71881698
Fax: +44 20 71881697
Email: joowook.ahn@gstt.nhs.uk

Peter Aka
Medical Research Council Laboratories
P.O. Box 273
Banjul
Gambia
Phone: +220 7926830
Email: paka@mrc.gm

Noora Alakulppi
Finnish Red Cross Blood Service
Research and Development
Kivihaantie 7
FIN-00310 Helsinki
Finland
Phone: +358 958011
Fax: +358 895801310
Email: noora.alakulppi@veripalvelu.fi

Thomas Albert
NimbleGen Systems, Inc.
Research and Development
One Science Court
Madison, WI
53711USA
Phone: +1 608 2187613
Fax: +1 608 2187601
Email: talbert@nimblegen.com

Anders Albrechtsen
Copenhagen University
Department of Biostatistics
Øster Farimagsgade 5, Entr. B
P.O.B. 2099
DK-1014 Copenhagen K
Denmark
Phone: +45 26 754449
Fax: +45 35 327907
Email: albrecht@binf.ku.dk

Rosa Aledo
Cardiovascular Research Center ICCC-CSIC
Hospital de la Santa Creu i Sant Pau
Av. Maria Claret, 167
Barcelona 08025
Spain
Phone: +34 93 5565904
Fax: +34 93 5565559
Email: raledo@csic-iccc.org

Signe Altmäe
Molecular and Cell Biology
Department of Biotechnology
Riia 23
51010 Tartu
Estonia
Phone: +372 5088723
Email: signe.altmae@ut.ee

Lluís Armengol
Center for Genomic Regulation
Genes & Disease Program
Plaça Charles Darwin, SN
08003 Barcelona
CATALUNYA
Spain
Phone: +34 93 3160105
Fax: +34 93 3160865
Email: lluis.armengol@crg.es

John Armour
University of Nottingham
Institute of Genetics
Queen's Medical Centre
Nottingham, NG7 2UH
UK
Phone: +44 115 8230308
Email: john.armour@nottingham.ac.uk

Orli G Bahcall
Nature Publishing Group
Nature Genetics
75 Varick Street, 9th Floor
New York, NY 10013
USA
Phone: +1 212 7269311
Email: o.bahcall@natureny.com

Antonio Barbadilla
Universitat Autònoma de Barcelona
Genètica i Microbiologia
Departament Genètica i Microbiologia
Facultat de Biociències, Campus UAB
Bellaterra (Barcelona) 08193
Spain
Phone: +34 93 5812730
Fax: +34 93 5812387
Email: antonio.barbadilla@uab.es

John Barber
Wessex Regional Genetics Laboratory
Salisbury District Hospital
Salisbury, SP2 8BJ
UK
Phone: +44 1722 429080
Fax: +44 1722 338095
Email: john.barber@salisbury.nhs.uk

Mònica Bayés
Centre for Genomic Regulation
Genes and Disease Programme
Dr. Aiguader, 88
Barcelona 08003
Spain
Phone: +34 93 2240951
Fax: +34 93 3160099
Email: monica.bayes@crg.es

Olivia Belbin
University of Nottingham
Clinical Chemistry
A Floor, West Block
Nottingham
UK
Phone: +44 1158230723
Fax: +44 1159709167
Email: olivia.belbin@nottingham.ac.uk

Jennifer Below
University of Chicago
Human Genetics
8244 South Luella Ave
Chicago, IL 60617
USA
Phone: +1 773 7211242
Email: below@uchicago.edu

Jaume Bertranpetit
Universitat Pompeu Fabra
Experimental and Health Sciences
Doctor Aiguader 88
08003 Barcelona
Spain
Phone: +34 93 3160840
Fax: +34 93 3160901
Email: jaume.bertranpetit@upf.edu

Anshu Bhardwaj
Centre for Cellular and Molecular Biology
Bioinformatics
Uppal Road
Hyderabad - 500007
India
Phone: +91 40 27192778
Fax: +91 40 27160591
Email: kanwal_ash@ccmb.res.in

Sanjeev Bhaskar
Wellcome Trust Sanger Institute
Exon Resequencing
Wellcome Trust Genome Campus
Hinxton, Cambridge
CB10 1SA
UK
Phone: +44 1223 834244
Email: sb12@sanger.ac.uk

Ewan Birney
Wellcome Trust Genome Campus
European Bioinformatics Institute
Hinxton, Cambridge
CB10 1SD
UK
Phone: +44 1223 494420
Fax: +44 1223 494468
Email: birney@ebi.ac.uk

Janusz Blasiak
University of Lodz
Department of Molecular Genetics
Banacha 12/16
90-237 Lodz
Poland
Phone: +48 42 6354334
Fax: +48 42 6354484
Email: januszb@biol.uni.lodz.pl

Elena Bosch
Pompeu Fabra University
Dept. of Experimental Sciences and Health
C/ Aiguader 88
08003 Barcelona
Spain
Phone: +34 93 3160841
Fax: +34 93 3160901
Email: elena.bosch@upf.edu

Nina Bosch
Center for Genomic Regulation
Genes and Disease
Doctor Aiguader 88
8003
Barcelona
Spain
Phone: +34 93 3160139
Fax: +34 93 3160099
Email: nina.bosch@crg.es

Maria Brión
University of Santiago de Compostela
CeGen
San Francisco s/n
15782 Santiago de Compostela
Spain
Phone: +34 98 1582327
Fax: +34 98 1580336
Email: brioniml@usc.es

Anthony J Brookes
University of Leicester
Department of Genetics
University Road
Leicester, LE1 7RH
UK
Phone: +44 116 2523401
Fax: +44 116 2523378
Email: ajb97@leicester.ac.uk

Jasper Brugts
Erasmus Medical Center
Cardiology
Kralinger Esch 244
3063 NB Rotterdam
Netherlands
Phone: +31 10 4634687
Email: j.brugts@erasmusmc.nl

Anna Brunet
Centre for Genomic Regulation
Genes and Disease Program and CeGen
Dr. Aiguader, 88
8003 Barcelona
Spain
Phone: +34 93 3160105
Fax: +34 93 3160065
Email: anna.brunet@crg.es

Tiffany Brunson
Morehouse School of Medicine
Cardiovascular Research Institute
720 Westview Drive S.W.
Atlanta, Georgia 30310-1495
USA
Phone: +1 516 6426426
Fax: +1 404 7521042
Email: tbrunson@msm.edu

Mariona Bustamante
University of Barcelona
Genetics
Av. Diagonal 645
Spain
Phone: +34 93 4021794
Fax: +34 93 4034420
Email: mbustamante@ub.edu

Anton Buzdin
Shemyakin-Ovchinnikov Institute of Bioorganic Chemistry
Russian Academy of Sciences
Miklukho-Maklaya, 16/10
Moscow 117997
Russian Federation
Phone: +7 495 3306574
Fax: +7 495 3306538
Email: bu3din@mail.ru

Patrick Cahan
Washington University in Saint Louis
Department of Internal Medicine
Campus Box 8007
660 South Euclid Avenue
St. Louis, MO 63110
USA
Phone: +1 314 7474436
Fax: +1 314 3629333
Email: pcahan@wustl.edu

Francesc Calafell
Universitat Pompeu Fabra
Departament de Ciències Experimentals i de la Salut
Dr. Aiguader 88
08003 Barcelona
Spain
Phone: +34 93 316.08.42
Fax: +34 93 316.09.01
Email: francesc.calafell@upf.edu

Anne Cambon-Thomsen
CNRS
Faculté de médecine
37 allées Jules Guesde
F-31073 Toulouse Cedex
France
Phone: +33 5 61145959
Fax: +33 5 61145623
Email: cambon@cict.fr

Danielle Carpenter
University of Leeds
MH Investigation Unit
8.2, Clinical Sciences Building
St James University Hospital
Leeds, LS97TF
UK
Phone: +44 113 2065274
Email: d.carpenter@leeds.ac.uk

Angel Carracedo
Galician Foundation of Genomic Medicine
Molecular Medicine Unit
15782 Santiago de Compostela
Spain
Phone: +34 600942293
Fax: +34 98 1580336
Email: apimlang@usc.es

Christopher Carter
176 Downs Road
Hastings
East Sussex
UK
Phone: +44 1424 432762
Email: chriscar@polygenicpathways.co.uk

Ferran Casals
Universitat Pompeu Fabra
Unitat de Biologia Evolutiva
Parc de Recerca Biomèdica
Carrer Dr Aiguader 88
Barcelona 08003
Spain
Phone: +34 93 3160845
Fax: +34 93 3160901
Email: ferran.casals@upf.edu

Isfahan Chambers
Morehouse School of Medicine
Cardiovascular Research Institute
720 Westview Drive
ATL, GA 30310
USA
Phone: +1 347 6135035
Email: ichambers@msm.edu

TingFung Chan
University of California - San Francisco
Cardiovascular Research Inst and Inst for Human Genetics
513 Parnassus Ave.
HSW-901A, Box 0793
San Francisco, CA 94143-0793
USA
Phone: +1 415 5143879
Fax: +1 415 4762986
Email: tingfung.chan@ucsf.edu

Stephen Chanock
National Cancer Institute
Advanced Technology Center
8717 Grovemont Circle
Bethesda, MD 20892-4605
USA
Phone: +1 3014357559
Fax: +1 301 4023134
Email: chanocks@mail.nih.gov

Fadi Charchar
University of Leicester
Cardiovascular Science, Glenfield Hospital
Leicester LE3 9QP
UK
Phone: +44 116 2544562
Email: fjc5@le.ac.uk

Carole Charlier
University of Liège
Centre for Biomedical Integrative Genoproteomics
GIGA Tower (B34 - 1° floor)
CHU, Sart Tilman Campus
1, Av de l'Hôpital - 4000 Liège
Belgium
Email: carole.charlier@ulg.ac.be

Vivian Cheung
University of Pennsylvania
Departments of Pediatrics and Genetics
Abramson Research Center, Room 516 G
3516 Civic Center Blvd.
Philadelphia, PA 19104
USA
Phone: +1 215 5904950
Fax: +1 215 5903709
Email: vcheung@mail.med.upenn.edu

George Church
Harvard Medical School, MIT
Lipper Center for Computational Genetics
NRB room 238, 77 Avenue Louis Pasteur
Boston, MA 02115
USA
Phone: +1 617 4327562
Fax: +1 617 4326513
Email: gmc@harvard.edu

Jim Collins
Affymetrix
3420 Central Expressway
Santa Clara, CA 95051
USA
Phone: +1 408 7315896
Email: jim_collins@affymetrix.com

Roger Colobran
Banc de Sang i Teixits (BST)
Universitat Autònoma de Barcelona
Departament de Biologia Cel·lular Fisiologia i Immunologia
Hospital Germans Trias i Pujol. Edifici de Recerca.
Badalona (Barcelona)
Spain
Phone: +34 93 4978660
Fax: +34 93 4978668
Email: rogercol@yahoo.com

Donald F Conrad
University of Chicago
Department of Human Genetics
CLSC 507
920 E. 58th Street
Chicago, IL 60637
USA
Phone: +1 773 8349838
Fax: +1 773 8340505
Email: Conrad@uchicago.edu

Nancy Cox
University of Chicago
Biological Sciences Division AMB W607A
5841 S. Maryland Ave
USA
Phone: +1 773 8341001
Fax: +1 773 7022567
Email: ncox@bsd.uchicago.edu

Pio d'Adamo
IRCCS "Burlo Garofolo"
Medical Genetics
via Dell'Istria 65/1
34137 Trieste
Italy
Phone: +39 040 3785538
Fax: +39 040 3785540
Email: dadamo@burlo.trieste.it

Mauro D'Amato
Karolinska Institutet
Dept Biosciences and Nutrition
Halsovagen 7-9, 14157 Huddinge, Stockholm
Sweden
Phone: +46 8 6089143
Email: mauro.damato@ki.se

Johannes Dapprich
Generation Biotech
32 Pin Oak Drive
Lawrenceville, NJ 08648
USA
Phone: +1 609 6370878
Fax: +1 609 6379483
Email: jdapprich@generationbiotech.com

Ariel Darvasi
Hebrew University of Jerusalem
Dept. of Genetics
Jerusalem 91904
Israel
Phone: +972 2 6584303
Fax: +972 2 5671179
Email: arield@cc.huji.ac.il

Subhajyoti De
MRC Laboratory of Molecular Biology
Structural Studies Division
Hills Road, Cambridge CB2 2QH
UK
Phone: +44 7809629481
Email: sde@mrc-lmb.cam.ac.uk

Francisco De La Vega
Applied Biosystems
Bioinformatics R&D
850 Lincoln Centre Dr
Foster City, CA 94404
USA
Phone: +1 650 6386989
Fax: +1 650 5542577
Email: delavefm@appliedbiosystems.com

Paul Dear
MRC Laboratory of Molecular Biology
Genomics group
Hills Road
Cambridge, CB2 2QH
UK
Phone: +44 7903 124940
Fax: +44 1223 412178
Email: phd@mrc-lmb.cam.ac.uk

Emmanouil Dermitzakis
Wellcome Trust Sanger Institute
Population and Comparative Genomics
Hinxton, Cambridge
CB10 1SA
UK
Phone: +44 1223 494866
Fax: +44 1223 494919
Email: md4@sanger.ac.uk

Andrew Devereau
National Genetics Reference Laboratory
Informatics
St Mary's Hospital
Hathersage Road
Manchester
UK
Phone: +44 161 2768703
Fax: +44 161 2766606
Email: andrew.devereau@cmmc.nhs.uk

Igor Deyneko
Helmholtz Centre for Infection Research
Department of Genome Analysis
Inhoffenstraße 7
D-38124 Braunschweig
Germany
Email: ide@helmholtz-hzi.de

Hernan Dopazo
Centro de Investigación Príncipe Felipe
Bioinformatics
c/ ep Avda. Autopista del Saler 16 (Junto al Oceanográfico)
46013, Valencia.
Spain
Phone: +34 96 3289680
Fax: +34 96 3289701
Email: hdopazo@cipf.es

Peg Eis
NimbleGen Systems, Inc
Business Development
1 Science Ct
Madison, WI
USA
Phone: +1 608 2187674
Fax: +1 608 2187601
Email: peis@nimblegen.com

Xavier Estivill
Center for Genomic Regulation
Genes and Disease Program
Passeig Maritim 37-49
08003, Catalonia
Spain
Phone: +34 93 2240959
Fax: +34 93 2240899
Email: xavier.estivill@crg.es

Israel Fernandez
Institut de recerca, Hospital Vall d'Hebron
Neurovascular lab
Passeig Vall d'Hebron 119-129
Spain
Phone: +34 93 4894029
Email: israelcadenas@yahoo.es

Olga Fernando
Universitat Pompeu Fabra
Departament de Ciències Experimentals i de la Salut
PRBB - Unitat de Biologia Evolutiva
Doctor Aiguader 88
08003 Barcelona
Spain
Phone: +34 933 160818
Fax: +34 933 160901
Email: olga.fernando@upf.edu

Lars Feuk
The Hospital for Sick Children
Program in Genetics and Genomic Biology
101 College Street
MaRS Centre - East Tower, Room 14-701
Canada
Phone: +1 416 8137654 ext 1358
Fax: +1 416 8138319
Email: lfeuk@rogers.com

Louisa Flintoft
Nature Publishing Group
Nature Reviews Genetics
MacMillan Building
4 Crinan Street
London N1 9XW
UK
Phone: +44 2078434516
Email: l.flintoft@nature.com

Elodie Gazave
Universitat Pompeu Fabra
Departament de Ciències Experimentals i de la Salut
Doctor Aiguader, 88
08003 Barcelona
Spain
Phone: +34 93 3160845
Fax: +34 93 3160901
Email: elodie.gazave@upf.edu

Ilse Gijselinck
University of Antwerp - CDE
VIB - Department of Molecular Genetics
Universiteitsplein 1
BE-2610 Antwerpen
Belgium
Email: ilse.gijselinck@ua.ac.be

Juan González
Center for Genomic Regulation
Genes and Disease Program
Plaça Charles Darwin S/N - PRBB
08003 Barcelona
Spain
Phone: +34 93 3160139
Fax: +34 93 3160099
Email: juanramon.gonzalez@crg.es

Esteban González-Burchard
University of California, San Francisco
Departments of Biopharmaceutical Sciences & Medicine
San Francisco General Hospital
Box 2911
USA
Phone: +1 415 5149677
Fax: +1 415 5144365
Email: esteban@sfgh.ucsf.edu

Anna González-Neira
Spanish National Cancer Centre
Genotyping Unit. Human Cancer Genetics Programme
C/ Melchor Fernández Almagro 3 - 28029 Madrid
Spain
Phone: +34 91 2246974
Fax: +34 91 2246923
Email: agonzalez@cnio.es

Jenny Göransson
Uppsala University
Department of Genetics & Pathology
Rudbeck Laboratory
SE-751 85 Uppsala
Sweden
Phone: +46 18 4714816
Fax: +46 18 4714808
Email: jenny.goransson@genpat.uu.se

Carolin Gräbsch
Helmholtz Centre for Environmental Research - UFZ
Department for Human Exposure Research and Epidemiology
Permoserstraße 15 / 04318 Leipzig
Germany
Email: carolin.graebsch@ufz.de

Mònica Gratacòs
Center for Genomic Regulation
Dr. Aiguader, 88
08003 - Barcelona
Spain
Phone: +34 93 2240951
Email: monica.gratacos@crg.es

Hákon Guðbjartsson
deCODE Genetics
Sturlugata 8
101 Reykjavík
Iceland
Phone: +354 570 1900
Email: hakon@decode.is

Jeff Gulcher
deCODE Genetics
Sturlagata 8
Reykjavik, Iceland 101
Iceland
Phone: +354 898 4976
Email: jgulcher@decode.is

Ivo Gut
Centre National de Genotypage
Department of Technology Development
2 rue Gaston Cremieux
91057 Evry
France
Phone: +33 160 878400
Fax: +33 160 878383
Email: ivogut@cng.fr

Hakon Hakonarson
Children's Hospital of Philadelphia
34th and Civic Center Blvd
Philadelphia PA, 19104
USA
Phone: +1 267 4260088
Fax: +1 267 4260363
Email: hakonarson@chop.edu

Ira Hall
Cold Spring Harbor Laboratory
One Bungtown Road
Cold Spring Harbor, NY, 11724
USA
Phone: +1 516 2873764
Fax: +1 516 3678381
Email: hall@cshl.edu

Pille Hallast
Institute of Molecular and Cell Biology, University of Tartu
Department of Biotechnology
23 Riia St.; 51010 Tartu
Estonia
Phone: +372 7 375009
Fax: +372 7 420286
Email: pille.hallast@ut.ee

Thomas Hansen
Research institut of biological psychiatry
Sct. Hans Hospital, Copenhagen University hospital
Boserupvej 2
DK-4000 Roskilde
Denmark
Phone: +45 46334953
Fax: +45 46334367
Email: thomas.hansen@shh.regionH.dk

Charlotte N. Henrichsen
University of Lausanne
Centre for Integrative Genomics
Batiment "Le Genopode"
Quartier Sorge
CH-1015 Lausanne
Switzerland
Phone: +41 21 6923967
Fax: +41 21 6923965
Email: charlotte.henrichsen@unil.ch

Stephanie Hesselson
UC San Francisco
CVRI, Room 901A, 513 Parnassus Ave
San Francisco, CA
USA
Phone: +1 608 2399164
Email: stephanie.hesselson@ucsf.edu

Kirk Hogan
University of Wisconsin
Department of Anesthesiology
B6/319 Clinical Science Center
600 Highland Avenue
Madison, Wisconsin
USA
Phone: +1 608 2638100
Fax: +1 608 2630575
Email: khogan@facstaff.wisc.edu

Ed Hollox
University of Leicester
Genetics
Adrian Building, University Road
Leicester LE1 7RH
UK
Phone: +44 116 2523407
Email: ejh33@le.ac.uk

Matthew Hurles
Wellcome Trust Sanger Institute
Human Genetics
Wellcome Trust Genome Campus
Hinxton, Cambridge, CB10 1SA
UK
Phone: +44 1223 495377
Fax: +44 1223 494919
Email: meh@sanger.ac.uk

Michael Inouye
Wellcome Trust Sanger Institute
High-throughput Genotyping
Wellcome Trust Genome Campus
Hinxton, Cambridge, CB10 1SA
UK
Phone: +44 1223 494717
Fax: +44 1223 494919
Email: mi1@sanger.ac.uk

Iuliana Ionita
Harvard University, Biostatistics
Department Of Biostatistics
Harvard School of Public Health
Boston MA 02115
USA
Phone: +1 201 7240891
Fax: +1 617 4325619
Email: iionita@hsph.harvard.edu

Michael James
Queensland Institute of Medical Research
Genetic Epidemiology
300 Herston Road
Qld 4006
Australia
Email: michael.james@qimr.edu.au

Aslaug Jonasdottir
deCODE Genetics
Genomics and Molecular Technologies
Sturlugata 8
IS-101 Reykjavik
Iceland
Phone: +354 5701942
Fax: +354 570 1903
Email: aslaug@decode.is

Maria Kapasa
Biomedical Research Foundation
Bioinformatics
Soranou Efessiou 4
11527 Athens
Greece
Phone: +30 210 6597474
Fax: +30 210 6597545
Email: mkapasa@bioacademy.gr

Masaru Katoh
National Cancer Center, Japan
Genetics and Cell Biology Section
5-1-1 Tsukiji, Chuo-ku, Tokyo 104-0045
Japan
Email: mkatoh-kkr@umin.ac.jp

John Kauwe
Washington University School of Medicine
Department of Psychiatry
Campus Box 8134 660 S. Euclid
St. Louis MO 63110-1093
USA
Phone: +1 314 7472984
Fax: +1 314 7472983
Email: keoni@icarus.wustl.edu

Namik Kaya
King Faisal Specialist Hospital and Research Center
KFSHRC, Genetics Department, MBC:03, PO Box 3354
Riyadh, 11211
Saudi Arabia
Phone: +966 1 4647272 ext 39612
Email: nkaya@kfshrc.edu.sa

Veronique Kiermer
Nature Publishing Group
Nature Methods
75 Varick Street, 9th Floor
New York, NY 10013
USA
Phone: +1 212 7269388
Email: v.kiermer@natureny.com

Jong-Won Kim
Samsung Medical Center, Sungkyunkwan University
Department of Laboratory Medicine and Genetics
50 Ilwon-dong, Kangnam-gu
Seoul, 135-710
Korea
Phone: +82 2 34102705
Fax: +82 2 34102719
Email: jwonk@smc.samsung.co.kr

Toshio Kojima
RIKEN
Genomic Sciences Center
1-7-22 Suehiro-cho, Tsurumi-ku, Yokohama
Japan
Email: tkojima@gsc.riken.jp

Eloise Kok
Forensic Medicine
University of Tampere, Medical School
Medisiinarinkatu 3
33014 University of Tampere
Finland
Phone: +358 442 936236
Fax: +358 3 35517511
Email: eloise.kok@uta.fi

Jussi Kollin
Helsinki Institute for Information Technology
P.O. Box 68 (Gustaf Hällströmin katu 2b)
FI-00014 University Of Helsinki
Finland
Phone: +358 9 19151288
Fax: +358 9 19151120
Email: jussi.kollin@cs.helsinki.fi

Jan Korbel
Yale University
Yale University Center for Excellence in Genome Science
266 Whitney Ave. PO Box 208114
New Haven, CT 06520
USA
Phone: +1 203 4325405
Fax: +1 203 4325175
Email: jan.korbel@yale.edu

Patricia Kramer
Oregon Health and Science University
Neurology CR131
3181 SW Sam Jackson Park Road
Portland OR 97239
USA
Phone: +1 503 4947196
Fax: +1 503 4947499
Email: kramer@ohsu.edu

Gregory Kryukov
Brigham and Women's Hospital / Harvard Medical School
Division of Genetics
New Research Building, Room 0464
77 Avenue Louis Pasteur
Boston, MA 02125, USA
USA
Phone: +1 617 5254757
Fax: +1 617 5254705
Email: gkryukov@rics.bwh.harvard.edu

Pui-Yan Kwok
University of California, San Francisco
513 Parnassus Ave. Box 0793, HSW-901G
San Francisco, CA 94143-0793
USA
Phone: +1 415 5143802
Fax: +1 415 4762956
Email: Pui.Kwok@ucsf.edu

Maris Laan
University of Tartu
Institute of Molecular and Cell Biology
Riia 23, 51010 Tartu
Estonia
Phone: +372 7374636
Fax: +372 7419914
Email: maris@ebc.ee

Hafid Laayouni
Universitat Pompeu Fabra
Departament de Ciències Experimentals i de la Salut
Dr. Aiguader, 88
08003 Barcelona
Spain
Phone: +34 93 3160845
Fax: +34 93 3160901
Email: hafid.laayouni@upf.edu

Stephen Laderman
Agilent Laborartories
5301 Stevens Creek Blvd. MS 54U-SB
Santa Clara, CA 95051-7201
USA
Phone: +1 408 5537302
Email: steve_laderman@agilent.com

Thomas LaFramboise
Case Western Reserve University School of Medicine
10900 Euclid Ave; BRB 630
Cleveland, OH 44106
USA
Phone: +1 216 3680150
Fax: +1 216 3683432
Email: thomas.laframboise@case.edu

Christoph Lange
Harvard School of Public Health
Biostatistics
655 Huntington Ave
Boston, MA 02115
USA
Phone: +1 617 9906068
Fax: +1 617 4325619
Email: clange@hsph.harvard.edu

Charles Lee
Brigham and Women's Hospital / Harvard Medical School
Pathology
221 Longwood Avenue
LMRC - Room 412A
Boston, MA 02115
USA
Phone: +1 617 2780031
Fax: +1 617 2645176
Email: clee@rics.bwh.harvard.edu

Heikki Lehvaslaiho
University of Western Cape
South African National Bioinformatics Institute
Modderdam Road
Private Bag X17
Belville
South Africa
Phone: +27 21 9592096
Fax: +27 21 9592512
Email: heikki@sanbi.ac.za

Samuel Levy
J. Craig Venter Institute
The Center for the Advancement of Genomics
9704 Medical Center Drive
Rockville, MD 20850
USA
Phone: +1 240 2682789
Fax: +1 240 2684000
Email: slevy@venterinstitute.org

Ling-Hui Li
Academia Sinica
Institute of Biomedical Sciences
128, Sec. 2, Academia Rd, Taipei
Taiwan, China
Phone: +886 2 27825258 Ext 163
Fax: +886 2 27824066
Email: lli@ibms.sinica.edu.tw

Yung-Feng Lin
Institute of Genome Sciences
National Yang-Ming University
No. 155, Sex. 2, Linong st., Beitou District
Taipei City 112, Taiwan R.O.C.
Taiwan, China
Phone: +886 2 28267000#6071
Fax: +886 2 2800552
Email: lyf@nhri.org.tw

Si Lok
University of Hong Kong
Genome Research Centre
L6-35, Laboratory Block, Li Ka Shing Faculty of Medcine
21 Sassoon Road, Pokulam, Hong Kong
PR China
Phone: +86 852 28199837
Fax: +86 852 28185653
Email: silok@hkusua.hku.hk

Ruy Lopez-Ridaura
National Institute of Public Health
Av Universidad 655
62100, Cuernavaca, Morelos
Mexico
Phone: +52 777 3112343
Fax: +52 777 3111148
Email: rlridaura@correo.insp.mx

Belén Lorente
Universitat Pompeu Fabra
Departament de Ciències Experimentals i de la Salut
Dr. Aiguader 88 (08003) Barcelona
Spain
Phone: +34 93 3160818
Fax: +34 93 3160901
Email: mlorente@prbb.org

Per Lundmark
Uppsala University
Medical Sciences, Molecular medicine
Entrance 70, 3rd floor, Research dept. 2
University Hospital
SE-751 85 UPPSALA
Sweden
Phone: +46 18 6114931
Fax: +46 18 553601
Email: per.lundmark@medsci.uu.se

Reedik Mägi
University of Tartu
Departmend of Bioinformatics
Riia str. 23, Tartu 51010
Estonia
Phone: +372 51985228
Fax: +372 7420286
Email: reedik.magi@ut.ee

Vsevolod Makeev
Institute of Genetics and Selection of Industrial Microorganisms
Laboratory of Bioinformatics (LIX)
91128 Palaiseau Cedex
1st Dorozhny proezd 1
Moscow, 117545
Russia
Phone: +7 495 3150156
Fax: +7 495 3150501
Email: vsevolod_makeev@mail.ru

Urko Martinez
Universitat Pompeu Fabra
Departament de Ciències Experimentals i de la Salut
c/ Dr. Aiguader 88 (08003) Barcelona
Spain
Phone: +34 93 3160818
Fax: +34 93 3160901
Email: urko.martinez@upf.edu

Cheryl Maslen
Oregon Health and Science University
Molecular and Medical Genetics
Mail code L465
3181 SW Sam Jackson Park Road
Portland, Oregon 97239
USA
Phone: +1 503 4942011
Fax: +1 503 4946986
Email: maslenc@ohsu.edu

Frank McCaughan
University College London
Centre for Respiratory Research
University College London
WC1E 6JF
UK
Phone: +44 20 76796975
Fax: +44 20 76796975
Email: frankmc@mrc-lmb.cam.ac.uk, f.mccaughan@ucl.ac.uk

Josep-Maria Mercader
Center for Genomic Regulation
Dr. Aiguader, 88, 08003 Barcelona - Catalunya
Spain
Phone: +34 93 3160105
Fax: +34 93 3160105
Email: josepmaria.mercader@crg.es

Montserrat Milà
Hospital Clínic
Biochemistry and Molecular Genetics Department
c/Villarroel, 170, 08036 Barcelona
Spain
Phone: +34 93 2275400 ext 2784/3406
Fax: +34 93 4515272
Email: mmila@clinic.ub.es

Dimitri Monos
CHOP/UPENN
3615 Civic Center Blvd.
Abramson Research Center, Room 1208B
Philadelphia, PA 19104
USA
Phone: +1 215 5901449
Fax: +1 215 5906361
Email: monosd@email.chop.edu

María-Cristina Morán Moguel
Instituto Mexicano del Seguro Social
Centro de Investigación Biomédica de Occidente
Sierra Mojada No. 800, Colonia Independencia
Guadalajara, Jalisco. 44340
Mexico
Phone: +52 33 36189410
Fax: +52 33 36181756
Email: cmmoguel@yahoo.com.mx

Jonathan Mudge
Wellcome Trust Sanger Institute
Hinxton, Cambridge CB10 1HH
UK
Phone: +44 1223 496837
Fax: +44 1223 496802
Email: jm12@sanger.ac.uk

Margarita Muiños-Gimeno
Centre for Genomic Regulation
Dr. Aiguader, 88
08003 Barcelona - Catalunya
Spain
Phone: +34 656254643
Email: margarita.muinos@crg.es

Edvin Munk
Sequenom
Mendelssohnstr. 15 D, 22761 Hamburg
Germany
Phone: +49 40 8996760
Email: emunk@sequenom.com

Robin Munro
InforSense, Colet Court
100 Hammersmith Road
London, W6 7JP
UK
Phone: +44 20 82378440
Fax: +44 20 82378441
Email: rmunro@inforsense.com

Masaaki Muramatsu
Tokyo Medical and Dental University
Molecular Epidemiology
2-3-10 Kandasurugadai, Chiyoda-ku, Tokyo, 101-0062
Japan
Email: muramatsu.epi@mri.tmd.ac.jp

Muhammad Naeem
National University of Sciences & Technology (NUST)
Dept of Biochemistry & Molecular Biology
NUST/Army Medical College
Abid Majeed Road, Rawalpindi
Pakistan
Phone: +92 3335324922, +92 5156132787
Fax: 92519272502
Email: mnaeem73@gmail.com

Kimitoshi Naito
Japan Biological Informatics Consortium
Strategic Planning Department
Time24 Bldg. 10f
Aomi2-45, Koto-Ku
Tokyo
Japan
Phone: +81 3 55318550
Fax: +81 3 55311560
Email: naito@jbic.or.jp

Arcadi Navarro
ICREA and Universitat Pompeu Fabra
Unitat de Biologia Evolutiva
C/ Dr. Aiguader 88
08003 Barcelona
Spain
Phone: +34 93 3160844
Fax: +34 93 3160901
Email: arcadi.navarro@upf.edu

Debbie Nickerson
University of Washington
Department of Genome Sciences
Foege S213B, Box 355065
Seattle, WA 98195
San Francisco, CA 94143
USA
Phone: +1 206 6857387
Fax: +1 206 2216498
Email: debnick@u.washington.edu

Tiit Nikopensius
Institute of Molecular and Cell Biology, University of Tartu
Biotechnology
Riia 23, 51010, Tartu
Estonia
Phone: +372 7 375034
Fax: +372 7 420286
Email: tiitn@ebc.ee

Silje Nordgard
Norwegian Cancer Hospital & University of Oslo
Dep of Genetics
Montebello
N-0310 Oslo
Norway
Phone: +47 98 445786
Fax: +47 22 934440
Email: nordgard@ulrik.uio.no

Karen Nuytemans
Universiteit Antwerpen
VIB - Department Molecular Genetics
Universiteitsplein 1
BE-2610 Antwerpen (Wilrijk)
Belgium
Phone: +32 3 2651039
Fax: +32 3 2651012
Email: karen.nuytemans@ua.ac.be

Avi Orr-Urtreger
Tel Aviv Sourasky Medical Center
Genetic Institute
6 Weizmann St
Tel Aviv, 64239
Israel
Phone: +972 3 6974905
Fax: +972 3 6974555
Email: aviorr@tasmc.health.gov.il

James Ostell
National Library of Medicine
Information Engineering
45 Center Drive Room 5as51
Bethesda MD 20892-6510
USA
Phone: +1 301 4355978
Fax: +1 301 4802918
Email: ostell@ncbi.nlm.nih.gov

Bernice Packer
National Cancer Institute
Core Genotyping Facility
8717 Grovemont Circle
Bethesda, MD 20892-4605
USA
Phone: +1 301 4966019
Fax: +1 301 4023134
Email: packerb@mail.nih.gov

Dimitri Pappaioannou
KREATECH Biotechnology
Product Development
Vlierweg 20
1032 LG Amsterdam
The Netherlands
Phone: +31 20 6919181
Fax: +31 20 6963531
Email: d.pappaioannou@kreatech.com

Bruno Paquin
Genizon BioSciences
Science & Technology
880 McCaffrey
St. Laurent, Quebec, H4T 2C7
Canada
Phone: +1 514 2703991 ext 247
Fax: +1 514 2700209
Email: bruno.paquin@genizon.com

Laurence Parnell
Human Nutrition Research Center on Aging
Nutrition and Genomics Laboratory
711 Washington Street
Boston, MA 02111
USA
Phone: +1 617 5563089
Fax: +1 617 5563211
Email: laurence.parnell@ars.usda.gov

Andrzej Pawlak
Institute of Human Genetics, Polish Academy of Sciences
Department of Function of Nucleic Acids
Strzeszynska 32 str., 60-479 Poznan
Poland
Phone: +48 616579227
Fax: +48 618233235
Email: pawlakal@man.poznan.pl

Luís Pérez-Jurado
Universitat Pompeu Fabra
Departament de Ciències Experimentals i de la Salut
c/ Dr. Aiguader 88 (08003) Barcelona
Spain
Phone: +34 93 3160818
Fax: +34 93 3160901
Email: luis.perez@upf.edu

George Perry
Brigham and Women's Hospital / Harvard Medical School
221 Longwood Avenue
LMRC - Room 404
Boston, MA 02115
USA
Phone: +1 617 2780064
Fax: +1 617 2645176
Email: pj.perry@asu.edu

Chris Ponting
Oxford University
MRC Functional Genetics Unit
Le Gros Clark Building
South Parks Road
Oxford OX1 3QX
UK
Phone: +44 1865 285855
Fax: +44 1865 285862
Email: chris.ponting@anat.ox.ac.uk

Boode Prathap Naidu
Centre for Cellular and Molecular Biology
Department of Evolutionary Biology and Medical Genetics
Uppal Road-500 007
Hyderabad, Andhra Pradesh
India
Phone: +91 40 27192636
Fax: +91 40 27160311
Email: bpn_naidu@ccmb.res.in

Tarmo Puurand
University of Tartu
Bioinformatics
Riia 23, 51010, Tartu
Estonia
Phone: +372 7375002
Fax: +372 7420286
Email: tarmo.puurand@ut.ee

Tasleem Raza
King George's Medical University
Post Graduate Department of Pathology
Genetics unit, PG Dept of Pathology
KGMU
Lucknow 226003
India
Phone: +91 9839179527
Email: tasleem24@gmail.com

Rolf Reist
Sequenom
Calle Cerro de la Carrasqueta 63
Portal D, 2 B
28035 Madrid
Spain
Phone: +34 91 3763759
Email: rreist.biobt@gmail.com

Marta Ribasés-Haro
Hospital Universitari Vall d'Hebron
Department of Psychiatry
Pg. Vall d'Hebron 119-129, 08035 Barcelona
Spain
Phone: +34 93 2746734
Fax: +34 93 4894587
Email: marta.ribases@gmail.com

Susan Ring
University of Bristol
Social Medicine
ALSPAC
24 Tyndall Ave
Bristol BS8 1TQ
UK
Phone: +44 117 3311623
Fax: +44 117 3311729
Email: s.m.ring@bris.ac.uk

Daniel Rios
European Bioinformatics Institute
Wellcome Trust Genome Campus
Hinxton, CB10 1SD Cambridge
UK
Phone: +44 1223 494684
Fax: +44 1223 494468
Email: dani@ebi.ac.uk

Mercedes Robledo
Spanish National Cancer Centre
Hereditary Endocrine Cancer Group
Melchor Fernández Almagro 3 - 28029 Madrid
Spain
Phone: +34 91 2246948
Fax: +34 91 2246923
Email: mrobledo@cnio.es

Aurea Rodriguez-Lopez
Center for Genomic Regulation
Charles Darwin s/n PRBB Building
08003, Catalonia
Spain
Phone: +34 933160159
Fax: +34 933160099
Email: aurea.rodriguez@crg.es

Carsten Rosenow
Illumina
9885 Town Centre drive
San Diego, CA 92121
USA
Phone: +1 858 2024886
Fax: +1 858 2024802
Email: crosenow@Illumina.com

Allen Roses
GlaxoSmithKline, Pharmacogenetics
Five Moore Drive
Research Triangle Park, NC 27709
USA
Phone: +1 919 4837418
Fax: +1 919 3156013
Email: allen.d.roses@gsk.com, allen.roses@duke.edu

Janna Saarela
National Public Health Institute
Molecular Medicine
FIN-00251 Helsinki
Finland
Phone: +358 9 47448975
Email: janna.saarela@ktl.fi

Maria Sasvari-Szekely
Semmelweis University
Medical Chemistry, Mol. Biol. and Pathobiochem.
H-1888 Budapest, Puskin u 9
Hungary
Phone: +36 204664564
Email: sas@puskin.sote.hu

Ann Saunders
GlaxoSmithKline
3030 Cornwallis Road
Durham, NC 27709
USA
Phone: +1 919 4839349
Email: ann.m.saunders@gsk.com

Ester Saus
Centre for Genomic Regulation
Genes and Disease Program
Dr. Aiguader, 88
08003 - Barcelona
Spain
Phone: +34 93 3160105
Fax: +34 93 3160065
Email: ester.saus@crg.es

Stephen Scherer
The Hospital for Sick Children
Genetics & Genome Biology
MaRS Building, East Tower
101 College St., 14th Fl., Rm. 14-701
Toronto, ON M5G 1L7
Canada
Phone: +1 416 8137613
Fax: +1 413 8138319
Email: steve@genet.sickkids.on.ca

Jean-Jacques Schott
INSERM U533, l'institut du thorax
Cardiovascular Genetics
Faculté de médecine
1 rue Gaston Veil
44035 Nantes Cedex 01
France
Phone: +33 2 40412960
Fax: +33 2 40412950
Email: jjschott@nantes.inserm.fr

Renno Schuurmans
Genizon BioSciences Inc
Sterreboslaan 4
1272 PA Huizen
The Netherlands
Phone: +31 65 4968420
Fax: +31 35 6949623
Email: renno.schuurmans@genizon.com

Rao Sethumadhavan
Institute of Technology University
School of Biotechnology Chemical & Biomedical
Engineering Vellore
Vellore 632014, Tamil, Nadu
India
Phone: +91 416 2202522
Fax: +91 416 2243092
Email: sethu_rao@bsnl.in

Snaevar Sigurdsson
Uppsala University, Medical Sciences
Uppsala University Hosptial, entrance 70
3th floor, Foavd2, Molmed, Lab20
Sweden
Phone: +46 18 6114951
Email: snaevar.sigurdsson@medsci.uu.se

Martin Sikora
Universitat Pompeu Fabra
Facultat De Ciències De La Salut I De La Vida
Doctor Aiguader 88
08003 barcelona
Spain
Phone: +34 93 3160802
Email: martin.sikora@upf.edu

Andrew Skol
University of Chicago
5841 S Maryland Ave, AMB W611A
Chicago, IL 60637
USA
Phone: +1 773 8343623
Fax: +1 773 7022567
Email: askol@uchicago.edu

Beatriz Sobrino
University of Santiago de Compostela
Cegen
Hospital Clinico Universitario
Edificio consultas, planta -2
Choupana s/n
Spain
Phone: +34 98 1951490
Fax: +34 98 1951473
Email: beaiml@usc.es

Kyuyoung Song
Univ of Ulsan College of Ulsan
388-1 Poongnap-dong
Songpa-ku
Seoul
Korea
Phone: +82 2 30104277
Fax: +82 2 30104248
Email: kysong@amc.seoul.kr

Richard Spielman
University of Pennsylvania School of Medicine
Department of Genetics
Pennsylvania 19104
USA
Phone: +1 215 8985763
Fax: +1 215 5739598
Email: spielman@pobox.upenn.edu

Marija Stankovic
Institute of Molecular Genetics and Genetic Engineering
Laboratory for Molecular Biology
Vojvode Stepe 444a
P.O. Box 23, 11010 Belgrade
Serbia
Phone: +381 11 3976658
Fax: +381 11 3975808
Email: marijamst@yahoo.com

Lincoln Stein
Cold Spring Harbor Laboratory
1 Bungtown Road
Cold Spring Harbor, NY 11542
USA
Phone: +1 516 3678380
Fax: +1 516 3678389
Email: lstein@cshl.edu

Vadim Stepanov
Institute for Medical Genetics
Lab for Evolutionary Genetics
634050 Tomsk
Russian Federation
Phone: +7 3822 512228
Fax: +7 3822 513744
Email: vadim.stepanov@medgenetics.ru

Marc Sultan
Max Planck Institute for Molecular Genetics
Vertebrate Genomics
Ihnestr. 63/73
14195 Berlin
Germany
Phone: +49 3084131222
Fax: +49 3084131128
Email: sultan@molgen.mpg.de

Shamil Sunyaev
Brigham and Women's Hospital / Harvard Medical School
New Research Building, Room 0464
77 Avenue Louis Pasteur
Boston, MA 02125, USA
USA
Phone: +1 617 5254735
Fax: +1 617 5254705
Email: ssunyaev@rics.bwh.harvard.edu

Paul Tam
University of Hong Kong
Department of Surgery
Department of Surgery, University of Hong Kong Medical
Centre, Queen Mary Hospital, Pokfulam Road
Hong Kong SAR
Hong Kong, SAR, China
Phone: +852 28554850
Fax: +852 28173155
Email: paultam@hkucc.hku.hk

Stefan Taudien
Leibniz Institute of Age Research (Fritz Lipmann Institute)
Genome Analysis
Beutenbergstr. 11
D-07745 Jena
Germany
Phone: +49 3641 656258
Fax: +49 3641 656255
Email: stau@fli-leibniz.de

Albert Tenesa
University of Edinburgh
MRC Human Genetics Unit
Western General Hospital
Crewe Road South
UK
Phone: +44 1314678451
Email: albert.tenesa@ed.ac.uk

Gilles Thomas
National Cancer Institute
8717 Grovemont Circle
Gaithersburg MD 20877
USA
Phone: +1 301 4029326
Fax: +1 301 4023134
Email: thomasgi@mail.nih.gov

Amy H. Y. Tong
University of Hong Kong
Genome Research Centre
L6-35, Laboratory Block, Li Ka Shing Faculty of Medcine
21 Sassoon Road, Pokulam, Hong Kong
PR China
Phone: +86 852 28199311
Fax: +86 852 28185653
Email: amytong@hkucc.hku.hk

Barbara Trask
Fred Hutchinson Cancer Research Center
Human Biology Division
1100 Fairview Ave N, C3-168
Seattle WA 98109
USA
Phone: +1 206 6671470
Fax: +1 206 6674023
Email: btrask@fhcrc.org

Shih-Feng Tsai
National Health Research Institutes
Divison of Molecular & Genomic Medicine
35 Keyan Road, Zhunan Town, Miaoli 350
Taiwan, China
Phone: +886 37246166 ext 35300
Fax: +886 37586459
Email: petsai@nhri.org.tw

Marianne Tuefferd
Johnson & Johnson Pharmaceutical Research &
Development
Janssen Pharmaceutica NV
functional genomics
Turnhoutseweg 30
2340 Beerse
Belgium
Phone: +32 14607114
Fax: +32 14606111
Email: mtueffer@prdbe.jnj.com

Alexander Varzari
National Center of Reproductive Health and Medical
Genetics
Kishinev, Moldova
Burebista str. 82, MD 2062 Kishinev, Moldova
Moldova
Phone: +373 22 777960
Email: a_varsahr@yahoo.com

Joris Veltman
Radboud University Nijmegen Medical Centre
Human Genetics
Box 9101
6500 HB Nijmegen
Netherlands
Phone: +31 24 3614941
Fax: +31 24 3668752
Email: j.veltman@antrg.umcn.nl

Arpana Vibhuti
Institute of Genomics and Integrative biology(IGIB)
Functional Genomics Unit
Mall road, Delhi-110 007
India
Phone: +91 11 27666156
Fax: +91 11 27667471
Email: arpee11@rediffmail.com

Salma Wakil
King Faisal Specialist Hospital And Research Center
Functional Genomics Dept. Of Genetics
MBC 03, PO Box 3354 Riyadh 11211
Saudi Arabia
Phone: +966 509840019
Fax: +966 14424585
Email: smajid@kfshrc.edu.sa

Lauren Weiss
Massachusetts General Hospital
Center for Human Genetic Research
185 Cambridge St
6th floor
Boston, MA 02114
USA
Phone: +1 617 2766577
Fax: +1 617 7260830
Email: laweiss@chgr.mgh.harvard.edu

Phoebe White
Applied Biosystems
850 Lincoln Centre Drive
Foster City
California 94404
USA
Phone: +1 650 5543071
Email: phoebe.white@appliedbiosystems.com

Kirk Wilhelmsen
UNC-Chapel Hill
Genetics and Neurology
4109E Neuroscience Research Bldg
CB#7264
Chapel hill, NC 27599-7264
USA
Phone: +1 919 9661373
Fax: +1 919 9663630
Email: kirk@med.unc.edu

Hsin-Chou Yang
Academia Sinica
Institute of Statistical Science
No. 128, Sec. 2, Academia Road,
Nankang, 115, Taipei
Taiwan, China
Phone: +886 2 27835611 ext 113
Fax: +886 2 27823047
Email: hsinchou@stat.sinica.edu.tw

Meredith Yeager
National Cancer Institute
Core Genotyping Facility
8717 Grovemont Circle
Gaithersburg MD 20877
USA
Phone: +1 301 4357613
Fax: +1 301 4023134
Email: yeagerm@mail.nih.gov

Yum Lina Yip
Swiss Institute of Bioinformatics
Swiss-Prot group
1 Rue Michel-Servet
CH1211 Geneva
Switzerland
Phone: +41 22 3795049
Fax: +41 22 3795858
Email: lina.yip@isb-sib.ch

Changqing Zeng
Beijing Genomics Institute
Airport Industrial Zone, B-6
PR China
Phone: +86 10 80481146
Fax: +86 10 80498676
Email: czeng@genomics.org.cn

Chunyan Zhao
Karolinska Institutet
Dept. of Biosciences and Nutrition
Novum, Karolinska Institutet
141 57 Huddinge
Sweden
Phone: +46 8 6089273
Fax: +46 8 7745538
Email: chunyan.zhao@cnt.ki.se, chunyanzhao@hotmail.com

NOTES

NOTES

NOTES

NOTES

NOTES

NOTES

NOTES

Useful Meeting Information

Dolce Sitges Meeting Rooms

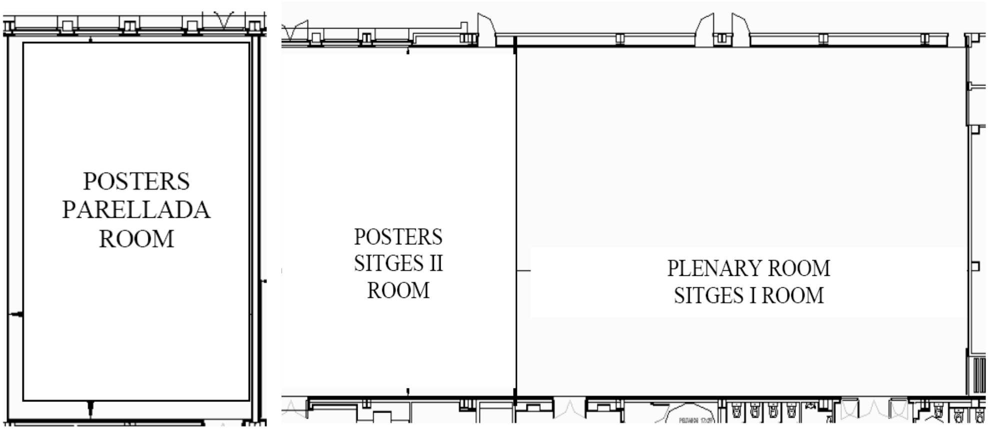

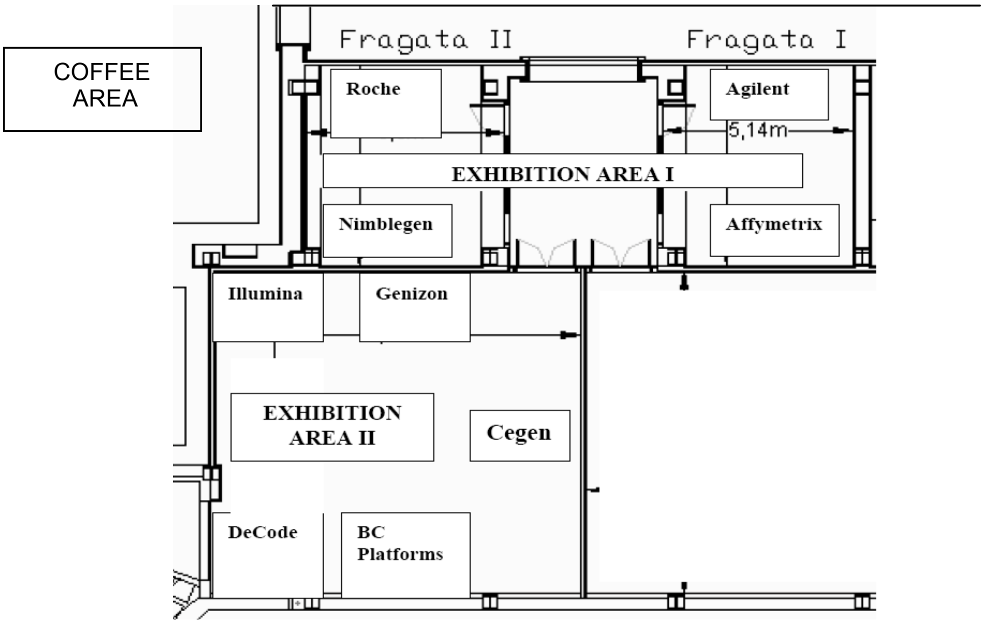

HOTEL DOLCE SITGES: conference venue and meeting accommodation

Address: Av. Cami de Miralpeix 12, Sitges 08870 Spain. Phone: +34 938 109 000

Official check in time is 14:00 hours, and check out time is 12:00 hours

The Hotel Dolce Sitges is a 5-star conference center and hotel, located in the town of Sitges about 25 km (~20 minutes drive) South from Barcelona airport. The hotel provides several swimming pools, a gym and a spa. The region is blessed with a gorgeous 4 km Mediterranean coastline (>20 different beaches) and many tourist attractions. Daily temperatures should be 18-25ºC. More information on the local area is available at http://www.sitgestour.com/informacio-util/index.php

TRAVEL TO THE CONFERENCE CENTER

A) FROM BARCELONA AIRPORT:

Conference representatives will be available at Barcelona airport (3 pm – 10 pm on Wednesday 5th September, and 8 am – 1 pm on Thursday 6th September) to help you transfer to the conference center. Upon arrival at the airport during these times, please go to the Botero' sculpture (the black fat horse) in terminal B where our staff will be waiting. They will direct you into group taxis and/or to shuttle busses as appropriate.

Alternatively, you can make your own way to the conference center, based upon the following information:

- TAXI: available outside the airport, cost about EURO 50, driver tipping not necessary (though permitted)

- BUS: to Sitges, departs hourly (07.40 - 23.40) from airport Terminal B, cost EURO 3, then take a taxi from the Sitges bus stop to the conference center (EURO 15).

- TRAIN: take the Barcelona train from the Airport station (13 and 43 minutes past each hour) to the first stop, "El Prat de Llobregat", and then catch a train heading towards "Vilanova i la Geltrú" which will stop at Sitges, cost EURO 3, then take a taxi from the Sitges train station to the conference center (EURO 10).

- CAR: Head in the direction of "Tarragona" following the motorway C-32 signs, and follow signs to Sitges. After the toll booths, take exit number 26 to "Sant Pere de Ribes - Vilanova". At the first roundabout, drive in the direction of "Sitges - Vilanova"; at the second roundabout follow the Sitges signs. Turn to the right following the Dolce Sitges signs at the Masia Can Girona Road.

A) FROM BARCELONA:

- TAXI: cost about EURO 70, driver tipping not necessary (though permitted)

- TRAIN: From the "Barcelona Sants" or the "Paseo de Gracia" train station, take the train heading towards "Vilanova- Sant Vicenç de Calders" or "Vilanova" which will stop at Sitges, cost EURO 3, then take a taxi from the Sitges train station to the conference center (EURO 10).

- CAR: Travel via "Gran Via de les Corts Catalanes" in the direction of "Tarragona" following the motorway C-32 signs, and follow signs to Sitges. After the toll booths, take exit number 26 to "Sant Pere de Ribes - Vilanova". At the first roundabout, drive in the direction of "Sitges - Vilanova"; at the second roundabout follow the Sitges signs. Turn to the right following the Dolce Sitges signs at the Masia Can Girona Road.

Arrangements for return travel from the conference center will be made during the meeting.

MEETING LOGISTICS

- The meeting will start after lunch (~2.30 pm) on Thursday 6th September and end 5.30 pm on Saturday 8th September.

- Lunches and coffee breaks will be provided on 6th, 7th and 8th September, and dinner for delegates staying in the hotel will be provided on 6th and 8th September. The main Conference Dinner is provided on 7th September as part of a visit to the Vallformosa winery (http://www.vallformosa.es/ang/html/index1.html) for those that have paid for this excursion. Other delegates must make their own arrangements for dinner that evening.

- Boards for poster presentations will measure 95 cm wide x 200 cm high. Tape and pins to attach the posters to the boards will be provided. Authors should put up the posters immediately after midday on Thursday 6th September, and dismantle them after 5 pm on Saturday 8th September.

- The official language of the Congress is English. No simultaneous translation will be provided.

- Free wireless internet connection will be provided throughout the conference center.

- The electricity supply is 220 volts, using standard European sockets of the round two-pin type.

- The currency of Spain is the Euro

- Local telephone information: Country code, +34; to call abroad, 00+; Police, 088; Fire Brigade, 080; Emergency, 112.

Abstracts book supported by the

Center for Genomic Regulation (CRG)

(www.crg.es)

HGV2007 - September 6-8, 2007

Hotel Dolce Sitges, Catalunya, Spain

http://hgv2007.nci.nih.gov

Cover Picture: Consol Bancells

Printed in Barcelona, Catalonia, Annus Domini MMVII

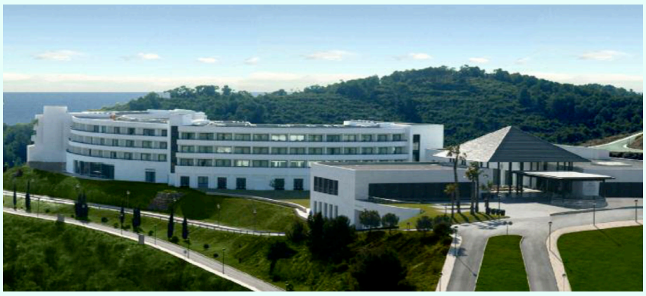

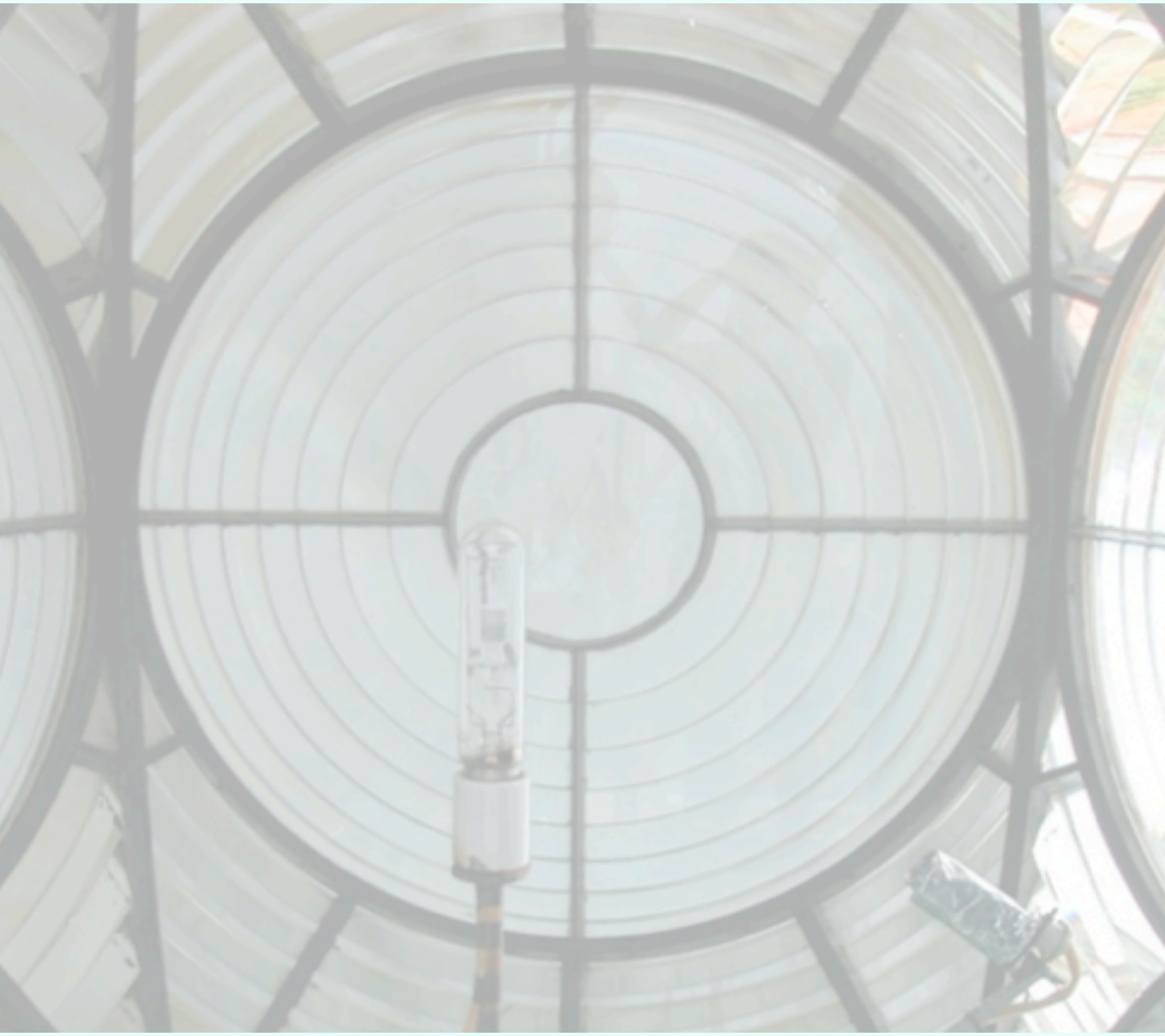

HGV2007

http://hgv2007.nci.nih.gov
