# Supplementary material for: SNPs Meet CNVs in Genome-Wide Association Studies: HGV2007 Meeting Report
Source: PLoS Genet. 2008 Apr 25;4(4):e1000068. doi: 10.1371/journal.pgen.1000068 (PMC2330071; doi:10.1371/journal.pgen.1000068)
Supplement: Text S2 — Full Meeting Report: An extended version of the meeting report (0.08 MB DOC) [file pgen.1000068.s002.doc]

**SNPs Meet CNVs in Genome-Wide Association Studies:**

**HGV2007 Meeting Report**

Xavier Estivill^1^, Nancy J. Cox^2^, Stephen J. Chanock^3^, Pui-Yan Kwok^4^, Stephen W. Scherer^5^, Anthony J. Brookes^6^

^1^Genes and Disease Program, Center for Genomic Regulation (CRG-UPF), and CIBERESP, Barcelona, Catalonia, Spain

^2^Departments of Medicine and Human Genetics, University of Chicago, Illinois, USA

^3^Division of Cancer Epidemiology and Genetics, and Center for Cancer Research, National Cancer Institute, Bethesda, Maryland, USA

^4^Department of Dermatology, Cardiovascular Research Institute, and Institute for Human Genetics, University of California, San Francisco, California, USA

^5^The Centre for Applied Genomics, Program in Genetics and Genomic Biology, Research Institute, The Hospital for Sick Children, Toronto, Canada

^6^Department of Genetics, University of Leicester, UK

The 9^th^ meeting on Human Genome Variation and Complex Genome Analysis was held in Sitges, Spain in September 2007. This annual meeting, which was originally focused on single nucleotide polymorphism (SNP), broadened its scope from 2006 onwards to encompass the entire range of genomic variability. Maintaining the relatively small format of 200 delegates, the meeting gathered leading investigators in copy number variation (CNV), SNP association studies, ultrasequencing, population genetics, statistical analysis and database management, as well as young investigators that have initiated their careers in these fields. The two-and-a-half-day meeting combined sponsorship by several academic institutions and corporate entities (http://hgv2007.nci.nih.gov/home.cfm) in a venue that facilitated interaction and communication between the participants.

The Sitges venue was particularly conducive to the formal and informal discussions that have long characterized this meeting. These discussions were wide-ranging, and while they often began in the meeting rooms, they usually continued through meals and into the evening.

A key focus was on how to facilitate the continued success of genome-wide association studies (GWAS). It has been widely appreciated that large-scale collaboration has been hugely beneficial to early GWAS, and there was considerable discussion at the meeting of the need to establish and maintain the bioinformatics infrastructure necessary to make maximum use of the data being generated. While initial efforts in the U.S. and Europe have made impressive inroads in serving both data and results of GWAS (summarized by a number of the participants – see below), the need for richer resources allowing integration of GWAS across more phenotypes, including expression phenotypes from multiple human tissues, was apparent. Such resources would allow more rapid and broad-based assessment of functional relationships among genetic variations (SNP and CNV), expression phenotypes, disease states, and related quantitative traits. There was also widespread sentiment that larger samples sizes, while clearly desirable, would, in the end, provide only a fraction of the contribution of genetic variation to complex disorders. Advances in analytic approaches coupling statistical genetics with bioinformatics may prove to be a fruitful avenue for extending results of GWAS.

There was vigorous discussion of the likely contribution of structural variation to human genetic disease. Although a number of participants confirmed the contribution of CNVs to various human disorders, known CNVs are highly skewed toward the lower end of the minor allele frequency spectrum. This was thought to reflect an initial bias toward detection of larger CNVs; that bias precludes gaining a comprehensive understanding of the contribution of this class of genetic variants to human disease and also reduces the likelihood of being able to reliably “tag” CNVs even if most were the consequence of a single (rather than recurrent) events. As technology improvements allow detection of smaller CNVs that may have a higher minor allele frequency as well as more precise delineation of the exact sequences involved, we will not only get a more accurate picture of the contribution of these variants to disease but also gain insights into the dynamics, the evolutionary history, and the consequences of such variants. A key question is whether such sites are generally uniquely created, with a single originating event, or rather are commonly regenerated, due perhaps to the presence of repetitive elements. This will determine whether such variants can be tagged and indirectly interrogated (e.g. through imputation) or will need to be directly interrogated, which will in turn influence the design of later generation platforms for GWAS.

Technology, particularly the newest sequencing technologies, was also a major topic of discussion. A number of new approaches as well as more streamlined versions of existing technologies were discussed. The meeting certainly highlighted the steady steps towards the eventual goal of sequencing entire human genomes at reasonable financial costs and with efficient computational algorithms.

***Genome-wide association scans and beyond***

**Pui-Yan Kwok** (University of California, San Francisco) went beyond the successful reports of genome-wide association scans (GWAS). He described the approaches that his group is taking in the analysis of narcolepsy, a disorder of excessive daytime sleepiness, atopy, and kidney transplantation. They have progressed in the study of these disorders and discussed issues regarding quality control of genotype data and automation in sample analysis and data production.

Considerable progress has been made in the last months on the identification of genetic variants that predispose to common human cancers. **Stephen Chanock** (National Cancer Institute, Bethesda) reported on the progress of his team in the analysis of regions that they reported previously for breast (FGFR2) and prostate cancer (on 8q24). He described the stepwise approaches used in GWAS for cancer. Ancestral recombination analysis of the 8q24 region identified independent regions of association flanking a hotspot of recombination. In addition, meta-analysis on data from several groups yielded identification of two regions on 8q24 separated by around 300 kb with highly significant association to prostate, breast, and colon cancers.

***Sequencing the individual genome: realities and challenges***

Sequencing the genome of single individuals is ongoing in different labs. One of the most challenging problems is the assembly of the sequences and the large number of differences between sequences, with many structural variation changes. **Samuel Levy** (J. Craig Venter Institute, Rockville) reported the details of the sequencing, assembly, and variant detection in the genome of Craig Venter. They used newly developed genome assembly strategies and comparative genome-to-genome mapping methods to identify 25-Mb diploid sequence differences, representing over 4 million DNA variants, of which more than one million were novel. Levy reported that non-SNP DNA variation accounts for 22% of all events identified and estimate that this non-SNP variation accounts for >75% of genetic differences. The study estimated that 0.5-1.0% of DNA sequence differs between unrelated humans (5-10 x more than previously thought). This project and others on genetic donors provide a preliminary glimpse of human sequence variation and provide insights into the much-publicized area of personalized medicine.

**Sanjeev Bhaskar** (Wellcome Trust Sanger Institute, Hinxton, Cambridge) described a pipeline for high-throughput sequencing of PCR products. They have re-sequenced exons from 14 human chromosomes in 48 Caucasian individuals (http://www.sanger.ac.uk/humgen/exoseq). He described the targeted sequencing for a variety of human diseases and traits, including epilepsy, cardiovascular disease, platelet biology, deafness, diabetes, depression and cognition.

**Ivo Gut** (Centre National de Génotypage, France) reported that his center is working on simplifying protocols for DNA sequencing using high-throughput mass spectrometers for readout. Their protocols use ribonucleotides incorporating DNA polymerases in primer extension reactions and PCR, followed by cleavage with NaOH and fingerprinting.

**George Church** (Harvard Medical School, MIT, Boston) suggested that next-generation sequencing methods that use solid-phase polymerase colonies plus sequencing-by-ligation or extension had brought down the sequencing cost over 100-fold. He showed that 1% of the genome harboring most causative alleles for medical and non-medical traits could be targeted for sequencing. He pointed out that by combining this approach with paired-end-tags for rearrangements and allele-specific RNA quantification, an affordable analysis of the human genome could be achieved at the individual level.

**Jan Korbel** (Yale University) presented the identification of a large set of structural variations in cell lines of African and European ancestry. They used mapped-end pairing and high-throughput sequencing with the 454-Roche sequencing platform. The sequence of the ends of circularized clones of around 3 kb allowed the identification of deletions, insertions and inversions, with an overlap of around 40% between the two samples. They compared the CNVs detected with those in the Database of Genomic Variants (60% overlap) and with respect to the Celera sequence (13-20% overlap) and with the fosmid end-pair sequencing performed by Evan Eichler in one of the samples (40% overlap). Overall, the approach is a clear step toward the characterization of structural variations at a high level of resolution and for the identification of the molecular mechanism(s) that produce structural variation in the genome.

**Francisco de la Vega** (Applied Biosystems) presented the new sequencing device of the company, named SOLiD™, based on oligonucleotide ligation, which was released recently. The length of reads is similar to that of the Illumina/Solexa™ system. He presented several applications of the system on amplicons and tricks on sample preparation.

***Copy number variations: from architectural changes to functional consequences***

CNV research in autism has been the focus of several groups. **Steve Scherer** (The Centre for Applied Genomics, Hospital for Sick Children and University of Toronto) reported the current view on chromosome rearrangements and imbalances in autism spectrum disorders. Full understanding of the role of CNV in autism, and indeed other complex disorders, is likely to require higher-resolution CNV scans of the genome and more family-based and population control data. Current data confirm that rare variants can contribute to autism, and only a few chromosome regions are involved in large numbers of cases (e.g. regions on chromosomes 7 and 15). Overall, chromosome rearrangements in autism are likely to be involved in between 10% and 20% of all cases. Better phenotyping as well as phenotype information in families is likely to be of value in establishing a relationship between specific rearrangements and phenotype.

Small noncoding RNAs (ncRNAs) have a key role in mRNA degradation, translational repression, heterochromatin structure and DNA removal. **Lluis Armengol** (Center for Genomic Regulation, Barcelona) reported that a new type of ncRNAs, the PIWI-interacting RNAs (piRNAs), mainly expressed in mammalian germline cells, might have a functional connection with CNVs and segmental duplications. He reported that a large proportion of piRNAs are located in CNVs and Segmental Duplications. This opens the possibility that a subset of Segmental Duplications could contain elements with a functional role in the integrity of the genome at the germline level.

**Nancy Cox** (University of Chicago) reviewed general approaches for direct (intensity-based information, utilization of genotype, call-rate, etc) and indirect assessment (linkage disequilibrium) of CNV information to study common disorders. She reported on the use of TUNA (Testing UNtyped Alleles) to utilize LD to interrogate CNVs for which multi-locus LD tags can be constructed. Combining information on LD, genotype features (such as homozygosity for deletions), local and consistent alterations in SNP call rates, and departure from Hardy-Weinberg equilibrium can be combined with intensity information to improve the ability to identify CNVs.

A key goal in studies of CNV is determining whether such variation commonly affects human phenotypes of interest, including common, complex disorders. **Iuliana Ionita (**Harvard University, Boston) reported on the development of an extension of a family-based association tests (FBAT) approach to analyze CNV data with family-based designs. The approach allows for the use of intensity information directly in the test statistic assessing the correlation between offspring intensity values and offspring phenotypes using parental intensity information to control for population admixture. A major caveat in results of studies conducted to date is the use of genotype data from platforms that lack resolution in CNV detection. It will be of great interest to see the application of the FBAT approaches described to data from the newer Affymetrix and Illumina platforms that provide better detection and resolution of CNVs.

**Don Conrad** (The University of Chicago) described new methodology for integrating CNVs into the study of genetic traits. He illustrated the utility of studying isolated groups, such as the Hutterite population for the identification and genotyping of CNVs for association studies with phenotypes such as biochemical parameters (cholesterol, triglycerides, insulin, glucose, serotonin, cortisol), blood data (eosinophilia, IgE, lymphocyte count), respiratory functional profiles (%FEV1, FEV1/FVC) and many others (height, age at menarche, etc.). He reported that T-cell lymphocyte receptor (TCR) rearrangements are inversely correlated with lymphocye count. He further noted, however, that many of TCR rearrangements reported in databases are artifacts.

CNVs have played an important role in the evolution of some gene families, notably for some receptors that have a role in the interaction of organisms with the environment. The evolution of mammalian chemosensory receptors is a nice example of this. **Barbara Trask** (Fred Hutchinson Cancer Research Center, Seattle) reported on the evolution of three families of chemosensory receptors: olfactory receptors (OR) and two classes of vomeronasal receptors (V1Rs and V2Rs). These genes families show species-specific gene duplication and loss, such that the functional repertoire varies between closely related species and even between individuals. Most striking is the catastrophic loss of V1Rs and V2Rs in primate and dog genomes. She showed that OR family size varied between species, with 800 in human, 1500 in mouse, to 3000 in cow. Most gene duplications are local, but occasional non-local duplications have distributed OR genes to over 50 locations and most chromosomes in rodents and involve segments much larger than the genes themselves. Some clusters expanded by tidy tandem duplications, while other clusters have undergone complex rearrangements, including inversions. The purifying selective pressures on rodent OR genes that have experienced recent duplication is less intense than on those that have maintained one-to-one orthology. Trask presented data indicating that the observed enrichment of ORs in human regions that varied in copy number was likely due to the neutral evolutionary consequence of such variation.

**George Perry**, from the group of Charles Lee (Brigham and Women’s Hospital, Boston) presented data about the distribution of amylase gene (AMY1) copies in different populations. His data are consistent with a positive or directional selection on AMY1 copy number in at least some high-starch populations but neutral evolution on AMY1 copy number in low-starch populations. While AMY2A and AMY2B are expressed in the pancreas, AMY1 genes are expressed in the saliva. Variability in copy number of the alpha-amylase family was described about 18 years ago, but the link with positive selection for higher AMY1 copy number in high-starch populations has not been demonstrated until now, and is consistent with an interaction between the digestion that occurs in the mouth and that happens in the intestine.

**Joris Veltman** (Radboud University Nijmegen Medical Centre, Nijmegen) presented the approach that his group used in the characterization of the genetics of mental retardation. They have used dense BAC arrays and SNP arrays to identify CNVs underlying mental retardation. The use of parallel approaches by investigators of different countries and data sharing has allowed them to identify new syndromes that were previously unrecognized. He also discussed the current and future use of different CNV analysis platforms in a clinical diagnostic setting.

**Matthew Hurles** (The Wellcome Trust Sanger Institute, Hinxton) reported on the use of short-read sequencing technologies to identify and characterise structural variation. He aligned map-paired sequence reads from either end of a single DNA molecule to a reference sequence to identify discrepant molecules. He also presented data on the development of a comprehensive map for common CNVs using high-density oligonucleotide arrays with 42 million probes across the genome. Finally, he stressed the need for improved methods for CNV-association to deal with multiallelic CNVs, and with differential biases in assessing CNVs in cases and controls.

**Jeff Gulcher** (DeCODE Genetics) reported on the deCODE strategy for the detection of CNVs using Illumina chips. The approach uses the Infinium II assay because it allows more representative amplification of the genome than standard methods based on PCR. They have identified over 15,000 non-redundant segments covering 190 Mb of sequence. These segments include mega-satellites, duplicons, unSNPable genome regions, the MHC region, and known rare and common CNVs. He showed several examples of the use of the Illumina CNV beadchip to the detection of the C4A CNV and the polymorphic D11W4 megasatellite.

One of the most difficult challenges in CNV analysis is the precise quantification of copy numbers in subjects that have several copies of a given gene or sequence. **John Armour** (Institute of Genetics, University of Nottingham) described the development of paralogue ratio tests (PRT) for high-throughput CNV genotyping. His group has used the variable *DEFB4* repeat unit on chromosome 8 versus a pseudogene *HSPDP3* on chromosome 5, allowing discrimination of between 2 and 7 copies of *DEFB4*. They have also used an LTR sequence at *CCL3L1* to compare to a chromosome 10 reference locus to enable detection of multiple copies of *CCL3L1*. Armour showed that further improvements in precision, economy and throughput for complex CNVs are needed to define their relationship with complex disorders.

Several GWAS for complex disorders have been reported. Besides the successful identification of new loci for several diseases, there are many regions that are not well covered by utilized platforms. **Xavier Estivill** (Center for Genomic Regulation, Barcelona) reported the common genomic features of disorders for which CNVs have been detected. A common feature of the identified associations between CNVs and complex/common disorders so far is the presence of segmental duplications. Moreover, all CNV loci that have been found associated with common disorders are both complex and multi-allelic. Since many CNVs are not easily tagged by SNPs, have a wide range of copy number variability, and/or fall in genomic regions not well covered by whole genome array assays, current GWAS might have missed the contribution of CNVs to complex disorders. He presented approaches of his lab to the analysis of common disorders with the identification of CNVs for psoriasis and asthma.

In the technology presentations, **Marcus Hausch** (Affymetrix) described the evolution of the arrays that the company has developed over the years and the advantages of the Genome-wide SNP 6.0 Array in the analysis of both SNPs and CNVs for association studies. Similarly, **Steve Laderman** (Agilent Technologies) described the new applications that the company has developed for structural variation analysis, including a large collection of oligonucleotide probes that they have developed for inclusion in a custom application named e-array, which allows a very flexible and cost-effective interrogation of any region of the genome.

***Allele-specific expression levels and phenotype***

Several common human disorders are caused by the variability in gene expression of specific genes. Therefore, the identification of genetic variants with regulatory effects on genes in cis or in trans could shed some light on the identification of the genetic component of human disease. **Vivian Cheung** (University of Pennsylvania, Philadelphia) reported on research to identify genetic variation affecting inter-individual gene expression. Gene-specific expression levels were used as a quantitative trait in genome-wide association studies. They have extended previously published data from 14 to 45 families. Among over 3,500 expression phenotypes they detected 235 with genome-wide significance. Most of the expression phenotypes are in *trans* (80%), some are in *cis* (5%) and about 15% have multiple effects. Interestingly, they have detected weaker effects on transcription regulation of *trans-* vs. *cis-* acting regulatory elements. She also presented data on the population differences in expression phenotypes. One of the genes with striking variability is cystatin B, a gene involved in progressive myoclonus epilepsy.

**Manolis Dermitzakis** (Wellcome Trust Sanger Institute) presented data on the widespread genetic variation in mRNA levels of many genes across populations. He performed a whole-genome gene expression study with 48,000 transcripts in the 370 samples of HapMap. He found that both *cis-* and *trans-* acting genetic variation influences mRNA levels and provided data that most *cis-*regulatory events are seen with both phase I and II of HapMap analysis, with 385 genes with *cis-*effects due to CNVs and 1395 due to SNPs. He stressed that many detected associations are shared across human populations and that signal is concentrated within 100 kb from the promoter symmetrically around transcription start sites.

***Keeping and handling information on genomic variation***

Currently, a variety of central genomic databases record information on SNPs. These databases are mostly gene-centric and provide limited information on the structural and functional consequences of SNPs. **Yum Lina** **Yip** (Swiss Institute of Bioinformatics, Geneva) presented on archiving single amino acid polymorphisms in the UniProt/Swiss-Prot knowledgebase. UniProtKB/Swiss-Prot (Swiss-Prot) (http://www.uniprot.org/) currently records more than 30,000 single amino acid polymorphisms (SAPs) in about 6,000 human proteins. The records are revised using text-mining techniques. Yip showed that Swiss-Prot does not simply catalogue amino acid changes predicted from nucleotide variations, but it stores, when available, information on direct protein sequencing and characterization including post-translational modifications. SAP information in Swiss-Prot complements other genomic and phenotypic databases, and is valuable for the understanding of SAPs and diseases.

**Andrew Devereau** (National Genetics Reference Laboratory, Manchester) reported on the use of a variation database for diagnostic molecular laboratories. Diagnostic laboratories generate variation data for genes of relevance to human health. Collection of data includes occurrences of multiple variants in a patient and the patient's phenotypic and demographic data. This allows data from different laboratories and different data sources to be integrated and analyzed for the interpretation of its clinical significance.

**Anthony Brookes** (Department of Genetics, University of Leicester, UK) presented progress towards developing HGVbaseG2P: a database of genotype-to-phenotype (G2P) relationships, which aims to pull together a comprehensive view of the world’s genetic association study findings. He also described GEN2PHEN (www.gen2phen.org): a European Commission Integrated Project designed to help provide globally relevant solutions for genotype-to-phenotype databasing. The project, which was formally launched in January 2008, will create generic G2P database software, web services, and enhanced integration capabilities for long-term support to the biomedical community.

Ensembl and NCBI constitute essential sources of genomic data. The new tools of sequencing and the information on CNVs pose new challenges for such data repositories. **Ewan Birney (**European Bioinformatics Institute, Hinxton, UK) presented an overview of the Ensembl infrastructures for genomic information, from its storage through to analysis and visualization. The data included variation information for over 6,000 human subjects and resequencing from six. Data from mouse and rat will also include resequencing of the corresponding genomes. There is also a focus on developing capacity to display association summaries for GWAS - the European Genotype Archive (EGA) project. He announced new graphic representations of CNVs in which the concept of a reference assembly will no longer be required.

**James Ostell (**National Library of Medicine, Bethesda) described several of the resources of the National Center for Biotechnology Information (NCBI). The Database of Genotype and Phenotype (dbGaP) holds phenotype data from long term clinical and cohort studies, and is linked to large-scale genotype results on the participants or to medical sequencing data in support of Genome Wide Association Studies (GWAS). The Collaboration, Education, and Test Translation (CETT) program links diagnostic information into a central research database for rare diseases.

**Lincoln Stein** (Cold Spring Harbor Laboratory, Cold Spring Harbor) presented the new features and tools of the HapMap web site and discussed progress towards providing views of resequencing data, particularly as it moves towards sequencing entire human genomes.

Micro RNAs (miRNAs) are an emerging source of possible gene regulation. We are at just the beginning of understanding the role of miRNAs in human disease. **Carole Charlier** (Centre for Biomedical Integrative Genoproteomics, University of Liège) reported on the use of Patrocles: a database of polymorphic miRNA-mediated gene regulation. Polymorphisms affecting miRNA-mediated regulation are expected to contribute to the genetic variation of traits with complex rather than Mendelian inheritance. Patrocles assists in the identification of SNPs that affect miRNA-mediated regulation.

**Lars Feuk** (The Centre for Applied Genomics, The Hospital for Sick Children, Toronto) described the latest version of the Database of Genomic Variants. They will introduce a web form for data submission and accession numbers, which should improve the accuracy of the deposited data. Challenges are definition of CNV boundaries, detection of false positives, accurate population and frequency information.

***Populations in genomic variability research***

Many regions of the genome that are involved in CNVs contain genes that have a role in adaptation to environment. **Chris** **Ponting (**University of Oxford, UK) discussed the elevated density of genes, evolutionary rates, and gene functions, noting data consistent with the possibility that some of these regions have been positively selected in the human population due to advantageous gene dosage effects of copy number variants. He presented data on large 0.5-1.0 Mb variants that exhibit an elevated G+C content, and infer that high G+C regions are unusually susceptible to copy number variation. He postulated that positive selection appears to have acted upon some of these genomic regions.

**Jaume Bertranpetit** (Pompeu Fabra University, Barcelona) discussed the possibilities of computing population recombination rates from SNP frequency data. They found that most of the variation is among major human groups and a minor component of population variation is within continents. They also found that most recombination hotspots are conserved among human populations. They detected that structure in present populations keep the footprint of the recent and fast evolving process of recombination.

After obtaining the sequence of the human genome it was claimed that there is no basis for racial differences from the biomedical point of view. However, **Esteban González-Burchard** (University of California, San Francisco) provided fundamental evidence of genetic differences between racial and ethnic populations relevant to differences in genetic risk for diseases such as Alzheimer’s disease and HIV-resistance. His group is undertaking a large study of the genetics of asthma in Latino Americans.

Differences in the population sub-structure within sample sets are potentially difficult issues in analyzing GWAS data, even in studies with individuals of European ancestry. **Gilles Thomas** (National Cancer Institute, Bethesda) presented data on population stratification in the two genome-wide studies in breast cancer and prostate cancer conducted in the Cancer Genetic Markers of Susceptibility (CGEMS) project of the National Cancer Institute initiative. After two GWAS on prospective cohorts of prostate and breast cancer they have attempted to characterize the population structure. They evaluated the continental admixture of each individuals participating in the study, detected relatedness between pairs of individuals and performed a principal components analysis of genotypes. Their results indicate reliable evidence of population structure. These observations have implication for the design of GWAS based on the participation of self described European Americans in order to best correct for population stratification in search for association.

The use of GWAS as one approach towards pharmacogenomics was presented by **Angel Carracedo** (University of Santiago de Compostela) but he also discussed more classical approaches towards identifying genetic variations associated with both toxicity and efficacy. The genetic response to drugs is a complex trait subject to the same research challenges as any other complex trait. He noted that failures in replication of pharmacogenomic findings are as common as such failures in other complex traits, but also emphasized the challenges of the current applications in clinical practice and changes in labeling that have been recommended by the regulatory agencies in Europe and USA (EMEA and FDA) for about ten different drugs.

The meeting devoted discussion to ethical aspects of individualized sequencing. **Anne Cambon-Thomsen** (Inserm and University Paul Sabatier Toulouse III, Toulouse) reviewed the issue of human biobanks for studying human genome variation. She reported on the networking of biobanks ([www.p3gconsortium.org](http://www.p3gconsortium.org/); http://www.biobanks.eu) and described some of the conflicting interests that have to be balanced, such as participant privacy, potential risks and benefits, methodological guidance for interpretation and use of data, professional recognition of investigators, sharing of samples and data, intellectual property rights, and characteristics of a centralized data repository or other repository.

The Sitges meeting also included over 150 posters presented over the three-day meeting. The general view was that each attendee came back home with new ideas in a field that is moving rapidly at the cutting edge of the discovery of the genetic variants that will define disease predisposition and should help to uncover new biological pathways for understanding human health and disease. The HGV2008 meeting will be in Toronto, Canada ([www.tcag.ca/hgv2008](http://www.tcag.ca/hgv2008)) in October 2008 with a focus on further steps into the sequencing and resequencing of the human genome.

**Acknowledgements.** Meeting support provided in part by grants from the National Human Genome Research Institute (R13 HG003953), the National Cancer Institute, the McLaughlin Centre for Molecular Medicine, the Spanish Ministerio de Educación y Ciencia (Accion Complementaria SAF-2007), Generalitat de Catalunya (ARCS1-2007-00046), and Fundación Genoma España.
